# Hemagglutinin double-mutation enhances binding of human-infecting avian influenza virus clade 2.3.4.4b H5Ny to human and SLe^X^ receptors

**DOI:** 10.1038/s44319-026-00816-2

**Published:** 2026-06-16

**Authors:** Xiyue Jin, Pu Han, Yuxuan Wang, Haichen Wang, Chunge Zhang, Antonio Di Maio, Jin Yu, Tianjiao Hao, Yuhang Gu, Zeyu Zhang, Wei Zhang, Jianxun Qi, Yuhai Bi, Xu Zhang, Lei Sun, Ningli Wang, Yan Liu, Hao Song, George F Gao

**Affiliations:** 1 CAS Key Laboratory of Pathogen Microbiology and Immunology, Institute of Microbiology, Chinese Academy of Sciences, Beijing, China; 2 Beijing Key Laboratory of Surveillance, Early Warning and Pathogen Research on Emerging Infectious Diseases, Beijing Center for Disease Prevention and Control, Beijing, China; 3 School of Life Sciences, Hebei University, Baoding, Hebei, China; 4 CAS Key Laboratory of Pathogenic Microbiology and Immunology, Institute of Microbiology, Center for Influenza Research and Early-warning (CASCIRE), CAS-TWAS Center of Excellence for Emerging Infectious Diseases (CEEID), Chinese Academy of Sciences, Beijing, China; 5 Glycosciences Laboratory, Department of Metabolism, Digestion and Reproduction, Faculty of Medicine, Imperial College London, London, UK; 6 Beijing Life Science Academy, Beijing, China; 7 School of Life Sciences, Yunnan University, Kunming, China; 8 Beijing Tongren Eye Center, Beijing Tongren Hospital, Capital Medical University, Beijing Institute of Ophthalmology, Beijing Key Laboratory of Ophthalmology & Visual Sciences, Beijing, China; 9 Department of Pathology, Beijing Ditan Hospital, Capital Medical University, Beijing, China; 10 National Key Laboratory of Intelligent Tracking and Forecasting for Infectious Diseases, Beijing Key Laboratory of Viral Infectious Diseases, Beijing Institute of Infectious Diseases, Beijing Ditan Hospital, Capital Medical University, Beijing, China; 11 National Institute for Viral Disease Control and Prevention, Chinese Center for Disease Control and Prevention, Beijing, China; 12 Present Address: National Key Laboratory of Intelligent Tracking and Forecasting for Infectious Diseases, Beijing Key Laboratory of Viral Infectious Diseases, Beijing Institute of Infectious Diseases, Beijing Ditan Hospital, Capital Medical University, Beijing, China

**Keywords:** Microbiology, Virology & Host Pathogen Interaction, Structural Biology

## Abstract

Clade 2.3.4.4b H5Ny highly pathogenic avian influenza viruses (HPAIVs) continue to circulate worldwide, posing zoonotic threats, especially with recent cattle outbreaks. The mechanisms by which these viruses adapt to mammalian hosts while maintaining a broad avian tropism remain poorly understood. Here, we demonstrate that two naturally occurring mutations (K222Q and S227R) in the hemagglutinin (HA) of a human-infecting H5N8 strain, first identified in 2020, enhance binding affinity for both α2-6-linked and Sialyl Lewis^X^ (SLe^X^) glycans, which may underlie the broad tissue binding and cross-species potential. Structural analyses reveal that these mutations expand receptor specificity for these glycans, which are abundant in the human respiratory tract and duck trachea, providing a possible molecular basis for cross-species transmission. Our findings suggest that clade 2.3.4.4b H5Ny viruses evolved dual receptor specificity as early as the 2020 Russian H5N8 strain, potentially contributing to sporadic human infections and widespread dissemination among birds and mammals.

## Synopsis

Clade 2.3.4.4b H5Ny avian influenza viruses have adapted to bind a broad range of α2-3-linked sialyl glycans like SLe^X^ in human trachea and duck intestinal tract as early as in human-infecting H5N8 in 2020, potentially responsible for ongoing sporadic human infections and wide dissemination in wild birds and poultry.

- H5N8 HA binds weakly to human α2-6 but strongly to avian α2-3 receptors.
- H5N8 HA strongly binds human conjunctiva, bovine mammary gland and duck trachea.
- H5N8 HA gains broad α2-3 recognition, notably SLe^X^, abundant in human conjunctiva and trachea.
- Naturally-occurring K222Q and S227R substitutions in HA enhance dual binding to α2-6 and SLe^X^ glycans.

## Abstract (web summary)

Clade 2.3.4.4b H5Ny avian influenza viruses have adapted to bind a broad range of α2-3-linked sialyl glycans like SLe^X^ in human trachea and duck intestinal tract as early as in human-infecting H5N8 in 2020, potentially responsible for ongoing sporadic human infections and wide dissemination in wild birds and poultry.

## Graphical abstract

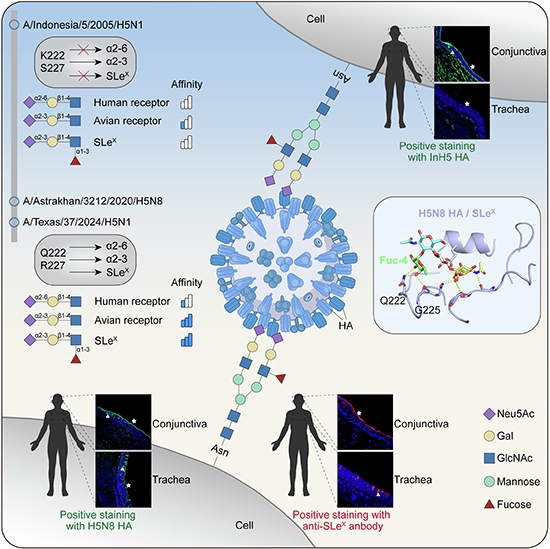

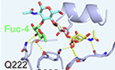

## Introduction

Since the first described HPAIV A/goose/Guangdong/1/1996 (Gs/Gd) H5N1 was identified in China in 1996, the Gs/Gd-lineage H5N1 virus has frequently reassorted with other influenza viruses and spread globally via migratory birds, posing a threat to animal and human public health. With frequent reassortments and ongoing antigenic drifts, the Gs/Gd-lineage has evolved into multiple distinct clades and subclades, among which the clade 2.3.4.4 has gradually become dominant since 2010 (Shi and Gao, 2021). Over the past decade, diverse H5Ny subtypes, including H5N1, H5N2, H5N6, and H5N8, have sporadically caused human infections with varying degrees of severity and mortality (Wang & Gao, 2025). From 2003 to 2024, a total of 954 cases with 464 deaths of influenza A (H5N1) human infections have been reported to the World Health Organization (WHO, 2024). Sustained human-to-human transmission has not yet been established, though.

In early 2024, an unprecedented outbreak of clade 2.3.4.4b HPAIV H5N1 infections in dairy herds occurred in the USA and has been reported in 985 herds in 17 states (CDC, 2025). The affected cows displayed clinical signs encompassing decreased feed intake, respiratory distress, altered fecal consistency, and sharply decreased milk production with abnormal milk (Burrough et al, 2024; Caserta et al, 2024). Infectious viruses with high titers were consistently reported in milk from the affected cows (Caserta et al, 2024; Imai et al, 2025; Kristensen et al, 2024). Although bovine H5N1 infections in cattle commonly present with mild to moderate disease, spillover to 70 farm workers has been documented as of March 10, 2025, resulting mostly in conjunctivitis or mild respiratory symptoms (CDC, 2025; Garg et al, 2025; Gu et al, 2024; Morse et al, 2024; Mostafa et al, 2025). Notably, experimental studies of a human-derived bovine H5N1 isolate, A/Texas/37/2024 (TxH5N1), proved lethal in mice and ferrets and spread systemically as well as partial transmission via respiratory droplets in ferrets (Gu et al, 2024; Pulit-Penaloza et al, 2024). A key element of this adaptation is the acquisition of a dual receptor-binding capability, retaining a strong preference for avian-like α2-3-linked sialic acid receptors while gaining the capacity to weakly bind human-like α2-6-linked sialyl glycans (Eisfeld et al, 2024; Gu et al, 2024; Song et al, 2025). Immunohistochemical staining demonstrated that TxH5N1 HA exhibited specific binding to bovine pulmonary and mammary epithelia as well as human conjunctival, tracheal, and mammary tissues, consistent with observed clinical manifestations (Song et al, 2025). Besides, the PB2-E627K substitution in TxH5N1 and the PB2-M631L substitution for the bovine isolates could have independently conferred virus mammalian adaptation by increasing viral polymerase activity in mammalian cells (Gu et al, 2024).

The origin of the currently epidemic clade 2.3.4.4b HPAIV H5N1 is traced back to a novel reassortant between H5N8 clade 2.3.4.4b and low-pathogenicity avian influenza virus (LPAIV) circulating in European wild birds (Bi et al, 2024; Xie et al, 2023). The viruses were introduced to North America in late 2021 through the transatlantic flyway from Europe. The outbreak of 2020-2021 2.3.4.4b H5N8 has significantly impacted poultry production, resulting in billions of dollars in losses for the sector since its emergence. Notably, the first human infections with H5N8 were reported in December 2020, when seven poultry workers in Russia contracted the virus (Pyankova et al, 2021). In November 2020, an outbreak of H5N8 avian influenza virus infection in whooper swans occurred in the Yellow River Reservoir region of China. The noticeable symptoms included weakness, cloudy eyes, and labored breathing. In addition, several deaths among the whooper swans were recorded (Li et al, 2021). Phylogenetic tree analysis showed that the strains from dead whooper swans and the infected workers both belong to the subclade 2.3.4.4b H5N8 and were closely related (Li et al, 2021). While significant progress has been made in understanding the receptor-binding properties of recent bovine H5N1 and its capacity to infect humans, the receptor-binding characteristics of H5N8, particularly the strain responsible for human infections, remain unclear. This study aims to address this gap by examining the receptor-binding properties of the first human-isolated H5N8 strain (A/Astrakhan/3212/2020, huH5N8), together with the whooper swan-infected H5N8 strain (A/whooper swan/Henan/CAS001-K/2020, wsH5N8), to better understand its potential for human infection and the broader epidemic risks.

In this study, we assessed the receptor-binding properties and tissue tropism of HA proteins from both huH5N8 and wsH5N8. We also elucidated the molecular mechanisms of receptor binding by determining the structures of the H5 in complex with receptor analogs. Our findings show that both H5N8 and bovine H5N1 have acquired dual receptor specificity, recognizing both α2-6-linked sialic acids and SLe^X^ glycans. This capability is partially conferred by naturally occurring amino acid substitutions K222Q and S227R in the HA protein. While these data suggest that such adaptation may enhance the ability to infect a range of hosts and could facilitate sporadic human infections, further studies are needed to directly evaluate the contribution of these mutations to the transmission of clade 2.3.4.4b H5Ny viruses.

## Results

### Receptor-binding specificity of huH5N8 and wsH5N8 HAs

**Figure EV1:**
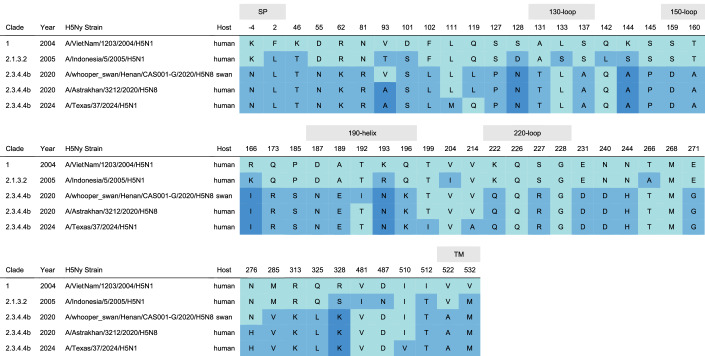
Sequence alignment of huH5N8, wsH5N8, TxH5N1, and early H5Ny HAs. Sequence alignment of huH5N8, wsH5N8, InH5, VN1203 and TxH5N1 HAs. Signal peptide region, secondary structural elements of the binding site (i.e., the 130-loop, 150-loop, 190-helix, and 220-loop), and transmembrane region are labeled.

The HA protein sequences of huH5N8 and wsH5N8 were aligned with those from the first human infection case caused by a bovine-derived H5N1 virus (A/Texas/37/2024, TxH5N1) (Uyeki et al, 2024). All three belong to clade 2.3.4.4b and share a conserved receptor-binding site (RBS). While distinct amino acid variations exist within the HA ectodomain between huH5N8 and TxH5N1 (positions 111, 119, 199, 214; H3 numbering) and between huH5N8 and wsH5N8 (positions 93, 192, 276), the conserved A160 substitution (observed in all three lineages) disrupts the N158 glycosylation motif, conferring increased binding avidity to α2-6-linked sialic acid receptors (Fig. EV1) (Gao et al, 2018). They also retain Q226 and G228 within the RBS (Fig. EV1).

**Figure 1:**
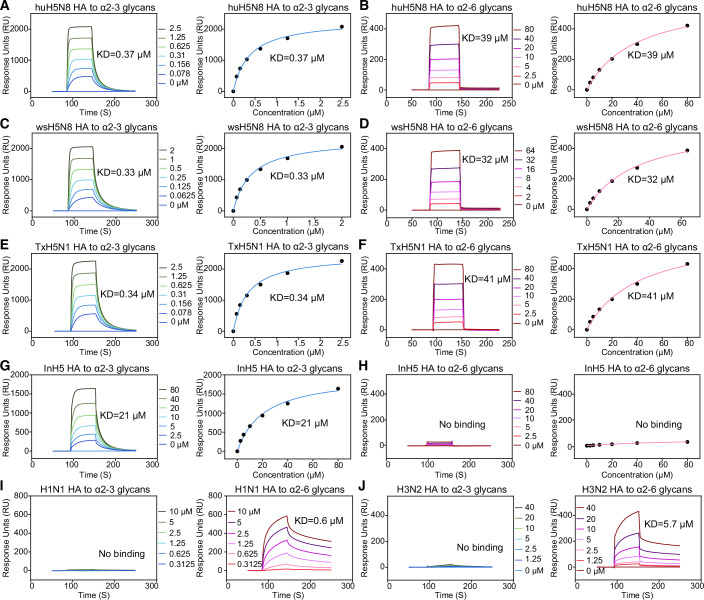
Receptor-binding properties of huH5N8 and wsH5N8 HAs at the protein level. BIAcore diagrams showing binding of huH5N8 HA to 3′SLNLN (**A**) and 6′SLNLN (**B**); binding of wsH5N8 HA to 3′SLNLN (**C**) and 6′SLNLN (**D**); binding of TxH5N1 HA to 3′SLNLN (**E**) and 6′SLNLN (**F**). Response units were plotted against protein concentrations. As controls, InH5 HA specifically binds 3’SLNLN (**G**, **H**), whereas H1N1 (**I**) and H3N2 (**J**) HA specifically bind 6’SLNLN. The plots and KD values are representative data from three biological replicates. Source data are available online for this figure.

To assess receptor-binding affinities, we produced recombinant HAs from huH5N8 and wsH5N8 using a baculovirus expression system and performed surface plasmon resonance (SPR) assays with canonical avian (α2-3-linked) and human (α2-6-linked) sialic acid receptor analogs. The huH5N8 HA exhibited slight binding to the human receptor analog with a KD (dissociation constant) of 39 μM while maintaining strong binding to the avian receptor analog with a KD of 0.37 μM (Fig. 1A,B). Similarly, the wsH5N8 HA bound to the avian receptor analog with high affinity of 0.33 μM (Fig. 1C), and showed slight binding to the human receptor analog with an affinity of 32 μM (Fig. 1D). These findings align with our previous characterization for TxH5N1 (Fig. 1E,F) (Song et al, 2025). Though the huH5N8, wsH5N8, and TxH5N1 we studied here possess T160A but still maintain Q226, they obtained a dual receptor-binding paradigm, acquiring slight binding to human-like α2-6-linked sialic acids and retaining a strong preference for avian-like α2-3-linked receptors (Fig. 1A–F). In contrast, the previous H5N1 strain A/Indonesia/5/2005 (InH5) showed exclusive avian receptor preference (KD = 21 μM) with undetectable human receptor binding (Fig. 1G,H) (Zhang et al, 2013a). Both H1N1 (A/South Carolina/1/1918) and H3N2 (a seasonal influenza strain, A/Kansas/14/2017) HAs exhibit high-affinity binding to the human receptor analog, with no detectable binding to the avian receptor analog (Fig. 1I,J), confirming the specificity and consistency of the SPR assays.

### Receptor binding analysis of H5N8 HA using glycan microarray

**Figure 2:**
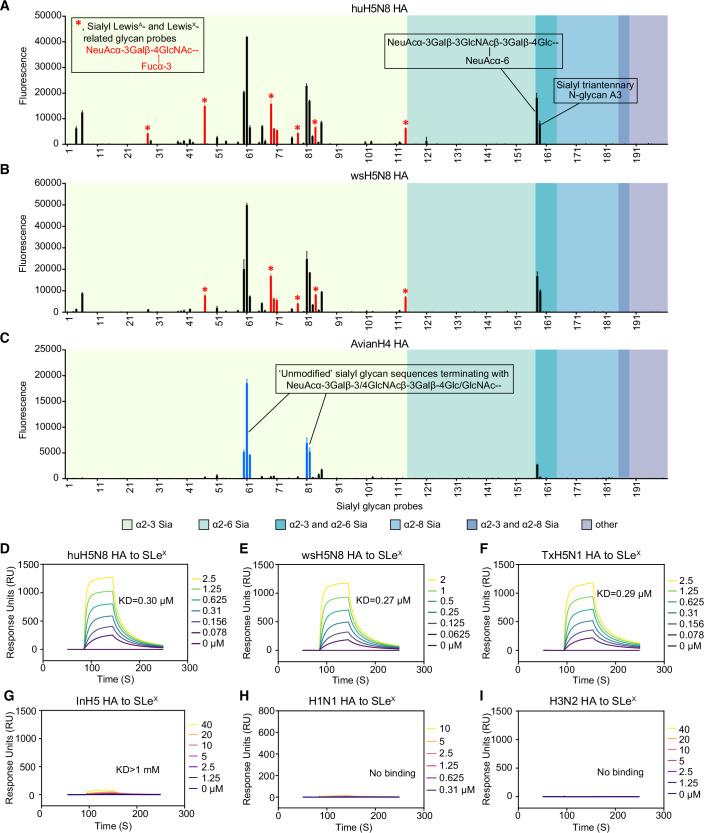
HuH5N8 and wsH5N8 HAs obtain a high affinity for SLe^X^ in comparison to early H5Ny. (**A**–**C**) Binding of huH5N8 (**A**), wsH5N8 (**B**), and AvianH4 (**C**) to the 200 sialyl glycan probes within the broad-spectrum glycan screening arrays is shown. Error bars represent half the difference in signal intensities between duplicate spots of each glycan probe. The various types of terminal sialic acid linkages are indicated by the colored panels as defined at the bottom of the figure. The full glycan array data are available in Appendix Fig. S1 and Dataset EV1. A detailed comparison of binding to selected sialyl glycan probes by the three HAs, along with the corresponding glycan sequences, is provided in Dataset EV2. (**D**–**I**) BIAcore diagrams showing binding of huH5N8 (**D**), wsH5N8 (**E**), TxH5N1 (**F**), InH5 (**G**), H1N1 (**H**), and H3N2 (**I**) HA to SLe^X^. The plots and KD values are representative data from three biological replicates. Source data are available online for this figure.

To systematically characterize the glycan receptor binding profiles of huH5N8 and wsH5N8 strains, we conducted glycan microarray analyses using HA proteins. The study incorporated a control avian strain, A/duck/Czech/1956 (AvianH4), previously shown to bind avian-type but not human-type receptors (Song et al, 2017). All three HAs (huH5N8, wsH5N8, and AvianH4) exclusively bind to α2-3-linked sialyl receptors, with no detectable interaction with α2-6-linked sialic acid glycans (Fig. 2A–C) or non-sialylated glycans included in the comprehensive screening arrays (Appendix Fig. S1). This binding pattern contrasts with previous SPR data (Fig. 1A–F), where weak human receptor analog binding was observed. The apparent discrepancy likely reflects methodological differences in protein concentrations: the microarray analyses used 50 μg/mL of HA, while SPR utilized 4.8 mg/mL. Notably, huH5N8 and wsH5N8 HAs displayed broader α2-3-linked receptor recognition than AvianH4, particularly showing enhanced binding to fucose-modified α2-3-linked sialyl glycans (Sialyl Lewis^A^- and Lewis^X^- related probes), highlighted in Fig. 2A–C. The results with SLe^X^ and its sulfated analogs were of particular interest, as they have been extensively studied for influenza virus recognition (Gambaryan et al, 2012; Gambaryan et al, 2008; Liu et al, 2010; Xiong et al, 2013). Both H5N8 HAs preferentially bound SLe^X^-Le^X^-terminating glycolipid (probe 84) over its non-fucosylated analog (probe 83), suggesting fucose enhances binding affinity. Surprisingly, sulfated SLe^X^ (probe 78) showed no binding enhancement compared to non-sulfated analog (probe 71), differing from patterns observed in poultry viruses (Gambaryan et al, 2012; Gambaryan et al, 2008) and the 2009 pandemic H1N1 viruses (Liu et al, 2010). These findings indicate H5N8 HAs have evolved expanded α2-3-linked sialic acid receptor recognition, particularly for SLe^X^-related glycans.

SPR quantification revealed distinct SLe^X^ binding capacities among H5Ny hemagglutinins. The HAs of the recently 2.3.4.4b strains (huH5N8, wsH5N8, and TxH5N1) exhibited a high affinity for SLe^X^ (KD = 0.3 μM), in stark contrast to InH5, which demonstrated negligible binding (KD > 1 mM) (Fig. 2D–G). The HAs of H1N1 and H3N2 showed undetectable binding to SLe^X^, as controls (Fig. 2H,I). Previous research indicated that the affinity of the classic VN1194 H5 for SLe^X^ is ~1/4 of that for 3’SLN, likely due to steric hindrance of K222 to Fuc-4 (Xiong et al, 2013). In this study, we found that the affinity of recent strains, including huH5N8, wsH5N8, and TxH5N1 HAs, for SLe^X^ is comparable to that for 3’SLNLN (Figs. 1A,C,E and 2D–F). Collectively, our results suggest that, at least since the emergence of the 2020 H5N8 viruses, the 2.3.4.4b H5Ny strains have acquired specific recognition of fucosylated α2-3-linked sialyl glycans (particularly SLe^X^), indicating ongoing adaptive evolution.

### Host tissue tropism of huH5N8 and wsH5N8 HAs

**Figure EV2:**
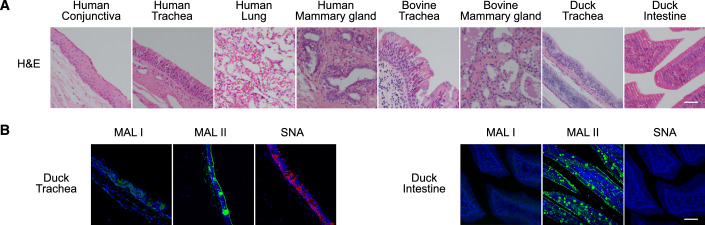
Representative images of H&E staining and staining of duck tissue sections with lectin. (**A**) Representative images of H&E staining. (**B**) Staining of duck tissue sections with lectin. MAL-I, MAL-II, and SNA staining of duck trachea and intestine tissue sections. Specific staining areas are green for MAL-I or MAL-II staining and red for SNA staining. Scale bar: 50 μm. MAL Maackia amurensis lectin, SNA Sambucus nigra lectin. Representative results from at least two independent experiments are shown.

**Figure 3:**
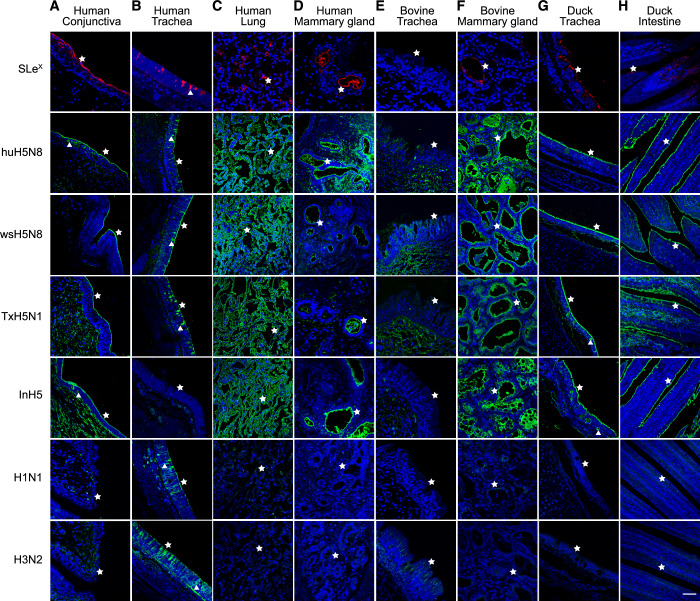
Host tissue tropism of H5N8 HAs and widespread distribution of SLe^X^ in tissues. (**A**–**D**) Staining of human conjunctiva (**A**), trachea (**B**), lung (**C**), and mammary gland tissue sections (**D**) with huH5N8, wsH5N8, TxH5N1, InH5, H1N1, H3N2 HA proteins, and anti-SLe^X^ antibodies. White triangles indicate goblet cells in (**A**, **B**); white pentagrams indicate conjunctival epithelium in (**A**), respiratory epithelium in (**B**), alveolar epithelium in (**C**), and mammary glandular epithelium in (**D**). (**E**, **F**) Staining of bovine trachea (**E**) and mammary gland (**F**) with the same HA proteins and anti-SLe^X^ antibodies. White pentagrams indicate respiratory epithelium in (**E**), and mammary glandular epithelium in (**F**). (**G**, **H**) Staining of duck trachea (**G**) and intestine (**H**) with the same HA proteins and anti-SLe^X^ antibodies. White triangles indicate goblet cells in (**G**); white pentagrams indicate respiratory epithelium in (**G**) and intestinal epithelium in (**H**). Scale bar: 50 μm. Representative results from at least two biological replicates are shown. Source data are available online for this figure.

To assess the tissue tropism profiles of huH5N8 and wsH5N8, as we recently analyzed for TxH5N1 (Song et al, 2025), we conducted immunohistochemical staining on paraffin-embedded human, bovine, and duck tissues using soluble recombinant HA proteins. The H&E staining was conducted to assess tissue quality (Fig. EV2). Both huH5N8 and wsH5N8 HAs demonstrated robust binding comparable to TxH5N1 HA across multiple human tissues, including human conjunctiva (Fig. 3A), trachea (Fig. 3B), lung (Fig. 3C), and mammary gland (Fig. 3D). These bindings were particularly pronounced in the stratified columnar epithelium of the conjunctiva (Fig. 3A), the tracheal epithelial pseudostratified ciliated columnar epithelium (Fig. 3B), the alveolar cells (Fig. 3C), and the mammary gland alveolar epithelium (Fig. 3D). Notably, these HAs also exhibited significant affinity for bovine mammary gland (Fig. 3F) and duck tracheal/intestinal tissues (Fig. 3G,H). In contrast, the early InH5 showed strong binding to most tissues except the human trachea. H1N1 and seasonal H3N2 HAs, known for their preferential recognition of human-like receptors, displayed poor binding across tissues aside from the human trachea (Fig. 3). Interestingly, none of the tested HAs bound effectively to bovine trachea (Fig. 3E). Collectively, huH5N8 and wsH5N8 HAs exhibited the broadest tissue tropism among the tested HAs, mirroring TxH5N1 HA, in both binding intensity and multi-species tissue penetration capacity.

We have recently characterized the receptor distribution in bovine and human tissues (Song et al, 2025). To investigate receptor distribution in duck tissues, we performed lectin staining using Maackia amurensis lectin (MAL) and Sambucus nigra lectin (SNA). MAL specifically binds to α2-3-linked sialyl glycans, whereas SNA binds to α2-6-linked sialyl glycans. MAL comprises two isotypes: MAL-I and MAL-II. MAL-I primarily targets SAα2-3Galβ1-4GlcNAc found in both N-glycans and O-glycans, while MAL-II specifically recognizes SAα2-3Galβ1-3GalNAc in O-glycans (Geisler and Jarvis, 2011). Our results showed that duck intestines showed predominant avian-type receptor expression, while tracheal tissues exhibited both avian and human-type receptors (Fig. EV2).

We further mapped SLe^X^ distribution across species using immunofluorescence (Fig. 3). The analysis revealed concentrated SLe^X^ signals in human conjunctiva (Fig. 3A), trachea (Fig. 3B), lung (Fig. 3C), and mammary gland (Fig. 3D), indicating robust SLe^X^ expression in these tissues. Notably, bovine specimens exhibited mammary gland SLe^X^ expression (Fig. 3F), contrasting with its absence in tracheal sections (Fig. 3E). Furthermore, avian tissues demonstrated SLe^X^ enrichment in both the trachea (Fig. 3G) and intestinal villi (Fig. 3H) of ducks. Collectively, the conserved expression of SLe^X^ across tissues in diverse species partially explains its unusually broad tissue tropism, which includes ocular, respiratory, and glandular systems.

### Structural basis of huH5N8 and wsH5N8 HAs receptor binding

**Figure EV3:**
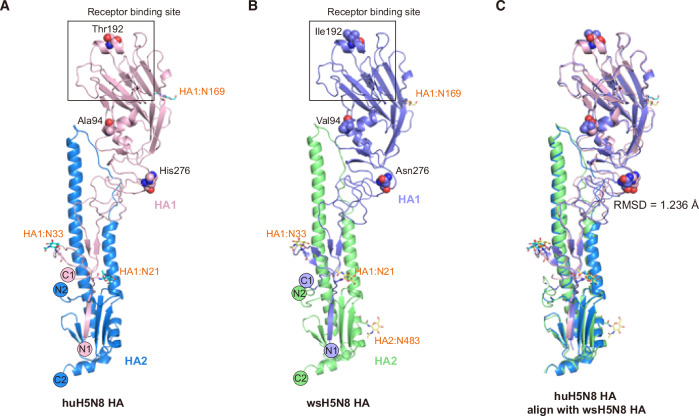
Structural comparison of huH5N8 HA and wsH5N8 HA monomer. (**A**, **B**) Cartoon representation of the huH5N8 HA and wsH5N8 HA monomer structure. HA1 is colored in light pink and HA2 in marine in huH5N8 HA, while HA1 is colored in slate and HA2 in lime in wsH5N8 HA (The N termini and C termini of HA1 and HA2 are labeled in their respective colors.). The three amino acids different between huH5N8 HA and wsH5N8 HA (position 94, 192, and 276) were presented in spheres. N-glycosylation sites and N-linked glycans (cyan in huH5N8 and pale yellow in wsH5N8) are highlighted in sticks and numbered at the Asn attachment site. (**C**) Comparison of the overall structure of huH5N8 HA and wsH5N8 HA. The RMSD is 1.744 Å for the trimer and 1.236 Å for one protomer.

High-resolution crystal structures of native HA trimers were determined to 3.19 Å (huH5N8) and 2.59 Å (wsH5N8) resolutions (Appendix Tables S1 and S2). Despite three residue substitutions (A93V, T192I, H276N; Fig. EV3), they maintained near-identical structure, evidenced by low RMSD values of 1.744 Å (trimeric assembly) and 1.236 Å (monomeric protomer) through structural superimposition.

**Figure EV4:**
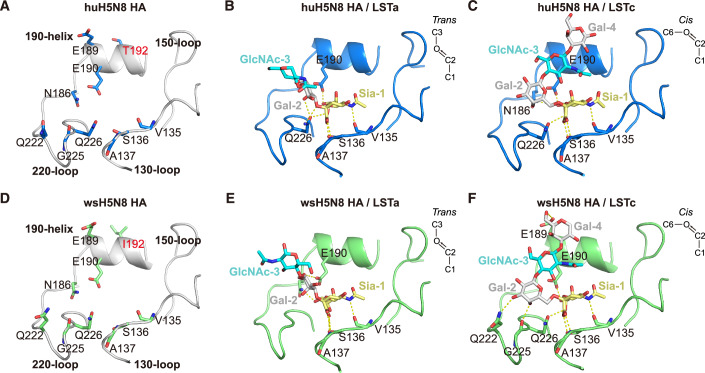
Molecular interactions of huH5N8 HA and wsH5N8 HA with either avian or human receptor analogs. Schematic representation of: (**A**) huH5N8 HA RBS with sticks representing key receptor-binding residues (colored in marine); (**B**) huH5N8 HA with LSTa bound; (**C**) huH5N8 HA with LSTc bound; (**D**) wsH5N8 HA RBS with sticks representing key receptor-binding residues (colored in lime); (**E**) wsH5N8 HA RBS with LSTa bound; and (**F**) wsH5N8 HA RBS with LSTc bound. The structure of the four secondary structural elements of the binding site (i.e., the 130-loop, 150-loop, 190-helix, and 220-loop) is labeled in ribbon representation, together with selected residues in stick representation. The hydrogen bonds are shown as dashed lines. The Sia-1 moiety of the receptor analogs is colored in pale yellow, the Gal-2 and Gal-4 moieties are colored in gray, and the GlcNAc-3 moiety is colored in aquamarine.

**Figure 4:**
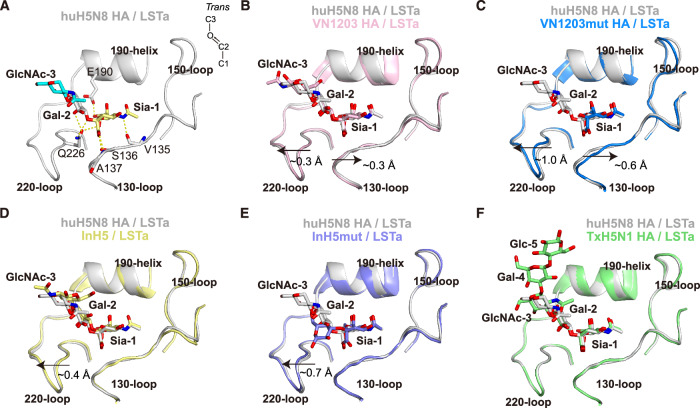
Structural comparison of huH5N8 HA with LSTa to other H5 HAs bound to LSTa. (**A**) Schematic representation of huH5N8 HA RBS with LSTa bound, with sticks representing key residues for receptor binding. Structures of the four secondary structural elements of the binding site (i.e., the 130-loop, 150-loop, 190-helix, and 220-loop) are labeled in ribbon representation, together with selected residues in stick representation. The hydrogen bonds are shown as dashed lines. The Sia-1 moiety of the receptor analogs is colored in pale yellow, the Gal-2 moiety is colored in gray, and the GlcNAc-3 moiety is colored in aquamarine. (**B**) Comparison of RBSs of huH5N8 HA/LSTa (gray) and VN1203 HA/LSTa (light pink; PDB code: 4BGY) complexes. (**C**) Comparison of huH5N8 HA/LSTa (gray) and VN1203mut/LSTa (marine; PDB code: 4KDN) complexes. (**D**) Comparison of huH5N8 HA/LSTa (gray) and InH5/LSTa (pale yellow; PDB code: 4K63) complexes. (**E**) Comparison of huH5N8 HA/LSTa (gray) and InH5mut/LSTa (slate; PDB code: 4K66) complexes. (**F**) Comparison of huH5N8 HA/LSTa (gray) and TxH5N1/LSTa (lime; PDB code: 9DIP) complexes. Source data are available online for this figure.

**Figure EV5:**
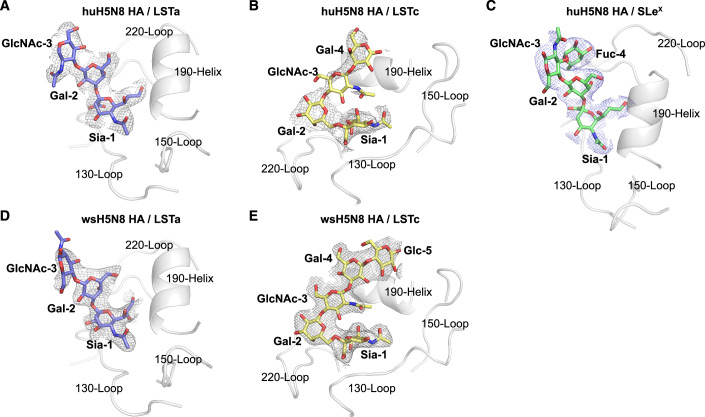
2Fo-Fc maps for the glycan receptors in the H5N8 HA/receptor complex structures. HuH5N8 HA with LSTa (**A**), LSTc (**B**), and SLe^X^ (**C**), and wsH5N8 HA with LSTa (**D**) and LSTc (**E**). The panels show sections of the 2Fo-Fc electron density maps contoured at 0.8 sigma. The figures were drawn using PyMOL software.

To elucidate the molecular determinants of avian receptor bias, we determined high-resolution structures of huH5N8 HA (3.12 Å) and wsH5N8 HA (2.61 Å) in complex with LSTa, a canonical α2-3-linked sialoside analog (Appendix Tables S1 and S2). Both complexes exhibited well-defined electron density for the terminal trisaccharide motif (Sia1-Gal2-GlcNAc3) (Fig. EV5A,D). The number of contacts between huH5N8 HA and LSTa is comparable to the number of contacts between wsH5N8 HA and LSTa (Appendix Table S4), suggesting a similar efficient binding mode for LSTa with both huH5N8 and wsH5N8 HAs. The T192I substitution between huH5N8 and wsH5N8 HAs is away from ligands (Fig. EV4), which was reported in enhancing human receptor binding in A/Thailand/1(KAN-1)/2004 (Yang et al, 2007). Both LSTa molecules in the H5N8 HAs adopted the typical *trans* conformation (Fig. 4A), similar to classic avian-adapted HAs (Zhang et al, 2013a) and TxH5N1 (Song et al, 2025) (Fig. 4F). Structural alignment demonstrated that the binding mode in huH5N8/LSTa complex closely resembles that of earlier InH5 and VN1203, rather than airborne-transmissible mutants (InH5mut containing H110Y/T160A/Q226L/G228S; VN1203mut containing N158D/N224K/Q226L/T318I; H3 numbering) (Fig. 4B–E). Importantly, the RBS region of huH5N8 HA is wider than that of InH5 and VN1203 (Fig. 4B,D), suggesting an evolutionary adaptation for enhanced binding to receptors. HuH5N8 maintained equivalent RBS width to TxH5N1 (Fig. 4F), consistent with the similar binding avidity (Fig. 1A,E).

**Figure 5:**
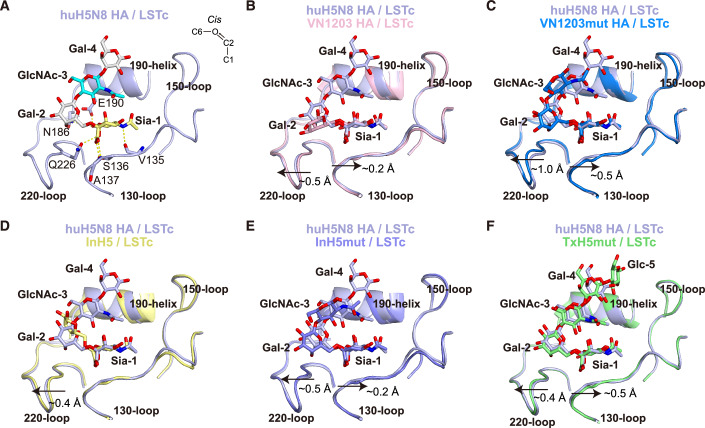
Structural comparison of huH5N8 HA with LSTc to other H5 HAs bound to LSTc. (**A**) Schematic representation of huH5N8 HA RBS with LSTc bound, with sticks representing key residues for receptor binding. Structures of the four secondary structural elements of the binding site (i.e., the 130-loop, 150-loop, 190-helix, and 220-loop) are labeled in ribbon representation, together with selected residues in stick representation. The hydrogen bonds are shown as dashed lines. The Sia-1 moiety of the receptor analogs is colored in pale yellow, the Gal-2 and Gal-4 moieties are colored in gray, and the GlcNAc-3 moiety is colored in aquamarine. (**B**) Comparison of RBSs of huH5N8 HA/LSTc (light blue) and VN1203 HA/LSTc (light pink; PDB code: 4BGX) complexes. (**C**) Comparison of huH5N8 HA/LSTc (light blue) and VN1203mut/LSTc (marine; PDB code: 4KDO) complexes. (**D**) Comparison of huH5N8 HA/LSTc (light blue) and InH5/LSTc (pale yellow; PDB code: 4K64) complexes. (**E**) Comparison of huH5N8 HA/LSTc (light blue) and InH5mut/LSTc (slate; PDB code: 4K67) complexes. (**F**) Comparison of huH5N8 HA/LSTc (light blue) and TxH5N1mut/LSTc (lime; PDB code: 9DIO) complexes. Source data are available online for this figure.

In contrast to the *trans* conformation observed with H5N8/LSTa complex, LSTc (the canonical α2-6-linked sialic analog) adopts a *cis* conformation in which its glycan chain folds back toward the 220-loop (Figs. 5A and EV4). The crystal structures of huH5N8 and wsH5N8 HAs in complex with LSTc, solved at 3.05 Å and 2.48 Å resolution, respectively (Appendix Tables S1 and S2), reveal comparable hydrogen bonds with those of LSTa (Appendix Table S4). The Gal-2/GlcNAc-3/Gal-4 moieties fold back and pass through the 190-helix both in the huH5N8 HA/LSTc and wsH5N8 HA/LSTc complex structures (Figs. 5A and EV4). A key determinant is Gln226, which forms a hydrophilic microenvironment that favors α2-3-linked sialic acids but partially repels the nonpolar portion of α2-6-linked glycan chains. Even so, N186, Q222, and Q226 help draw the LSTc glycan closer to the 220-loop, stabilizing this *cis* conformation and modestly enhancing LSTc binding affinity. Structural comparisons of huH5N8/LSTc with various H5N1/LSTc complexes, including both earlier InH5 and VN1203 strains, as well as airborne-transmissible mutants (InH5mut, VN1203mut) (Lu et al, 2013; Zhang et al, 2013a), showed that huH5N8 aligns more closely with the mutant binding modes (Fig. 5B–E). Interestingly, structural alignment revealed similar *cis* conformations between huH5N8/LSTc and human-receptor-preferential TxH5N1mut/LSTc (Q226L) (Lin et al, 2024), though the RBS region of TxH5N1mut HA is wider by 0.9 Å (Fig. 5F). Retention of Gln226 and Gly228 underscores H5N8’s predominant affinity for avian-like receptors and its limited α2-6-linked glycans recognition capacity. Meanwhile, the limited mutations in the 220-loop and 190-helix suggest these H5N8 HAs, and TxH5N1 are at an early stage of α2-6-linked sialyl glycans receptor adaptation, compared with mutated strains, like the InH5mut HA.

Collectively, the complex structures demonstrate that the recently 2.3.4.4b H5Ny HA binds to human receptors in a manner that closely resembles the binding mode of airborne-transmissible H5N1 mutants (Fig. 5B–F). In contrast, engagement with avian receptors maintains the canonical binding geometry observed in avian-adapted HAs (Fig. 4B–F). This structural plasticity suggests an evolutionary trajectory in which partial adaptation to human receptors occurs without a complete loss of avian host specificity.

### Structural basis of the enhanced affinity of H5N8 HA to SLe^X^

**Figure 6:**
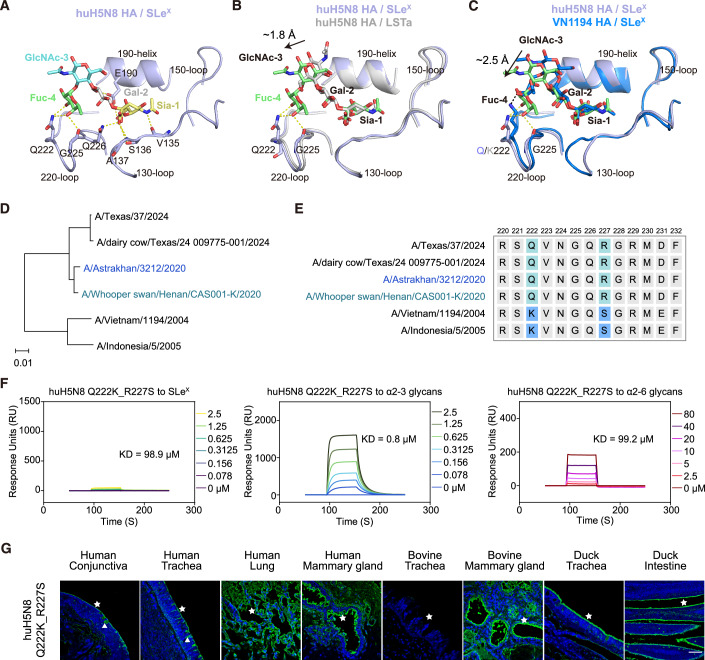
Structural basis of the enhanced affinity of huH5N8 HA to SLe^X^. (**A**) Structure analysis of huH5N8 HA in complex with SLe^X^. The Sia-1 moiety of the receptor analogs is colored in pale yellow, the Gal-2 moiety is colored in gray, the GlcNAc-3 moiety is colored in aquamarine, and the Fuc-4 moiety is colored in lime. The hydrogen bonds are shown as dashed lines. (**B**) Comparison of RBSs of huH5N8 HA/SLe^X^ (light blue) on huH5N8 HA/LSTa (gray). The SLe^X^ in huH5N8 is colored in lime. (**C**) Comparison of RBSs of huH5N8 HA/SLe^X^ (light blue) on VN1194 H5N1 HA/SLe^X^ complexes (marine, PDB code: 3ZNM). The SLe^X^ in huH5N8 HA is colored in lime, while the SLe^X^ in VN1194 is colored in marine. The hydrogen bonds are shown as dashed lines. (**D**) Phylogenetic tree of the representative HAs from different H5N1 strains. (**E**) Sequence alignment of huH5N8, wsH5N8, InH5, VN1194 and TxH5N1 HAs. (**F**) BIAcore diagrams showing binding of huH5N8 Q222K_R227S to SLe^X^, 3’SLNLN, and 6’SLNLN. The plots and KD values are representative data from three independent experiments. (**G**) HuH5N8 Q222K_R227S HA proteins staining human conjunctiva, human trachea, human lung, human mammary gland, bovine trachea, bovine mammary gland, duck trachea, and duck intestine tissue sections. White triangles indicate goblet cells, and white pentagrams indicate conjunctival epithelium. Scale bar: 50 μm. Representative results from at least two biological replicates are shown. Source data are available online for this figure.

To elucidate the binding mechanism, we determined the cryo-EM structure of the complex of huH5N8 HA and SLe^X^ at 2.62 Å resolution (Appendix Table S3). The electron density map clearly revealed Sia-1, Gal-2, GlcNAc-3, and fucose (Fuc-4) positions, with all sugar rings except for GlcNAc-3 directly interacting with the RBS (Fig. EV5C). Compared to the huH5N8/LSTa complex, SLe^X^ formed two additional hydrogen bonds (Appendix Table S4). Gln222 and Gly225 established three hydrogen bonds with Fuc-4 (Fig. 6A), pulling the GlcNAc-3 glycan ring closer to the 220-loop by ~1.8 Å. This movement repositioned Gal-2’s hydrophilic glycosidic oxygen about 0.9 Å toward the 190-helix. Consequently, this led to the weakening of the hydrogen bonds between Gln226 and Gal-2, while enabling the formation of a new hydrogen bond between Glu190 and the O6-hydroxyl of Gal-2 (3.3 Å) compared to the huH5N8/LSTa complex (Fig. 6B; Appendix Table S4). A comparison with the earlier VN1194 H5 HA/SLe^X^ complex (PDB: 3ZNM) revealed that VN1194 forms only one hydrogen bond between Fuc-4 and K222(Xiong et al, 2013), whereas huH5N8 HA establishes three hydrogen bonds via Q222 and G225, drawing Fuc-4 ~2.5 Å closer to the 220-loop (Fig. 6C). Consistent with our results, Weber et al, observed that the fucose forms additional hydrogen bonds with Q222 and G225 residues in a 2.3.4.4b strain (A/duck/France/161108 h/2016) (Weber et al, 2026), further validating the key role of fucose-mediated contacts in SLe^X^ recognition by H5Ny HAs. These structural findings, consistent with SPR data (Fig. 2), demonstrated enhanced SLe^X^ binding in huH5N8 through fucose-mediated interactions.

### Q222K and R227S double mutations reduce α2-6-linked sialyl glycans binding and significantly diminish SLe^X^ binding

To identify key amino acids that enhance receptor binding, we performed evolutionary and sequence analyses of HAs. Phylogenetic analysis showed that the VN1194 and InH5 are closely related, while huH5N8 and TxH5N1 also share a close relationship (Fig. 6D). Sequence alignment revealed strict conservation at position 225, whereas positions 222 and 227 exhibited evolutionary divergence (Fig. 6E). Notably, concurrent K222Q and S227R substitutions emerged in both huH5N8 and the recent TxH5N1 strain. To assess their functional relevance, we introduced the Q222K and R227S double mutations into huH5N8 HA and performed SPR assays. Strikingly, the huH5N8 Q222K_R227S variant displayed a reduction in receptor-binding affinities, with a 2-fold decrease for human receptor analogs, a twofold decrease for avian receptors, and a more than 300-fold decrease for SLe^X^ (Fig. 6F). These results indicate that subtle 220-loop changes can dramatically alter receptor specificity. The natural K222Q and S227R substitutions in huH5N8, wsH5N8, and TxH5N1 HAs are therefore critical for dual binding to both human-like receptors and SLe^X^.

We further examined the tissue tropism of huH5N8 Q222K_R227S HA. Despite reduced binding affinity, huH5N8 Q222K_R227S HA maintained a binding profile similar to wild-type huH5N8 HA across multiple tissues (Fig. 6G), including the conjunctiva, trachea, lung, and mammary gland in humans, as well as bovine mammary gland, duck tracheal, and duck intestinal tissues. Also, the bovine trachea showed poor staining with the mutant HA. Most tissues retained abundant avian receptors, while the human trachea exhibited strong expression of human receptors with a minor avian receptor presence (Song et al, 2025). Because affinities for α2-3 and α2-6 receptors decreased by only half, respectively, huH5N8 Q222K_R227S HA still stains well with these tissues.

## Discussion

The global epidemic of H5 HPAIV has entered a new phase after 2014, as 2.3.4.4 clade viruses carrying multiple neuraminidase subtypes (N2, N6, N8) expanded from a South Korea focus to a pan-continental phenomenon (Global Consortium for H5N8 and Related Influenza Viruses, 2016). Particularly, the 2.3.4.4b H5N8 subtype established an intercontinental transmission through migratory bird flyways spanning East Asia, Europe, and Africa. This viral lineage persists through multiple epidemic cycles, with recurrent outbreaks documented during 2016/2017 and 2020/2021 in both domestic poultry and wild avifauna. The currently epidemic 2.3.4.4b H5N1 emerged from 2.3.4.4b H5N8 viruses in Europe in 2020 with an N1 NA and five internal genes derived through reassortment with LPAIV circulating in Europe (Xie et al, 2023). Since its emergence, this H5N1 lineage has triggered an unprecedented multi-species epizootic. The virus exhibits remarkable ecological plasticity, achieving ecological dominance across both wild birds and commercial poultry worldwide. Phylogenetically validated evidence indicates that HPAIV clade 2.3.4.4b H5N1 has established sustained intraspecific transmission capacity in minks, marine mammals, and cattle (Aguero et al, 2023; Caserta et al, 2024; Leguia et al, 2023). Humans exposed to infected cattle generally develop mild illnesses, predominantly conjunctivitis, yet the observed limited airborne or fomite-based transmission in ferrets suggests a retained viral adaptive potential that warrants ongoing public health monitoring (Pulit-Penaloza et al, 2024).

Human seasonal IAVs preferentially bind to α2-6-linked sialyl glycans, while AIVs tend to bind to α2-3-linked sialyl glycans. The binding specificity of IAVs is crucial for their adaptation to human infection and plays a significant role in interspecies transmission of the virus (Liu et al, 2022; Shi et al, 2014). In this study, we aimed to evaluate the receptor-binding properties and molecular basis of two H5N8 isolates from 2020: huH5N8 from Russia and wsH5N8 from China. Both H5N8 HAs exhibited slight binding to human-like α2-6-linked sialic acids while maintaining a strong preference for avian-like α2-3-linked receptors. Furthermore, H5N8 effectively bound to human conjunctival, tracheal, and mammary tissues, as well as bovine mammary tissues, mirroring observations made with bovine H5N1 (Song et al, 2025). These findings suggest that, starting with the 2020 H5N8 isolates, clade 2.3.4.4b viruses already possessed the ability to engage both bovine mammary tissue and human conjunctiva.

Previous studies have identified key mutations that alter receptor-binding properties in H5N1 influenza viruses. One study demonstrated that the combination of mutations N158D/N224K/Q226L/T318I confers both a preference for human-type receptor binding and airborne transmissibility in ferret models for the A/Vietnam/1194/2004 H5N1 strain (Imai et al, 2012). Subsequent research revealed that two mutations, T160A and either Q226L or G228S, can independently establish human-type receptor specificity for the A/Indonesia/5/2005 H5N1 strain (Herfst et al, 2012; Linster et al, 2014). A recent study revealed that a Q226L substitution can switch the binding specificity of TxH5N1 HA from avian-type receptors to human-type receptors (Lin et al, 2024). These mutations acquire human-receptor binding through two mechanisms: N158D or T160A eliminates the glycosylation site at position 158, while Q226L creates a hydrophobic pocket that enhances interactions with α2-6-linked sialic acid receptors. Here, we demonstrate that even in the absence of the Q226L substitution, the recent 2.3.4.4b strains, including huH5N8, wsH5N8, and TxH5N1, have acquired dual receptor binding. Notably, structural alignment demonstrated comparable *cis* conformations between huH5N8/LSTc and the human-receptor-preferential TxH5N1mut/LSTc (Lin et al, 2024).

Prior studies have demonstrated that the early H5N1 strains, A/Vietnam/1203/2004 (clade 1) and A/Indonesia/5/2005 (clade 2.1.3.2), displayed negative staining to human tracheal tissue epithelial cells (Chutinimitkul et al, 2010; Imai et al, 2012). In contrast, Sun et al found that a clade 2.3.4.4 H5N8 strain, A/duck/northern China/08/2014, exhibited dual receptor-binding specificity as determined by ELISA assays, and could effectively bind to the human tracheal epithelium (Sun et al, 2022). These observations are consistent with our findings, further supporting that certain H5Ny viruses possess limited yet detectable affinity for human-like receptors. In addition, a recent study reported that the HAs of A/duck/France/161108 h/2016 (clade 2.3.4.4b H5N8), efficiently bound to both chicken and human tracheal tissues (Weber et al, 2026). Another article found that the A/Caspian gull/Netherlands/1/2022 H5N1 virus (clade 2.3.4.4b) attached more extensively to the human respiratory tract than A/Indonesia/5/2005 (clade 2.1.3.2) (Bauer et al, 2026). Song and his associates further demonstrated that TxH5N1 HA strongly binds to bovine and human conjunctival tissues, as well as mammary glands (Song et al, 2025). In this study, we systematically compared and observed that the HAs of A/Astrakhan/3212/2020 H5N8 exhibit strong binding to human tracheal and conjunctival tissues, bovine mammary gland, and duck tracheal epithelium, similar to the binding characteristics of TxH5N1 HAs.

Hiono et al studied recombinant HAs from two H5N2 influenza strains: A/chicken/Ibaraki/1/2005 (clade Am_nonGsGD; IBR) and A/duck/Mongolia/54/2001 (EA_nonGsGD; MNG) using glycan microarray analysis. The IBR HA (R222/R227) selectively binds to SLe^X^ glycans, whereas the MNG HA (K222/S227) preferentially binds to non-fucosylated α2-3 glycans. Remarkably, swapping these residues completely reversed the receptor-binding specificity of the HAs (Hiono et al, 2016). Similarly, Guo et al reported that Q222/R227 enhances SLe^X^ binding in the HA of A/chicken/Netherlands/14015526/2014 H5N8 (clade 2.3.4.4c), while retaining its affinity for α2-3 glycans, as shown by glycan array analysis (Guo et al, 2017). Good et al demonstrated that A/Texas/37/2024 H5N1 exhibits broad α2,3-sialoglycan binding, including SLe^X^ (Good et al, 2024). More recently, two studies further reported SLe^X^-binding capacity in the clade 2.3.4.4b strains A/duck/France/161108 h/2016 H5N8 and A/Bovine/OH/B24OSU-432/2024 H5N1 (Ghotekar et al, 2025; Weber et al, 2026). Our study focuses on the currently predominant clade 2.3.4.4b strains, particularly those that have infected humans, including A/Texas/37/2024 H5N1, A/Astrakhan/3212/2020 H5N8, and A/whooper swan/Henan/CAS001-K/2020 H5N8. By combining structural, biophysical, and mutational analyses, we further revealed how naturally occurring substitutions (Q222K/R227S) shape the receptor-binding pocket to accommodate SLe^X^ and human-type glycans. Importantly, to our knowledge, this work provides the first experimentally determined structure of a clade 2.3.4.4b H5 HA bound to SLe^X^, supported by quantitative SPR kinetics and mutational validation, thereby elucidating the molecular mechanism underlying the SLe^X^ preference and the potential for mammalian adaptation of these human-infecting strains.

Beyond studies on H5 subtype IAVs, research has also explored the role of SLe^X^ in H7 subtype infections. H7 IAVs can sporadically infect mammals, including humans, and frequently cause outbreaks in domestic poultry. Guan et al demonstrated that SLe^X^ expression varies across wild dabbling duck species. Importantly, their work revealed that H7 IAVs exhibit strong SLe^X^-binding affinity, enhancing viral attachment and infection. Binding assays showed that the H7 viruses specifically adhered to SLe^X^-expressing chicken colon crypt cells, while binding was significantly weaker in mallard cells lacking SLe^X^. These findings strongly suggest that SLe^X^ facilitates viral attachment and infection initiation (Guan et al, 2022). Spruit et al discovered that attachment of NeuGc-adapted H7 IAVs to tri-antennary N-glycans (particularly the α2,3-linked NeuAc and SLe^X^ structures) enables viral replication and shedding in chickens and ducks, potentially promoting cross-species transmission of equine-adapted H7 viruses (Spruit et al, 2024). Our study indicates that H5N8 HAs have evolved expanded recognition of α2-3-linked sialic acid receptors, and further reveals that SLe^X^ is widely distributed across diverse host tissues, including the human trachea, bovine mammary glands, and duck trachea. This broad tissue tropism could potentially contribute to sporadic human infections and may play a role in dissemination among wild birds, livestock, and other mammalian hosts. The enhanced binding affinity of 2.3.4.4b H5Ny HAs for SLe^X^ and α2-6 sialic acid receptors is suggestive of evolutionary adaptations that may be relevant to: (1) avian tracheal and intestinal dissemination, (2) mammalian respiratory transmission, and (3) mammary gland tropism, potentially enabling intraspecies transmission in bovines. Further functional studies, including assessments using loss-of-function viruses, are needed to directly evaluate the contribution of these mutations to 2.3.4.4b H5Ny virus transmission. In addition, close monitoring should consider the possibility that binding to non-sialic acid receptors, such as MHC class II (Giotis et al, 2019; Karakus et al, 2024; Karakus et al, 2019), may also influence influenza virus tropism as the virus continues to evolve.

Differences in research methods might explain the inconsistent findings regarding α2-6-linked glycan receptor binding among H5Ny viruses. Our glycan microarray analyses, conducted at relatively low HA concentrations, did not detect α2-6-linked glycan receptor binding for H5N8 HAs. In contrast, sensitive SPR assays at higher protein concentrations revealed weak but measurable binding to human receptor analogs. These discrepancies could explain the conflicting results in recent reports, some of which concluded that bovine H5 lacks α2-6-linked receptor binding based on glycan microarray analyses (Chopra et al, 2025; Good et al, 2024; Lin et al, 2024; Pulit-Penaloza et al, 2024; Santos et al, 2025). More importantly, recent virus-based solid-phase assays detected limited α2-6-linked receptor interactions (Eisfeld et al, 2024; Gu et al, 2024), which aligns with our findings. While the viral α2-6-linked receptor binding affinity remains relatively low, even a modest enhancement could amplify multivalent binding avidity, thereby elevating the risk of enhanced viral attachment and transmission outcomes. Recent investigations into bovine H5N1 challenge our data regarding the expanded breadth of receptor binding (Good et al, 2024). Specifically, they emphasize the T199I mutation near the RBS as a factor contributing to increased RBS flexibility. This mechanism contrasts with our conclusion that the K222Q and S227R mutations are the critical adaptive substitutions that enhance binding to both α2-6-linked and SLe^X^ glycans. Structural alignment also revealed equivalent RBS widths between huH5N8, which possesses T199, and TxH5N1, which possesses I199 (Figs. 4F and EV1). Nevertheless, these results collectively suggest that clade 2.3.4.4b H5N1 and H5N8 are undergoing convergent evolution, progressively enhancing their receptor repertoire and posing new risks for multi-species transmission.

The strategic implementation of comprehensive poultry vaccination is essential for combating the zoonotic avian influenza virus at its source (Shi et al, 2023). While many European and North American nations primarily rely on mass culling to contain HPAIV, some countries, including China, have adopted a vaccination-plus strategy. This approach effectively managed and controlled losses during the global H5 avian influenza outbreaks and successfully prevented human infections from the H7N9 virus through the vaccination of poultry (Shi et al, 2023). This success underscores the critical role of adaptive vaccine development synchronized with real-time virological monitoring for effective zoonosis containment. However, this vaccination program for poultry has attracted some debate, as recent research indicates that the inactivated vaccine could accelerate mutations of the H9N2 AIVs (Hu et al, 2025). This underscores the critical importance of closely monitoring vaccine efficacy and necessitates the development of more targeted vaccination strategies for effective control of avian influenza.

### Limitations of the study

Our study primarily investigates the receptor-binding properties of viral H5Ny HAs. Although integrating SPR, glycan microarray analysis, immunohistochemical staining, and structural biology approaches provides compelling evidence, it remains essential to confirm these findings at the viral level. Furthermore, cross-species adaptation involves more than just HA-mediated receptor binding; factors such as viral polymerase function, HA-NA balance, and host immune responses also require thorough exploration to fully understand the evolutionary trajectory and zoonotic potential of these H5Ny viruses.

## Methods

**Table:** Reagents and tools table

Reagent/Resource	Reference or Source	Identifier or Catalog Number
**Experimental Models**		
DH10Bac Chemically Competent Cell	Tsingke	Cat# TSC-C15
Sf9 cells	Invitrogen	Cat# 11496015
High Five cells	Invitrogen	Cat# B85502
**Recombinant DNA**		
pFastBac1-InH5 HA	(Zhang et al., 2013a)	N/A
pFastBac1-H1N1 HA	(Zhang et al, 2013b)	N/A
pFastBac1-H3N2 HA	(Hao et al, 2025)	N/A
**Antibodies**		
Anti-His-tag mAb	MBL	Cat# D291-3
Anti-Sialyl Lewis^X^ mAb	Biolegend	Cat# 368102
Goat anti-Mouse IgG (H+L) Superclonal^TM^ Secondary Antibody, Alexa Fluor^TM^ 488	Invitrogen	Cat# A28175
**Oligonucleotides and other sequence-based reagents**		
**Chemicals, Enzymes and other reagents**		
Insect-XPRESS	LONZA	Cat# 12-730Q
FuGENE® 6 Transfection Reagent	Promega	Cat# E2691
Thrombin	Sigma	Cat# T4648
Goat Serum	Beyotime	Cat# C0265
Maackia Amurensis Lectin I (MAL-I), Fluorescein	Vector Laboratories	Cat# FL-1311-2
Maackia Amurensis Lectin II (MAL-II), Biotinylated	Vector Laboratories	Cat# B-1265-1
**Continued**		
Streptavidin FITC	Invitrogen	Cat# 11-4317-87
Sambucus Nigra Lectin (SNA, EBL), CY5	Vector Laboratories	Cat# CL-1305-1
DAPI	Beyotime	Cat# C1005
IHC Biotin Block Kit	Sangon Biotech	Cat# E674001-0006
Improved Citrate Antigen Retrieval Solution	Beyotime	Cat# P0083
SlowFade^TM^ Diamond Antifade Mountant	Invitrogen	Cat# S36963
LS-tetrasaccharide a (LSTa; NeuAcα2-3Galβ1-3GlcNAcβ1-3Galβ1-4Glc)	Accurate Chemical &Scientific Corporation	Cat# 55/50
LS-tetrasaccharide c (LSTc; NeuAcα2-6Galβ1-4GlcNAcβ1-3Galβ1-4Glc)	Accurate Chemical &Scientific Corporation	Cat# 55/52
Sialyl Lewis X (SLe^X^; NeuAcα2-3Galβ1-4(Fucα1-3)GlcNAc)	MedChemExpress	Cat# HY-W020790
Biotinylated 3′SLNLN (NeuAcα2-3Galβ1-4GlcNAcβ1-3Galβ1-4GlcNAcβ1-SpNH-LC-LC-Biotin)	Consortium for Functional Glycomics	Cat# B178
Biotinylated 6′SLNLN (NeuAcα2-6Galβ1-4GlcNAcβ1-3Galβ1-4GlcNAcβ1-SpNH-LC-LC-Biotin)	Consortium for Functional Glycomics	Cat# B179
Biotinylated SLe^X^ (Neu5Acα2-3Galβ1-4(Fucα1-3)GlcNAcβ-C3-Biotin)	GlycoNZ	Cat#0062-BM
**Software**		
GraphPad Prism9	GraphPad Software	N/A
MEGA11	Tamura et al, 2021	https://www.megasoftware.net/
BIAcore 3000 Evaluation software	GE Healthcare	N/A
Leica TCS SP8 laser scanning confocal microscopy	Leica	https://www.leica-microsystems.com
HKL3000	Minor et al, 2006	N/A
CCP4 suit	Agirre et al, 2023	https://www.ccp4.ac.uk/
Coot	Emsley et al, 2010	https://www2.mrc-lmb.cam.ac.uk/perso nal/pemsley/coot/
Phenix	Adams et al., 2010	http://www.phenix-online.org/
Pymol software	N/A	https://pymol.org/2/
**Other**		
Human tissue sections	This paper	N/A
Cattle tissue sections	This paper	N/A
Duck tissue sections	This paper	N/A
HisTrap HP 5 mL column	GE Healthcare	Cat# 17524802
RESOURCE^TM^ Q, 6 ML	GE Healthcare	Cat# 17117901
Superdex^TM^ 200 Increase 10/300 GL column	GE Healthcare	Cat# 28990944
Sensor Chip SA	GE Healthcare	Cat# BR100032
Crystallization kits	Hampton Research and Molecular Dimensions	http://www.hamptonresearch.com; https://www.moleculardimensions.com/

### Gene cloning, expression, and protein purification

The genes encoding the ectodomains of the A/Astrakhan/3212/2020 HA (huH5N8 HA), A/whooper swan/Henan/CAS001-K/2020 HA (wsH5N8 HA), A/Texas/37/2024 HA (TxH5N1), A/Indonesia/5/2005 HA (InH5), A/duck/Czech/1956 HA (AvianH4 HA), A/South Carolina/1/1918 HA (H1N1 HA), and A/Kansas/14/2017 HA (H3N2 HA) were synthesized and cloned into the baculovirus transfer vector pFastBac1 in-frame with an amino-terminal gp67 signal peptide for secretion, a C-terminal thrombin cleavage site, a trimerization foldon sequence and a His_6_-tag at the extreme carboxy terminus for purification, as previously reported (Shi et al, 2013). Transfection and virus amplification were performed according to the Bac-to-Bac baculovirus expression system manual (Invitrogen). Hi5 cells in the log phase were infected and harvested 48 h post-infection. Soluble HAs were recovered from cell supernatants by metal affinity chromatography using a HisTrap HP column, then purified by ion-exchange chromatography using a RESOURCE^TM^ Q column. The purified proteins were subjected to thrombin digestion (overnight at 4 °C) to remove the C-terminal trimerization foldon sequence and His_6_-tag. The proteins were further purified by gel filtration chromatography using a Superdex^TM^200 Increase 10/300 GL column with a running buffer of 20 mM Tris-HCl 50 mM NaCl (pH 8.0) for crystallization and PBST (10 mM Na_2_HPO_4_, 1.76 mM KH_2_PO_4_, 137 mM NaCl, 2.7 mM KCl, pH 7.4, 0.005% Tween20) for SPR experiments.

### Crystallization, data collection, and structure determination

HuH5N8 HA and wsH5N8 HA were crystallized via the sitting-drop vapor diffusion method with 8 mg/mL at 18 °C. The huH5N8 HA crystals grew in a reservoir solution of 3% dextran sulfate sodium salt, 0.1 M bicine pH 7.7, 18% w/v PEG20000. The wsH5N8 HA crystals grew in a reservoir solution of 3% dextran sulfate sodium salt, 0.1 M bicine pH 8.5, 19% (w/v) PEG20000. For receptor analog complexes, crystals were soaked in a reservoir solution containing 1 mM LSTa or 10 mM LSTc for 4 h. All crystals were flash-cooled in liquid nitrogen after a brief soaking in reservoir solution with the addition of 17% (v/v) glycerol. The X-ray diffraction data were collected at the Shanghai Synchrotron Radiation Facility (SSRF) beamline 02U1. All data were processed with HKL3000 software.

The huH5N8 and wsH5N8 HA structures were determined by molecular replacement using Phaser from the CCP4 program suite, with the structure of the H5N1 HA from A/Indonesia/5/2005 (PDB code: 4K65) as a search model. The HA receptor analog complexes were subsequently solved using the refined HA structure as the input model. The receptor analogs were manually built using COOT based on the simulated annealing omit Fo-Fc maps and were further refined by PHENIX. Final statistics for data collection and structure refinement are represented in Appendix Tables S1 and  S2. The stereochemical quality of the final model was assessed with the program PROCHECK.

### Cryo-EM sample preparation, data collection, and structure determination

For receptor analog complexes, 0.3 mg/mL huH5N8 HA proteins were incubated with 10 mM SLe^X^ for 4 h. Then, 3 μL of the huH5N8 complexed with SLe^X^ was applied to graphene oxide (GO) grids (GO on Quantifoils R1.2/1.3 300 mesh copper grids, R1.2/1.3). The blotted grids (2 s, 100% humidity, and 4 °C) were rapidly frozen in liquid ethane (Vitrobot Mark IV, Thermo Fisher Scientific). The movie stacks were collected on a 300 kV Titan Krios transmission electron microscope equipped with a Gatan K3 detector and GIF Quantum energy filter in super-resolution counting mode (magnification of 105,000×, physical pixel size of 0.85 Å) using EPU. Each movie was dose-fractionated into 32 frames with a total dose of 50 e-/Å2 (defocus range of -1.0 to -2.0 µm). The datasets were processed using cryoSPARC v.4.1.0. The model was manually mutated and refined in Coot. Automated refinement was performed using Phenix with secondary structure and geometry restraints. Molprobity was used to validate the geometry and evaluate the structural quality.

### SPR experiments

The affinity and binding kinetics of HAs to receptor analogs were all analyzed at 25 °C on a BIAcore 3000 machine with streptavidin chips (SA chips). 6’SLNLN and 3’SLNLN were kindly provided by the Consortium for Functional Glycomics (Scripps Research Institute, Department of Molecular Biology, La Jolla, CA). Three biotinylated receptor analogs, 6’SLNLN, 3’SLNLN, and SLe^X^ were immobilized on the chip, and a blank channel was used as the negative control. Thrombin-digested HAs were serially diluted to different concentrations using PBST and then flowed through the chip. The response units were measured. The sensor surface was regenerated with 10 mM NaOH at the end of each cycle. Sensorgrams were globally fitted with BIAcore 3000 analysis software (BIAevaluation Version 4.1). The affinity values of huH5N8, wsH5N8, TxH5N1, and InH5 HAs were calculated with a steady-state affinity model due to the fast Ka (association rate constant) and Kd (dissociation rate constant). The affinity values of H1N1 and H3N2 HAs were calculated using a 1:1 Langmuir binding mode.

### Glycan microarray analyses

The neoglycolipid (NGL)-based microarray system was used to analyze the binding specificities of the recombinant HA proteins (C-terminal-His_6_-tagged huH5N8 HA, wsH5N8 HA, and AvianH4 HA). A broad-spectrum screening microarray of 668 sequence-defined lipid-linked glycan probes as previously described was used. The full list of glycan probes and their sequences are given in Dataset EV1. Details of the preparation of the glycan probes and the generation of the microarrays are in the Supplementary Glycan Microarray Document (Appendix Table S5) in accordance with the MIRAGE (Minimum Information Required for A Glycomics Experiment) guidelines for reporting of glycan microarray-based data. The microarray analyses were performed essentially as described. In brief, after blocking of the slides for 1 h with HBS buffer (10 mM HEPES, pH 7.4, 150 mM NaCl) containing 1% (w/v) BSA (bovine serum albumin), 0.02% (w/v) blocker Casein, and 10 mM CaCl_2_, the microarrays were overlaid with the His_6_-tagged HA pre-complexed with mouse monoclonal anti-poly-histidine and biotinylated anti-mouse IgG antibodies at a ratio of 2:2:1 (by weight) and diluted in the blocking solution to provide a final HA concentration of 50 µg/mL. Binding was detected with Alexa Fluor-647-labeled streptavidin (Molecular Probes) at 1 µg/mL incubated for 30 min. All steps were carried out at room temperature (RT) except for the precomplexing step, which was carried out on ice. Imaging and data analysis are described in the Supplementary MIRAGE document (Appendix Table S5).

### Immunofluorescence staining assays

The study protocol was approved by the Ethics Committee of Beijing Ditan Hospital, Capital Medical University (approval number: DTEC-KY2024-031-01). All procedures used in this research involving human participants were performed based on the ethical standards. The cow tissues were collected from a slaughterhouse in Beijing, China. Human tissue sections were kindly provided by Beijing Ditan Hospital Capital Medical University and Tongren Hospital Capital Medical University. Immunofluorescence assays were performed as described previously(Song et al, 2025). Briefly, human, bovine, and duck paraffin-embedded tissue sections were deparaffinized, rehydrated, and incubated with 10% goat serum (GSA) in PBS for 30 min at RT to block nonspecific binding.

For tissue tropism, purified HAs (still with trimerization foldon sequence and His_6_-tag) were preincubated with mouse anti-His antibody at a molar ratio of 2:1 and incubated on ice for 20 min. The pre-complexed HAs were applied to human, bovine, and duck tissue sections at the HA concentration of 50 µg/mL and incubated overnight at 4 °C. The sections were then washed three times for 5 min with PBS and incubated with 20 µg/mL Alexa Fluor 488 goat anti-mouse IgG for 1 h at RT. Finally, the sections were counterstained with DAPI for 30 min at RT to detect nuclei. After thorough washing with PBST, the sections were mounted and observed using confocal laser scanning microscopy (Leica TCS SP8 laser scanning confocal microscope).

For both SLe^X^ and lectin staining, sections underwent antigen retrieval by heating in 0.01 M sodium citrate buffer (pH 6.0) at 95–100 °C for 20 min, followed by cooling to RT. (1) For SLe^X^ staining, the sections were washed three times with PBS and incubated with 20 µg/mL anti-Sialyl Lewis^X^ antibody overnight at 4 °C. After three 5-minute PBS washes, sections were incubated with 20 µg/mL Alexa Fluor 677 goat anti-mouse IgG for 1 h at RT. (2) For Fluorescein-conjugated MAL-I or CY5-conjugated SNA (Vector Laboratories), sections were washed three times with PBS and incubated with 20 µg/mL lectins overnight at 4 °C, as previously described (Song et al, 2025). For MAL-II staining, biotinylated-conjugate MAL-II was used. After antigen repair and PBS wash, slides were blocked with the IHC Biotin Block Kit (Sangon Biotech) per the manufacturer’s protocol, followed by overnight incubation with 20 µg/mL MAL-II at 4 °C. After lectin incubation, sections were treated with FITC-conjugated streptavidin (Invitrogen) for 1 h at 4 °C. Finally, sections were counterstained with DAPI for 30 min at RT to visualize nuclei, washed thoroughly with PBST, mounted, and imaged using a Leica TCS SP8 confocal microscope.

### Graphics

Some of the graphical elements used in the synopsis figure were provided by BioRender.com.

## Supplementary information

Appendix

Peer Review File

Dataset EV1

Dataset EV2

Source data Fig. 1

Source data Fig. 2

Source data Fig. 3

Source data Fig. 4

Source data Fig. 5

Source data Fig. 6

Expanded View Figures

## Data Availability

The structures of huH5N8 HA, swH5N8 HA, huH5N8 HA/LSTa, huH5N8 HA/LSTc, huH5N8 HA/SLe^X^, swH5N8 HA/LSTa, and swH5N8 HA/LSTc have been deposited in the Protein Data Bank (PDB; www.rcsb.org) under accession numbers 8X28, 8X2C, 8X26, 8X27, 8X2F, 8X29, and 8X2D, respectively. The source data of this paper are collected in the following database record: biostudies:S-SCDT-10_1038-S44319-026-00816-2.
