# Supplementary material for: Hemagglutinin double-mutation enhances binding of human-infecting avian influenza virus clade 2.3.4.4b H5Ny to human and SLeX receptors
Source: EMBO Rep. 2026 Jun 16;27(14):4079–99. doi: 10.1038/s44319-026-00816-2 (PMC13400655; doi:10.1038/s44319-026-00816-2)
Supplement: Supplementary file 1 — Appendix [file 44319_2026_816_MOESM1_ESM.pdf]

Appendix for: Double-mutation enhances binding of human-infecting clade 2.3.4.4b H5 to human and SLe^X receptors

Appendix Table of Contents:

Page 3: Appendix Figure S1

Page 4: Appendix Table S1

Page 5: Appendix Table S2

Page 6: Appendix Table S3

Page 7: Appendix Table S4

Page 8-11: Appendix Table S5

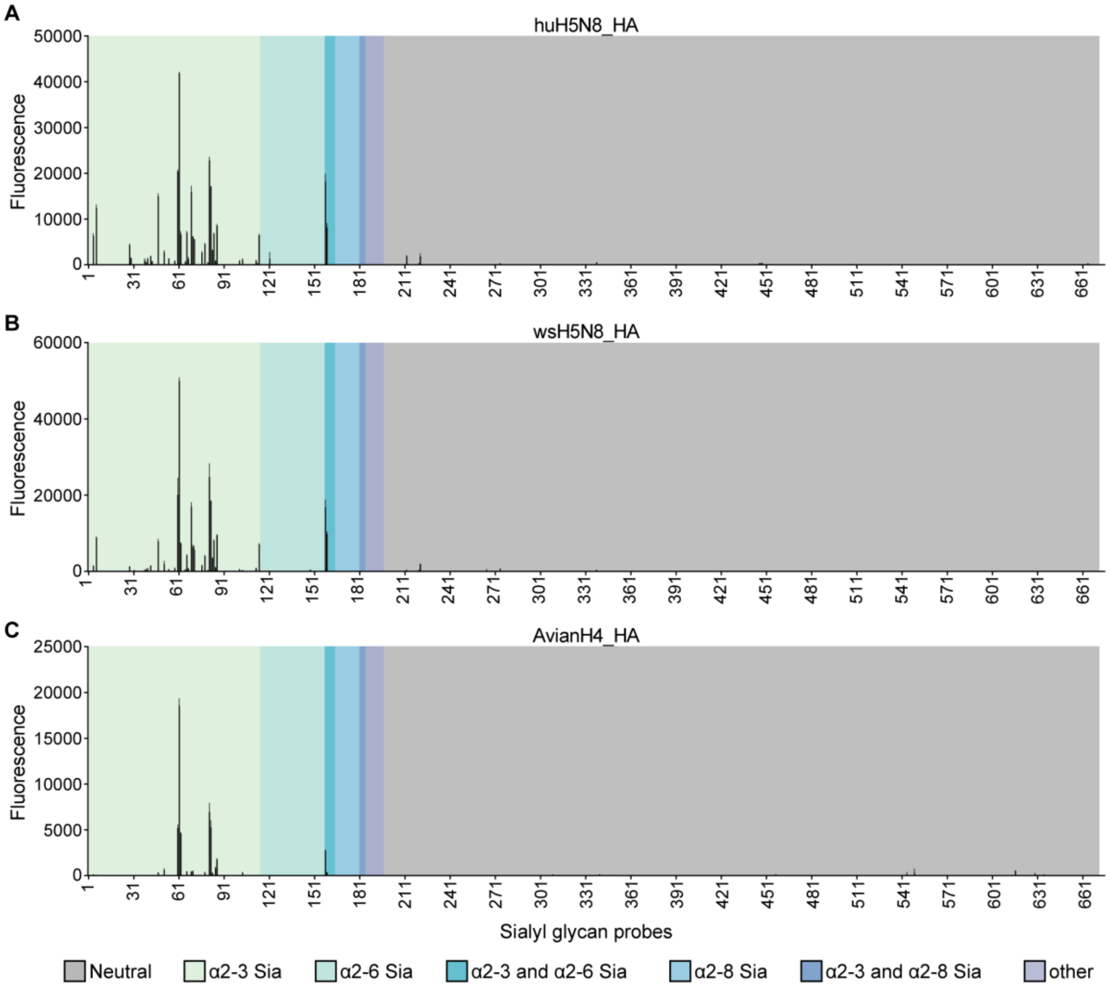

Appendix Figure S1. Glycan microarray screening analyses of HAs.

The results are the means of fluorescence intensities of duplicate spots, printed at 5 fmol per spot. The error bars represent half of the difference between the two values. In the glycan array the 668 lipid-linked probes are grouped according to sialyl linkages as annotated by the colored panels. The list of glycan probes with their sequences and binding scores are in Dataset EV2. Representative results from at least two independent experiments are shown.

Appendix Table S1. Crystallographic data collection and refinement statistics for huH5N8 HA.

	huH5N8 HA	huH5N8 HA / LSTa	huH5N8 HA / LSTc
**Data collection**			
Space group	P 1 21 1	P 1 21 1	P 1 21 1
Cell dimensions			
a, *b*, *c* (Å)	72.93, 267.38, 131.19	72.98, 263.81, 131.62	72.83, 263.93, 131.20
α, β, γ (°)	90.00, 99.46, 90.00	90.00, 101.64, 90.00	90.00, 101.08, 90.00
Resolution (Å)	267.38 - 3.19 (3.27 - 3.19)	50.00-3.12 (3.23-3.12)	50.00-3.05 (3.16-3.05)
R_{merge}	0.349 (1.999)	0.171 (1.302)	0.154 (1.274)
$I / \sigma I$	5.2 (1.2)	7.8 (1.0)	9.1 (1.1)
$CC_{1/2}$	0.964	0.985	0.987
Completeness (%)	100.0 (100.0)	99.9 (100.0)	96.7 (94.1)
Redundancy	6.8 (6.9)	4.8 (4.9)	5.3 (5.2)
**Refinement**			
Resolution (Å)	133.69-3.19 (3.30-3.19)	35.74 - 3.12 (3.23 - 3.12)	31.72 - 3.04 (3.15 - 3.04)
No. reflections	82113	84599 (6807)	89362 (8318)
R_{work} / R_{free}	0.2476/0.26248	0.2656/0.2710	0.2213/0.2473
No. atoms			
Protein	22592	23745	23627
Ligand/ion	14	35	32
Water	0	0	0
B-factors			
Protein	79.2	107.3	98.0
Ligand/ion	107.4	100.9	106.8
Water	-	-	-
**R.m.s. deviations**			
Bond lengths (Å)	0.006	0.006	0.005
Bond angles (°)	0.705	1.030	0.795
**Ramachandran plot**			
Favored (%)	94.97	95.79	95.58
Allowed (%)	4.96	4.14	4.38
Outliers (%)	0.07	0.07	0.04

*Values in parentheses are for highest-resolution shell.

Appendix Table S2. Crystallographic data collection and refinement statistics for wsH5N8 HA.

	wsH5N8 HA	wsH5N8 HA / LSTa	wsH5N8 HA / LSTc
**Data collection**			
Space group	P 1 21 1	P 1 21 1	P 1 21 1
Cell dimensions			
a, *b*, *c* (Å)	81.41, 107.03, 134.76	80.15, 108.50, 133.19	80.68, 107.03, 134.33
α, β, γ (°)	90.00, 101.05, 90.00	90.00, 99.79, 90.00	90.00, 100.47, 90.00
Resolution (Å)	50-2.60 (2.69-2.60)	50-2.60 (2.69-2.60)	50-2.50 (2.59-2.50)
R_{merge}	0.154 (1.509)	0.188 (1.100)	0.123 (0.838)
$I / \sigma I$	11.1 (1.1)	9.1 (1.3)	14.8 (1.9)
$CC_{1/2}$	0.996	0.975	0.985
Completeness (%)	99.8 (99.9)	98.1 (99.1)	100.0 (100.0)
Redundancy	6.7 (6.6)	4.3 (4.4)	5.6 (5.7)
**Refinement**			
Resolution (Å)	28.27 - 2.59 (2.68 - 2.59)	25.01 - 2.61 (2.71 - 2.61)	49.63 - 2.48 (2.57 - 2.48)
No. reflections	69913 (6357)	66499 (6288)	78660 (7300)
R_{work} / R_{free}	0.2306/0.2701	0.1904/0.2222	0.2342/0.2524
No. atoms			
Protein	12022	12367	12327
Ligand/ion	10	14	20
Water	116	401	275
B-factors			
Protein	68.0	45.0	73.8
Ligand/ion	80.9	68.0	74.0
Water	51.0	37.5	54.2
**R.m.s. deviations**			
Bond lengths (Å)	0.006	0.010	0.011
Bond angles (°)	0.660	1.97	1.126
**Ramachandran plot**			
Favored (%)	96.16	97.11	97.05
Allowed (%)	3.84	2.82	2.95
Outliers (%)	0.00	0.07	0.00

*Values in parentheses are for highest-resolution shell.

Appendix Table S3. Cryo-EM data collection, refinement and validation statistics for huH5N8 HA and SLe^{X} complex.

	huH5N8 HA / SLe^{X} (PDB D_1300033112)
**Data collection and processing**	
Magnification	105k
Voltage (kV)	300
Electron exposure (e–/Å^{2})	60
Defocus range (μm)	-1.0 ~ -2.0
Pixel size (Å)	0.69
Symmetry imposed	C1
Initial particle images (no.)	803,862
Final particle images (no.)	362,087
Map resolution (Å)	2.62
FSC threshold	0.143
**Refinement**	
Initial model used (PDB code)	4UO6
Model resolution range (Å)	Up to 2.9
Map sharpening *B* factor (Å^{2})	116
Model composition	
Non-hydrogen atoms	12098
Protein residues	1472
Ligands	24
R.m.s. deviations	
Bond lengths (Å)	0.005
Bond angles (°)	0.813
Validation	
MoProbity score	1.69
Clashscore	6.11
Poor rotamers (%)	1.16
Ramachandran plot	
Favored (%)	95.48
Allowed (%)	4.52
Disallowed (%)	0.00

Appendix Table S4. Interaction between receptor analogs and H5N8 HAs.

Receptor analog		huH5N8 HA	wsH5N8 HA
LSTa	SIA	Y98 (8,**2**), L133 (4), G134 (3), V135 (11,**1**), S136 (12,**1**), A137 (5,**1**), W153 (18), I155 (2), H183 (7,**1**), E190 (9,**1**), L194 (9), Q226 (18,**1**), S228 (1)	Y98 (11,**2**), L133 (4), G134 (3), V135 (11,**1**), S136 (13,**1**), A137 (5,**2**), W153 (19), I155 (2), H183 (4), N182 (4,**1**), E190 (8,**1**), L194 (8), Q226 (15), S228 (2)
	GAL	Q226 (9,**2**)	N186 (1), E190 (3,**1**), Q226 (6)
	NAG		
Total		116,**10**	119,**9**
LSTc	SIA	Y98 (7,**2**), L133 (6), G134 (3), V135 (13,**1**), S136 (13,**1**), A137 (5,**1**), W153 (22), I155 (3), H183 (5,**1**), N186 (2,**1**), E190 (12,**1**), L194 (10), Q226 (12,**1**) S228 (1)	Y98 (6,**2**), L133 (5), G134 (3), V135 (10,**1**), S136 (12,**1**), A137 (5,**1**), W153 (21), I155 (2), H183 (6,**1**), N186 (1), E190 (8,**1**), L194 (5), Q226 (12,**1**),
	GAL	222Q (1), 225G (2), 226Q (7)	Q222 (3,**1**), G225 (5,**1**), Q226 (11)
	NAG		N193 (1), L194 (1)
	GAL	N193 (6)	E189 (10,**1**), N193 (13)
Total		130,**9**	140,**11**
SLe^X	SIA	Y98 (7,**2**), L133 (5), G134 (1), V135 (7,**1**), S136 (4,**1**), A137 (6,**1**), W153 (12), I155(1), H183 (4,**1**), N186 (1), E190 (6,**1**), L194 (4), Q226 (10,**1**), S228 (1)	
	GAL	N186 (1), E190 (2,**1**), Q226 (6),	
	NAG		
	FUC	Q222 (11,**2**), G225 (7,**1**), Q226 (1), R227 (2)	
Total		99,**12**	

Residues without underscores in parentheses indicate the number of vdw contacts between the receptor analogs and the huH5N8 HA or wsH5N8 HA RBS regions. Underlined numbers in bold format indicate the number of H-bonds between the residues and the sugar ring. Vdw contact was analyzed at a cutoff of 4.5 Å and H-bonds at a cutoff of 3.5 Å.

Appendix Table S5. Supplementary glycan microarray document based on MIRAGE Glycan Microarray guidelines (doi:10.3762/mirage.3) for the glycan array experiments.

Classification	Guidelines
**1. Sample: Glycan Binding Sample**	
Description of Sample	Sample name:<br>A/Astrakhan/3212/2020_H5N8 (huH5N8)<br>A/whooper_swan/Henan/CAS001-K/2020_H5N8 (wsH5N8)<br>A/duck/Czech/1956_H4N6 (AvianH4)<br>Origin: recombinant<br>Method of preparation:<br>Please see the *Materials and Methods* section in the main text.
Sample modifications	Not relevant.
Assay protocol	Microarray analyses were performed essentially as described (Liu et al., Methods Mol. Biol. 2012), for modifications of the protocols please see “Glycan array experiments” under *Experimental* section in the main text.
**2. Glycan Library**	
Glycan description for defined glycans	A broad-spectrum screening microarray containing 668 sequence-defined lipid-linked glycan probes was used. The probe names and corresponding structures are in **Dataset EV1**. These are a sub-set of a recently generated large screening microarray containing around 900 glycan probes (in-house designation ‘Array Sets 42-56’, which will be published elsewhere). The NGL probes are from the collection assembled in the course of research in the Glycosciences Laboratory (https://glycosciences.med.ic.ac.uk/glycanLibraryList.html).
Glycan description for undefined glycans	Not relevant.
Glycan modifications	For NGLs, unless otherwise specified these were prepared from reducing oligosaccharides by reductive amination with the amino lipid, 1,2-dihexadecyl-*sn*-glycero-3-phosphoethanolamine [(DHPE) (Chai et al., Methods Enzymol. 2003)]; AO, NGLs prepared from reducing oligosaccharides by oxime ligation with an aminooxy

	functionalized DHPE [(AOPE) (Liu et al., Chem. Biol. 2007)]. For full description on the definition of lipid moieties of the glycan probes please see https://glycosciences.med.ic.ac.uk/docs/lipids.pdf
**3. Printing Surface; e.g., Microarray Slide**	
Description of surface	Nitrocellulose-coated glass microarray slides.
Manufacturer	16-pad UniSart® 3D Microarray Slide from Sartorius (Goettingen, Germany)
Custom preparation of surface	Not relevant.
Non-covalent Immobilisation	The lipid-linked oligosaccharide probes were formulated as liposomes by adding carrier lipids, 1,2-dihexanoyl-*sn*-glycero-3-phosphocholine (DHPC) and cholesterol for arraying and non-covalent immobilization on nitrocellulose-coated glass slides (Liu et al., Methods Mol. Biol. 2012).
**4. Arrayer (Printer)**	
Description of Arrayer	Nano-Plotter 2.1 (GeSiM, Radeberg, Germany).
Dispensing mechanism	Non-contact liquid delivery with four dispensing tips.
Glycan deposition	Approximately 0.33 nl was printed per spot. Lipid-linked glycan probes were printed at 2 and 5 fmol per spot.
Printing conditions	The printing solutions were all aqueous based. Printing was performed at ambient temperature and relative humidity of 58%. The 'liposome' printing solutions contained 100 pmol/µl of DHPC and cholesterol (both from SIGMA) as lipid carriers in addition to the lipid-linked glycan probes. The concentrations of the lipid-linked glycan probes were 5 and 15 pmol/µl for the 2 and 5 fmol per spot levels, respectively. The printing solutions also contained Cy3 NHS ester (GE Healthcare) at 20 ng/ml (26 fmol/µl) as a marker to monitor the printing process.
**5. Glycan Microarray with "Map"**	
Array layout	Each array slide contained 16-pad subarrays. Each pad was set up for printing 64 probes maximum, each at 2 levels in duplicate (four spots for one probe in a row); up to 256 spots (16x16) in total in each pad.

	The 668 lipid-linked probes in the screening arrays were printed on multiple subarrays for parallel binding analyses.
Glycan identification and quality control	The quality control of the glycan microarrays was routinely carried out with (i) a panel of biotinylated plant lectins (Vector Laboratories), e.g. *Ricinus Communis* Agglutinin I (RCA_{120}), *Aleuria aurantia* lectin (AAL), Concanavalin A (ConA) and wheat germ agglutinin (WGA), (ii) anti-carbohydrate antibodies, and (iii) commercial bacterial adhesins and toxins. Predicted binding results were recorded. These data will be published and shared via the GlyGen Glycan Array Repository currently under development as part of the NIH-funded GlyGen initiative (https://www.glygen.org/), which has entered its final testing phase. In the meantime, selected datasets can be seen in the shared Google folder via the link https://drive.google.com/drive/folders/1hMrbWX4k3XxBd8FIB8cHbPkHB-sxNzkj?usp=sharing.<br><br>The sialylated glycan probes included in the present glycan array analyses have been well validated in previous studies with influenza viruses (Childs, et al Nat Biotechnol. 2009), and a number of viral adhesive proteins, including VP1 proteins of polyomaviruses, simian virus 40 (Campanero-Rhodes, et al, Nat Cell Biol. 2007), human JC polyomavirus (Neu, et al, Cell Host Microbe. 2010), the fiber knob of human adenovirus 52 (Lenman, et al. Proc Natl Acad Sci U S A. 2018), and adhesins of *Streptococcus sanguinis* (Shahin et al, Proc Natl Acad Sci U S A 2023).
**6. Detector and Data Processing**	
Scanning hardware	GenePix 4300A (Molecular Devices, UK)
Scanner settings	Scanning resolution: 10 µm / pixel<br><br>Laser channel: Red (scan wavelength 635 nm)<br><br>PMT: 350<br><br>Scan power: 50% to achieve maximum signal without spot saturation.
Image analysis software	GenePix® Pro 7 (Molecular Devices)
Data processing	The gpr files were entered into an in-house microarray database using software (http://www.beilstein-institut.de/en/publications/proceedings/glyco-2009) for data processing. No particular normalization method or statistical analysis was used for the results of the screening arrays.
**7. Glycan Microarray Data Presentation**	
Data presentation	The microarray binding results focused on sialyl glycan probes are in Fig. 2 and Dataset EV2. The full results are in Appendix Fig. S1 and Dataset EV1.

8. Interpretation and Conclusion from Microarray Data	
Data interpretation	No software or algorithms were used to interpret processed data.
Conclusions	Under the assay conditions used, the three recombinant HAs showed binding to α2-3-linked sialyl glycans in the arrays. The two H5N8 HAs bound to a broader range of probes than the avianH4 HA, including fucosylated sialyl glycans such as Sialyl Lewis a and Lewis x related sequences, which were not recognized by the avain H4 HA.
