# Supplementary figures and images for: Hemagglutinin double-mutation enhances binding of human-infecting avian influenza virus clade 2.3.4.4b H5Ny to human and SLeX receptors
Source: EMBO Rep. 2026 Jun 16;27(14):4079–99. doi: 10.1038/s44319-026-00816-2 (PMC13400655; doi:10.1038/s44319-026-00816-2)

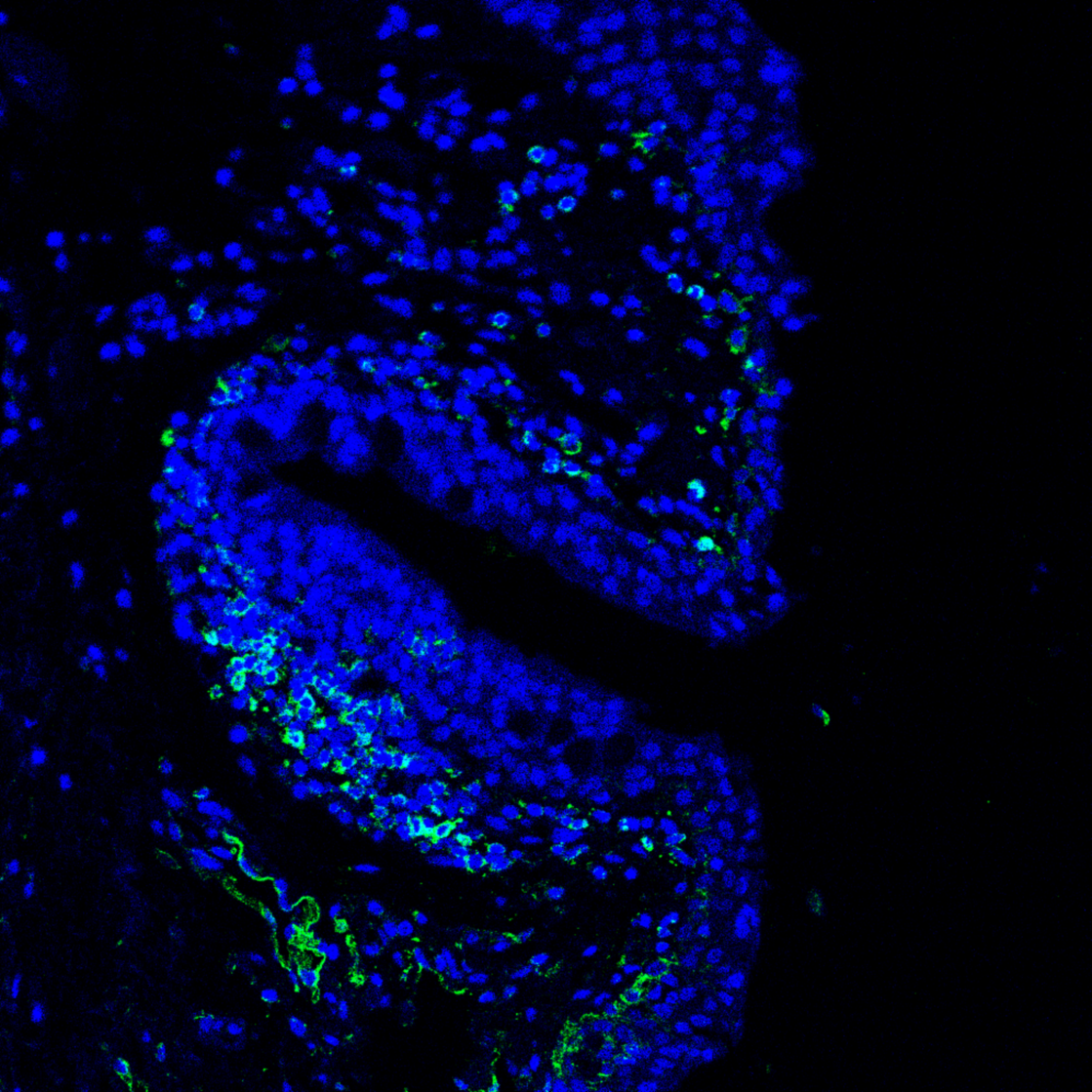

Supplement: Supplementary file 7 — Source data Fig. 3 [file 44319_2026_816_MOESM7_ESM.zip › Figure 3/3A/A-H1N1.tif]

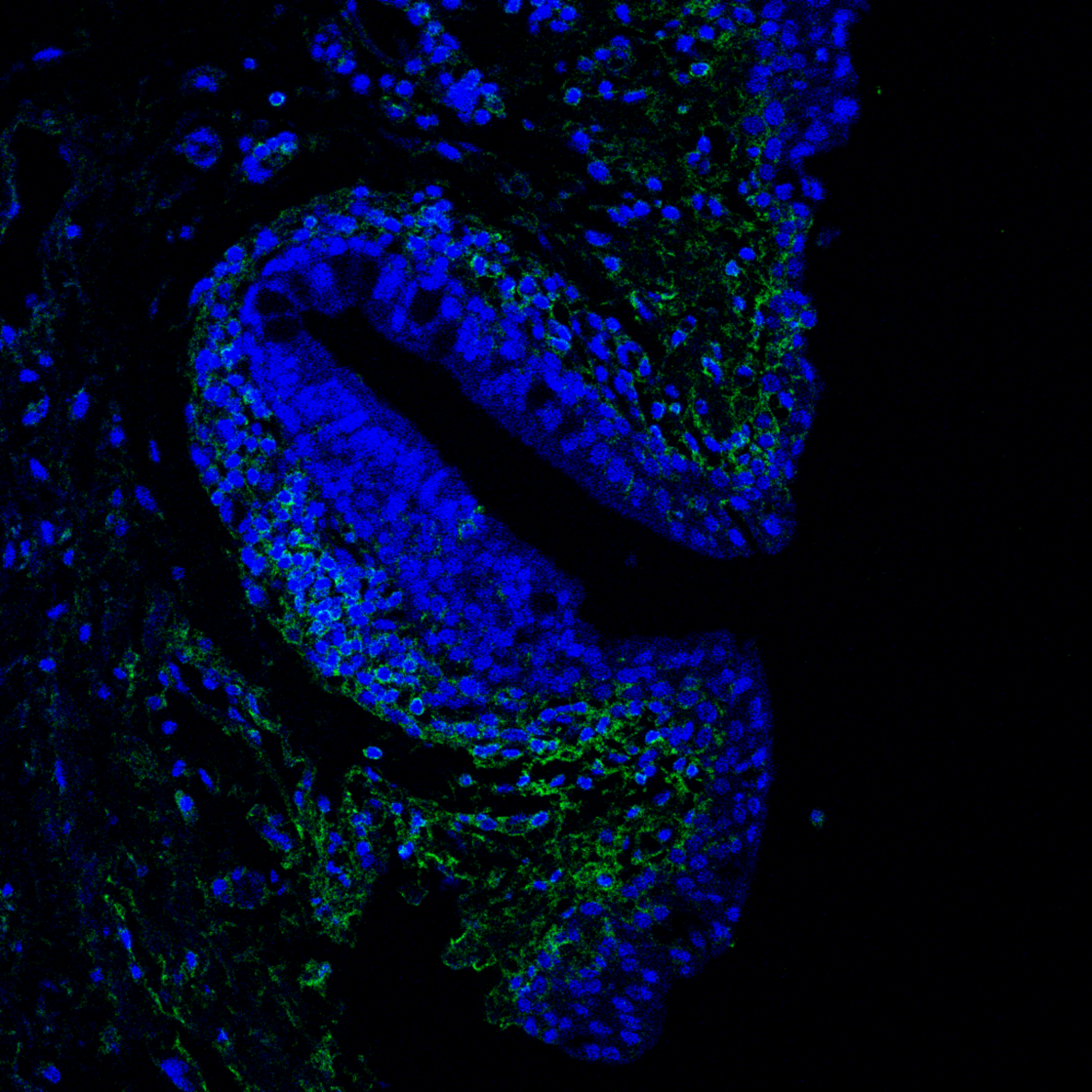

Supplement: Supplementary file 7 — Source data Fig. 3 [file 44319_2026_816_MOESM7_ESM.zip › Figure 3/3A/A-H3N2.tif]

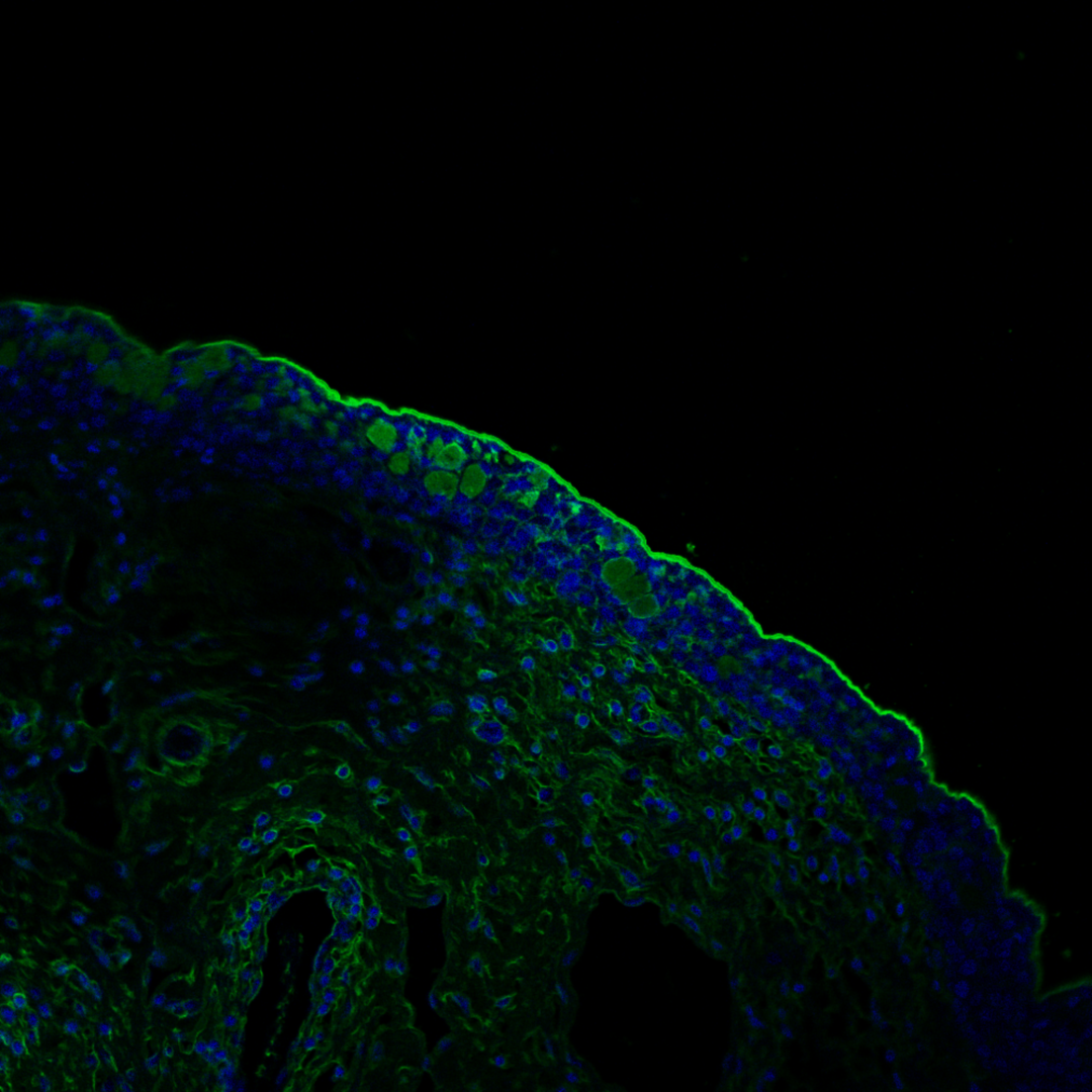

Supplement: Supplementary file 7 — Source data Fig. 3 [file 44319_2026_816_MOESM7_ESM.zip › Figure 3/3A/A-H5N8.tif]

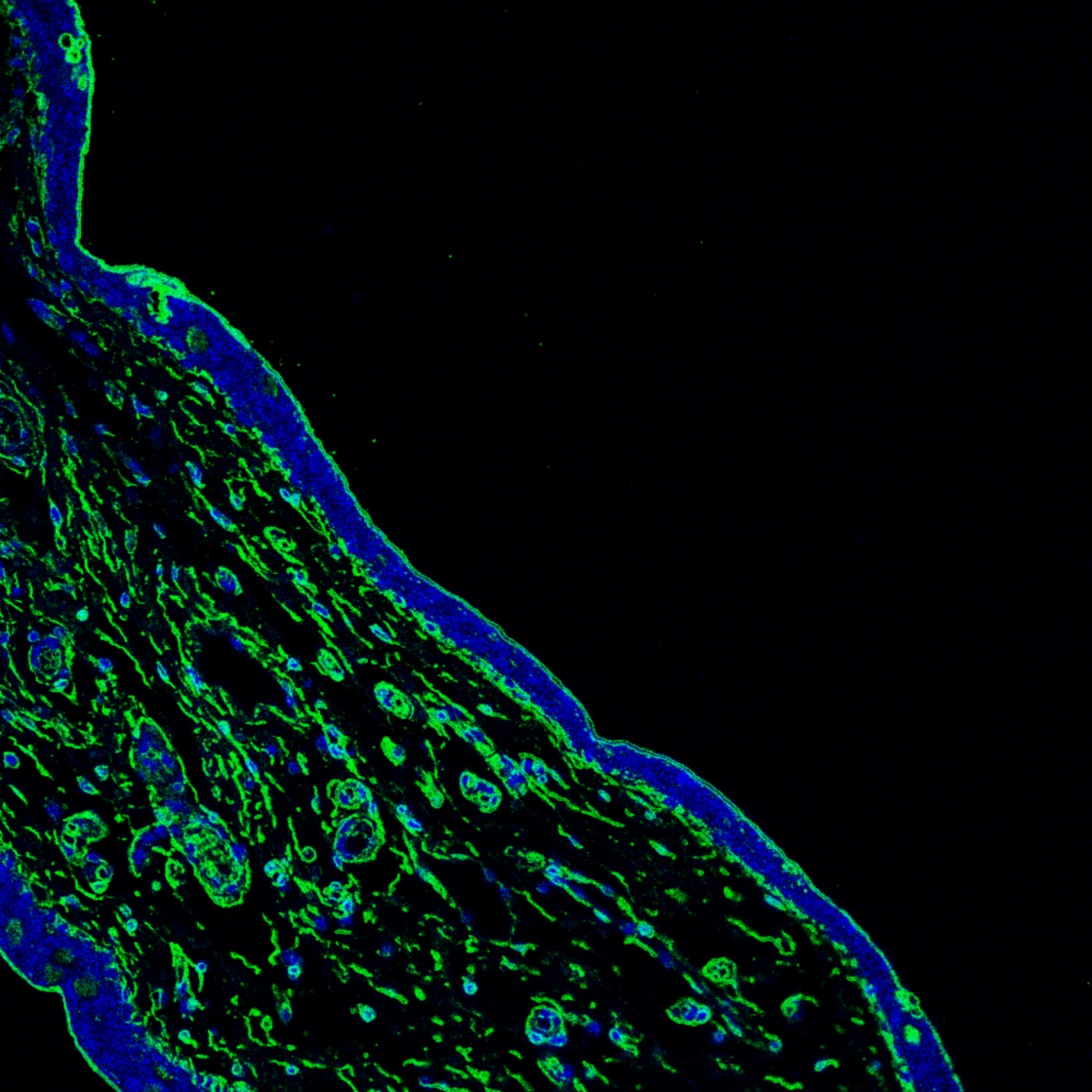

Supplement: Supplementary file 7 — Source data Fig. 3 [file 44319_2026_816_MOESM7_ESM.zip › Figure 3/3A/A-lnH5.tif]

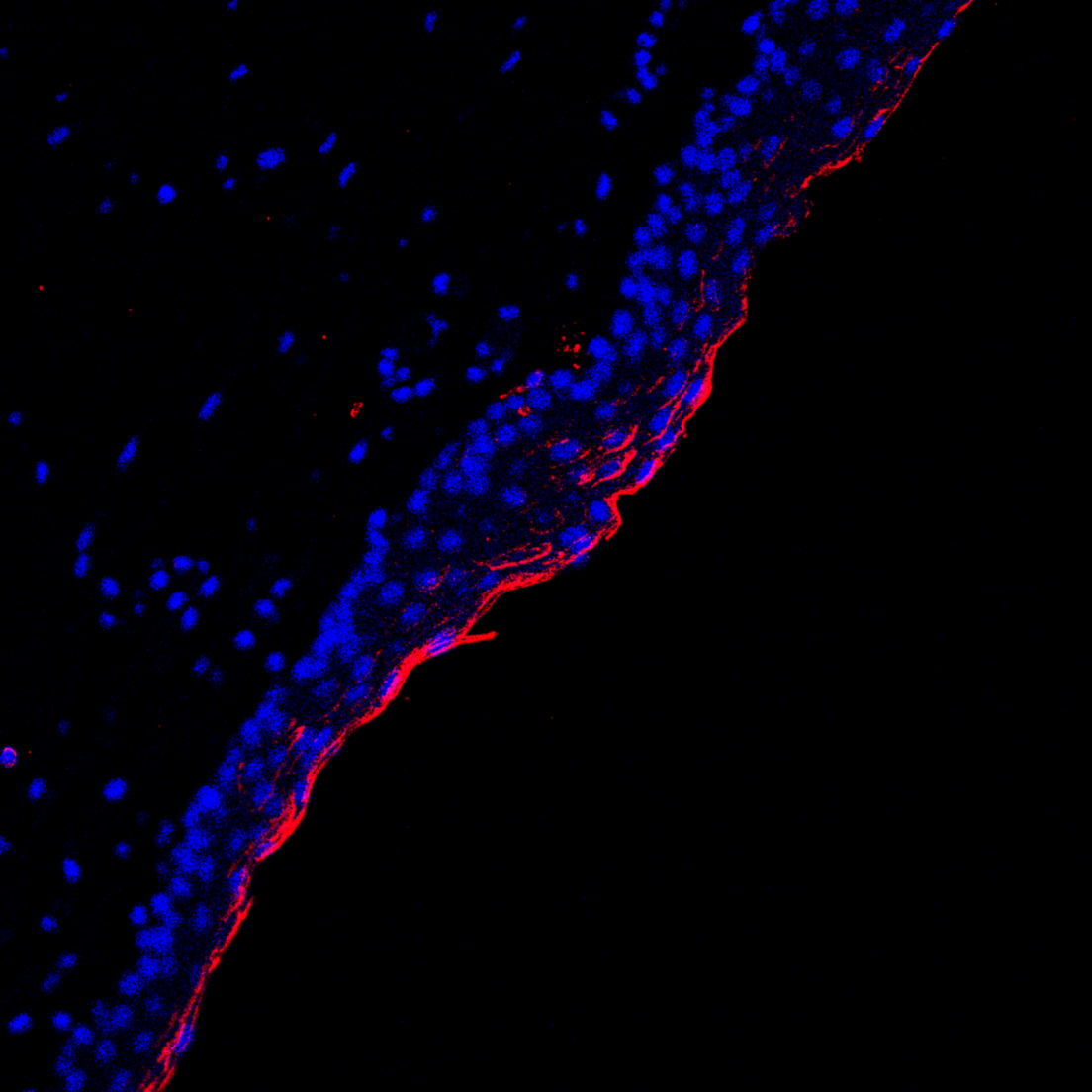

Supplement: Supplementary file 7 — Source data Fig. 3 [file 44319_2026_816_MOESM7_ESM.zip › Figure 3/3A/A-SLEX.tif]

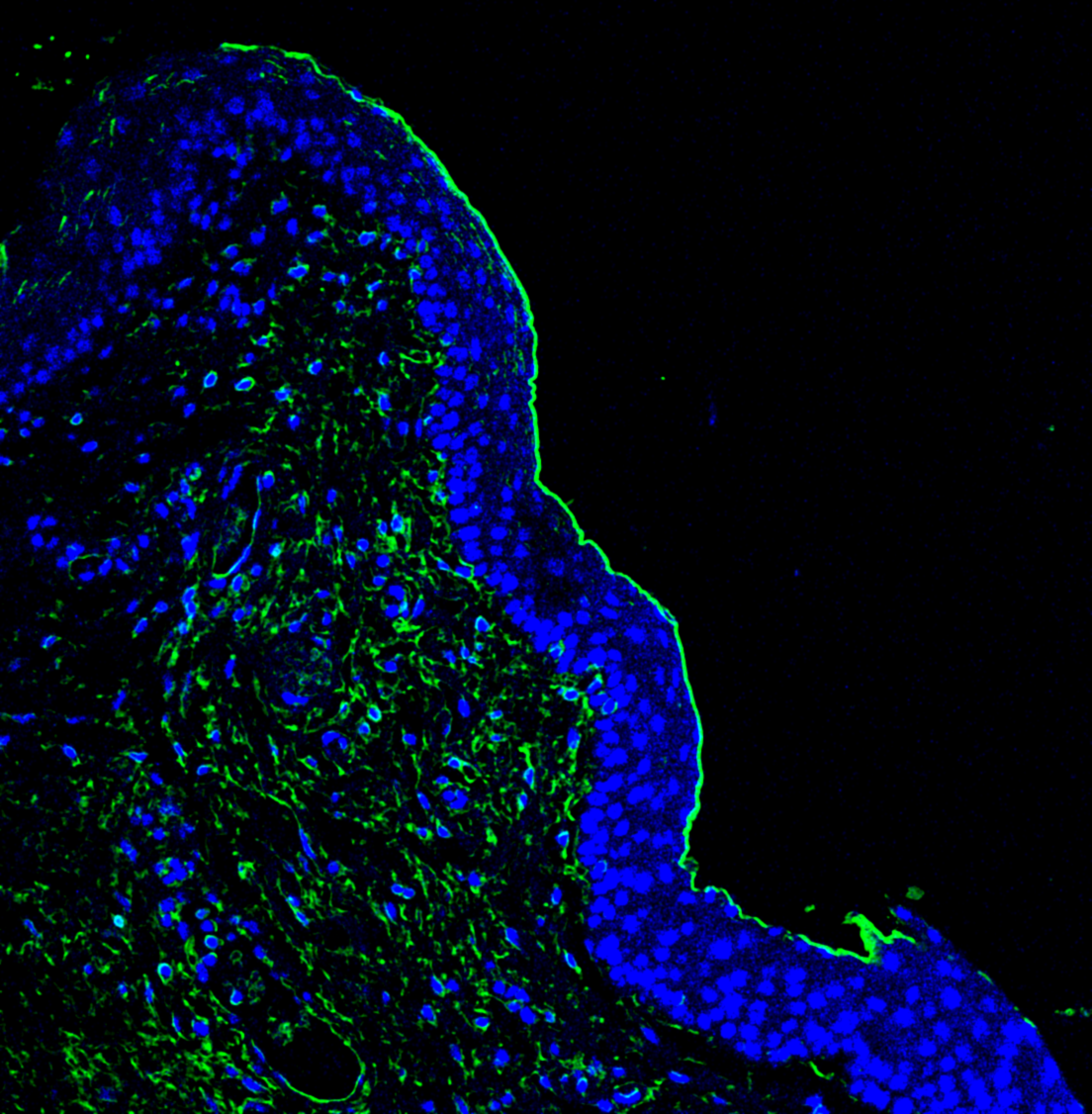

Supplement: Supplementary file 7 — Source data Fig. 3 [file 44319_2026_816_MOESM7_ESM.zip › Figure 3/3A/A-TxH5N1.tif]

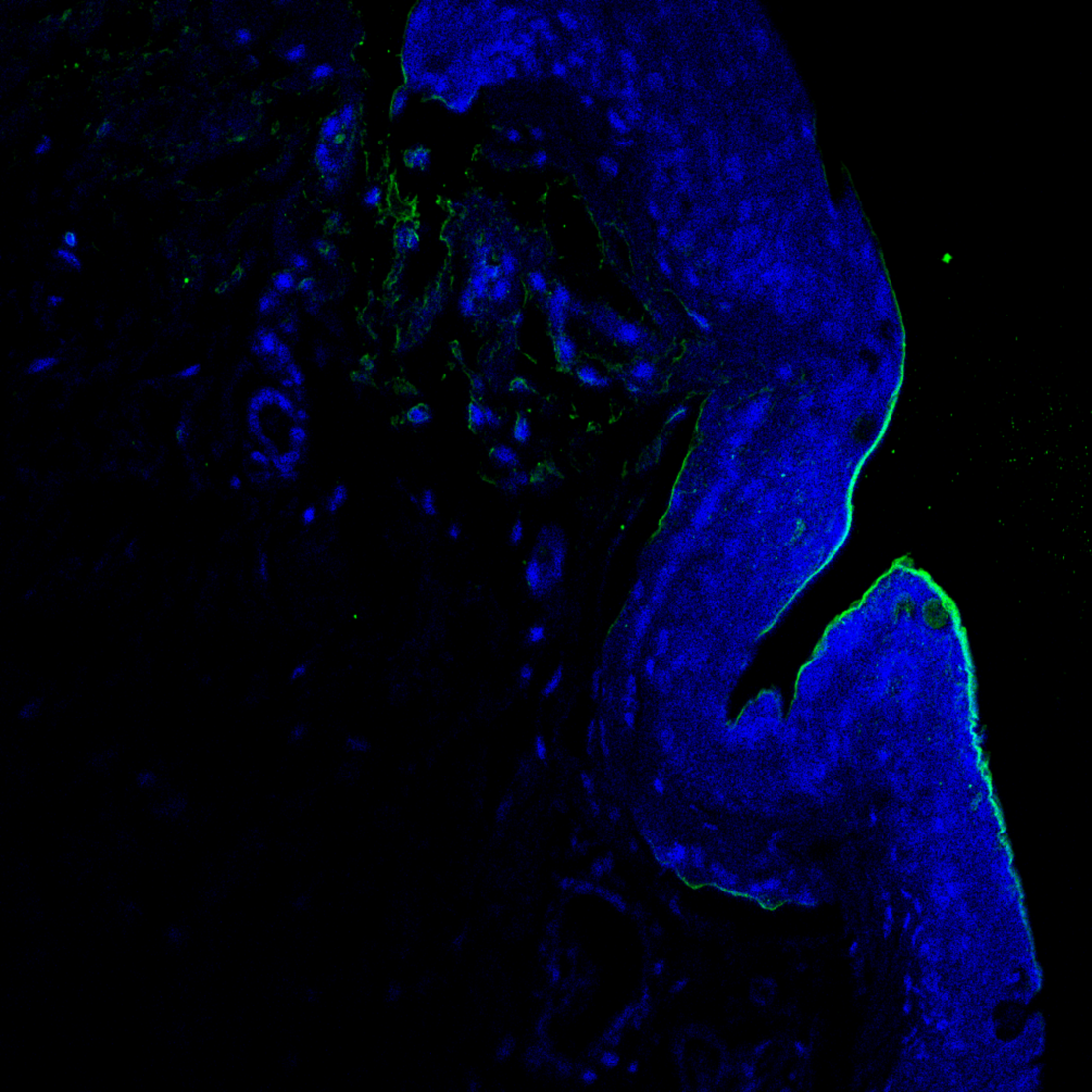

Supplement: Supplementary file 7 — Source data Fig. 3 [file 44319_2026_816_MOESM7_ESM.zip › Figure 3/3A/A-wsH5N8.tif]

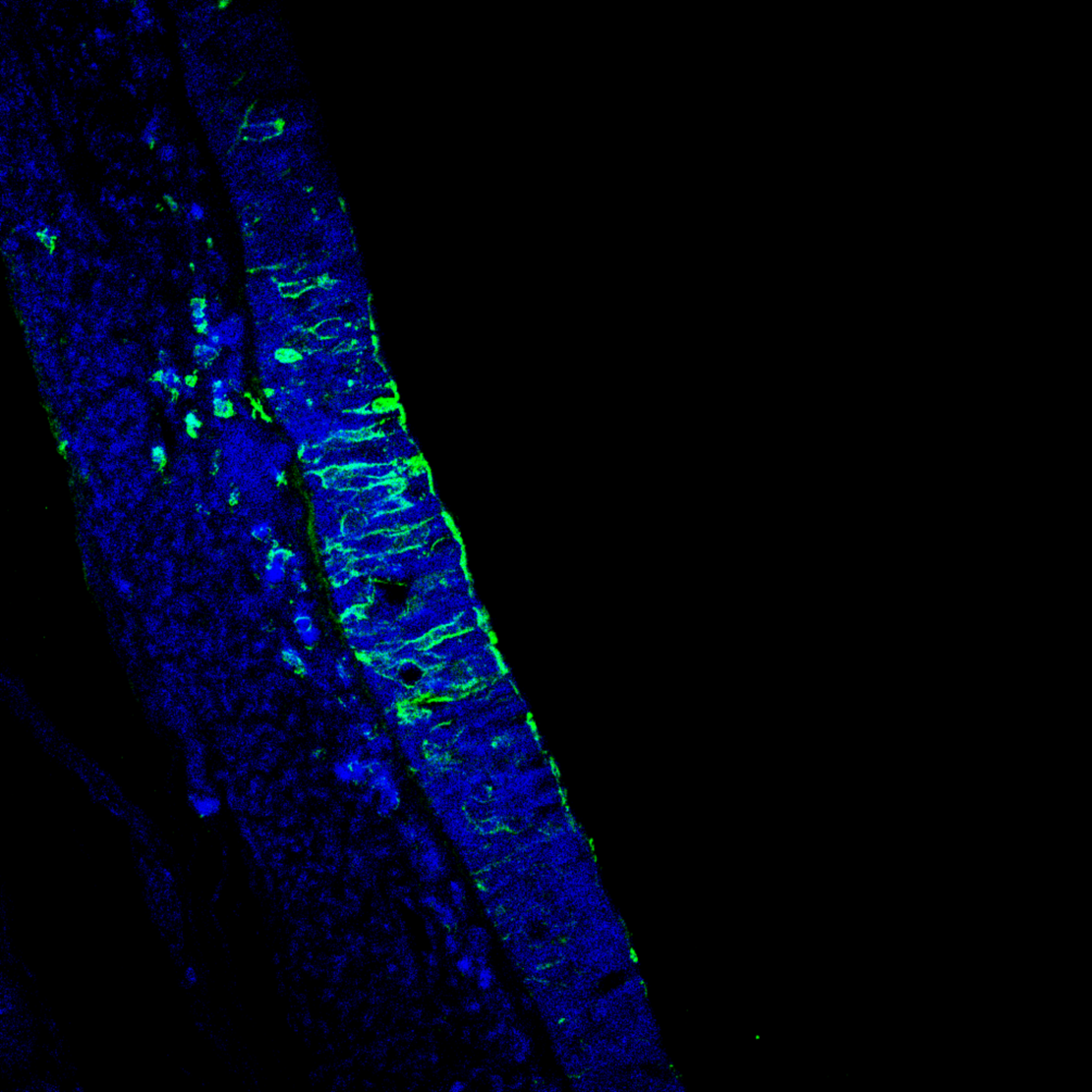

Supplement: Supplementary file 7 — Source data Fig. 3 [file 44319_2026_816_MOESM7_ESM.zip › Figure 3/3B/B-H1N1.tif]

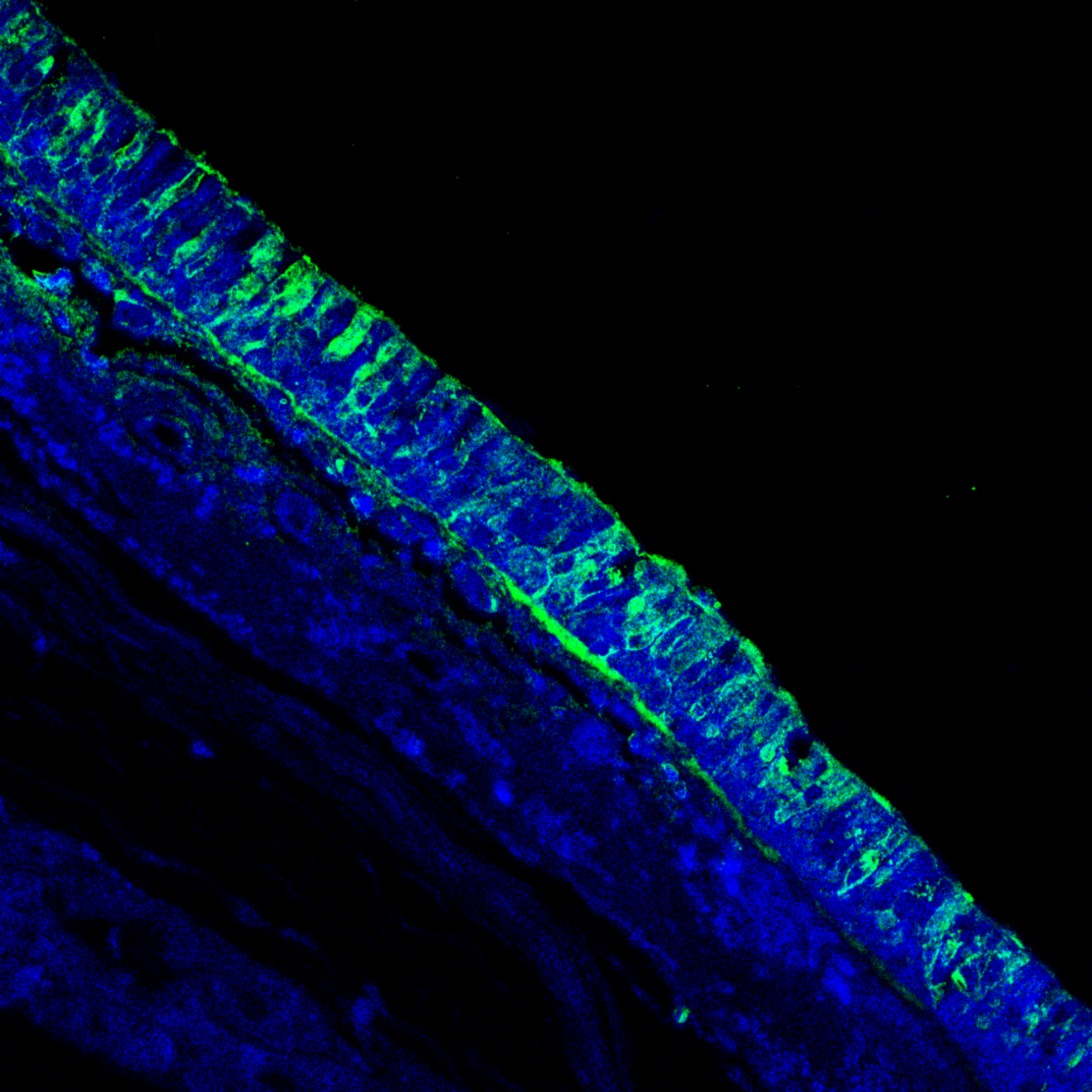

Supplement: Supplementary file 7 — Source data Fig. 3 [file 44319_2026_816_MOESM7_ESM.zip › Figure 3/3B/B-H3N2.tif]

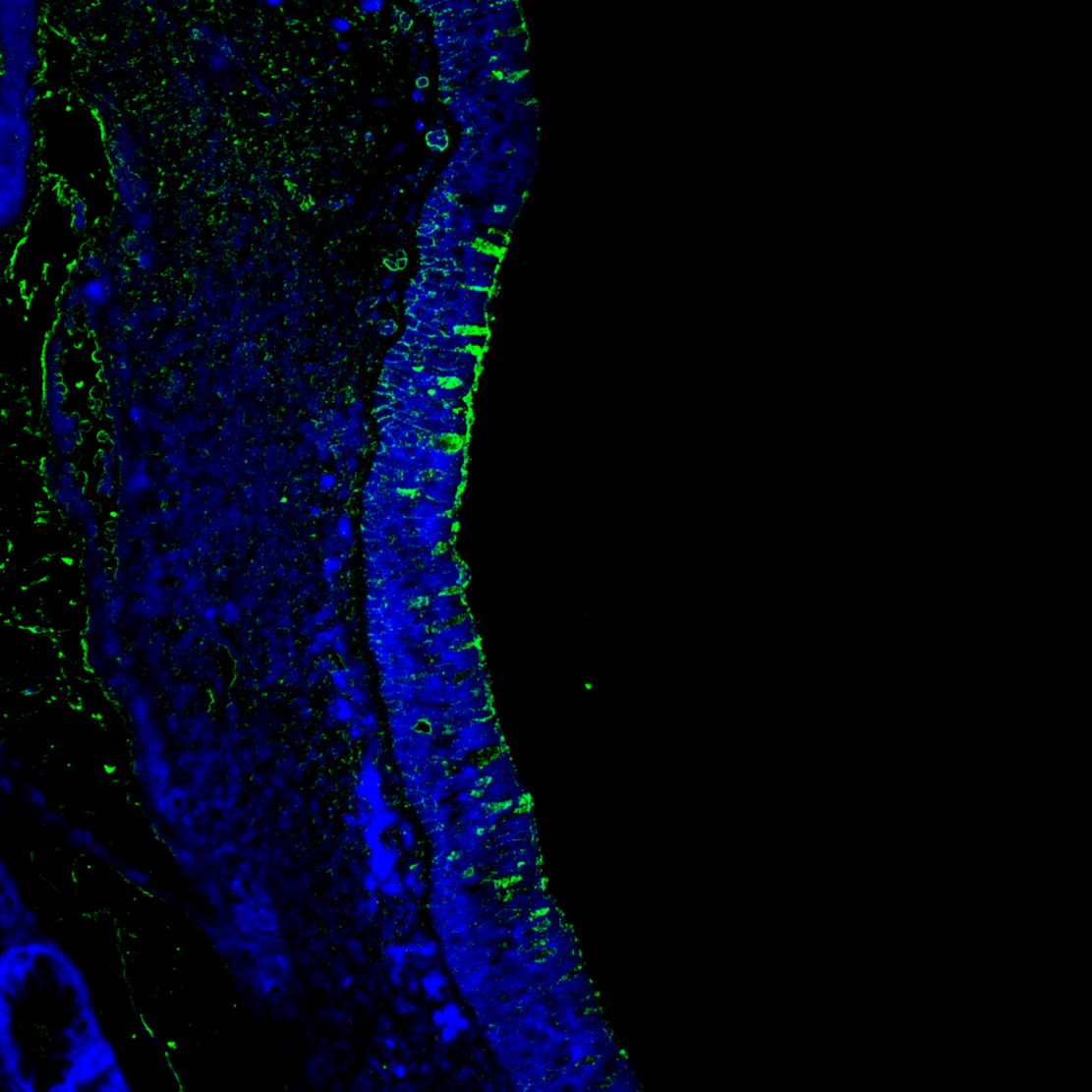

Supplement: Supplementary file 7 — Source data Fig. 3 [file 44319_2026_816_MOESM7_ESM.zip › Figure 3/3B/B-H5N8.tif]

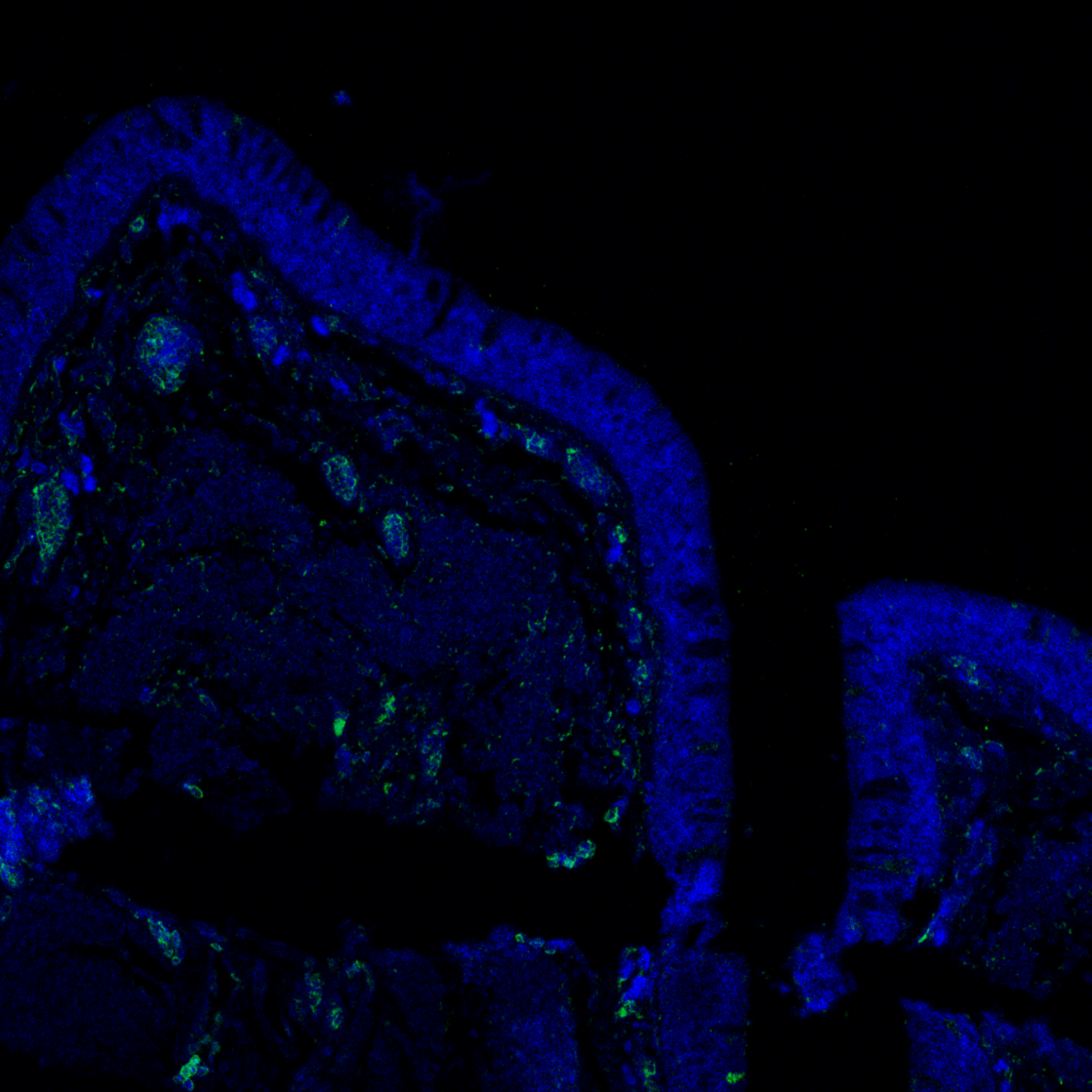

Supplement: Supplementary file 7 — Source data Fig. 3 [file 44319_2026_816_MOESM7_ESM.zip › Figure 3/3B/B-lnH5.tif]

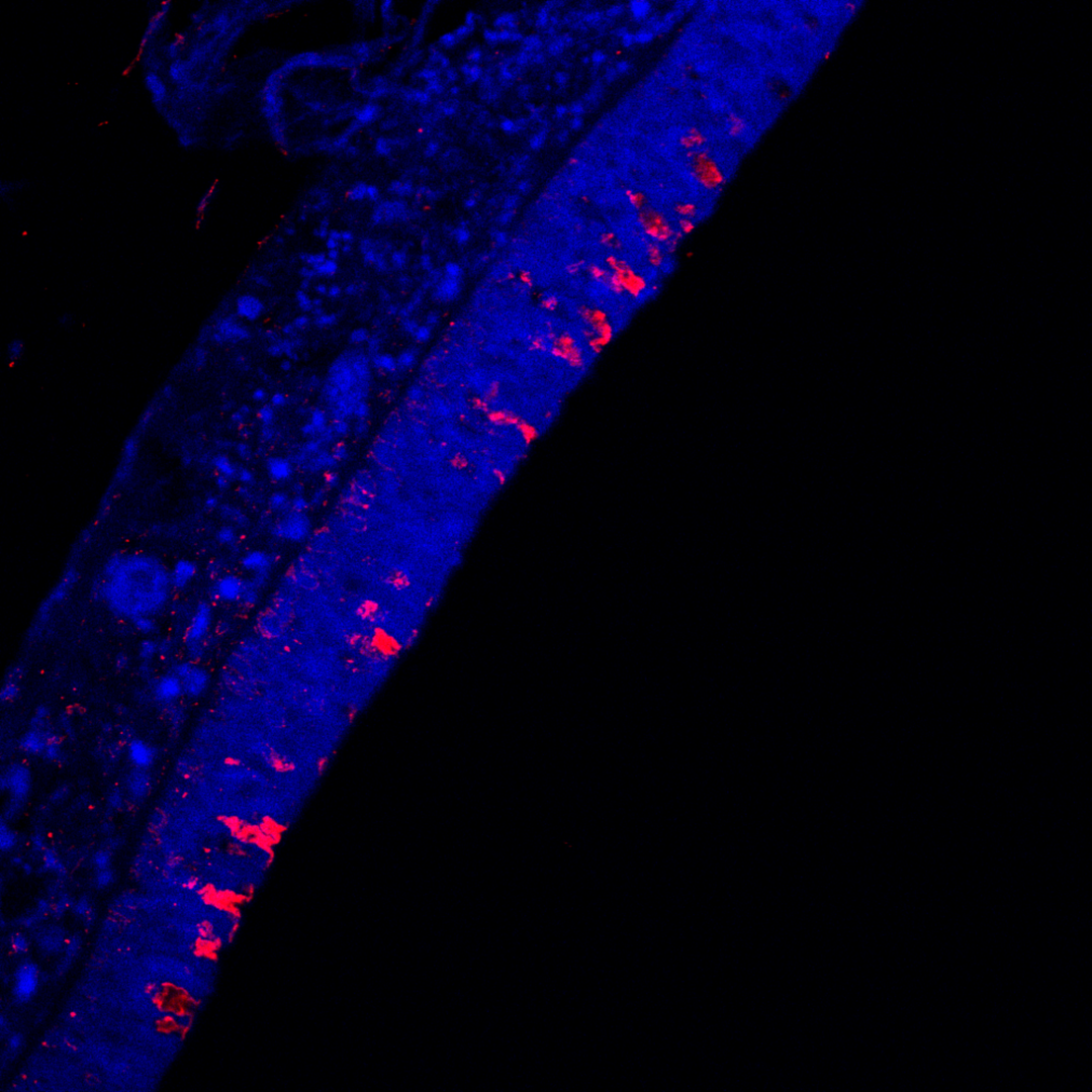

Supplement: Supplementary file 7 — Source data Fig. 3 [file 44319_2026_816_MOESM7_ESM.zip › Figure 3/3B/B-SLEX.tif]

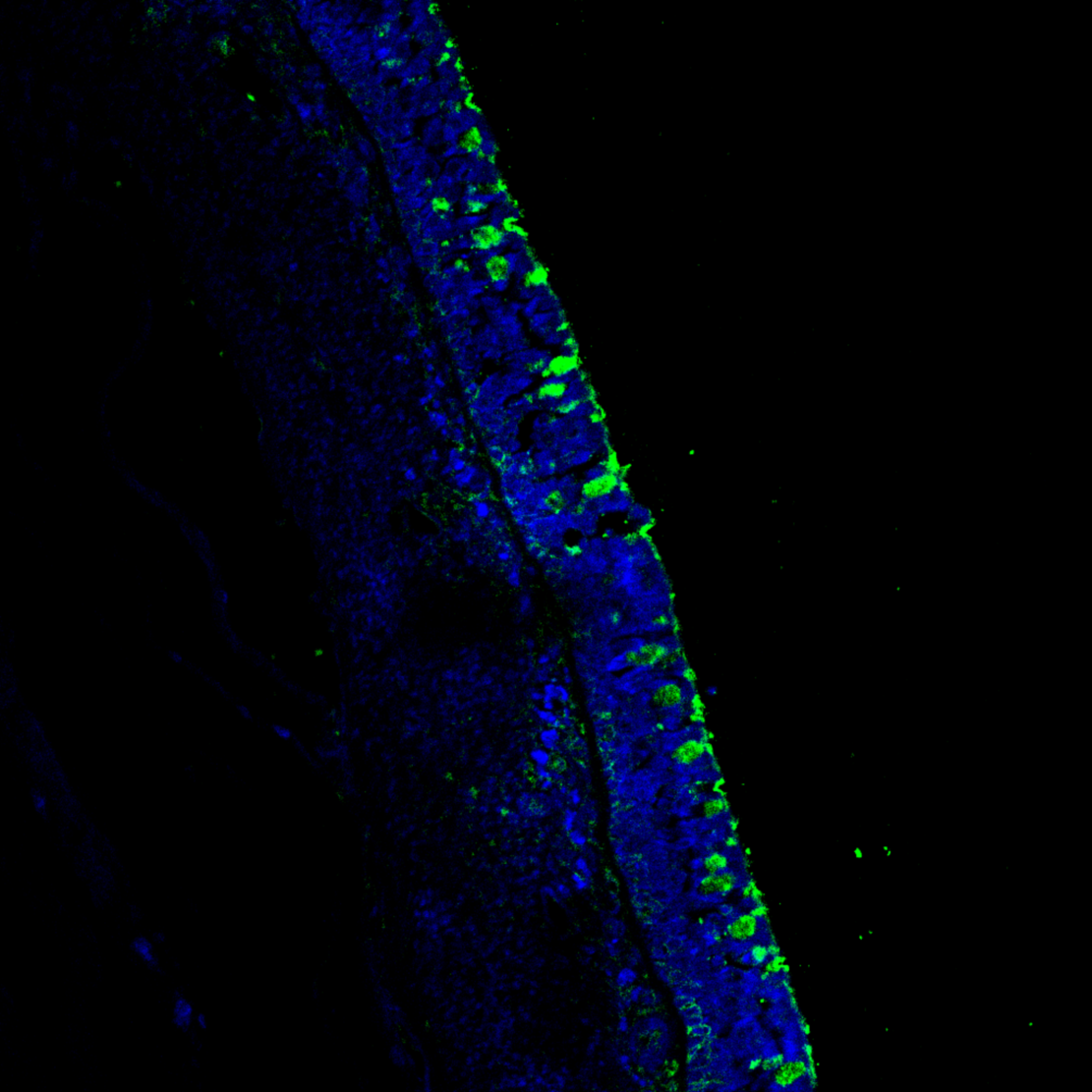

Supplement: Supplementary file 7 — Source data Fig. 3 [file 44319_2026_816_MOESM7_ESM.zip › Figure 3/3B/B-TxH5N1.tif]

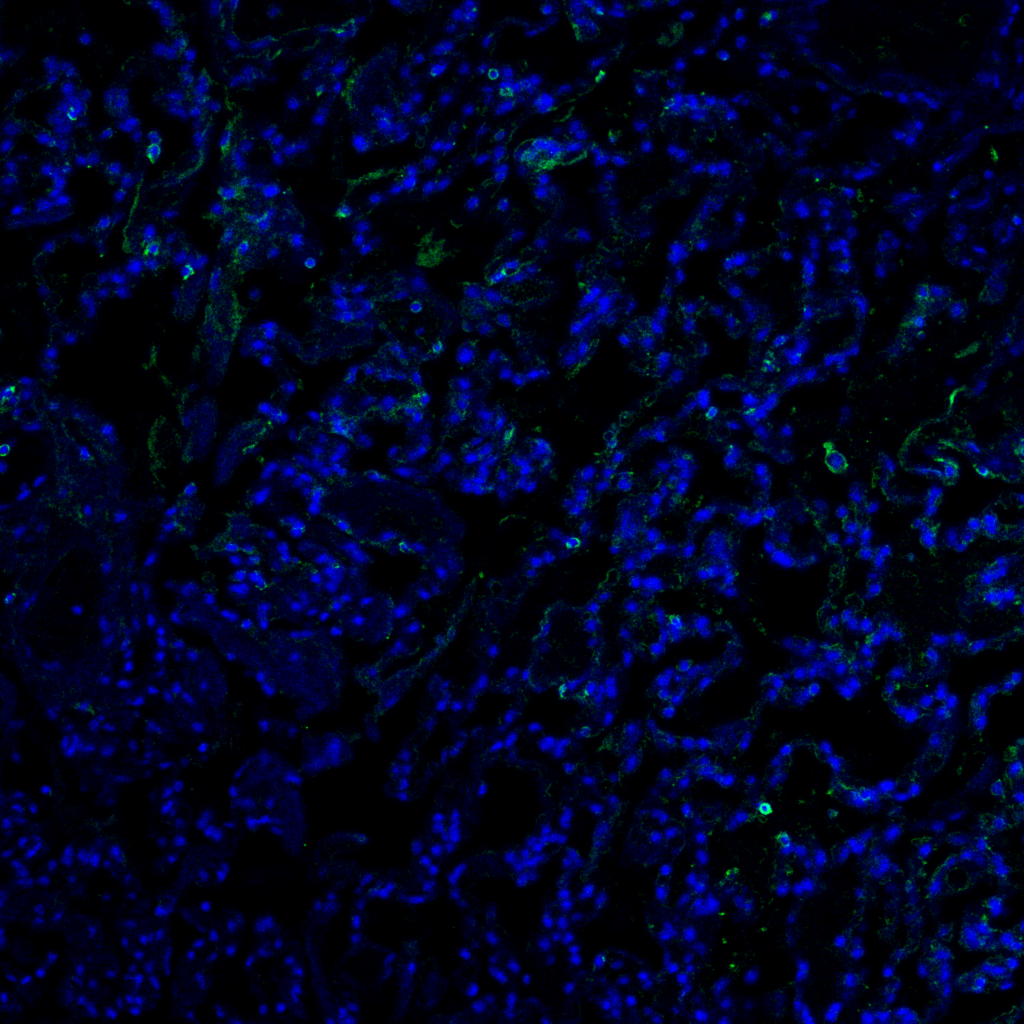

Supplement: Supplementary file 7 — Source data Fig. 3 [file 44319_2026_816_MOESM7_ESM.zip › Figure 3/3C/C-H1N1.tif]

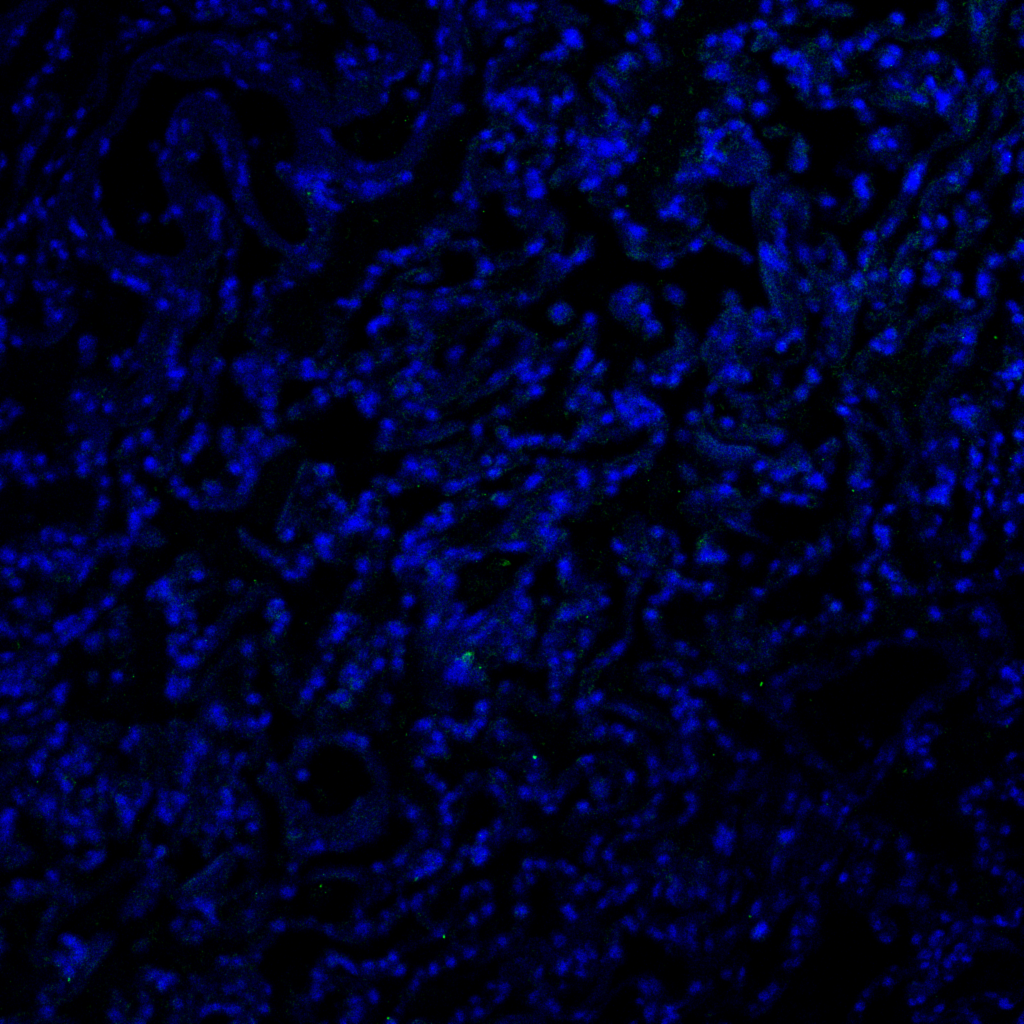

Supplement: Supplementary file 7 — Source data Fig. 3 [file 44319_2026_816_MOESM7_ESM.zip › Figure 3/3C/C-H3N2.tif]

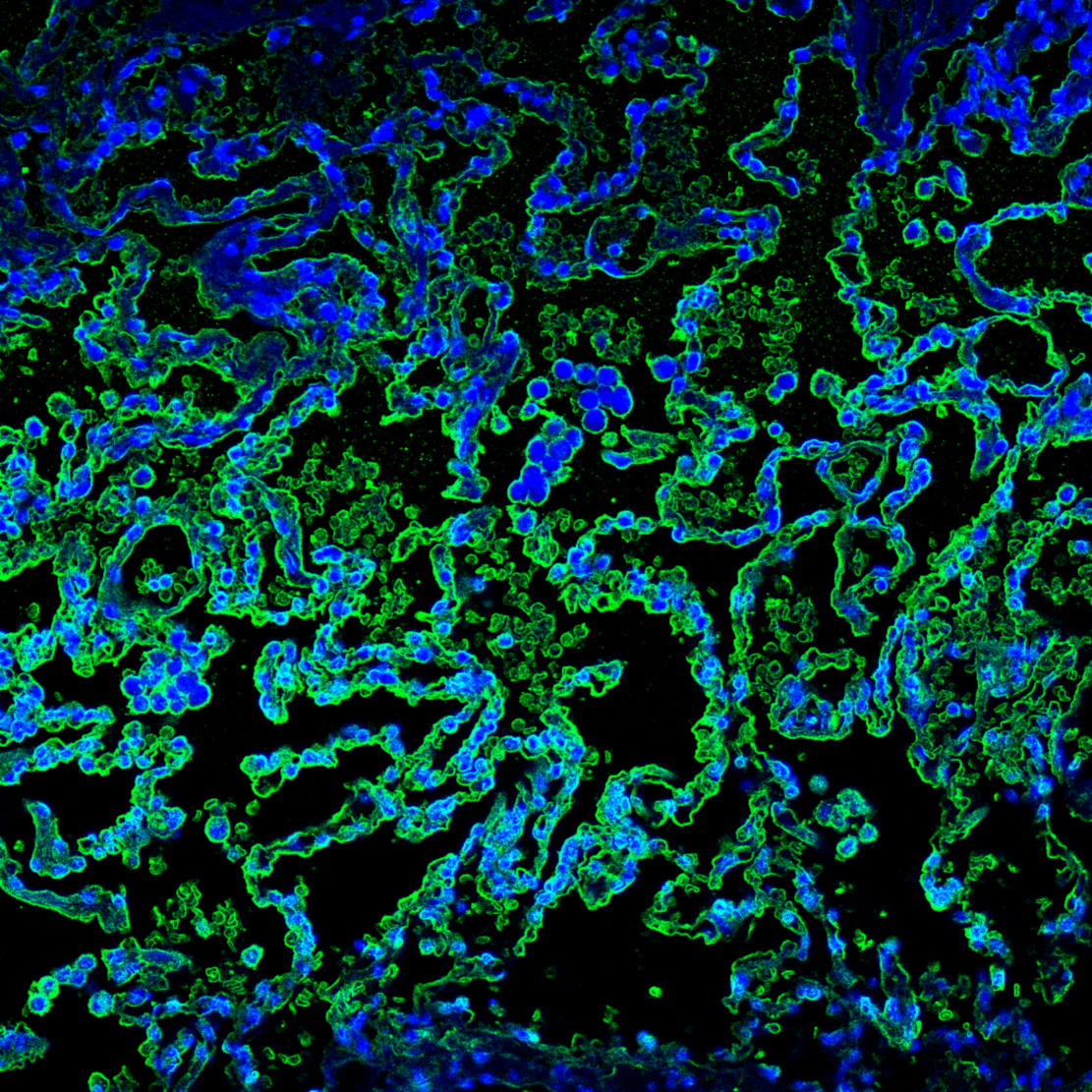

Supplement: Supplementary file 7 — Source data Fig. 3 [file 44319_2026_816_MOESM7_ESM.zip › Figure 3/3C/C-H5N8.tif]

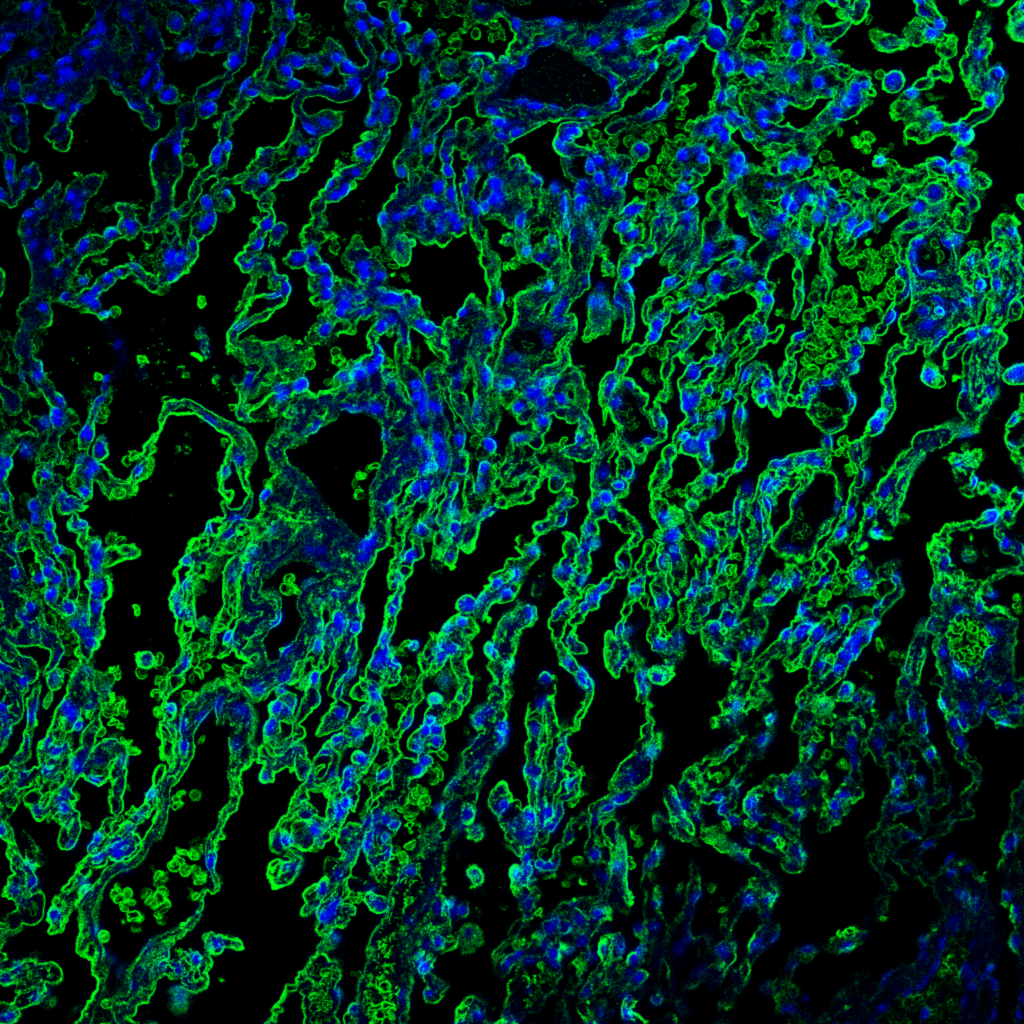

Supplement: Supplementary file 7 — Source data Fig. 3 [file 44319_2026_816_MOESM7_ESM.zip › Figure 3/3C/C-lnH5.tif]

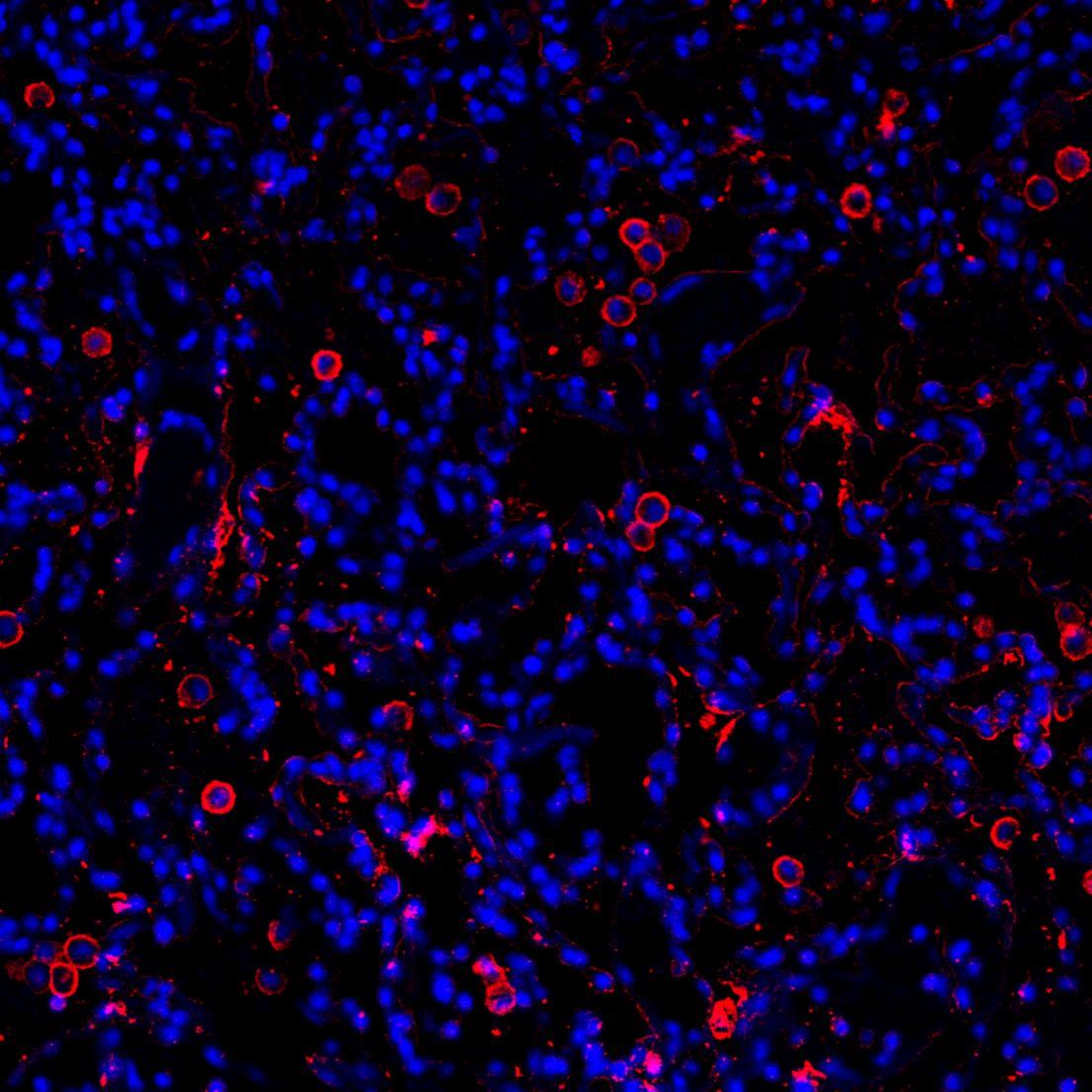

Supplement: Supplementary file 7 — Source data Fig. 3 [file 44319_2026_816_MOESM7_ESM.zip › Figure 3/3C/C-SLEX.tif]

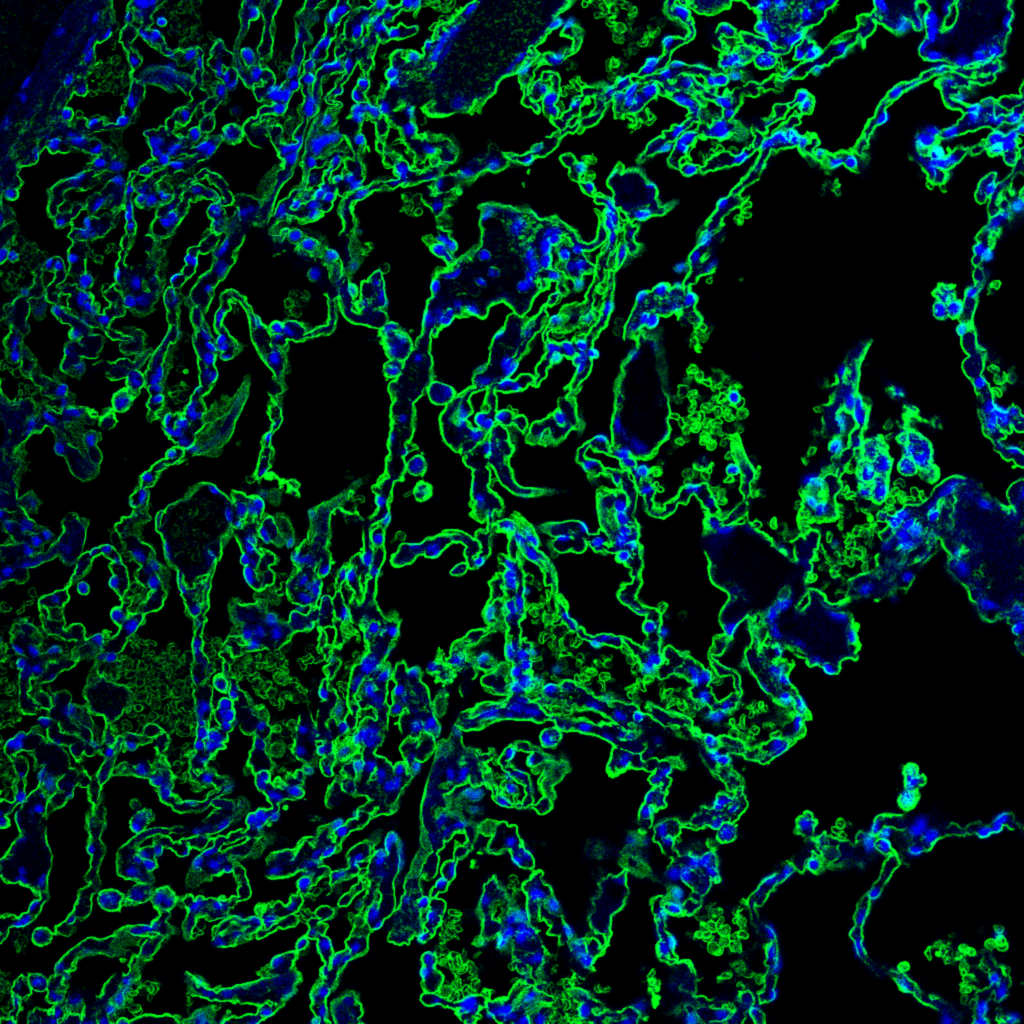

Supplement: Supplementary file 7 — Source data Fig. 3 [file 44319_2026_816_MOESM7_ESM.zip › Figure 3/3C/C-TxH5N1.tif]

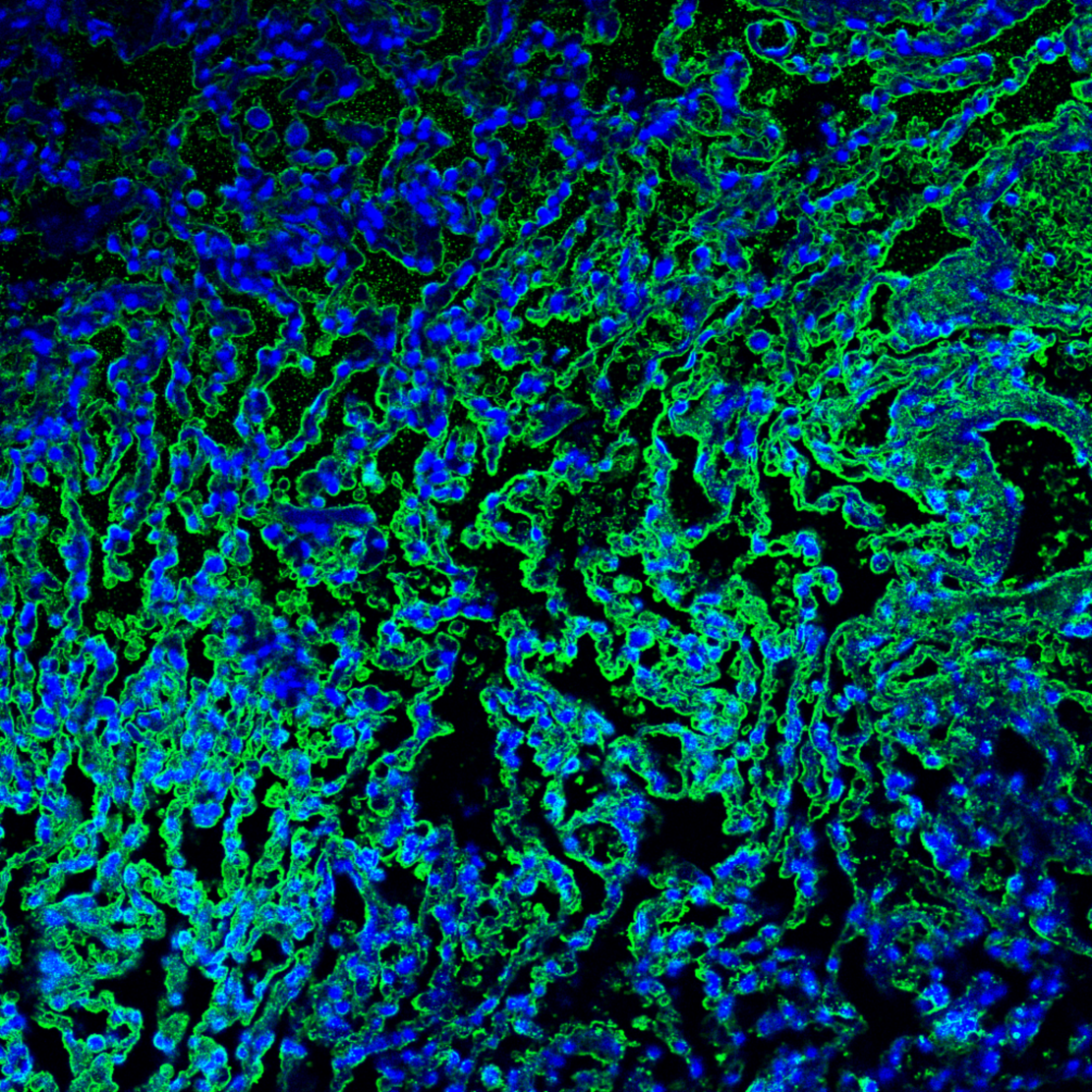

Supplement: Supplementary file 7 — Source data Fig. 3 [file 44319_2026_816_MOESM7_ESM.zip › Figure 3/3C/C-wsH5N8.tif]

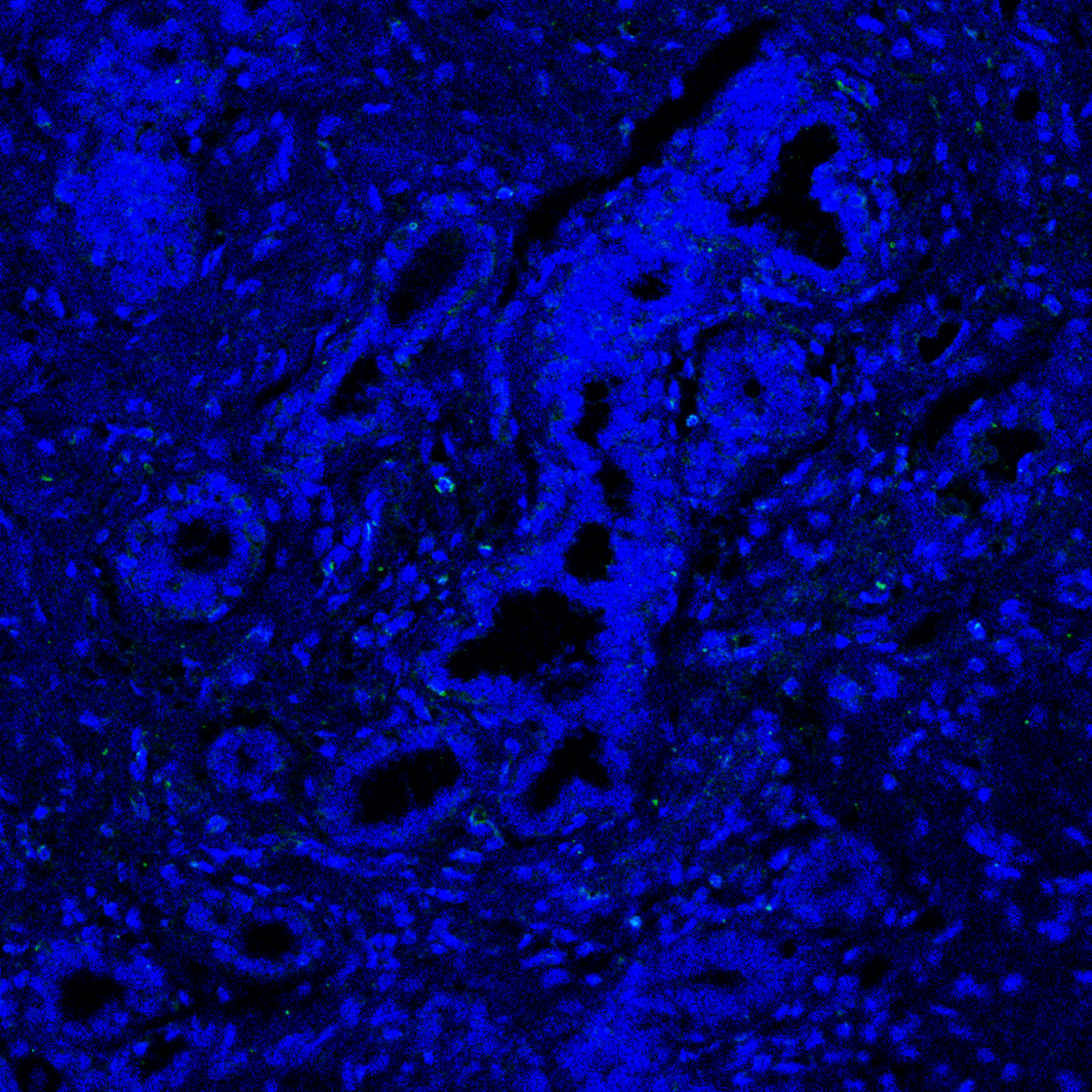

Supplement: Supplementary file 7 — Source data Fig. 3 [file 44319_2026_816_MOESM7_ESM.zip › Figure 3/3D/D-H1N1.tif]

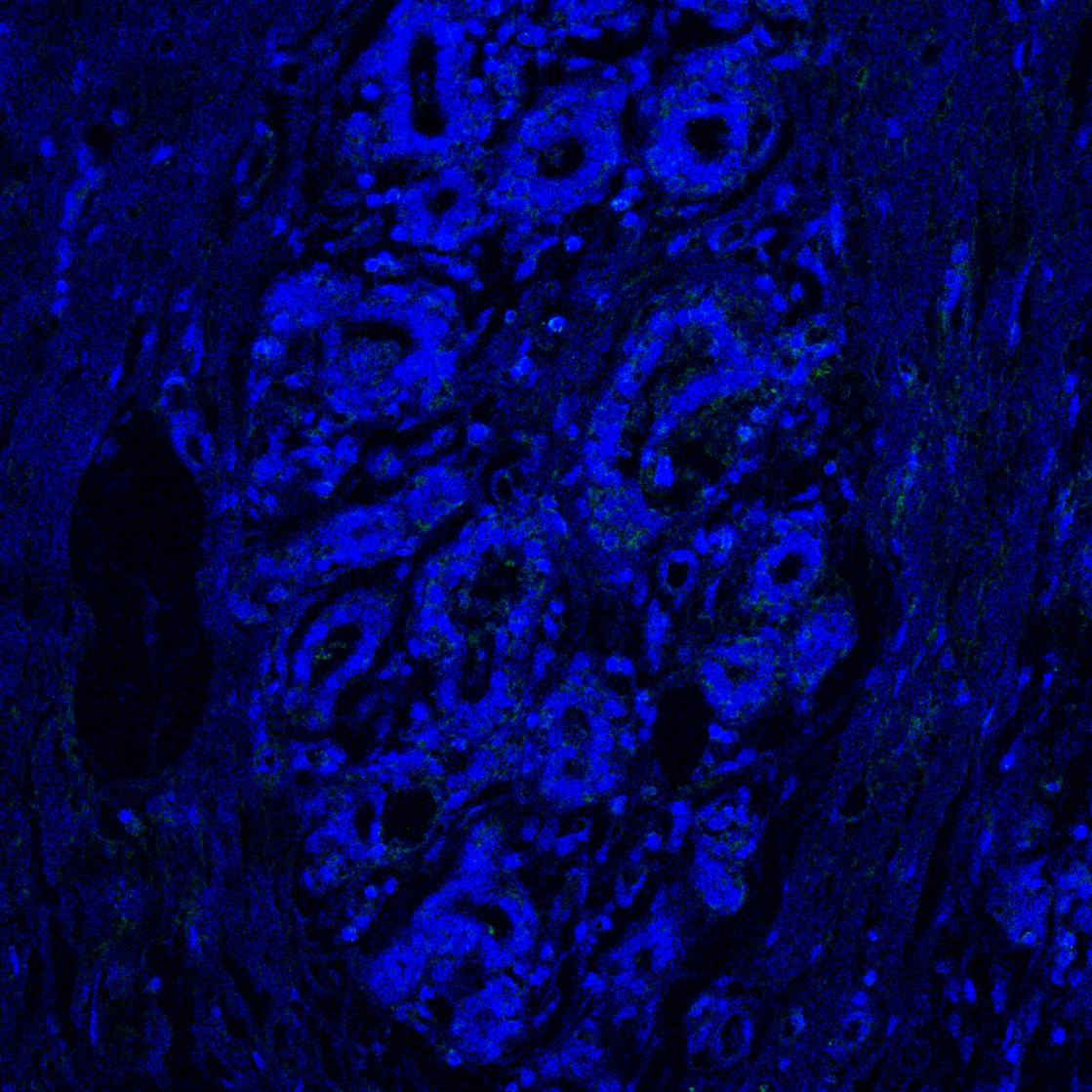

Supplement: Supplementary file 7 — Source data Fig. 3 [file 44319_2026_816_MOESM7_ESM.zip › Figure 3/3D/D-H3N2.tif]

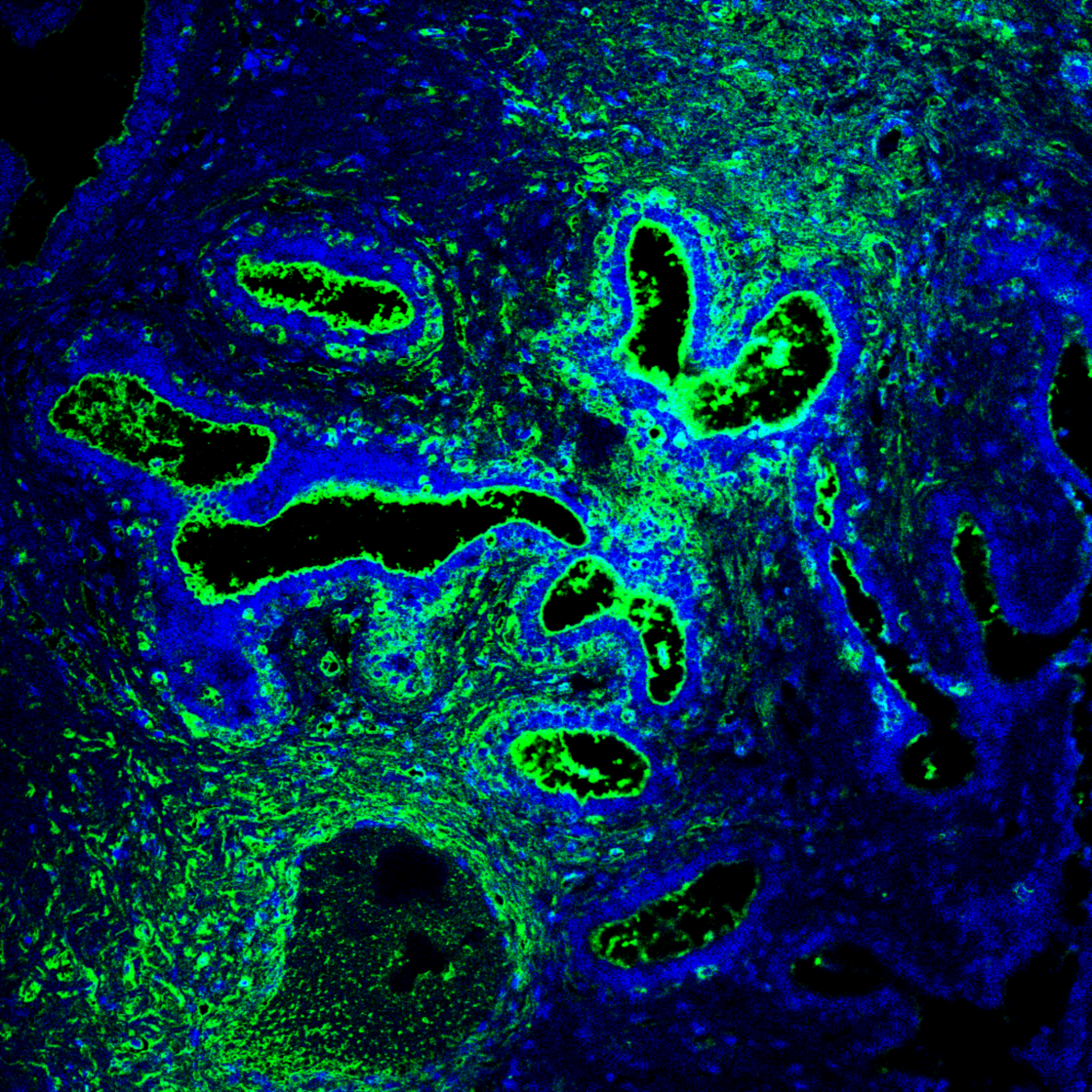

Supplement: Supplementary file 7 — Source data Fig. 3 [file 44319_2026_816_MOESM7_ESM.zip › Figure 3/3D/D-H5N8.tif]

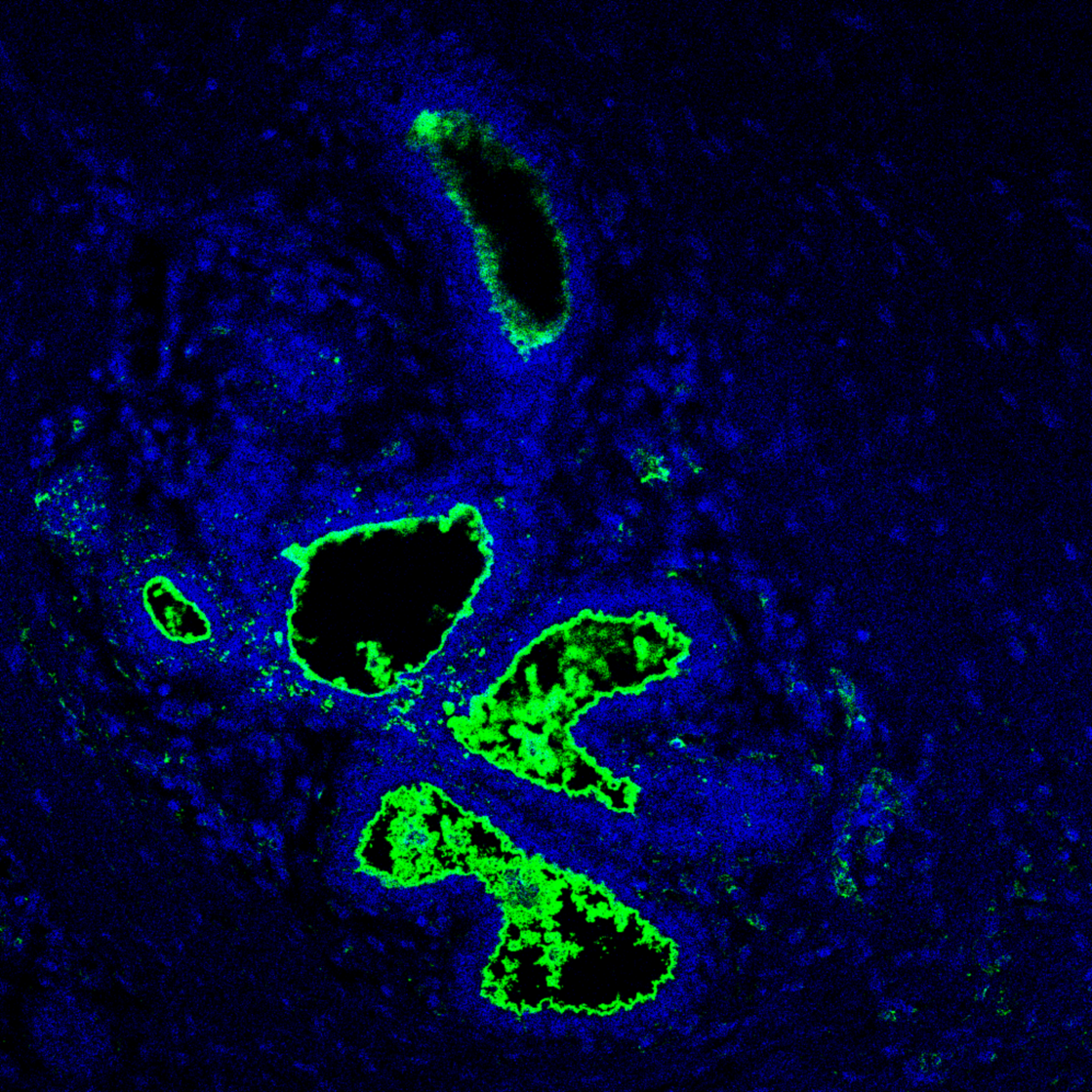

Supplement: Supplementary file 7 — Source data Fig. 3 [file 44319_2026_816_MOESM7_ESM.zip › Figure 3/3D/D-lnH5.tif]

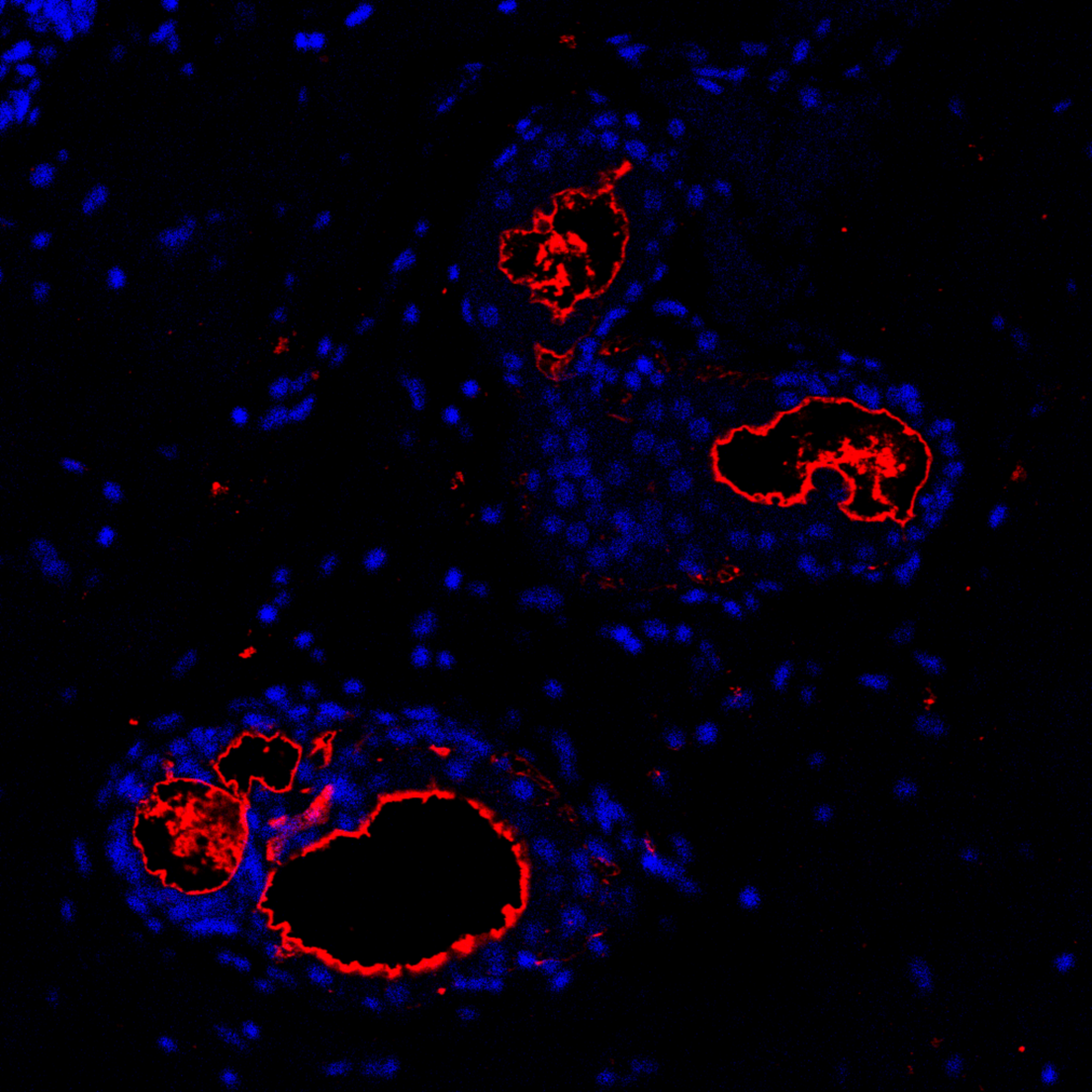

Supplement: Supplementary file 7 — Source data Fig. 3 [file 44319_2026_816_MOESM7_ESM.zip › Figure 3/3D/D-SLEX.tif]

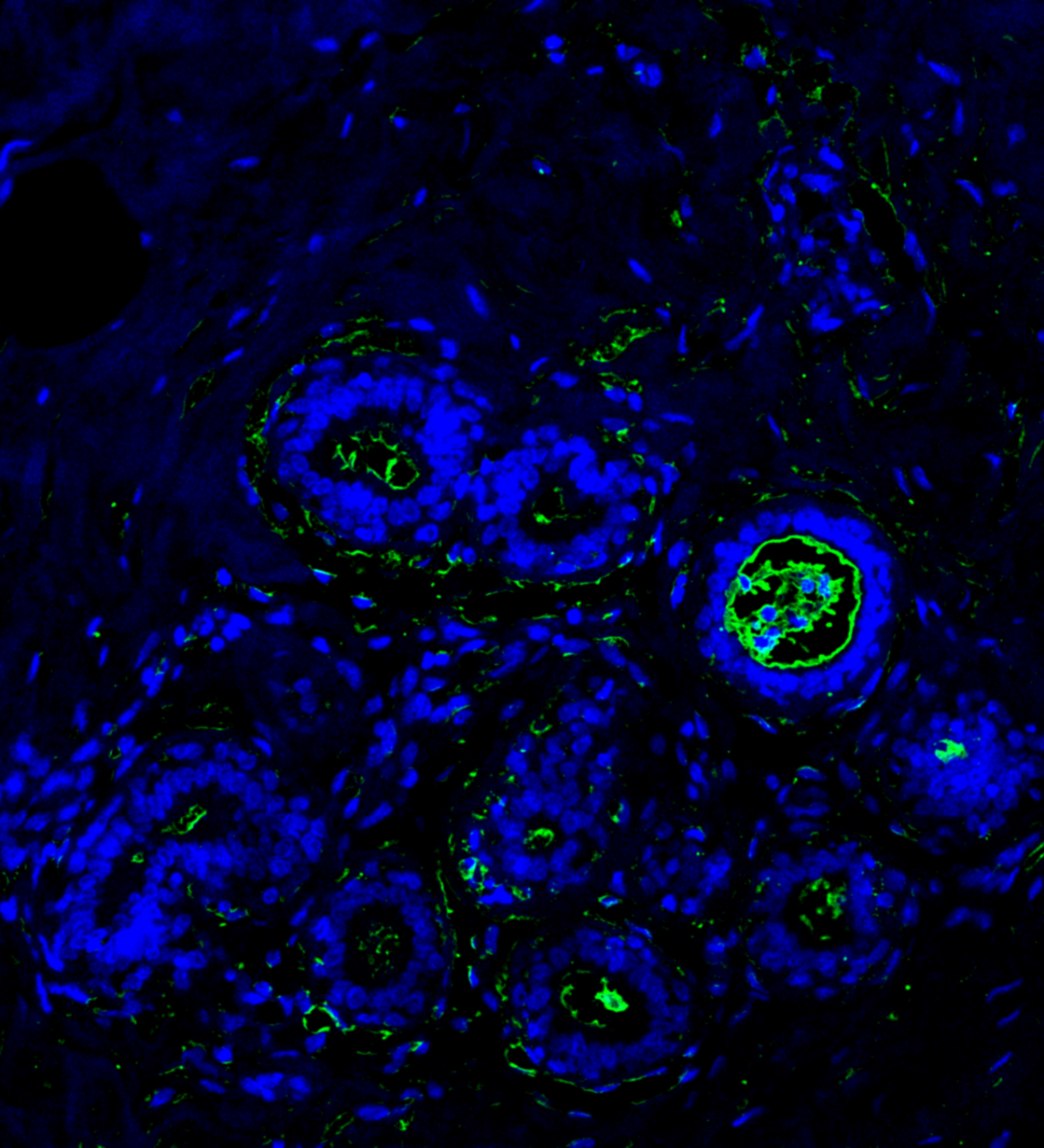

Supplement: Supplementary file 7 — Source data Fig. 3 [file 44319_2026_816_MOESM7_ESM.zip › Figure 3/3D/D-TxH5N1.tif]

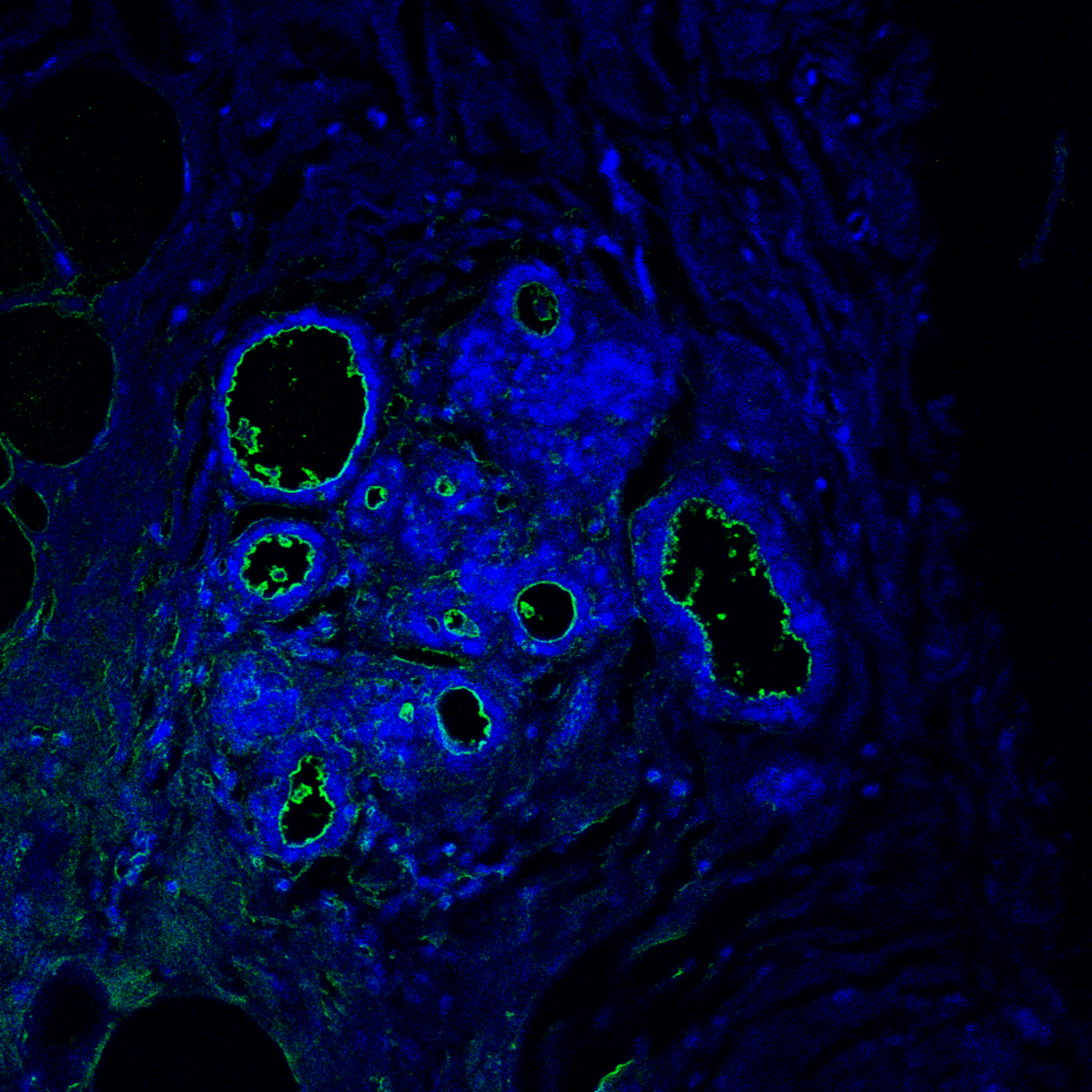

Supplement: Supplementary file 7 — Source data Fig. 3 [file 44319_2026_816_MOESM7_ESM.zip › Figure 3/3D/D-wsH5N8.tif]

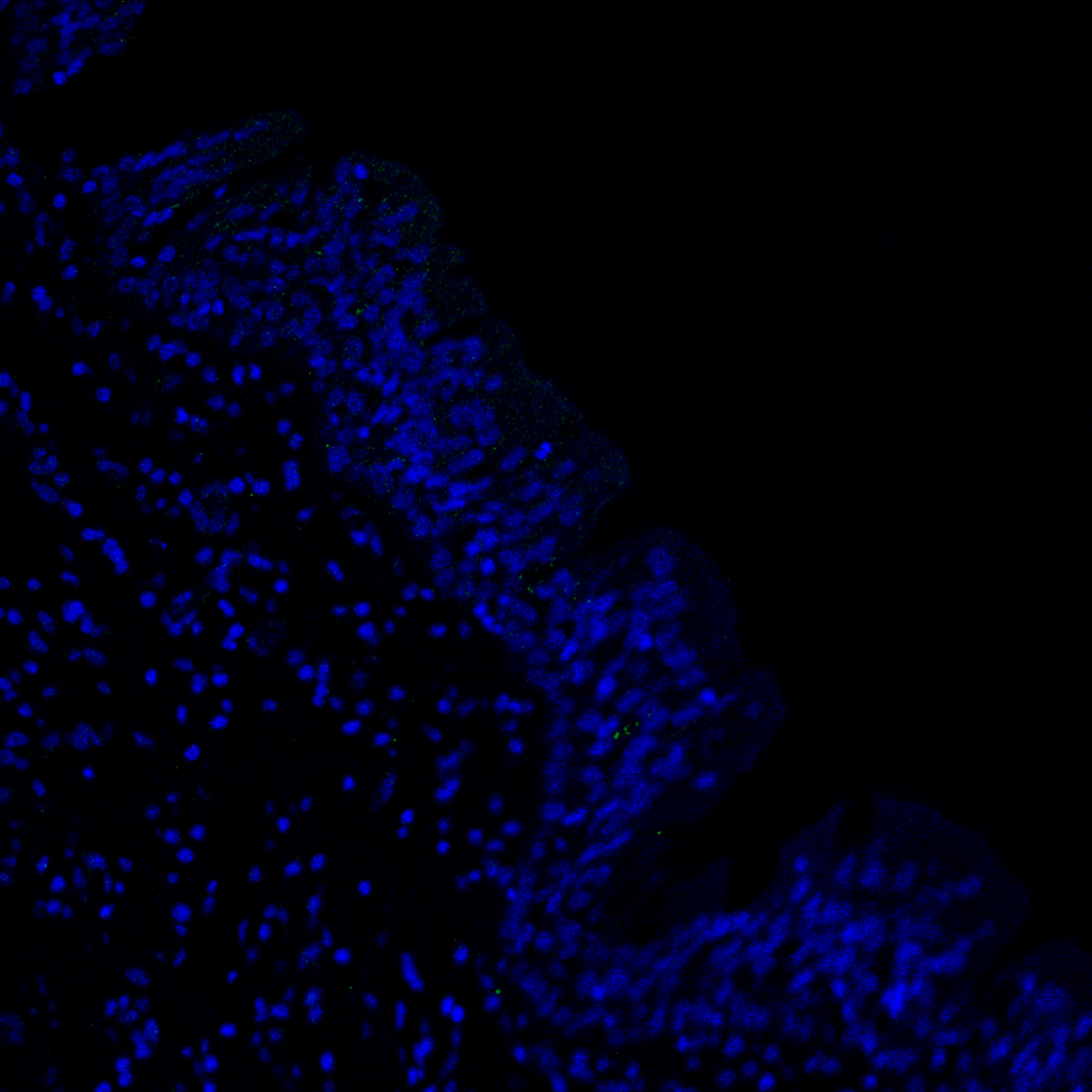

Supplement: Supplementary file 7 — Source data Fig. 3 [file 44319_2026_816_MOESM7_ESM.zip › Figure 3/3E/E-H1N1.tif]

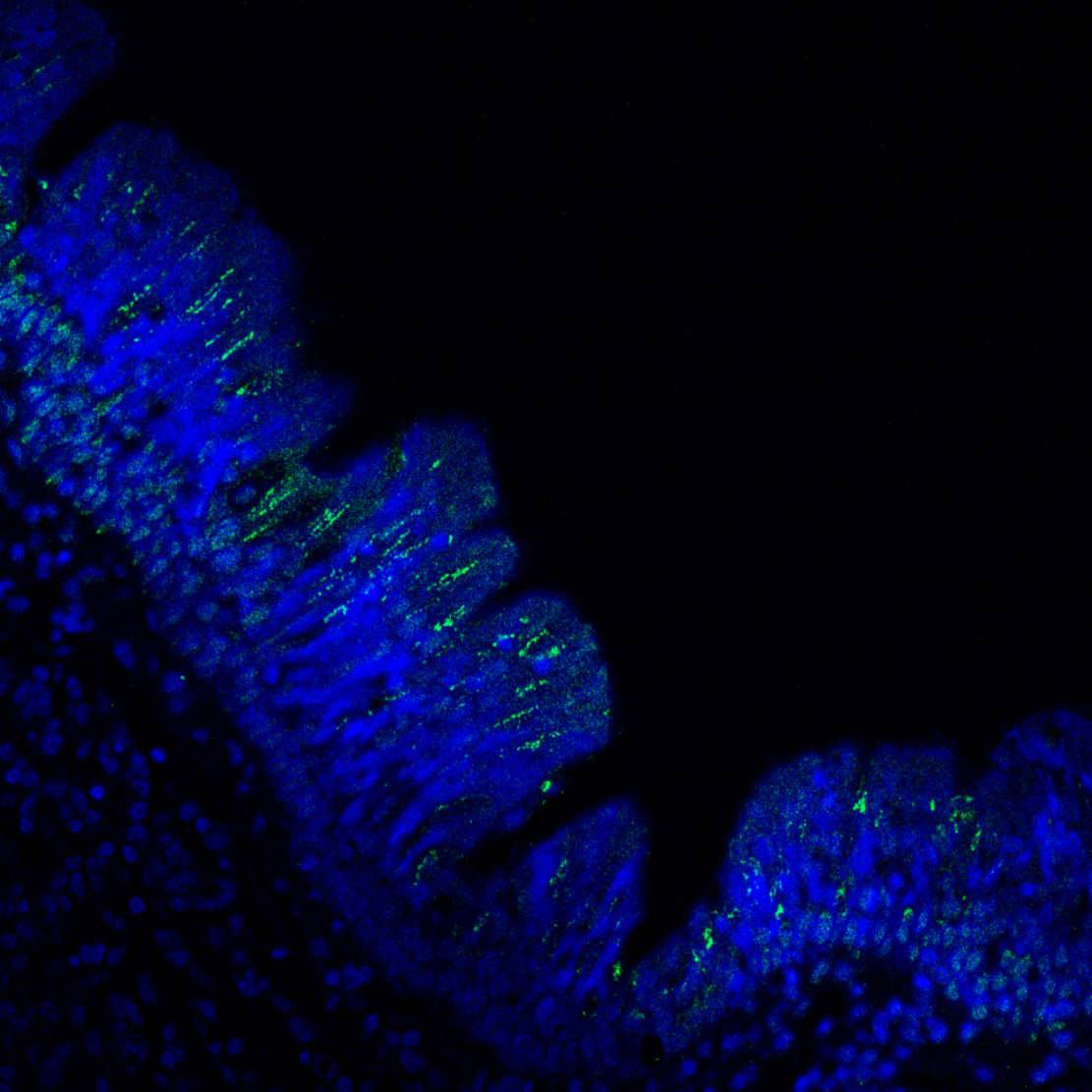

Supplement: Supplementary file 7 — Source data Fig. 3 [file 44319_2026_816_MOESM7_ESM.zip › Figure 3/3E/E-H3N2.tif]

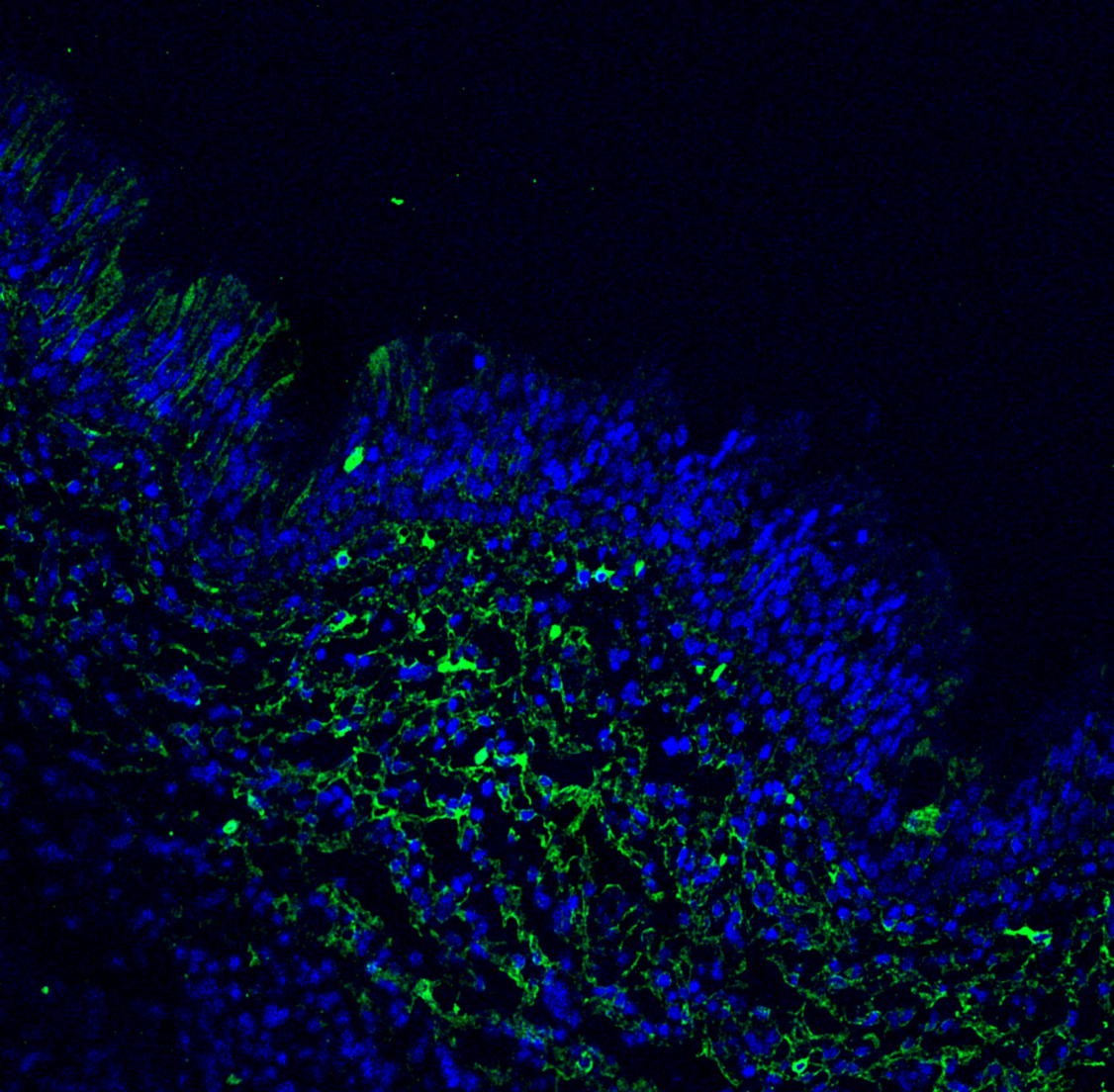

Supplement: Supplementary file 7 — Source data Fig. 3 [file 44319_2026_816_MOESM7_ESM.zip › Figure 3/3E/E-H5N8.tif]

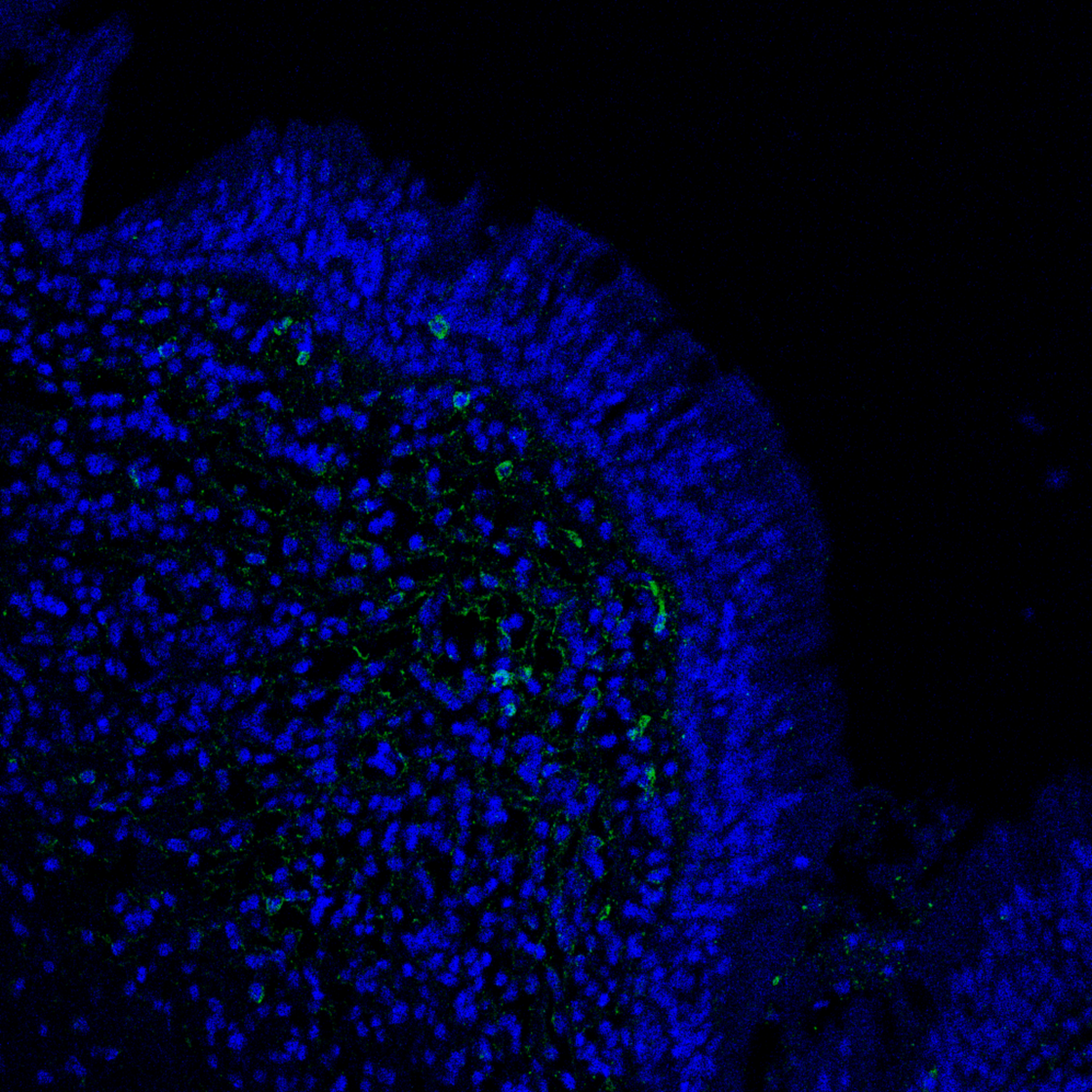

Supplement: Supplementary file 7 — Source data Fig. 3 [file 44319_2026_816_MOESM7_ESM.zip › Figure 3/3E/E-lnH5.tif]

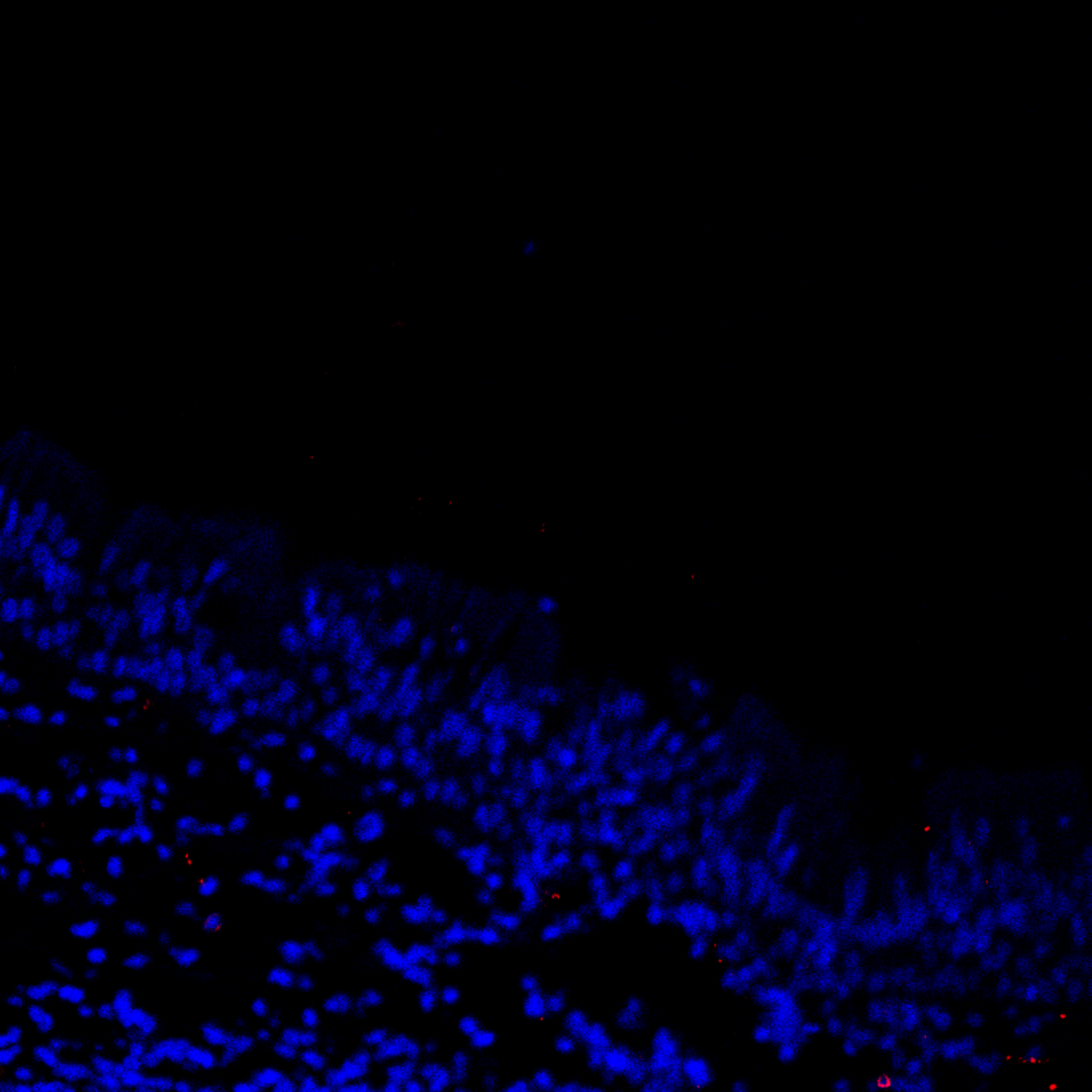

Supplement: Supplementary file 7 — Source data Fig. 3 [file 44319_2026_816_MOESM7_ESM.zip › Figure 3/3E/E-SLEX.tif]

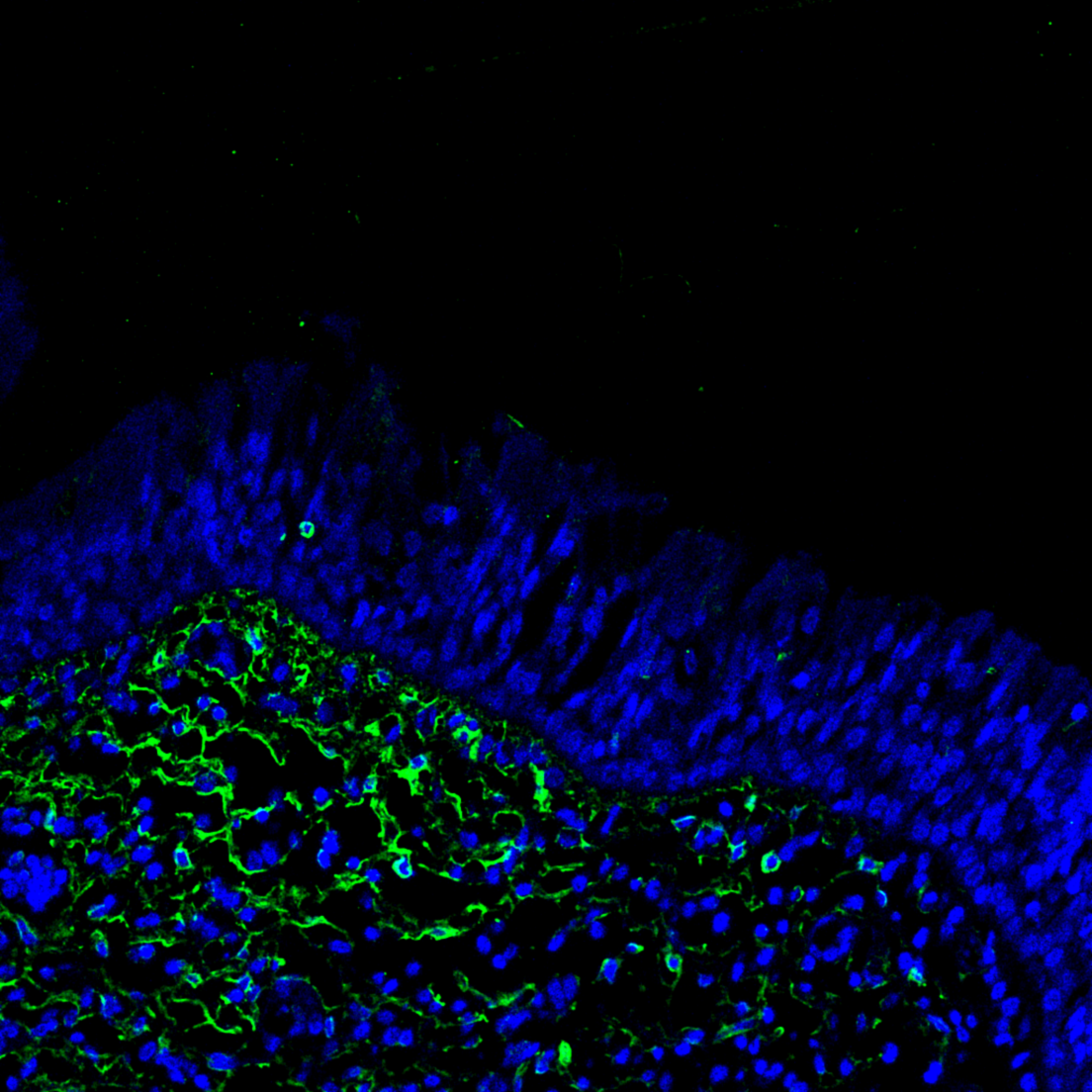

Supplement: Supplementary file 7 — Source data Fig. 3 [file 44319_2026_816_MOESM7_ESM.zip › Figure 3/3E/E-TxH5N1.tif]

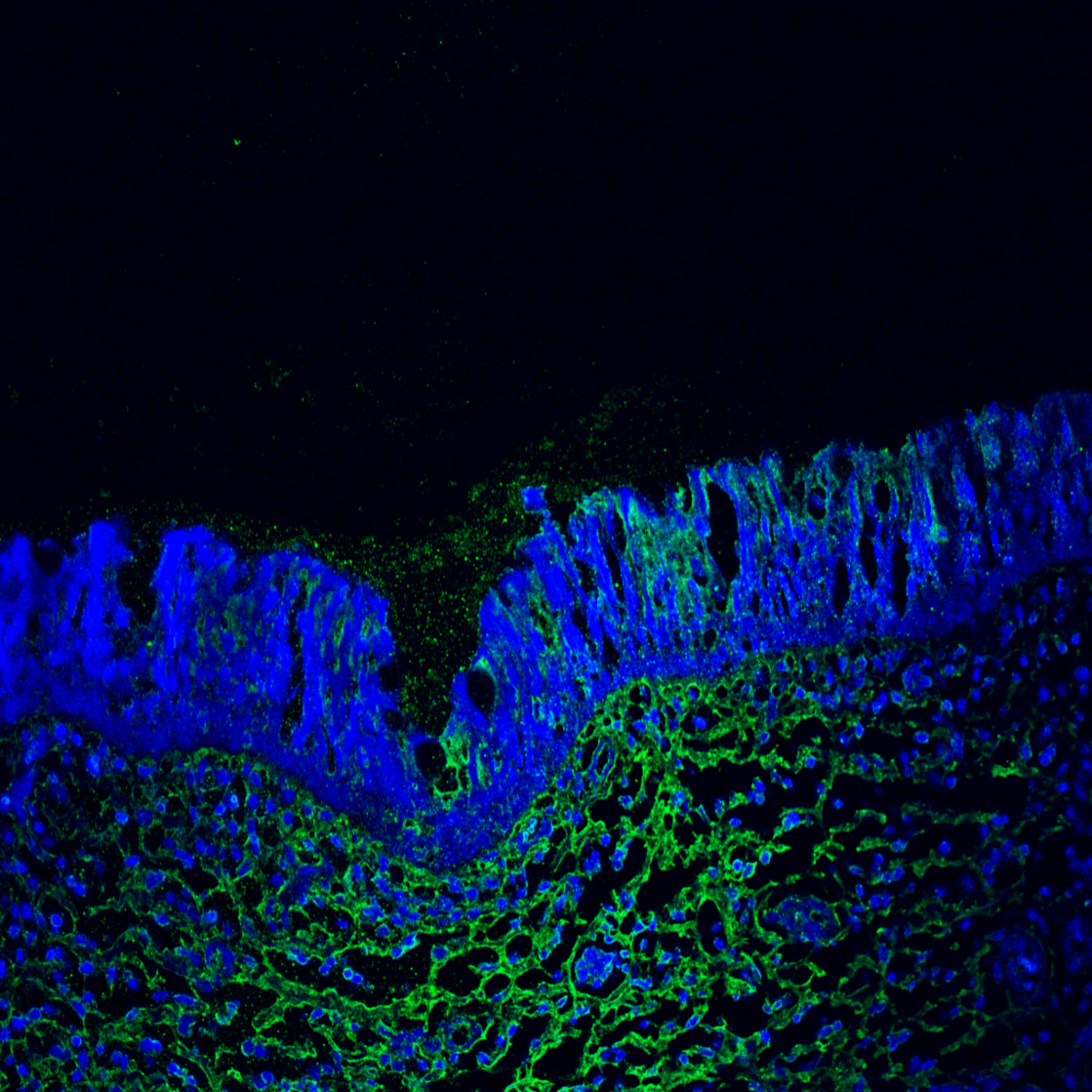

Supplement: Supplementary file 7 — Source data Fig. 3 [file 44319_2026_816_MOESM7_ESM.zip › Figure 3/3E/E-wsH5N8.tif]

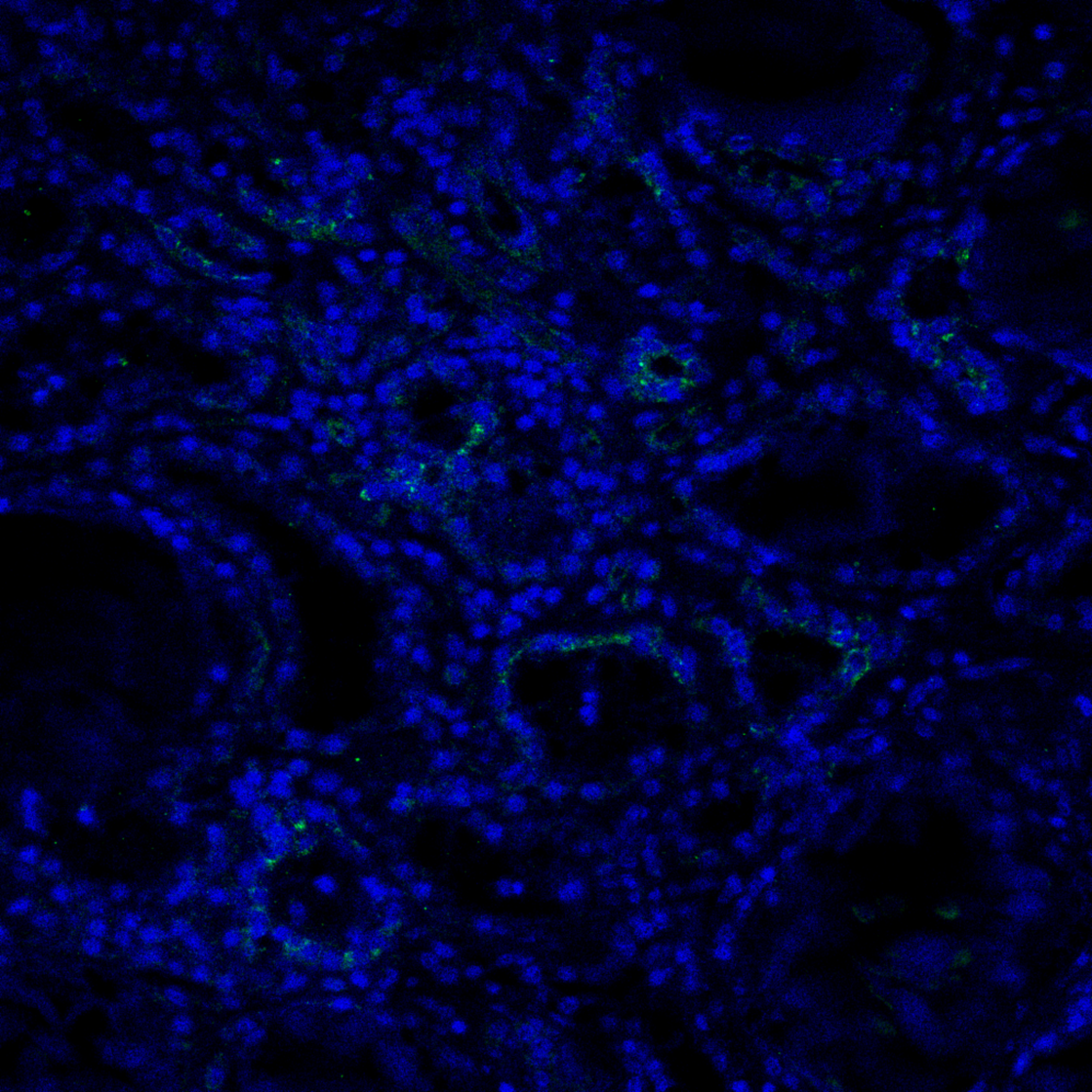

Supplement: Supplementary file 7 — Source data Fig. 3 [file 44319_2026_816_MOESM7_ESM.zip › Figure 3/3F/F-H1N1.tif]

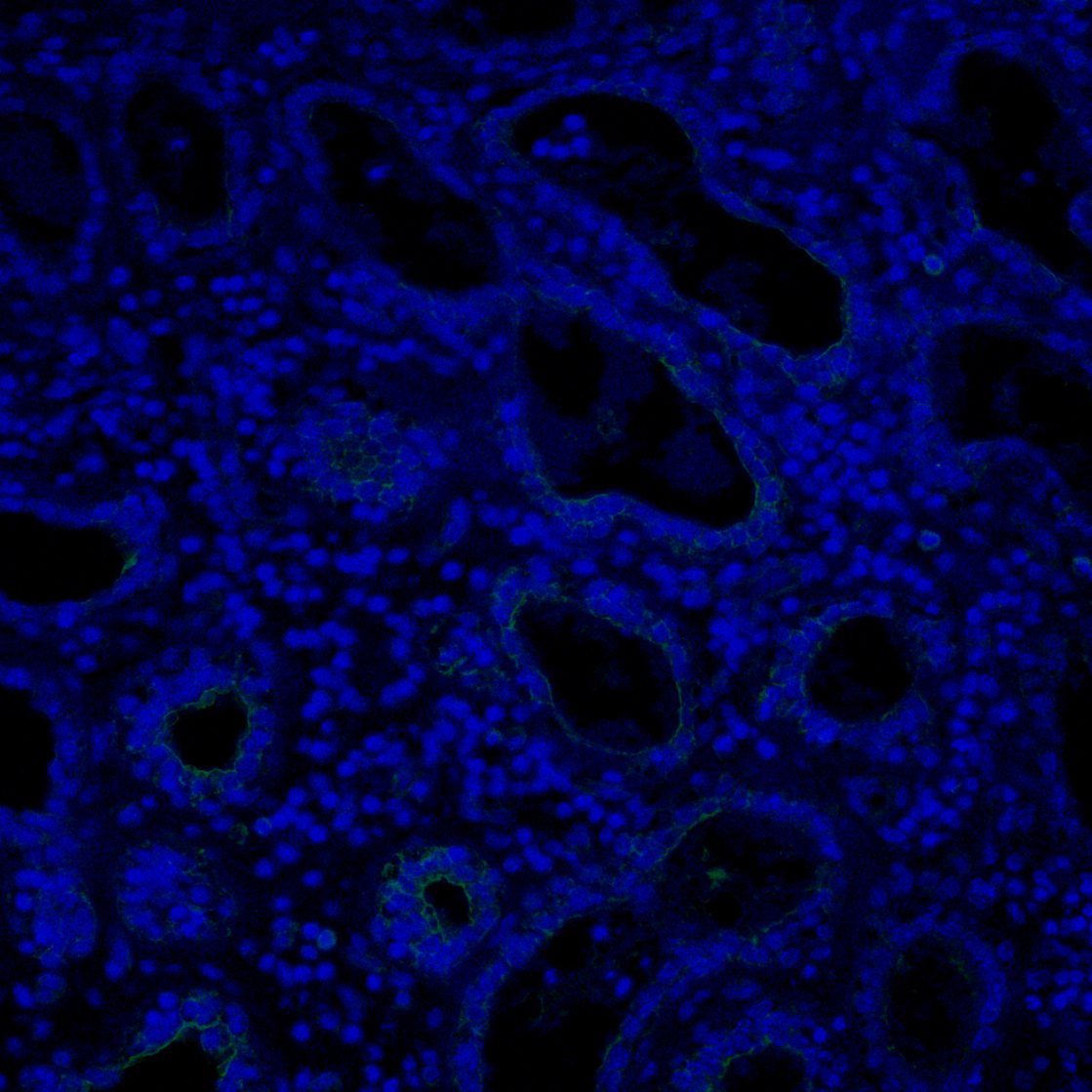

Supplement: Supplementary file 7 — Source data Fig. 3 [file 44319_2026_816_MOESM7_ESM.zip › Figure 3/3F/F-H3N2.tif]

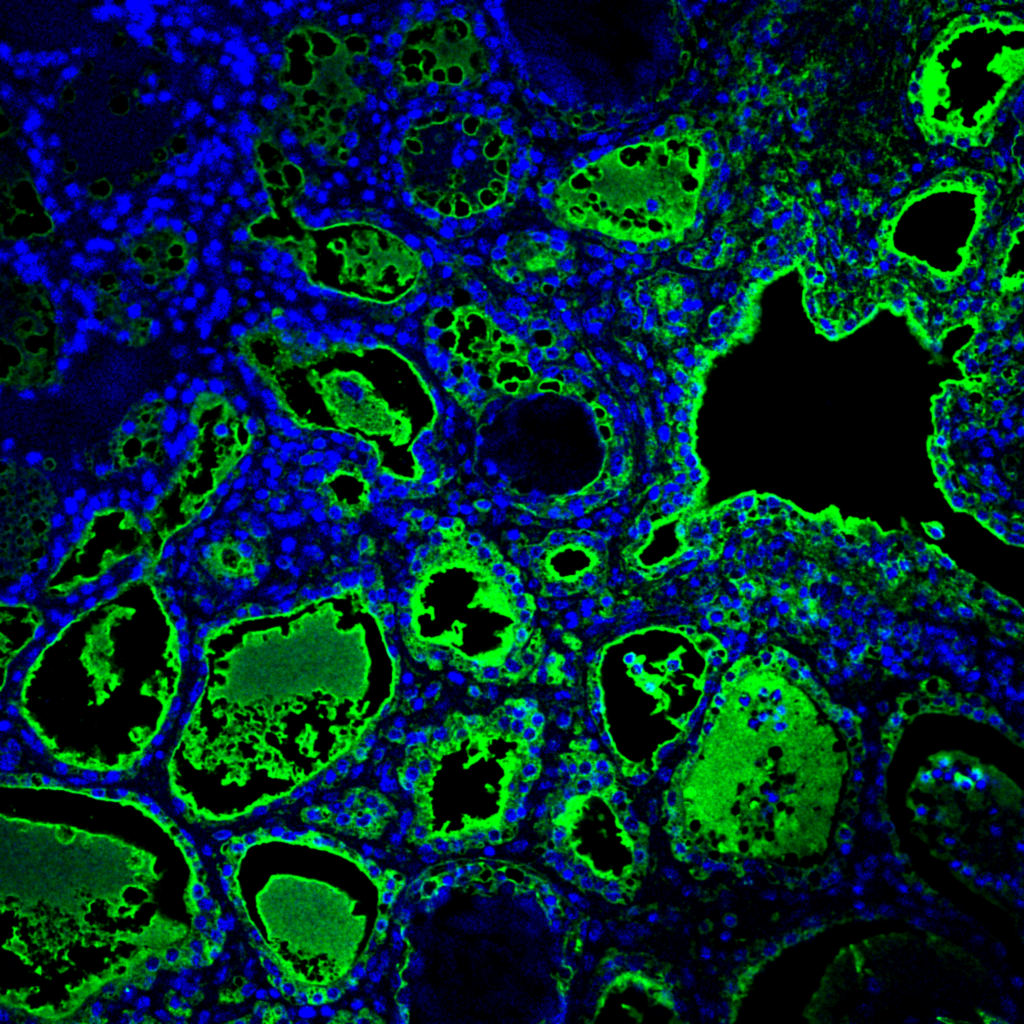

Supplement: Supplementary file 7 — Source data Fig. 3 [file 44319_2026_816_MOESM7_ESM.zip › Figure 3/3F/F-H5N8.tif]

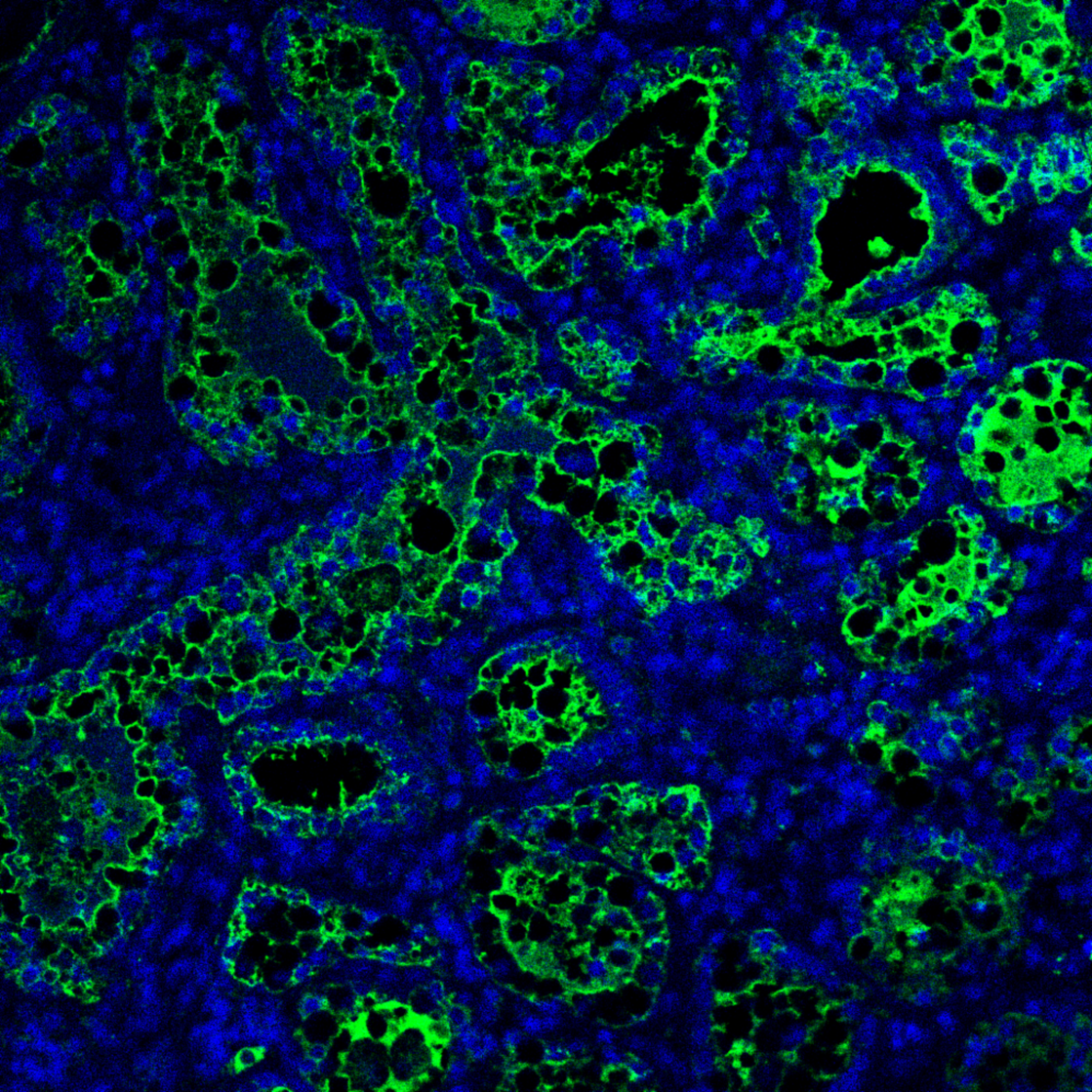

Supplement: Supplementary file 7 — Source data Fig. 3 [file 44319_2026_816_MOESM7_ESM.zip › Figure 3/3F/F-lnH5.tif]

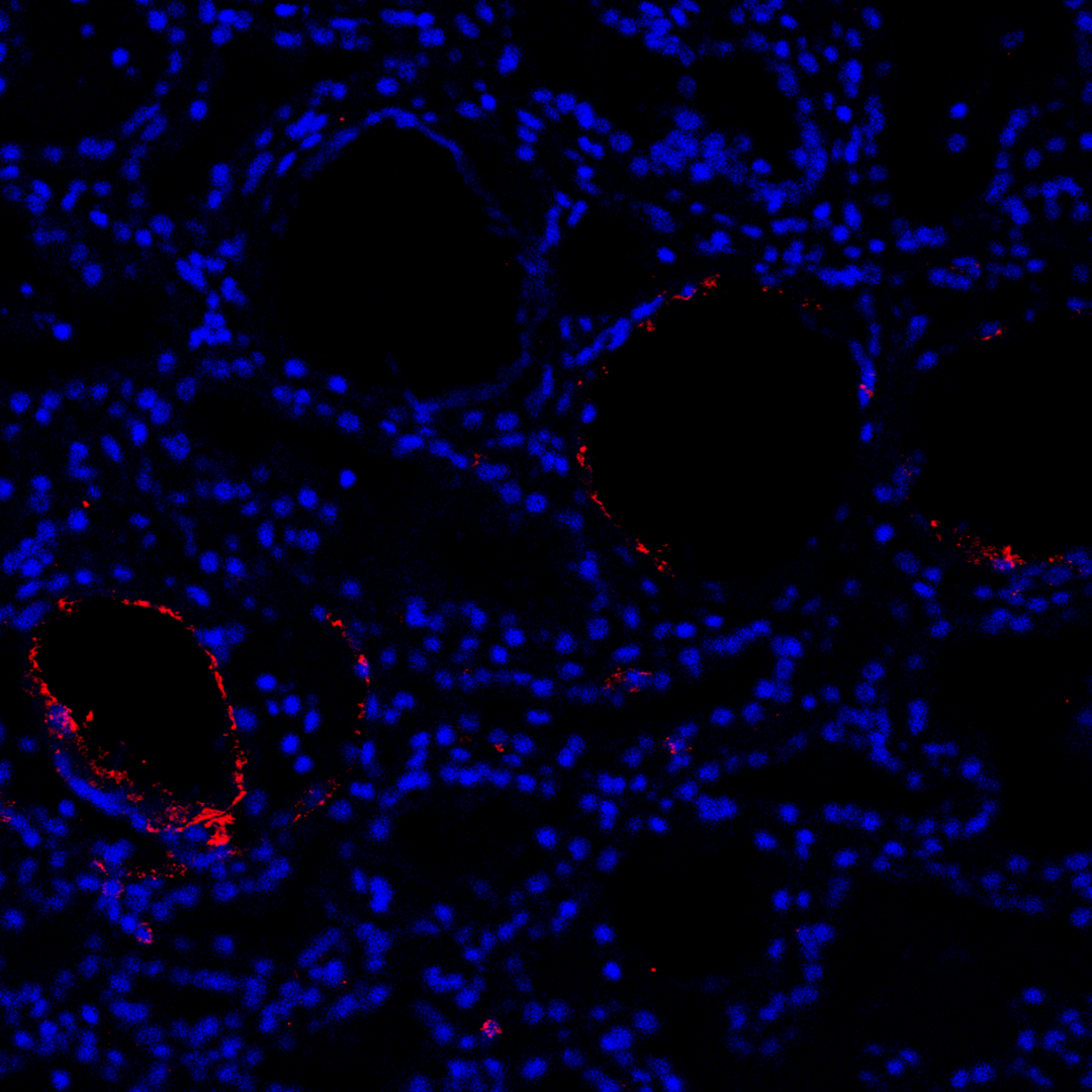

Supplement: Supplementary file 7 — Source data Fig. 3 [file 44319_2026_816_MOESM7_ESM.zip › Figure 3/3F/F-SLEX.tif]

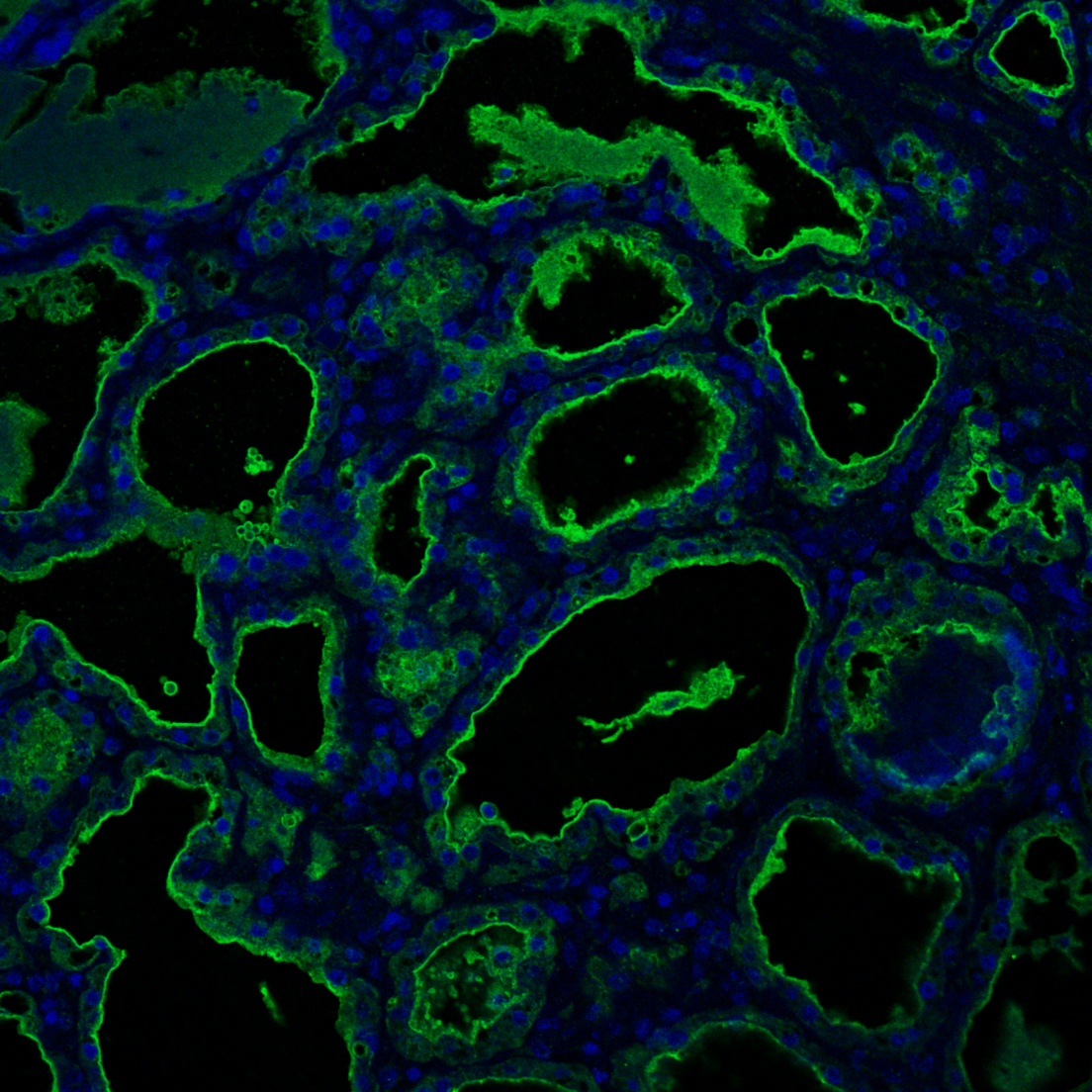

Supplement: Supplementary file 7 — Source data Fig. 3 [file 44319_2026_816_MOESM7_ESM.zip › Figure 3/3F/F-TXH5N1.tif]

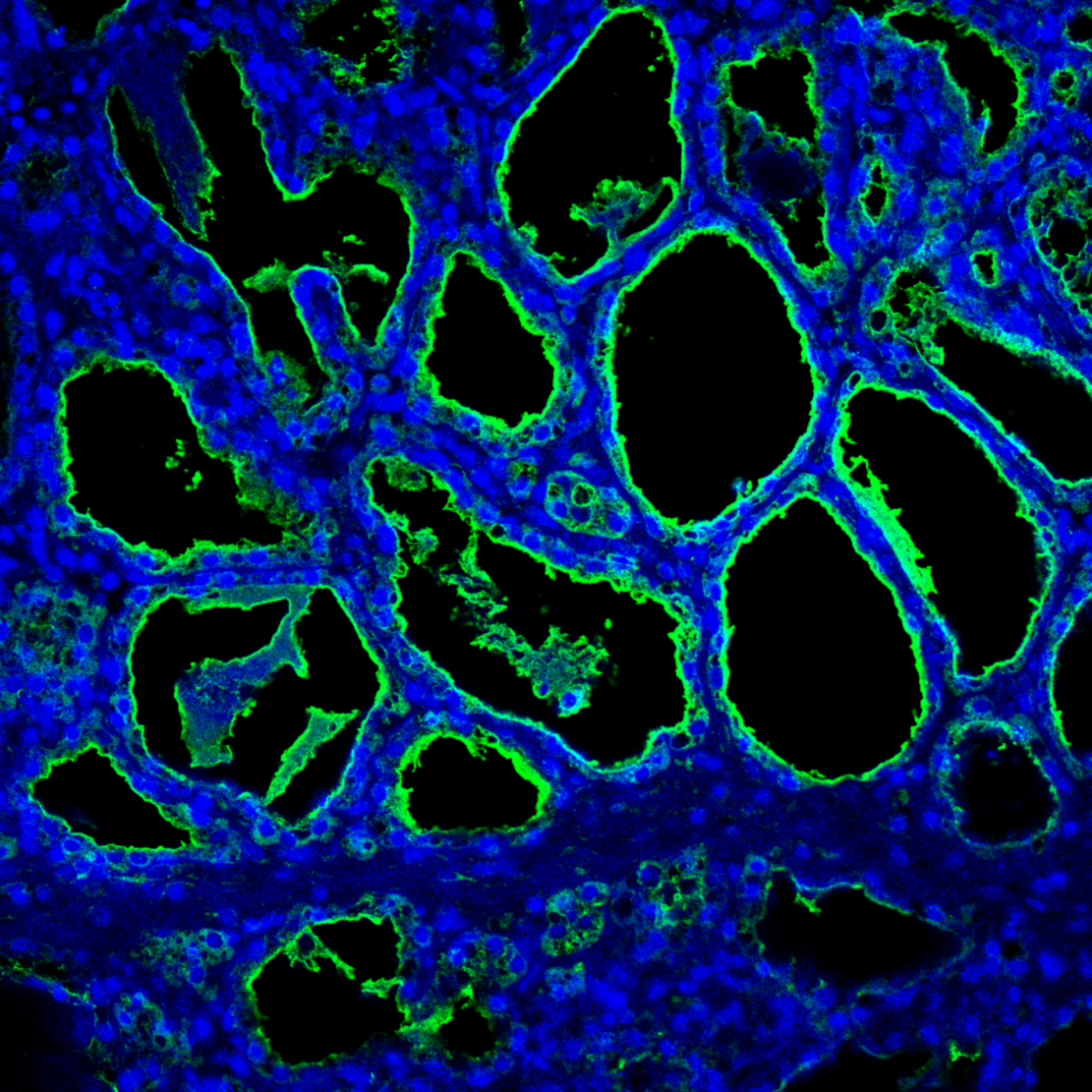

Supplement: Supplementary file 7 — Source data Fig. 3 [file 44319_2026_816_MOESM7_ESM.zip › Figure 3/3F/F-wsH5N8.tif]

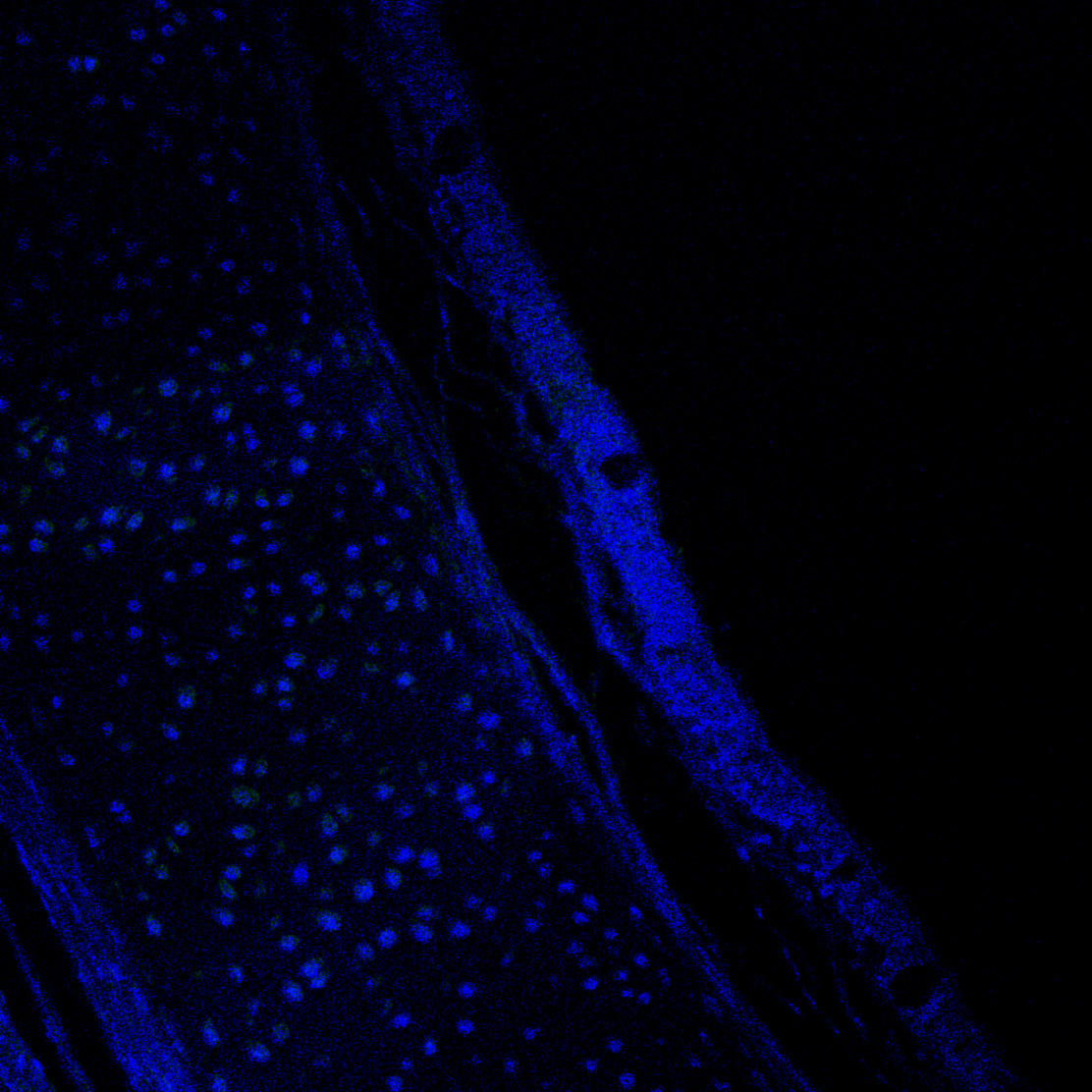

Supplement: Supplementary file 7 — Source data Fig. 3 [file 44319_2026_816_MOESM7_ESM.zip › Figure 3/3G/G-H1N1.tif]

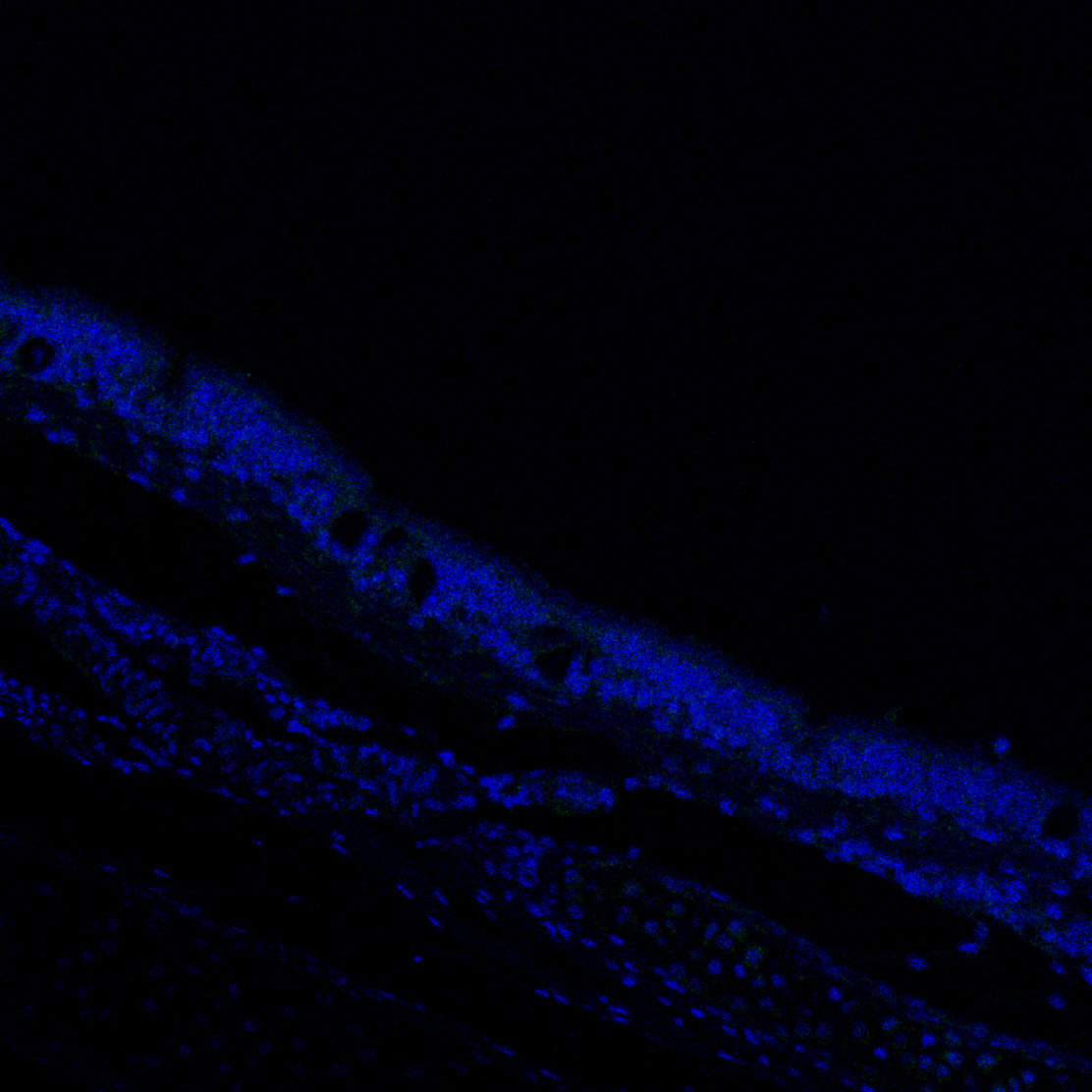

Supplement: Supplementary file 7 — Source data Fig. 3 [file 44319_2026_816_MOESM7_ESM.zip › Figure 3/3G/G-H3N2.tif]

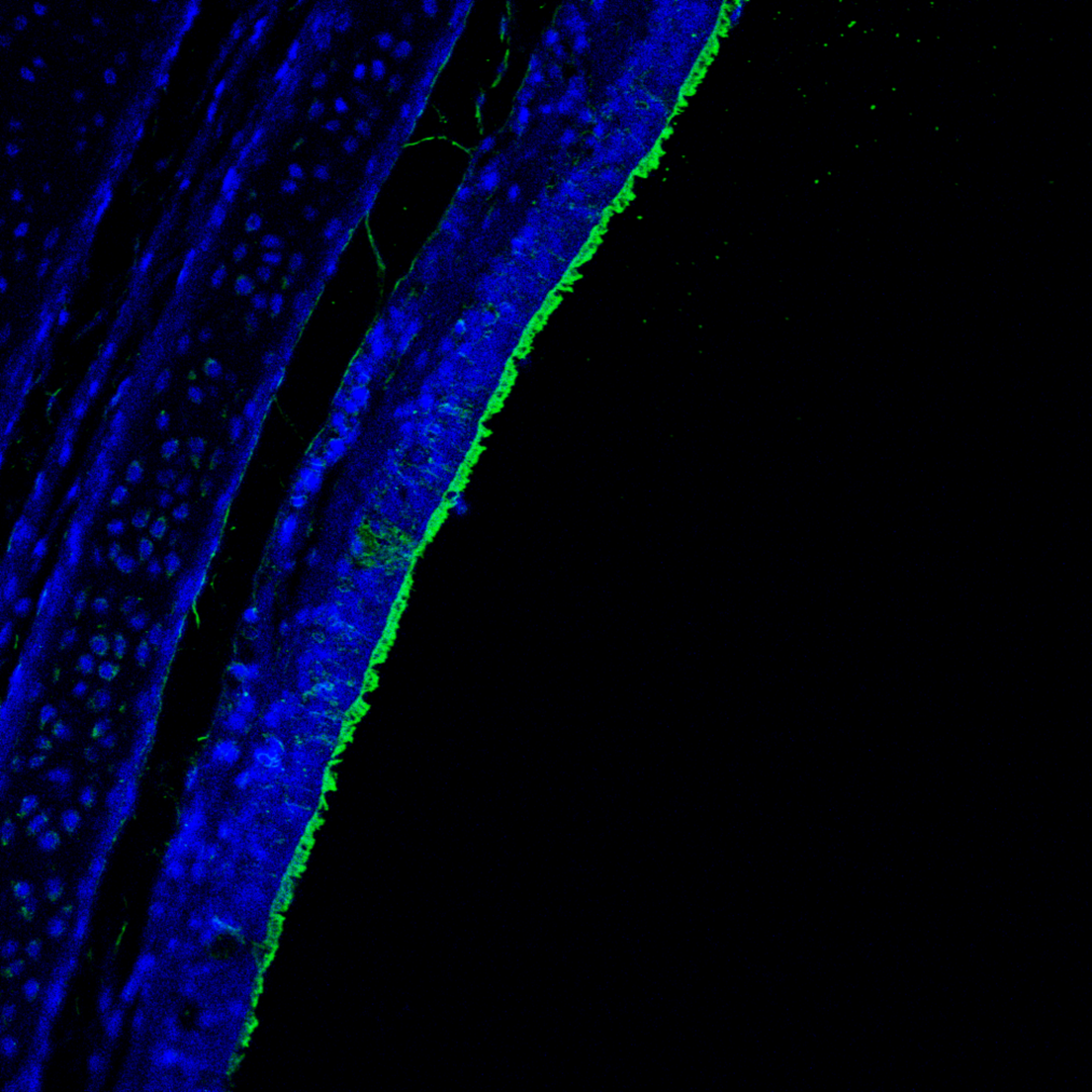

Supplement: Supplementary file 7 — Source data Fig. 3 [file 44319_2026_816_MOESM7_ESM.zip › Figure 3/3G/G-H5N8.tif]

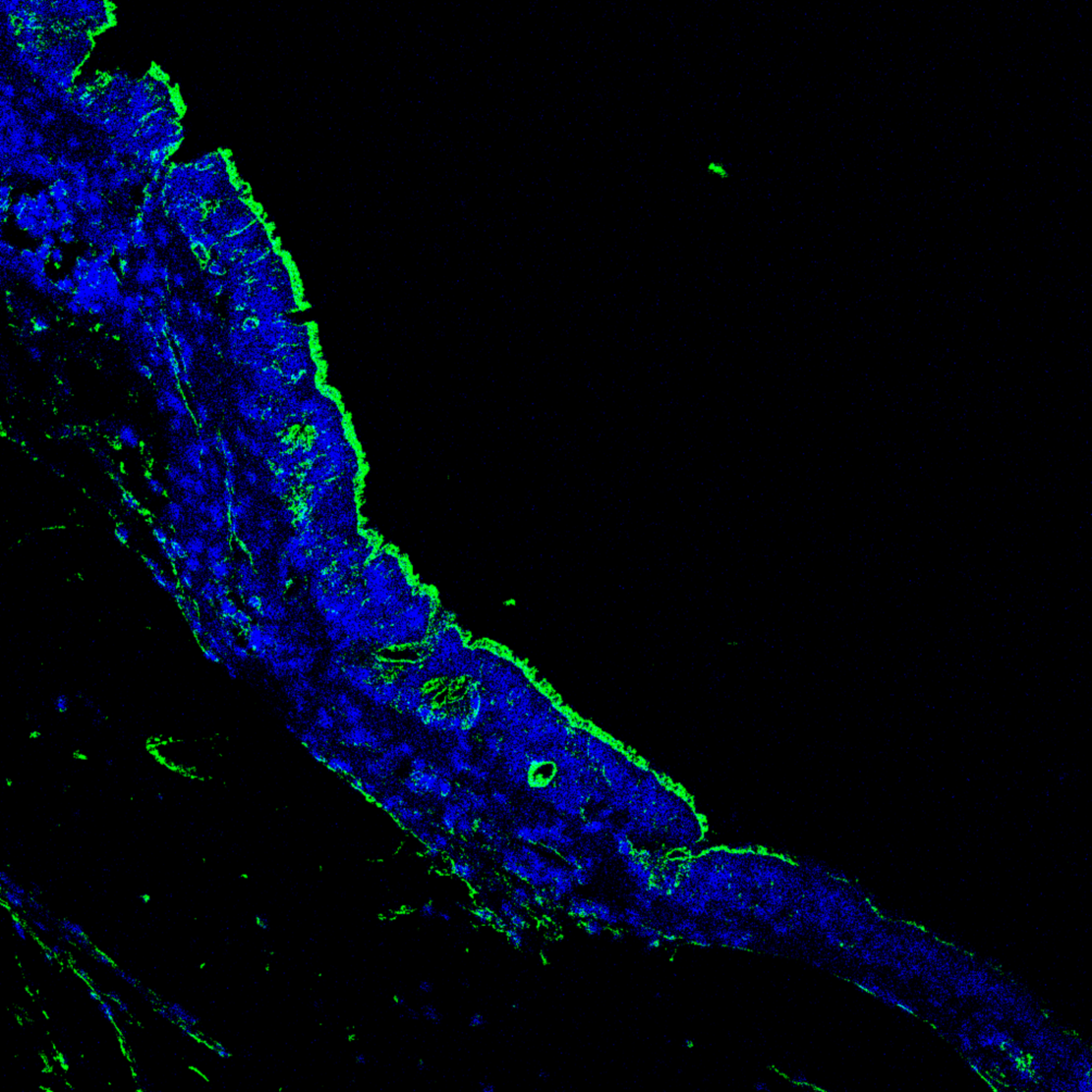

Supplement: Supplementary file 7 — Source data Fig. 3 [file 44319_2026_816_MOESM7_ESM.zip › Figure 3/3G/G-lnH5.tif]

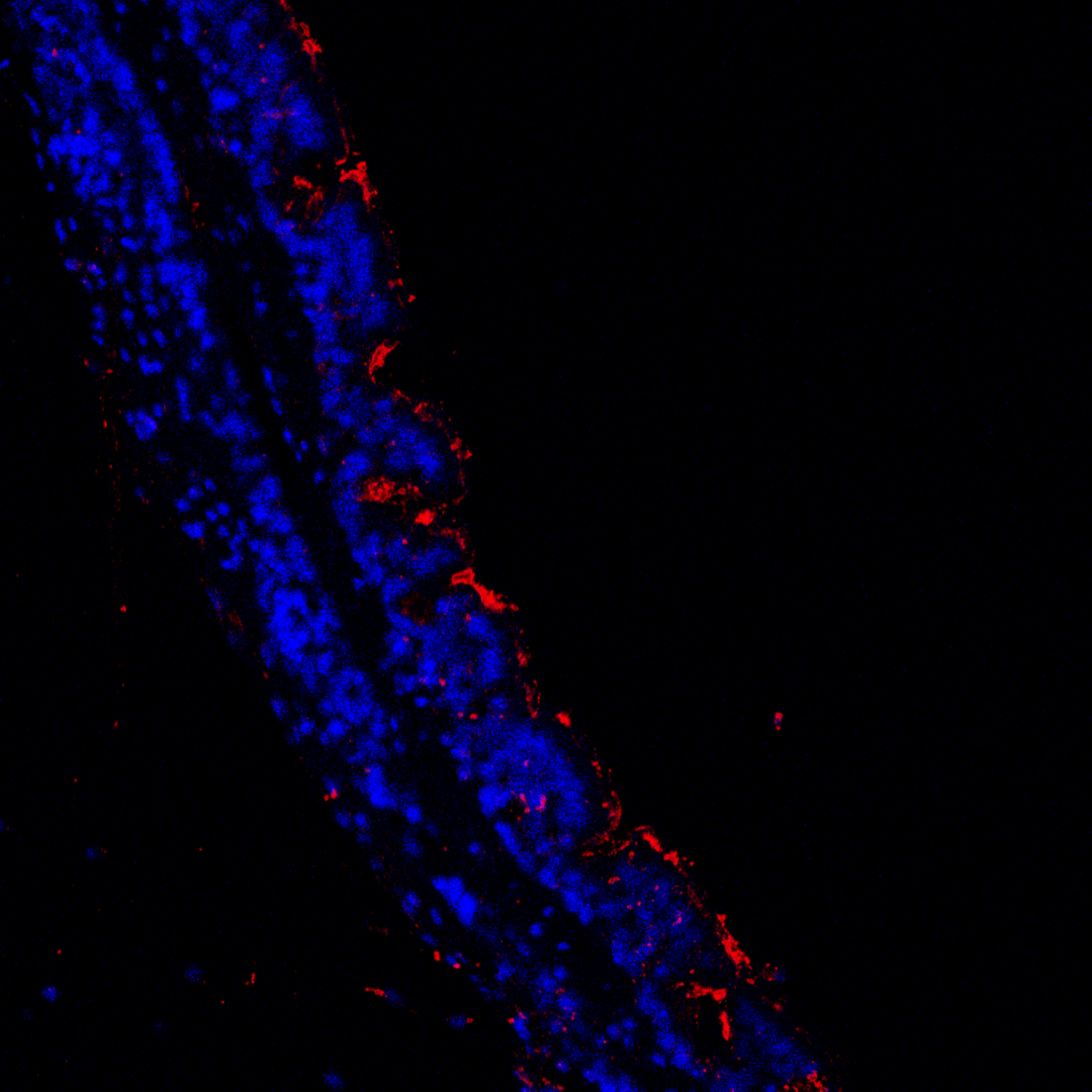

Supplement: Supplementary file 7 — Source data Fig. 3 [file 44319_2026_816_MOESM7_ESM.zip › Figure 3/3G/G-SLEX.tif]

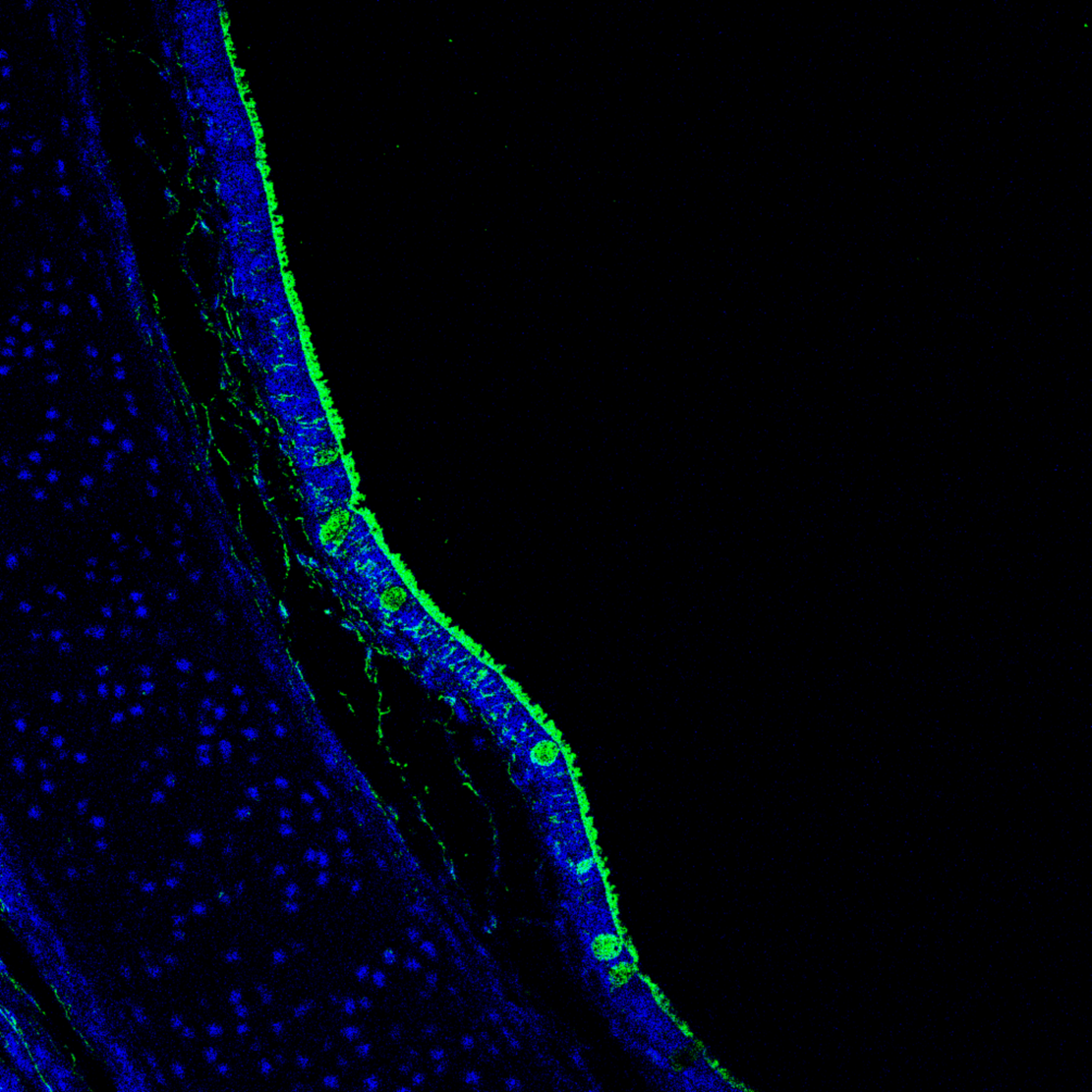

Supplement: Supplementary file 7 — Source data Fig. 3 [file 44319_2026_816_MOESM7_ESM.zip › Figure 3/3G/G-TxH5N1.tif]

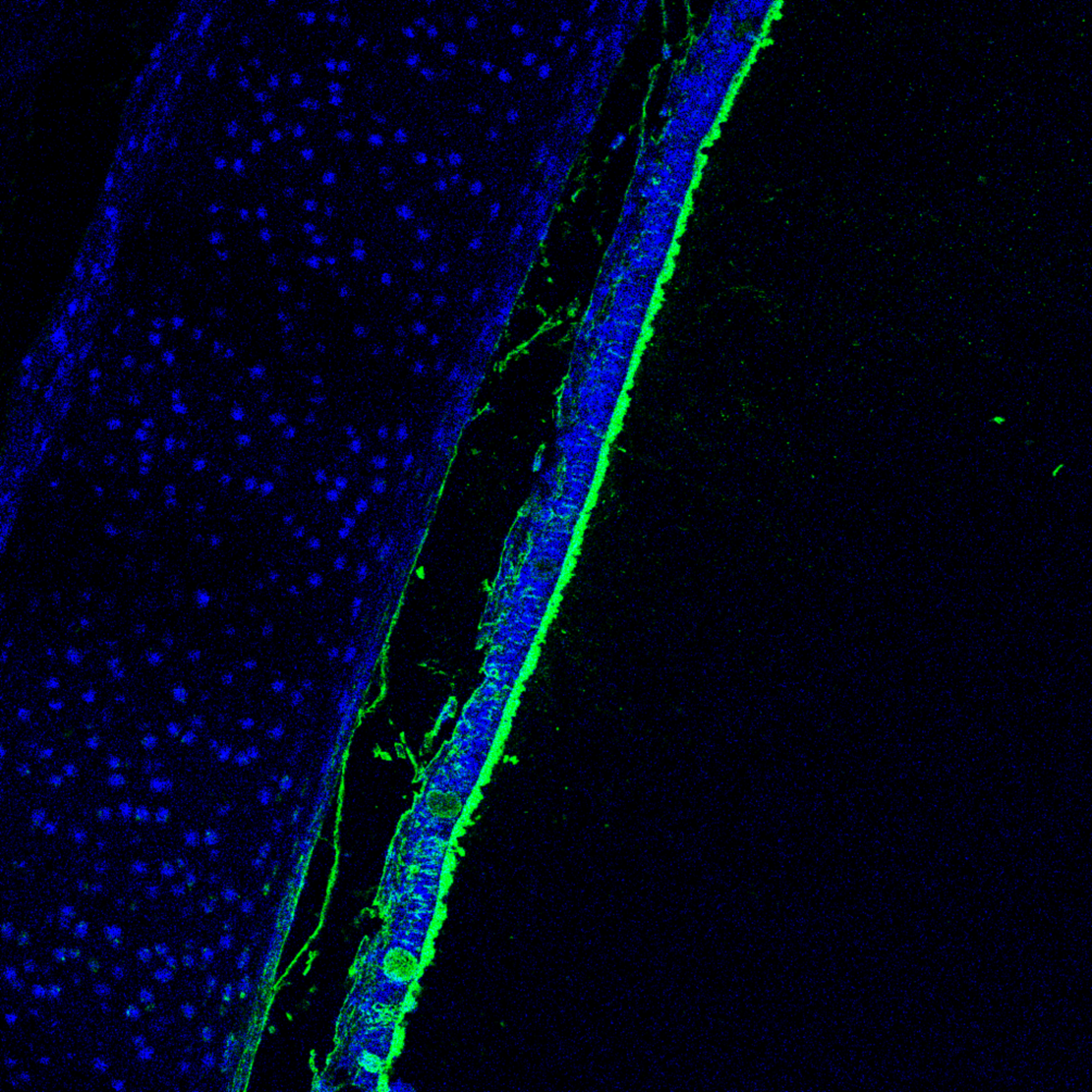

Supplement: Supplementary file 7 — Source data Fig. 3 [file 44319_2026_816_MOESM7_ESM.zip › Figure 3/3G/G-wsH5N8.tif]

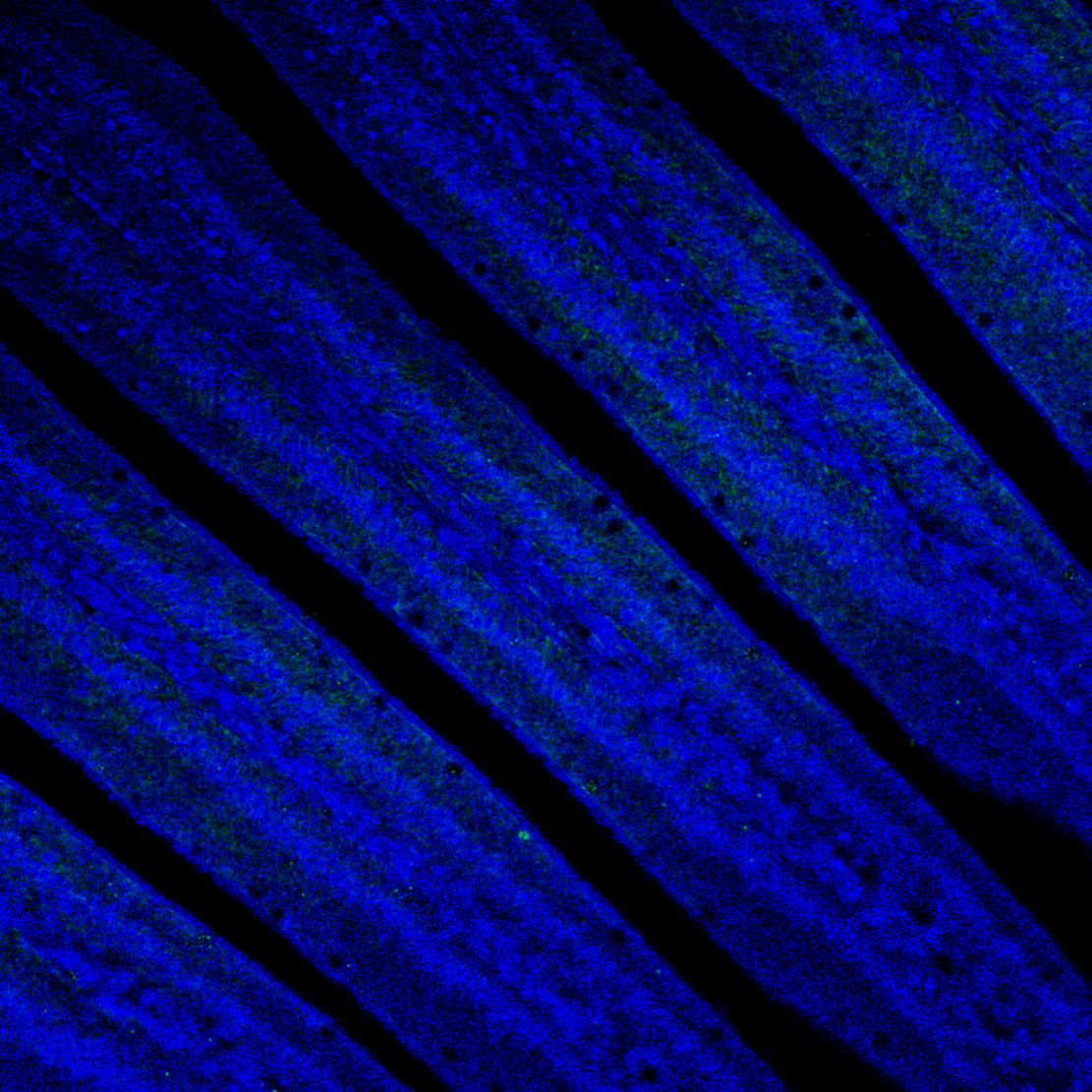

Supplement: Supplementary file 7 — Source data Fig. 3 [file 44319_2026_816_MOESM7_ESM.zip › Figure 3/3H/H-H1N1.tif]

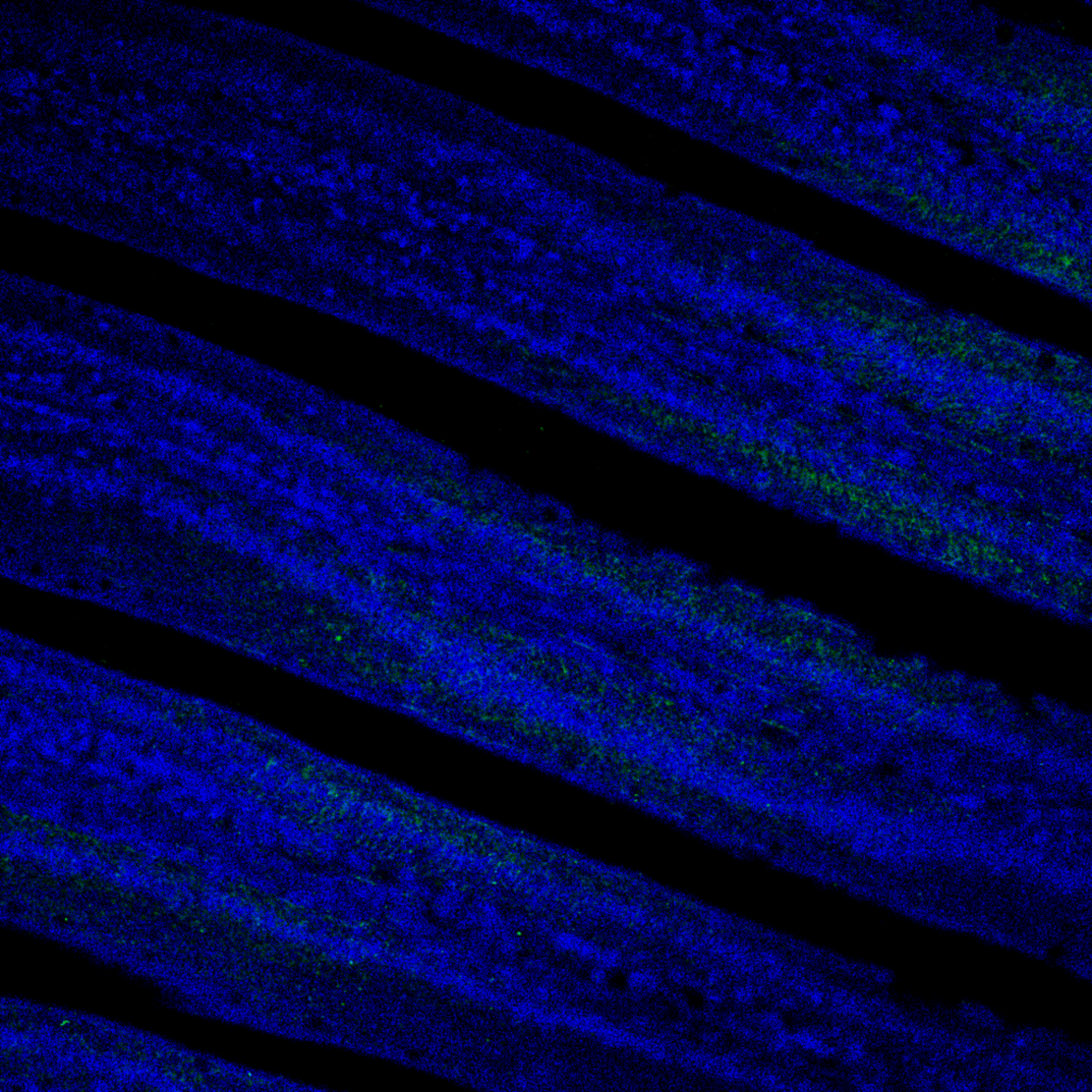

Supplement: Supplementary file 7 — Source data Fig. 3 [file 44319_2026_816_MOESM7_ESM.zip › Figure 3/3H/H-H3N2.tif]

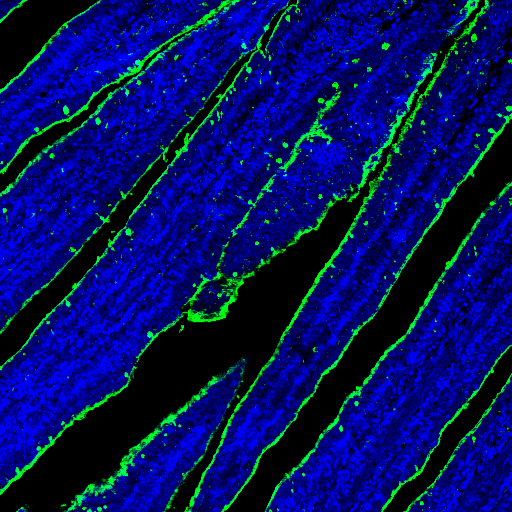

Supplement: Supplementary file 7 — Source data Fig. 3 [file 44319_2026_816_MOESM7_ESM.zip › Figure 3/3H/H-H5N8.tif]

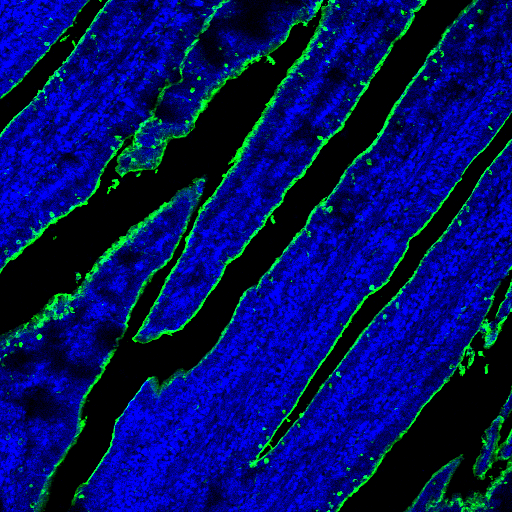

Supplement: Supplementary file 7 — Source data Fig. 3 [file 44319_2026_816_MOESM7_ESM.zip › Figure 3/3H/H-InH5.tif]

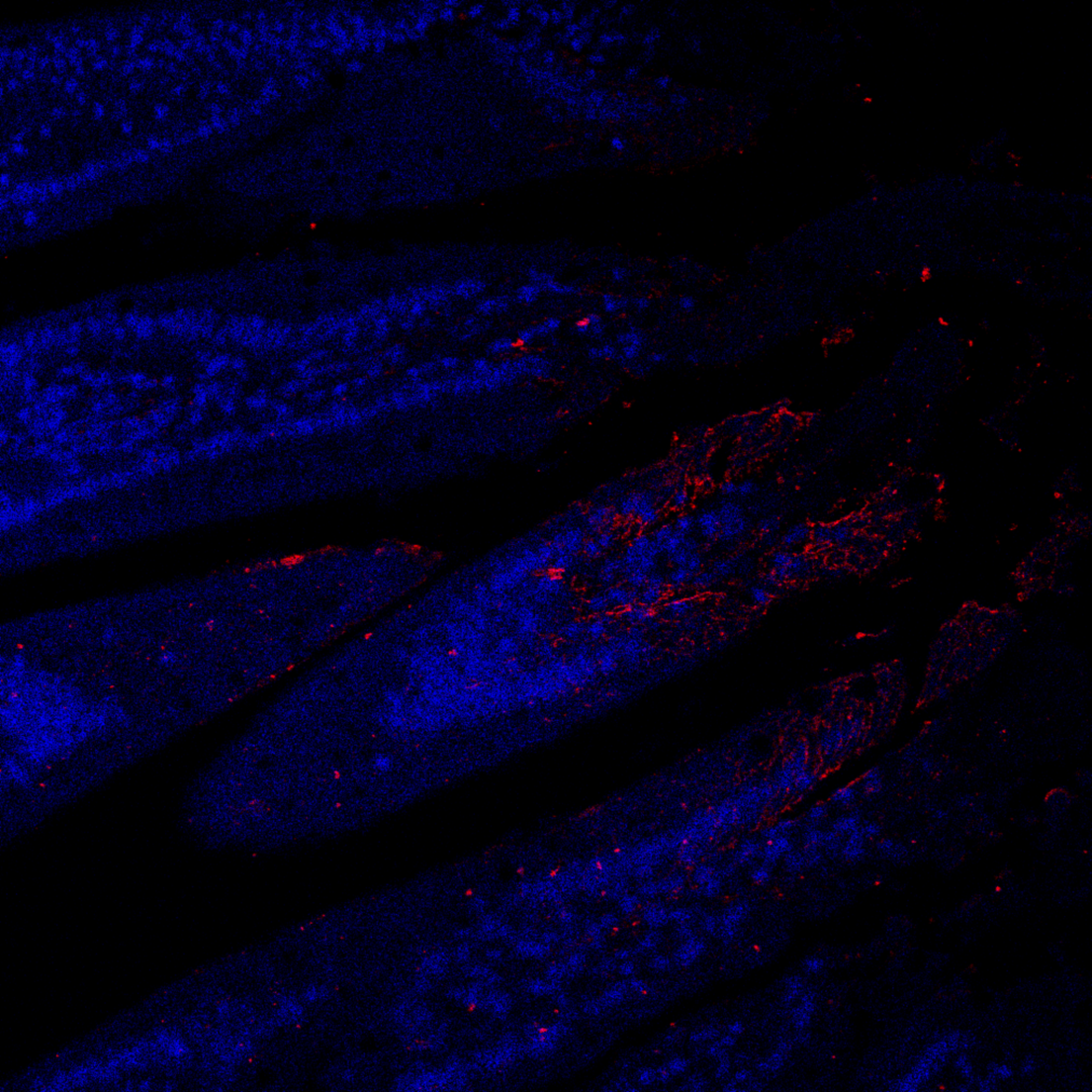

Supplement: Supplementary file 7 — Source data Fig. 3 [file 44319_2026_816_MOESM7_ESM.zip › Figure 3/3H/H-SLEX.tif]

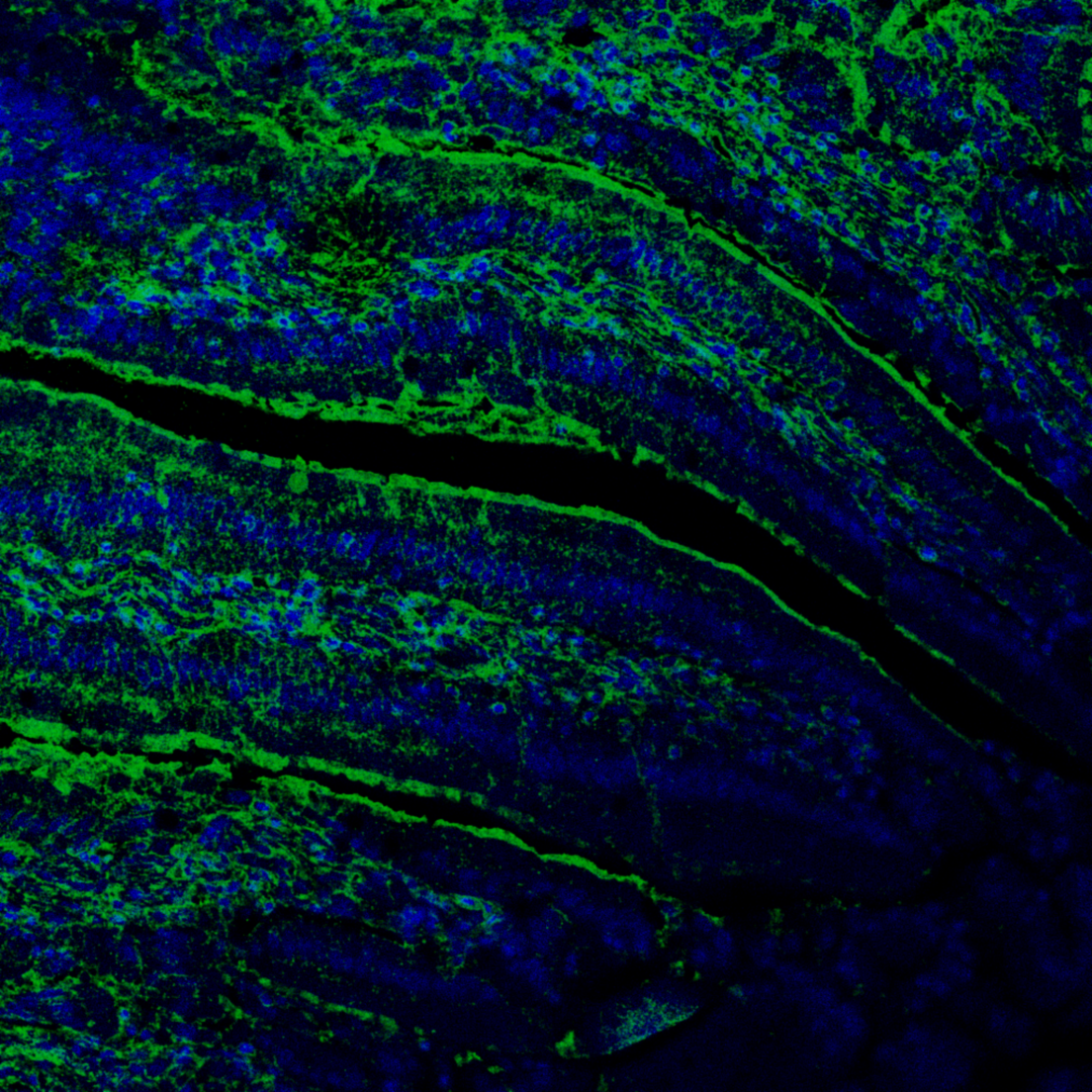

Supplement: Supplementary file 7 — Source data Fig. 3 [file 44319_2026_816_MOESM7_ESM.zip › Figure 3/3H/H-TxH5N1.tif]

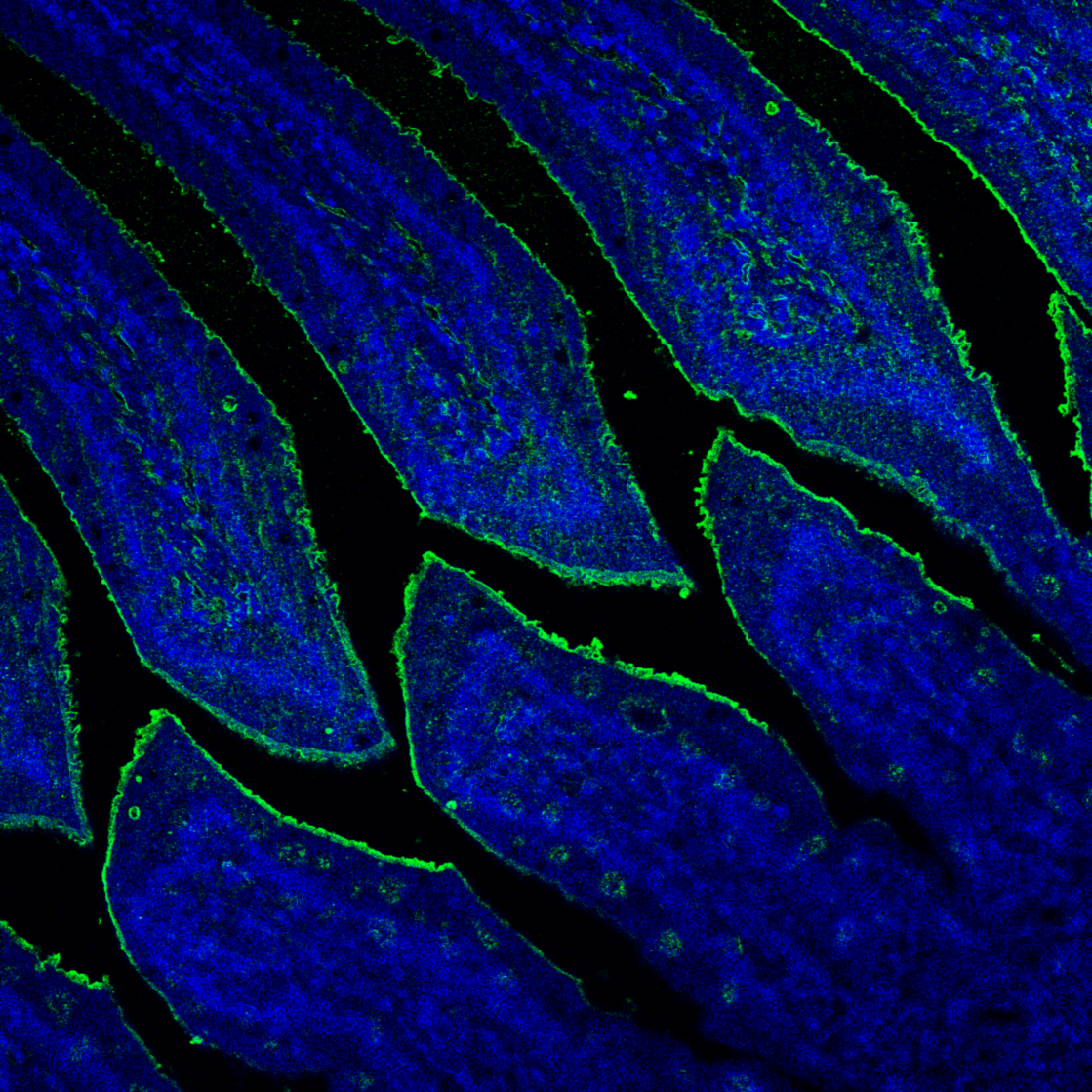

Supplement: Supplementary file 7 — Source data Fig. 3 [file 44319_2026_816_MOESM7_ESM.zip › Figure 3/3H/H-wsH5N8.tif]

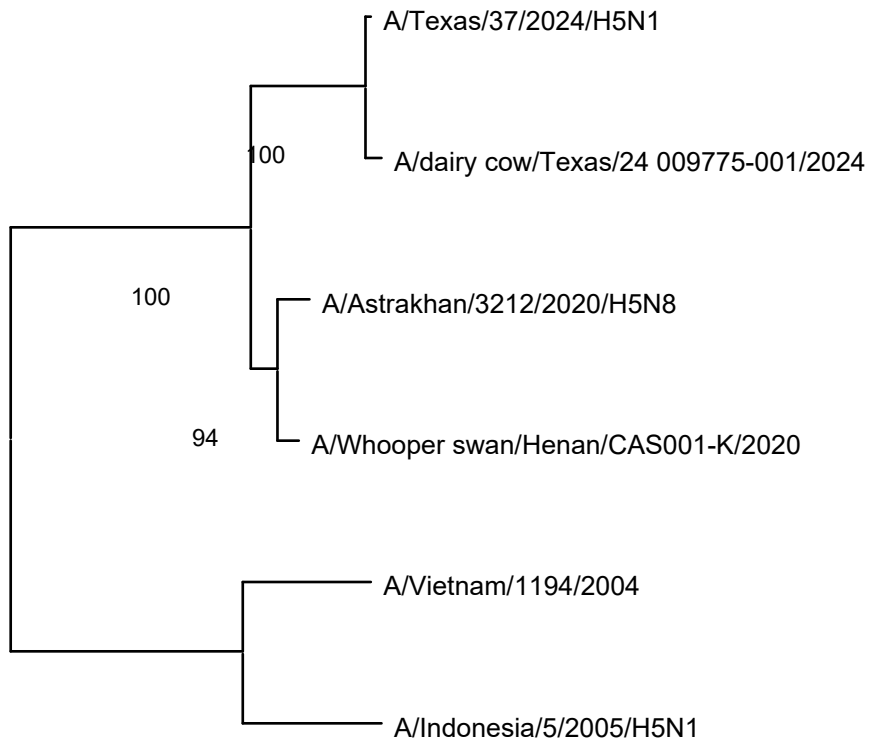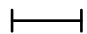

0.01

Supplement: Supplementary file 10 — Source data Fig. 6 [file 44319_2026_816_MOESM10_ESM.zip › Figure 6/6D/Phylogenetic tree.pdf]

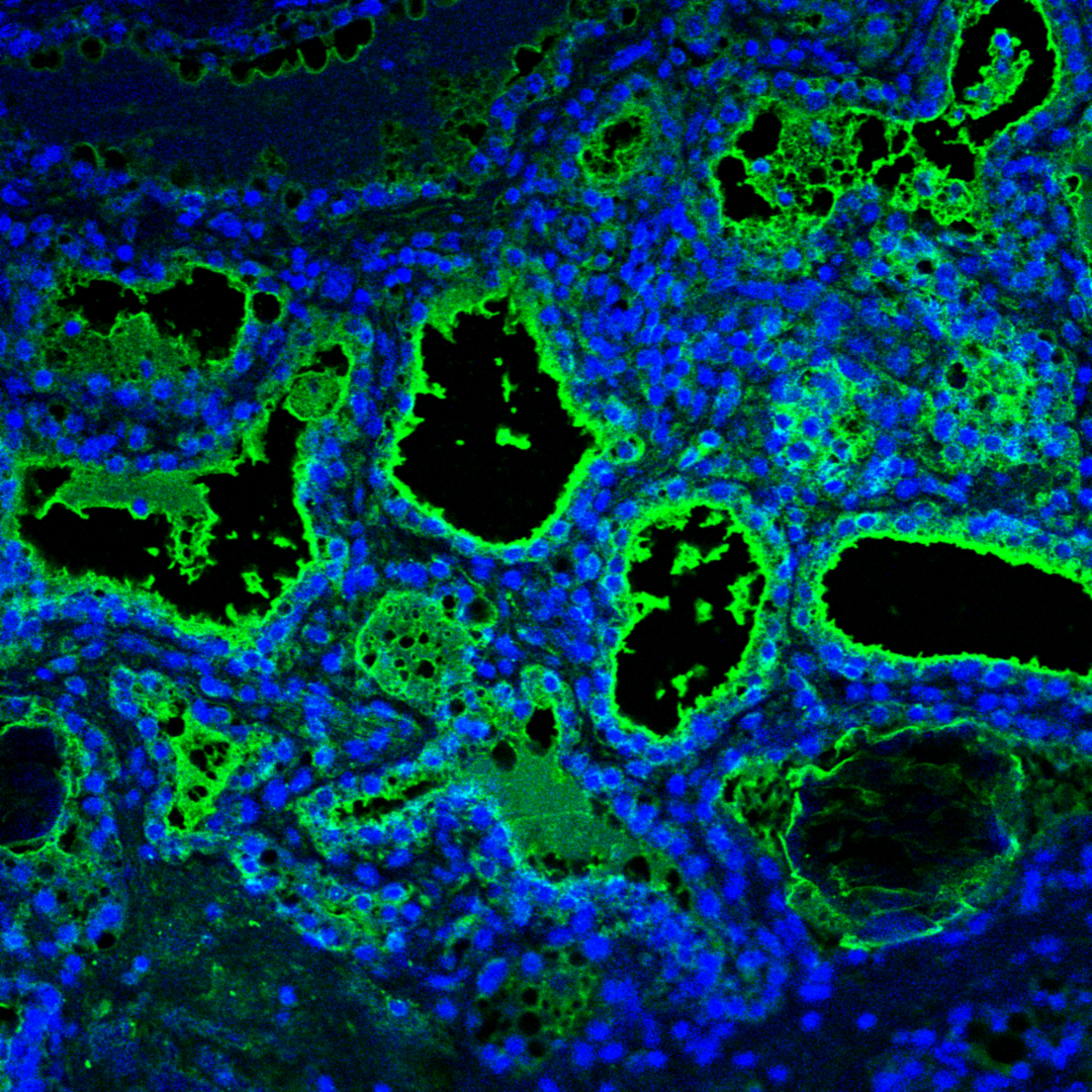

Supplement: Supplementary file 10 — Source data Fig. 6 [file 44319_2026_816_MOESM10_ESM.zip › Figure 6/6G/H5N8-DM bovine mammary gland.tif]

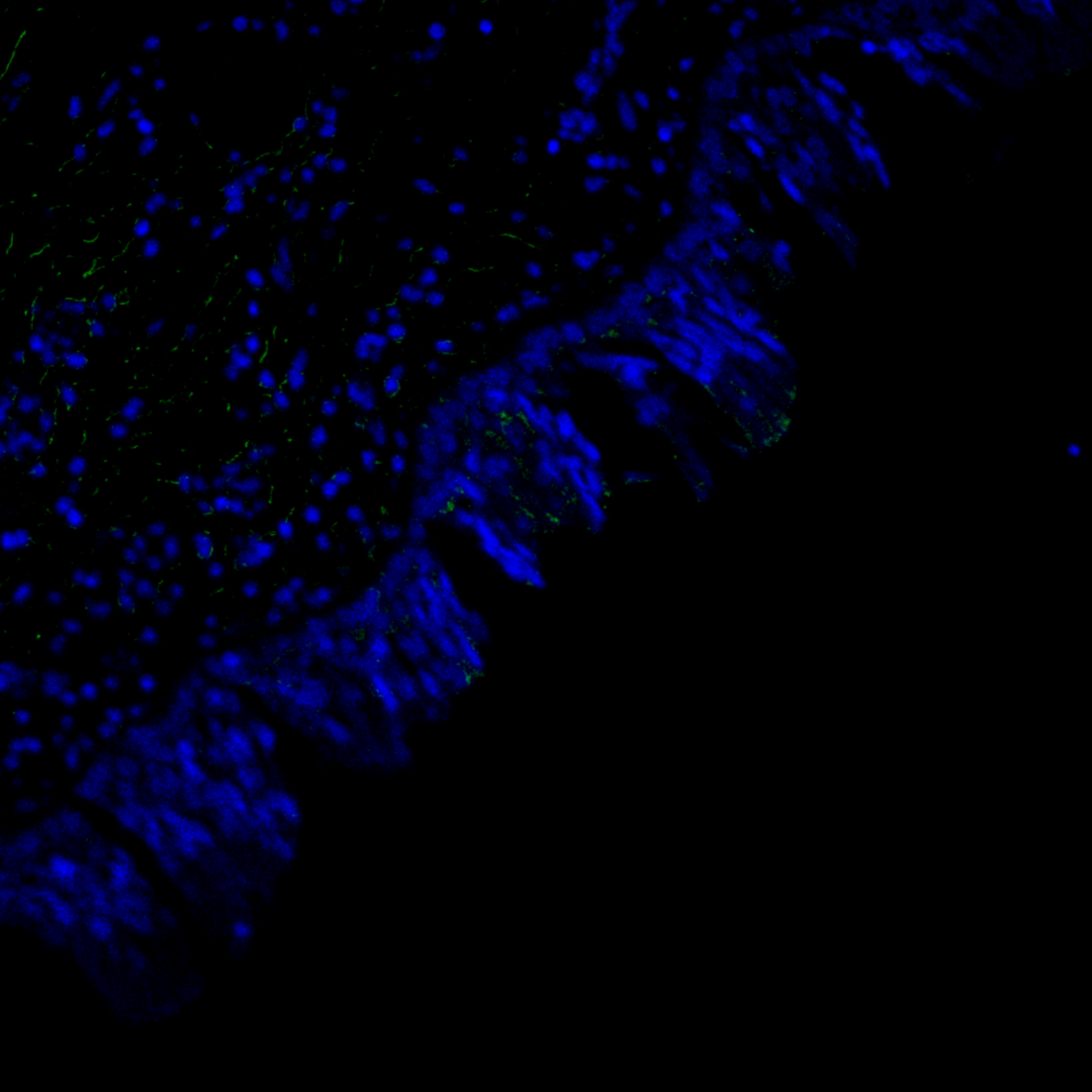

Supplement: Supplementary file 10 — Source data Fig. 6 [file 44319_2026_816_MOESM10_ESM.zip › Figure 6/6G/H5N8-DM bovine trachea.tif]

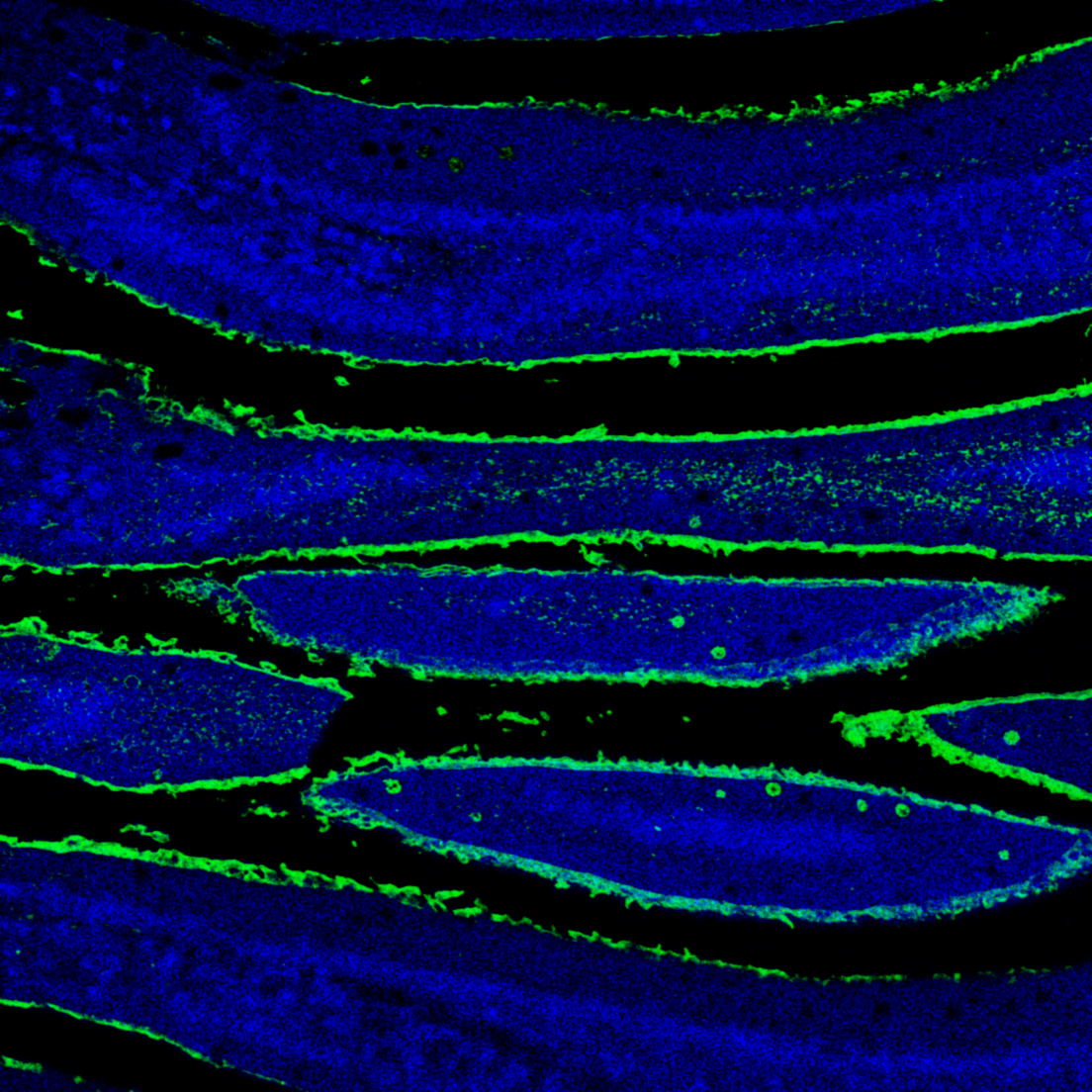

Supplement: Supplementary file 10 — Source data Fig. 6 [file 44319_2026_816_MOESM10_ESM.zip › Figure 6/6G/H5N8-DM duck intestine.tif]

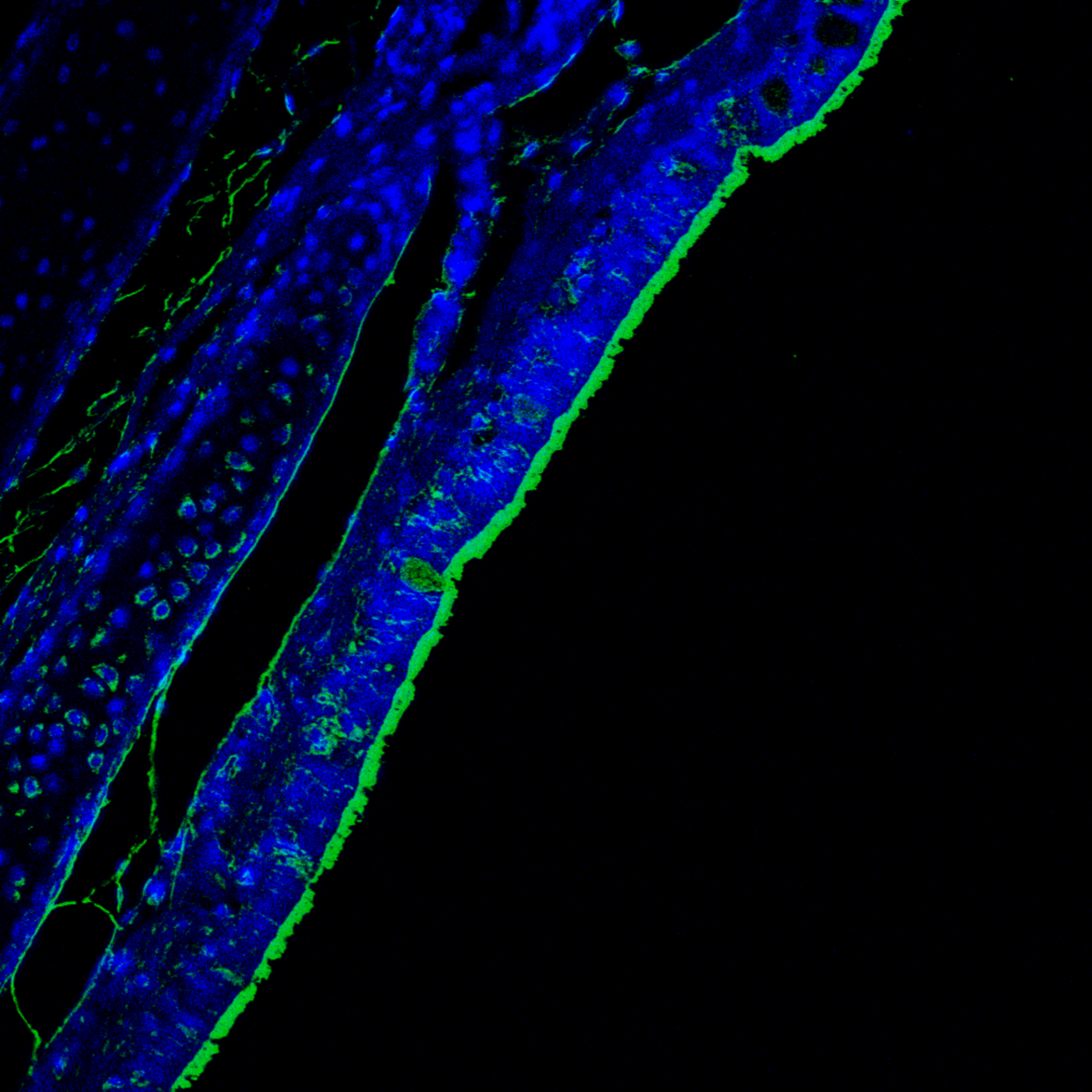

Supplement: Supplementary file 10 — Source data Fig. 6 [file 44319_2026_816_MOESM10_ESM.zip › Figure 6/6G/H5N8-DM duck trachea.tif]

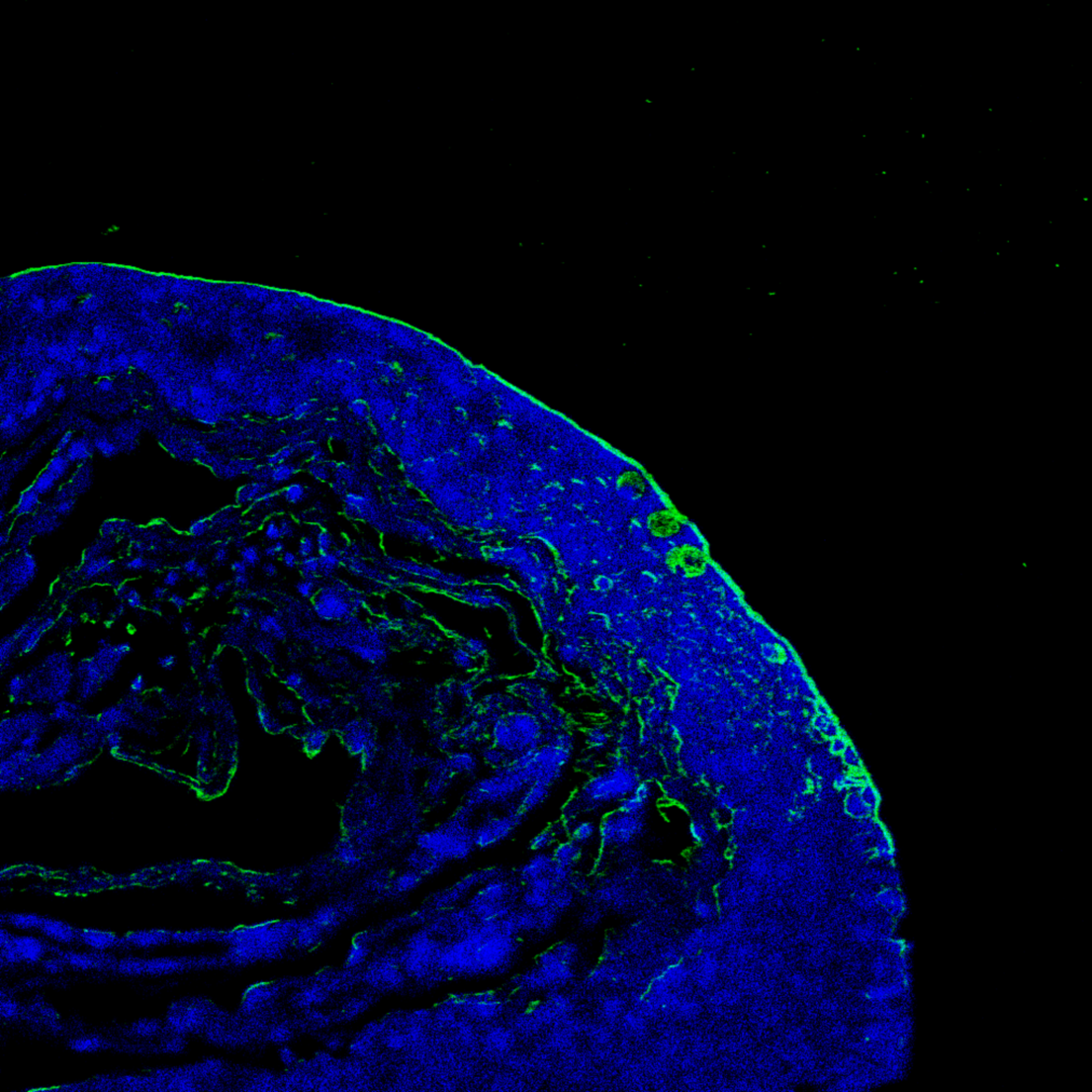

Supplement: Supplementary file 10 — Source data Fig. 6 [file 44319_2026_816_MOESM10_ESM.zip › Figure 6/6G/H5N8-DM human conjunctiva.tif]

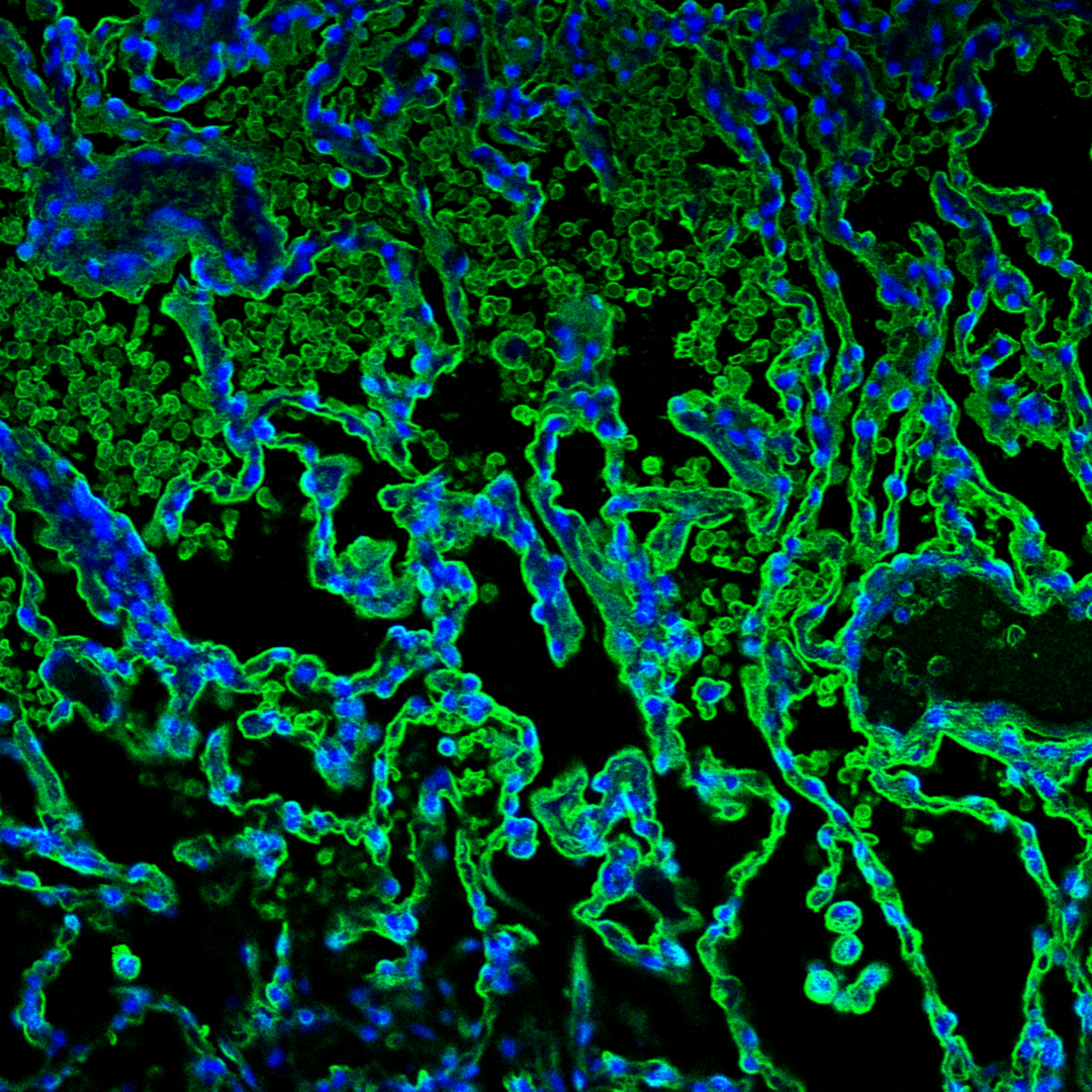

Supplement: Supplementary file 10 — Source data Fig. 6 [file 44319_2026_816_MOESM10_ESM.zip › Figure 6/6G/H5N8-DM human lung.tif]

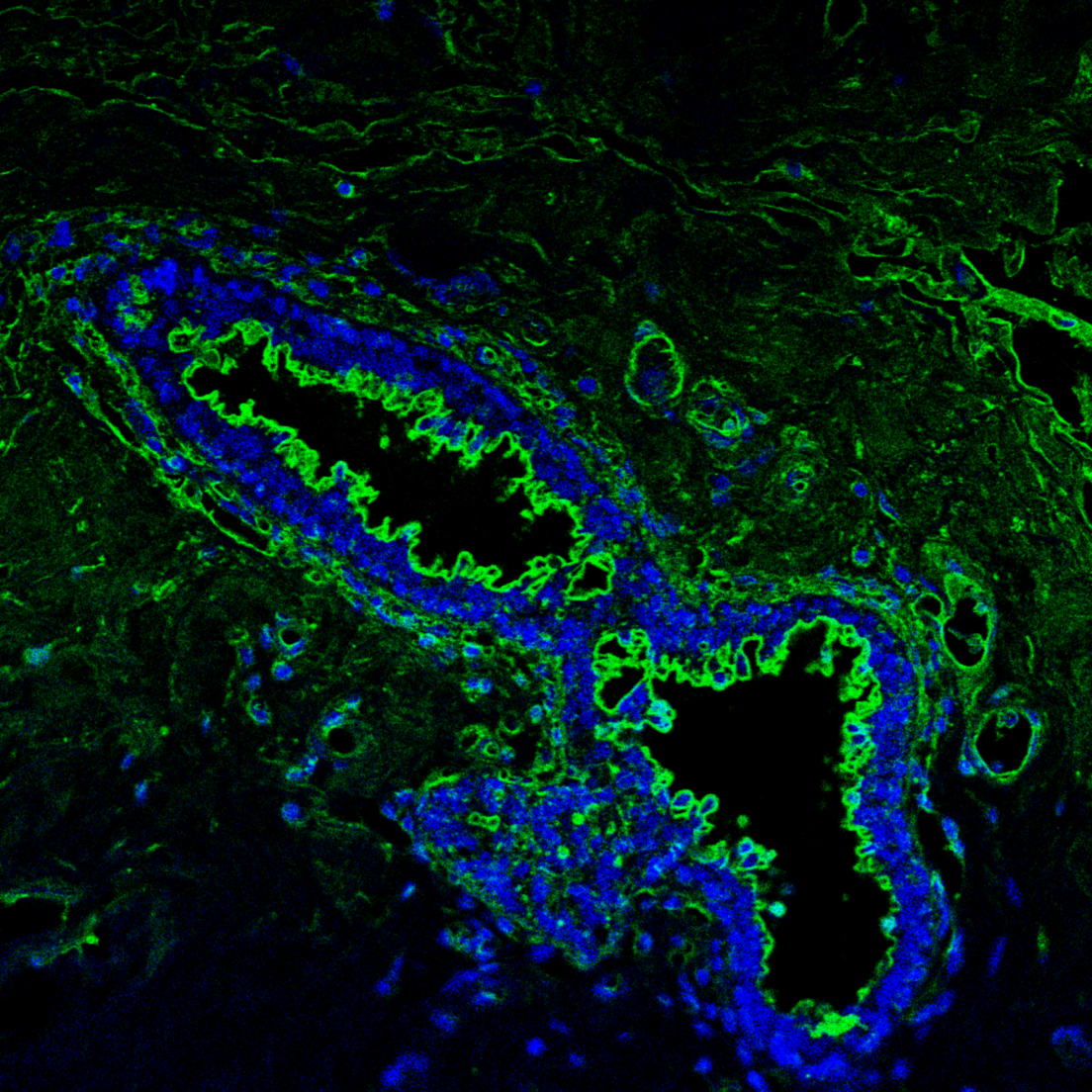

Supplement: Supplementary file 10 — Source data Fig. 6 [file 44319_2026_816_MOESM10_ESM.zip › Figure 6/6G/H5N8-DM human mammary gland.tif]

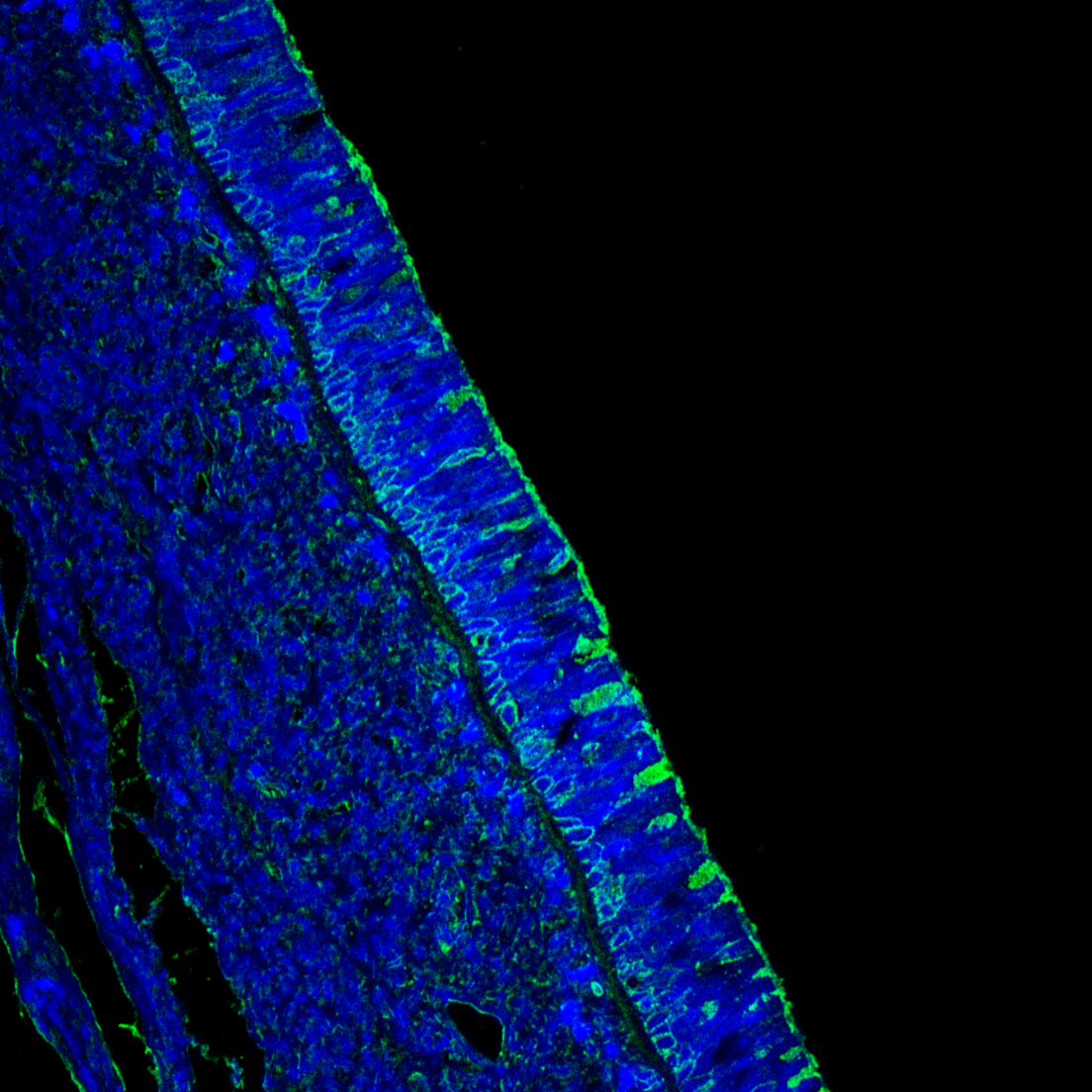

Supplement: Supplementary file 10 — Source data Fig. 6 [file 44319_2026_816_MOESM10_ESM.zip › Figure 6/6G/H5N8-DM human trachea.tif]
