# Supplementary material for: Hemagglutinin double-mutation enhances binding of human-infecting avian influenza virus clade 2.3.4.4b H5Ny to human and SLeX receptors
Source: EMBO Rep. 2026 Jun 16;27(14):4079–99. doi: 10.1038/s44319-026-00816-2 (PMC13400655; doi:10.1038/s44319-026-00816-2)
Supplement: Supplementary file 10 — Source data Fig. 6 [file 44319_2026_816_MOESM10_ESM.zip › Figure 6/6E/AA sequence of HA.docx]

>*A/Astrakhan/3212/20201212_H5N8*

MENIVLLLAIVSLVKSDQICIGYHANNSTEQVDTIMEKNVTVTHAQDILEKTHNGKLCDLNGVKPLILKDCSVAGWLLGNPMCDEFIRVPEWSYIVERANPANDLCYPGSLNDYEELKHLLSRINHFEKILIIPKSSWPNHETSLGVSAACPYQGAPSFFRNVVWLIKKNDAYPTIKISYNNTNREDLLILWGIHHSNNAEEQTNLYKNPTTYISVGTSTLNQRLVPKIATRSQVNGQRGRMDFFWTILKPDDAIHFESNGNFIAPEYAYKIVKKGDSTIMKSGVEYGHCNTKCQTPVGAINSSMPFHNIHPLTIGECPKYVKSNKLVLATGLRNSPLREKRRKRGLFGAIAGFIEGGWQGMVDGWYGYHHSNEQGSGYAADKESTQKAIDGVTNKVNSIIDKMNTQFEAVGREFNNLERRIENLNKKMEDGFLDVWTYNAELLVLMENERTLDFHDSNVKNLYDKVRLQLRDNAKELGNGCFEFYHKCDNECMESVRNGTYDYPQYSEEARLKREEISGVKLESIGTYQILSIYSTAASSLALAIMMAGLSLWMCSNGSLQCRICI

>A/*whooper_swan/Henan/CAS001-K/2020_H5N8*

VSLVKSDQICIGYHANNSTEQVDTIMEKNVTVTHAQDILEKTHNGKLCDLNGVKPLILKDCSVAGWLLGNPMCDEFIRVPEWSYIVERANPVNDLCYPGSLNDYEELKHLLSRINHFEKILIIPKSSWPNHETSLGVSAACPYQGAPSFFRNVVWLIKKNDAYPTIKISYNNTNREDLLILWGIHHSNNAEEQINLYKNPTTYISVGTSTLNQRLVPKIATRSQVNGQRGRMDFFWTILKPDDAIHFESNGNFIAPEYAYKIVKKGDSTIMKSGVEYGNCNTKCQTPVGAINSSMPFHNIHPLTIGECPKYVKSNKLVLATGLRNSPLREKRRKRGLFGAIAGFIEGGWQGMVDGWYGYHHSNEQGSGYAADKESTQKAIDGVTNKVNSIIDKMNTQFEAVGREFNNLERRIENLNKKMEDGFLDVWTYNAELLVLMENERTLDFHDSNVKNLYDKVRLQLRDNAKELGNGCFEFYHKCDNECMESVRNGTYDYPQYSEEARLKREEISGVK

>*A/Vietnam/1194/2004_H5N1*

LLFAIVSLVKSDQICIGYHANNSTEQVDTIMEKNVTVTHAQDILEKTHNGKLCDLDGVKPLILRDCSVAGWLLGNPMCDEFINVPEWSYIVEKANPVNDLCYPGDFNDYEELKHLLSRINHFEKIQIIPKSSWSSHEASLGVSSACPYQGKSSFFRNVVWLIKKNSTYPTIKRSYNNTNQEDLLVLWGIHHPKDAAEQTKLYQNPTTYISVGTSTLNQRLVPRIATRSKVNGQSGRMEFFWTILKPNDAINFESNGNFIAPEYAYKIVKKGDSTIMKSELEYGNCNTKCQTPMGAINSSMPFHNIHPLTIGECPKYVKSNRLVLATGLRNSPQRERRRKKR**↑**GLFGAIAGFIEGGWQGMVDGWYGYHHSNEQGSGYAADKESTQKAIDGVTNKVNSIIDKMNTQFEAVGREFNNLERRIENLNKKMEDGFLDVWTYNAELLVLMENERTLDFHDSNVKNLYDKVRLQLRDNAKELGNGCFEFYHKCDNECMESVRNGTYDYPQYSEEARLKREEISGVKLESIGIYQILSIYSTVASSLALAIMVAGLSLWMCSNGSLQCRICI

> *A/Indonesia/5/2005_H5N1*

MEKIVLLLAIVSLVKSDQICIGYHANNSTEQVDTIMEKNVTVTHAQDILEKTHNGKLCDLDGVKPLILRDCSVAGWLLGNPMCDEFINVPEWSYIVEKANPTNDLCYPGSFNDYEELKHLLSRINHFEKIQIIPKSSWSDHEASSGVSSACPYLGSPSFFRNVVWLIKKNSTYPTIKKSYNNTNQEDLLVLWGIHHPNDAAEQTRLYQNPTTYISIGTSTLNQRLVPKIATRSKVNGQSGRMEFFWTILKPNDAINFESNGNFIAPEYAYKIVKKGDSAIMKSELEYGNCNTKCQTPMGAINSSMPFHNIHPLTIGECPKYVKSNRLVLATGLRNSPQRESRRKKR**↑**GLFGAIAGFIEGGWQGMVDGWYGYHHSNEQGSGYAADKESTQKAIDGVTNKVNSIIDKMNTQFEAVGREFNNLERRIENLNKKMEDGFLDVWTYNAELLVLMENERTLDFHDSNVKNLYDKVRLQLRDNAKELGNGCFEFYHKCDNECMESIRNGTYNYPQYSEEARLKREEISGVKLESIGTYQILSIYSTVASSLALAIMMAGLSLWMCSNGSLQCRICI

>A/Texas/37/2024_H5N1

MENIVLLLAIVSLVKSDQICIGYHANNSTEQVDTIMEKNVTVTHAQDILEKTHNGKLCDLNGVKPLILKDCSVAGWLLGNPMCDEFIRVPEWSYIVERANPANDLCYPGSLNDYEELKHMLSRINHFEKIQIIPKSSWPNHETSLGVSAACPYQGAPSFFRNVVWLIKKNDAYPTIKISYNNTNREDLLILWGIHHSNNAEEQTNLYKNPITYISVGTSTLNQRLAPKIATRSQVNGQRGRMDFFWTILKPDDAIHFESNGNFIAPEYAYKIVKKGDSTIMKSGVEYGHCNTKCQTPVGAINSSMPFHNIHPLTIGECPKYVKSNKLVLATGLRNSPLREKRRKRGLFGAXAGFIEGGWQGMVDGWYGYHHSNEQGSGYAADKESTQKAIDGVTNKVNSIIDKMNTQFEAVGREFNNLERRIENLNKKMEDGFLDVWTYNAELLVLMENERTLDFHDSNVKNLYDKVRLQLRDNAKELGNGCFEFYHKCDNECMESVRNGTYDYPQYSEEARLKREEISGVKLESVGTYQILSIYSTAASSLALAIMMAGLSLWMCSNGSLQCRICI

>A/dairy cow/Texas/24_009775-001/2024_H5N1

MENIVLLLAIVSLVKSDQICIGYHANNSTEQVDTIMEKNVTVTHAQDILEKTHNGKLCDLNGVKPLILKDCSVAGWLLGNPMCDEFIRVPEWSYIVERANPANDLCYPGSLNDYEELKHMLSRINHFEKIQIIPKSSWPNHETSLGVSAACPYQGAPSFFRNVVWLIKKNDAYPTIKISYNNTNREDLLILWGIHHSNNAEEQTNLYKNPITYISVGTSTLNQRLAPKIATRSQVNGQRGRMDFFWTILKPDDAIHFESNGNFIAPEYAYKIVKKGDSTIMKSGVEYGHCNTKCQTPVGAINSSMPFHNIHPLTIGECPKYVKSNKLVLATGLRNSPLREKRRKRGLFGAIAGFIEGGWQGMVDGWYGYHHSNEQGSGYAADKESTQKAIDGVTNKVNSIIDKMNTQFEAVGREFNNLERRIENLNKKMEDGFLDVWTYNAELLVLMENERTLDFHDSNVKNLYDKVRLQLRDNAKELGNGCFEFYHKCDNECMESVRNGTYDYPQYSEEARLKREEISGVKLESVGTYQILSIYSTAASSLALAIMMAGLSLWMCSNGSLQCRICI
