# Supplementary material for: Hemagglutinin double-mutation enhances binding of human-infecting avian influenza virus clade 2.3.4.4b H5Ny to human and SLeX receptors
Source: EMBO Rep. 2026 Jun 16;27(14):4079–99. doi: 10.1038/s44319-026-00816-2 (PMC13400655; doi:10.1038/s44319-026-00816-2)
Supplement: Supplementary file 11 — Expanded View Figures [file 44319_2026_816_MOESM11_ESM.pdf]

Expanded View Figures

				SP													130-loop						150-loop	
Clade	Year	H5Ny Strain	Host	-4	2	46	55	62	81	93	101	102	111	119	127	128	131	133	137	142	144	145	159	160
1	2004	A/VietNam/1203/2004/H5N1	human	K	F	K	D	R	N	V	D	F	L	Q	S	S	A	L	S	Q	K	S	S	T
2.1.3.2	2005	A/Indonesia/5/2005/H5N1	human	K	L	T	D	R	N	T	S	F	L	Q	S	D	A	S	S	L	S	S	S	T
2.3.4.4b	2020	A/whooper_swan/Henan/CAS001-G/2020/H5N8	swan	N	L	T	N	K	R	V	S	L	L	L	P	N	T	L	A	Q	A	P	D	A
2.3.4.4b	2020	A/Astrakhan/3212/2020/H5N8	human	N	L	T	N	K	R	A	S	L	L	L	P	N	T	L	A	Q	A	P	D	A
2.3.4.4b	2024	A/Texas/37/2024/H5N1	human	N	L	T	N	K	R	A	S	L	M	Q	P	N	T	L	A	Q	A	P	D	A

							190-helix							220-loop								
Clade	Year	H5Ny Strain	Host	166	173	185	187	189	192	193	196	199	204	214	222	226	227	228	231	240	244	266	268	271
1	2004	A/VietNam/1203/2004/H5N1	human	R	Q	P	D	A	T	K	Q	T	V	V	K	Q	S	G	E	N	N	T	M	E
2.1.3.2	2005	A/Indonesia/5/2005/H5N1	human	K	Q	P	D	A	T	R	Q	T	I	V	K	Q	S	G	E	N	N	A	M	E
2.3.4.4b	2020	A/whooper_swan/Henan/CAS001-G/2020/H5N8	swan	I	R	S	N	E	I	N	K	T	V	V	Q	Q	R	G	D	D	H	T	M	G
2.3.4.4b	2020	A/Astrakhan/3212/2020/H5N8	human	I	R	S	N	E	T	N	K	T	V	V	Q	Q	R	G	D	D	H	T	M	G
2.3.4.4b	2024	A/Texas/37/2024/H5N1	human	I	R	S	N	E	T	N	K	I	V	A	Q	Q	R	G	D	D	H	T	M	G

													TM	
Clade	Year	H5Ny Strain	Host	276	285	313	325	328	481	487	510	512	522	532
1	2004	A/VietNam/1203/2004/H5N1	human	N	M	R	Q	R	V	D	I	I	V	V
2.1.3.2	2005	A/Indonesia/5/2005/H5N1	human	N	M	R	Q	S	I	N	I	T	V	M
2.3.4.4b	2020	A/whooper_swan/Henan/CAS001-G/2020/H5N8	swan	N	V	K	L	K	V	D	I	T	A	M
2.3.4.4b	2020	A/Astrakhan/3212/2020/H5N8	human	H	V	K	L	K	V	D	I	T	A	M
2.3.4.4b	2024	A/Texas/37/2024/H5N1	human	H	V	K	L	K	V	D	V	T	A	M

Figure EV1. Sequence alignment of huH5N8, wsH5N8, TxH5N1, and early H5Ny HAs.

Sequence alignment of huH5N8, wsH5N8, InH5, VN1203 and TxH5N1 HAs. Signal peptide region, secondary structural elements of the binding site (i.e., the 130-loop, 150-loop, 190-helix, and 220-loop), and transmembrane region are labeled.

© The Author(s)

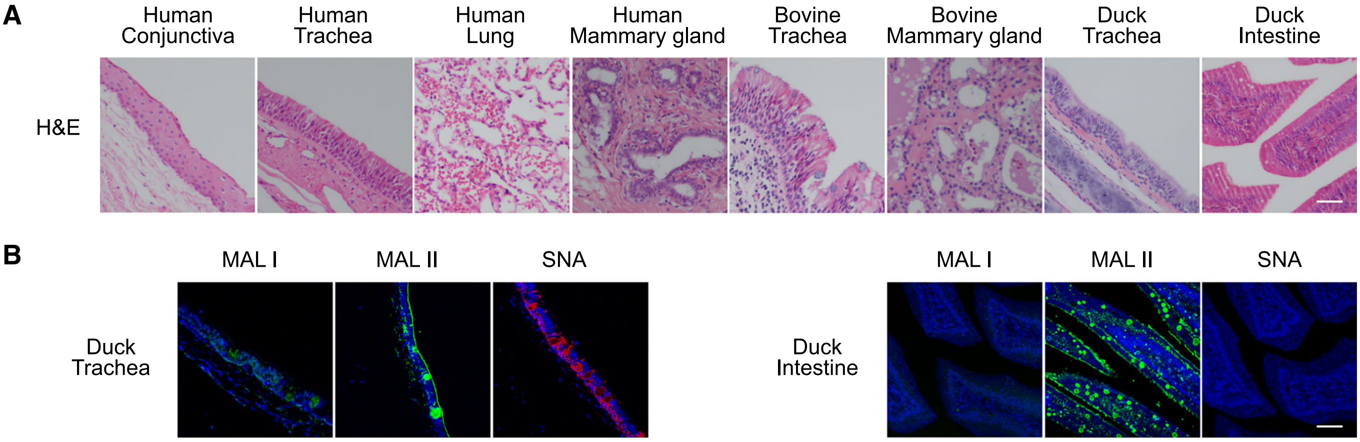

Figure EV2. Representative images of H&E staining and staining of duck tissue sections with lectin.

(**A**) Representative images of H&E staining. (**B**) Staining of duck tissue sections with lectin. MAL-I, MAL-II, and SNA staining of duck trachea and intestine tissue sections. Specific staining areas are green for MAL-I or MAL-II staining and red for SNA staining. Scale bar: 50 µm. MAL Maackia amurensis lectin, SNA Sambucus nigra lectin. Representative results from at least two independent experiments are shown.

 © The Author(s)

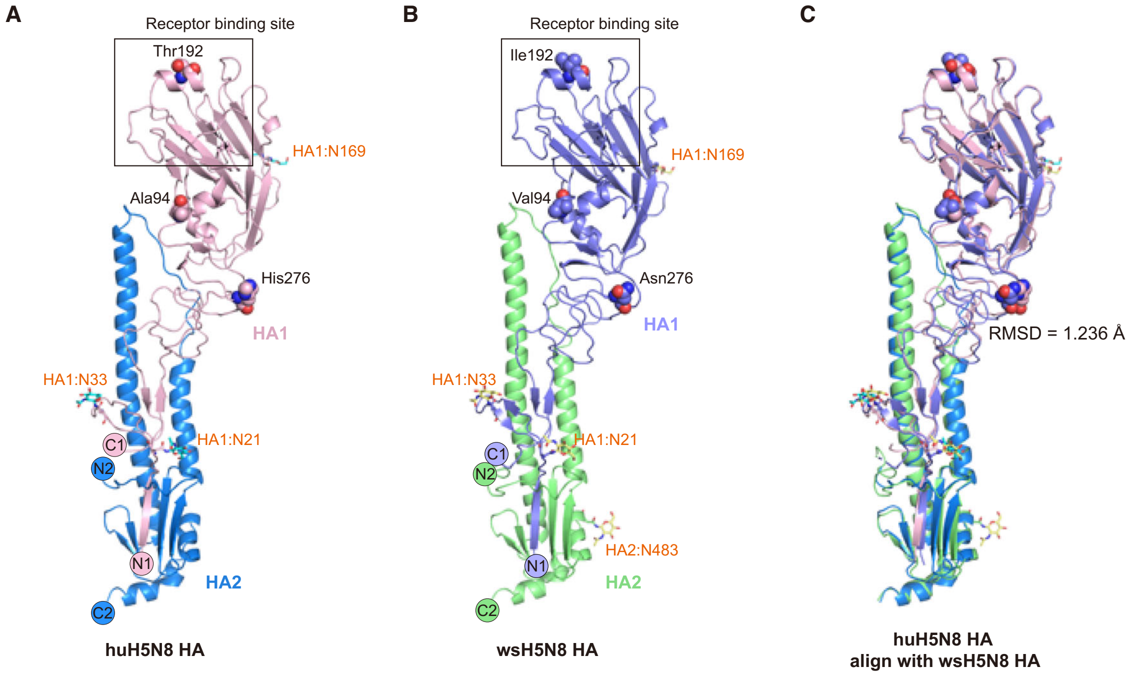

Figure EV3. Structural comparison of huH5N8 HA and wsH5N8 HA monomer.

(**A**, **B**) Cartoon representation of the huH5N8 HA and wsH5N8 HA monomer structure. HA1 is colored in light pink and HA2 in marine in huH5N8 HA, while HA1 is colored in slate and HA2 in lime in wsH5N8 HA (The N termini and C termini of HA1 and HA2 are labeled in their respective colors.). The three amino acids different between huH5N8 HA and wsH5N8 HA (position 94, 192, and 276) were presented in spheres. N-glycosylation sites and N-linked glycans (cyan in huH5N8 and pale yellow in wsH5N8) are highlighted in sticks and numbered at the Asn attachment site. (**C**) Comparison of the overall structure of huH5N8 HA and wsH5N8 HA. The RMSD is 1.744 Å for the trimer and 1.236 Å for one protomer.

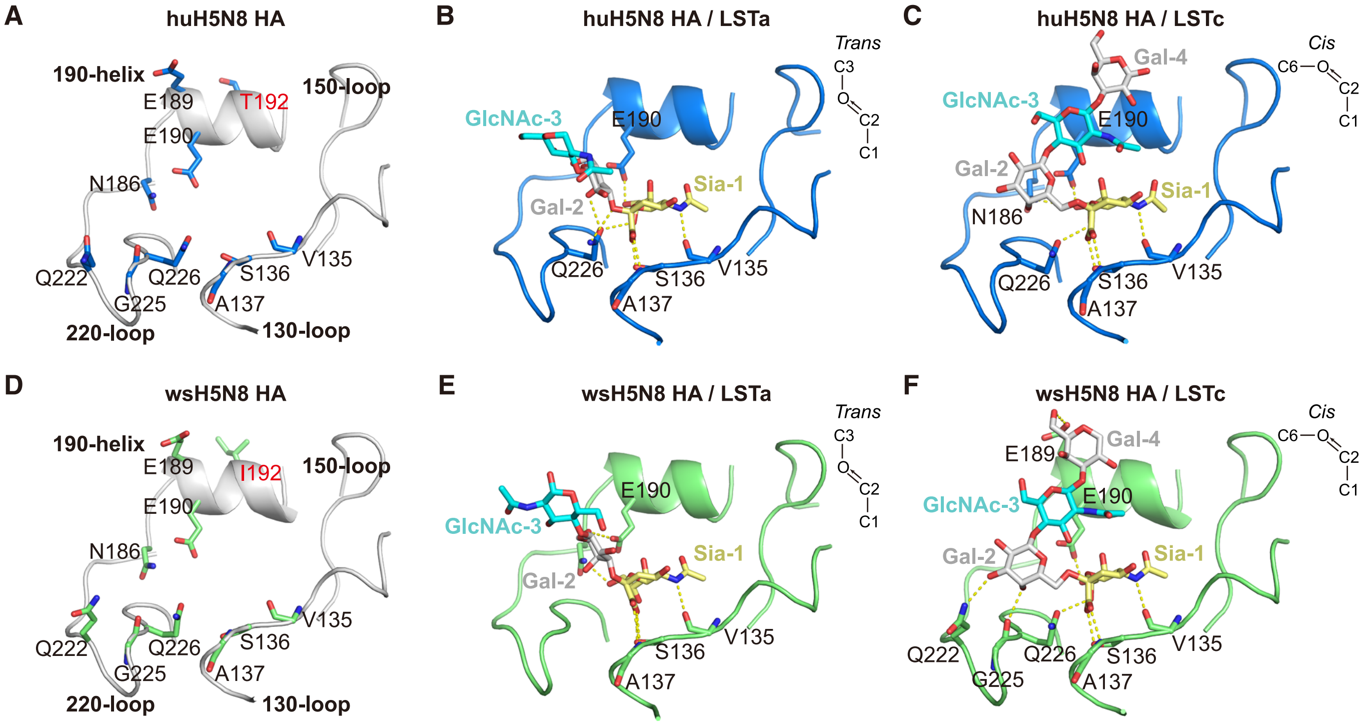

Figure EV4. Molecular interactions of huH5N8 HA and wsH5N8 HA with either avian or human receptor analogs.

Schematic representation of: (**A**) huH5N8 HA RBS with sticks representing key receptor-binding residues (colored in marine); (**B**) huH5N8 HA with LSTa bound; (**C**) huH5N8 HA with LSTc bound; (**D**) wsH5N8 HA RBS with sticks representing key receptor-binding residues (colored in lime); (**E**) wsH5N8 HA RBS with LSTa bound; and (**F**) wsH5N8 HA RBS with LSTc bound. The structure of the four secondary structural elements of the binding site (i.e., the 130-loop, 150-loop, 190-helix, and 220-loop) is labeled in ribbon representation, together with selected residues in stick representation. The hydrogen bonds are shown as dashed lines. The Sia-1 moiety of the receptor analogs is colored in pale yellow, the Gal-2 and Gal-4 moieties are colored in gray, and the GlcNAc-3 moiety is colored in aquamarine.

© The Author(s)

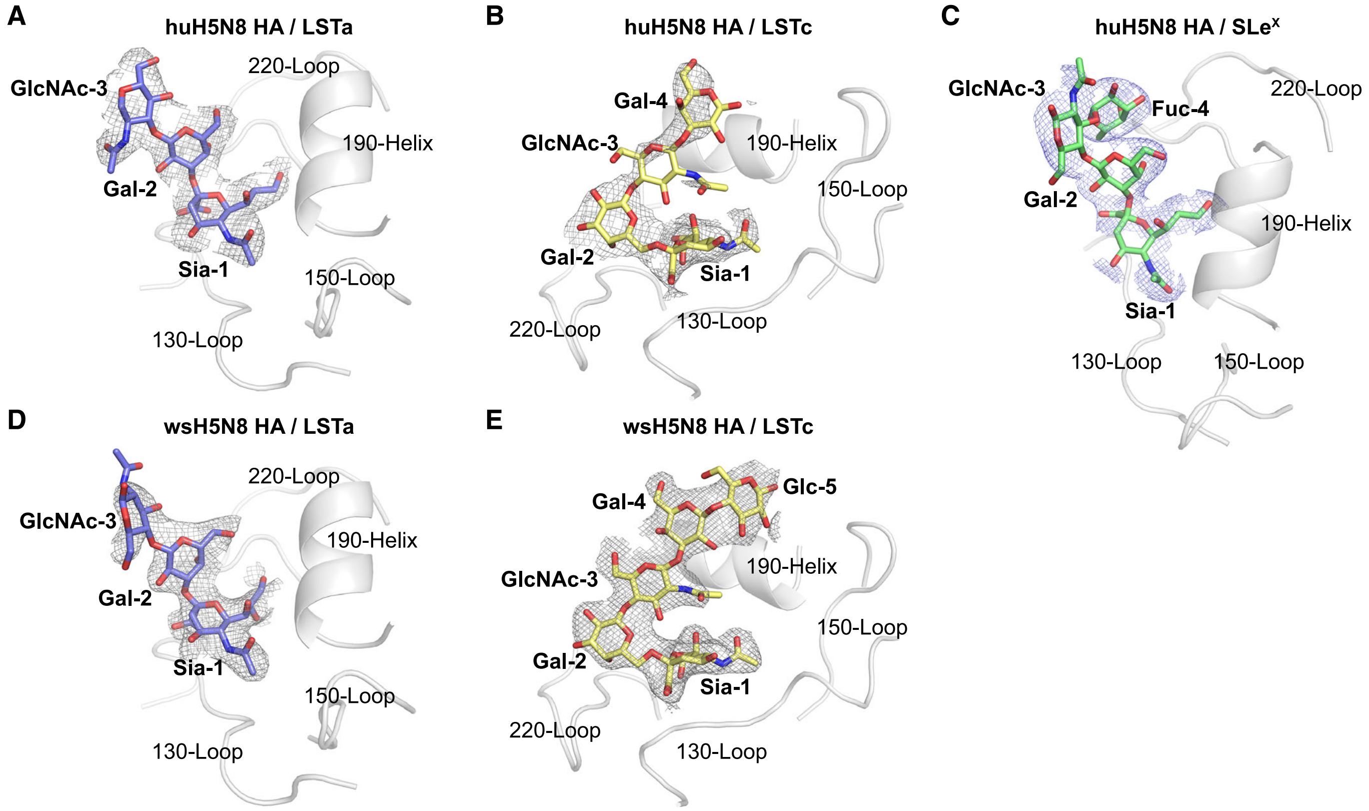

Figure EV5. 2Fo-Fc maps for the glycan receptors in the H5N8 HA/receptor complex structures.

HuH5N8 HA with LSTa (**A**), LSTc (**B**), and SLe^{x} (**C**), and wsH5N8 HA with LSTa (**D**) and LSTc (**E**). The panels show sections of the 2Fo-Fc electron density maps contoured at 0.8 sigma. The figures were drawn using PyMOL software.

© The Author(s)
