# Supplementary material for: Hemagglutinin double-mutation enhances binding of human-infecting avian influenza virus clade 2.3.4.4b H5Ny to human and SLeX receptors
Source: EMBO Rep. 2026 Jun 16;27(14):4079–99. doi: 10.1038/s44319-026-00816-2 (PMC13400655; doi:10.1038/s44319-026-00816-2)
Supplement: Supplementary file 2 — Peer Review File [file 44319_2026_816_MOESM2_ESM.pdf]

EMBO reports

Double-mutation enhances binding of human-infecting clade 2.3.4.4b H5 to human and SLeX receptors

George Gao, Xiyue Jin, Pu Han, Yuxuan Wang, Haichen Wang, Chunge Zhang, Antonio Di Maio, Jin Yu, Tianjiao Hao, Yuhang Gu, Zeyu Zhang, Wei Zhang, Jianxun Qi, Yuhai Bi, Xu Zhang, Lei Sun, Ningli Wang, Yan Liu, and Hao Song

Corresponding author(s): George Gao (gaof@im.ac.cn) , Yan Liu (yan.liu2@imperial.ac.uk), Hao Song (songhao@im.ac.cn)

Review Timeline:

Transfer Date:	9th Dec 25
Editorial Decision:	24th Mar 26
Revision Received:	17th Apr 26
Editorial Decision:	28th Apr 26
Revision Received:	12th May 26
Accepted:	22nd May 26

Editor: Martina Rembold

Transaction Report: This manuscript was transferred to EMBO Reports following peer review at The EMBO Journal.

(Note: With the exception of the correction of typographical or spelling errors that could be a source of ambiguity, letters and reports are not edited. Depending on transfer agreements, referee reports obtained elsewhere may or may not be included in this compilation. Referee reports are anonymous unless the Referee chooses to sign their reports.)

Referee #1:

The paper of Xiyue Jin describes the receptor binding properties of the 2.3.4.4b H5N8 viruses, which are the predecessors of the currently circulating H5N1 viruses. The authors employ three H5 HA proteins derived from insect cells and determine glycan binding by SPR, glycan array analysis, tissue binding, and structural analysis. Although the work is carefully done, many of the novelty claims are not true, and most observations have been previously published and/or reported on bioRxiv by several other groups. It is a pity that previous literature is hardly properly discussed nor cited, to make it appear that novel observations are made.
Given that the H5N8 HA and H5N1 HA currently circulating are so closely related, as noted by the authors, I do not see the added value of the current study. Perhaps the paper is of interest to a more specialized journal.
Major novelty problems include
• Slex binding and the responsible amino acids have been shown previously by Hiono PMID: 26542967 and Guo PMID: 27869615. Although the latter is cited, it appears to be a complete afterthought in the discussion. Furthermore, there are numerous literature examples of the H5N2 strain from the Hiono paper and a 2016 2.3.4.4b H5N8 HA, including the mutations described in this paper, that demonstrated SleX binding on a variety of glycan structures.
• Slex binding is also observed for H7 HA proteins and viruses. This needs to be discussed. Why do those viruses not have an extended host repertoire?
• A recent PNAS paper demonstrated receptor binding properties of H5N8-derived H5 proteins, including binding to human tracheal tissues.
• A recent Science paper demonstrated strict avian-type receptor specificity of 2.3.4.4b H5 and employed mutants that convert binding to human-type receptors, including structural analyses. Although it is cited, this is done in a byline.
• Slex binding by H5N8 and H5N1 viruses has been demonstrated by many papers, including the cited Good manuscript and several recent bioRxiv preprints. https://doi.org/10.1101/2025.06.26.661710 https://doi.org/10.1101/2025.04.24.650378
• Binding of H5N1 HA proteins and viruses to human respiratory tract tissue sections has also been demonstrated by several other groups using a variety of different H5 HA proteins and viruses isolated between 2016 and 2024 (doi: https://doi.org/10.1101/2024.11.27.625596
• The lack of binding to bovine respiratory tissues has also been previously demonstrated.
• There are numerous published manuscripts that refute the binding of H5 proteins and viruses to 2,6-linked SIA, including virus-based ELISAs and glycan array analyses, yet none of these are discussed.

• Is there any reason to note cite bioRxv manuscripts? In the light of open science, I think this is warranted
• Also, the structural analyses are hardly novel; a recent preprint demonstrates the fucose in a 2016 H5N8 HA protein.
Experimental problems include
• The receptor binding studies have been conducted with H5 proteins, which are derived from insect cells; their glycosylation patterns are profoundly different compared to proteins derived from mammalian cells. In nature, these H5 proteins are produced in avian or mammalian cells and then depleted of sialic acid by the neuraminidase. In light of the discussion on human-type receptor binding, I believe it is warranted to deploy these H5 proteins from mammalian cells treated with neuraminidase. Would the SPR binding to 2,6-linked SIA become negligible?
• The authors state that the lack of human-type receptor binding on the glycan array is due to a low amount of HA. Why not add more? Or analyze a virus on the array as done by others previously?
• The Slex ligand identified in this study is a mere terminal structure of more complex N- and O-linked glycans; additional data regarding binding such structures and those structures in the structural analyses would significantly add novelty to the manuscript and should be required before publication.
• The specificity of the SleX antibody has not been identified using glycan array analyses, or SPR analyses. Similarly, the H4 control is a significantly different subtype. The InH5, TxH5, H5 SleX mutants, H1, and H3 controls should also be analyzed on the glycan array.
• The tissue staining panels have different orientations; specifically, for the tracheal section, they should have the same orientation. Several plant lectin controls are missing, and finally, H-E or brightfield images are necessary to assess tissue quality properly.
• The level of reproducibility not given for any of the assays, this is vital for a Journal such as EMBOJ
• With this suite of synthetic SleX structure, it is surprising that the authors did not utilize them in competition experiments to illustrate their biological significance. Specifically, in the context of fixed tissue based binding experiments. Even in the absence of soluble glycocalyx, competition experiments in these tissue base studies would have been helpful.
• Formalin-fixed tissue from chicken and human are great starting points for binding experiments. However, normal biology of respiratory virus infection via respiratory tract would first encounter soluble glycocalyx, such as mucus. The 'mucus hurdle' is likely to remove most viral particles, which is also likely to neutralizing. Supplementation of relevant mucus on these fixed tissued based binding experiment could be very informative (and potentially strengthen) the claim of the authors regarding SleX in the biology of IAV.
• Other complementary experimental approach could be glycosylase enzyme treatment

experiments to evaluate the contribution of SleX in IAV biology. fucosidases should be employed.

Referee #2:

This study investigated the specificity and structural basis for the influenza virus clade 2.3.4.4b H5N8 hemagglutinin (HA) recognition of human and avian glycan receptors. In 2020, the 2.3.4.4b H5N8 virus caused significant losses in poultry and infected a number of humans, and this virus has reassorted with LPAIVs to generate the current HPAIV H5N1 outbreak in dairy cows. Understanding how these viruses have adapted to transmit between avian and mammalian hosts is essential to develop preventative measures.

In this study, two H5N8 HA proteins, from strains obtained from human and whooper swan samples, were demonstrated with SPR to bind both a2-3 (avian) and a2-6 (human) glycans, albeit the human glycan with ~100-fold lower affinity. These data are consistent with previously published data on the related HA from TX H5N1. A glycan array extended these findings and identified fucose-modified a2-3 glycans (SLeX) as having enhanced binding by the H5N8 HAs, and SPR studies confirmed high nanomolar affinity for SLeX. The HAs were used to stain diverse human, bovine, and duck tissues. The H5N8 HAs broadly bound all tissues that had SLeX, consistent with previous studies on TX H5N1 HA, and suggesting a potential role in SLeX in tissue tropism. Six crystal structures of H5N8 HAs alone and bound to a2-3 (avian) and a2-6 (human) glycans provide a molecular basis for glycan specificity. Finally, the cryo-EM structure of the H5N8 HA bound to SLeX reveals a structural basis for its preference for this glycan, and mutagenesis studies support the role of Q222K and R227S substitutions in these recent H5Nx HAs for SLeX binding. Notably, this is the first reported structure of a 2.3.4.4b H5 HA and SLeX. Overall, this study expands our understanding of glycan interactions by the recently emerged H5Nx HAs and adds depth to our understanding of the ability of these viruses to spread between avian and mammalian hosts.

Minor comments:

Line 88 - Clarify that these were human deaths. Also, it would be helpful to explain the symptoms and locations of infection in these individuals.

Line 112-113 - Add residues 226 and 228 to the Appendix Figure S1.

Line 137 - Reference Figure 1 again here for clarity.

Figure 3 - The triangles and pentagrams in the images are too small to distinguish, and the arrows are unnecessary to point out the green stain.

Line 182-183 states "Collectively, the conserved SLeX expression across distant species tissues creates a permissive molecular environment for H5N8 HA-mediated infection." While HA binding and SLeX stain similar tissues, these data are correlative and do not definitively show what receptor is promoting binding to these tissues. Thus, it is recommended to modify this statement.

Tables S1 and S2 - Please add the CC1/2 values for all crystal structures, to validate cutoff resolution.

Tables S1 and S2 - The Ramachandran outliers should be fixed in these structures.

Line 193 - Should be labeled Fig. S4A, S4D (not S3A, S3D).

Figure 5 - The panel labels are in the incorrect order.

Referee #3:

This is a very detailed study on the naturally occurring double mutants K222Q and S227R of the haemagglutinin of avian influenza H5N8. This avian virus subtype was reported to infect human in 2020 and was found to be able to bind to both alpha 2,6 linked and Sialyl Lewis X glycans. Structural analysis showed that these mutants can bind to these glycans abundantly present in human respiratory and duck intestinal epithelium. The authors speculated that these may facilitate cross species transmission and the reason for sporadic infection in human and widespread dissemination in birds and other mammals.

Strength
1. The finding in this study confirms the importance of K222Q and S227R in its dual binding specificity to different types of glycans.
2. This group is very strong in the area of structural biology of virus-receptor interaction.

Weakness which need to be addressed before publication

1. There is no demonstration of the importance of this mutants in vivo. All the experiments are interaction between protein-glycan or virus-cell line.
2. The author should check the differential pathogenicity of the wild type and mutants in mice or chickens (mainly 2,3 sialic acid) & ferret (mainly 2, 6 sialic acid)
3. The meaning of the sentence in line 30 to 32 is misguided. How can the double mutant facilitates robust staining of mammalian tissue. Such careless mistakes must be corrected in the abstract.

Recommendation
This paper should be accepted after addressing the weaknesses.

Point-by-point responses to reviewers' comments

We appreciate the referees' assessment of our work, and their comments on aspects that could be improved. Below we respond to each referee's points in detail, with notes as to where changes to the manuscript have been made.

Reviewers' comments:

Reviewer #1: The paper of Xiyue Jin describes the receptor binding properties of the 2.3.4.4b H5N8 viruses, which are the predecessors of the currently circulating H5N1 viruses. The authors employ three H5 HA proteins derived from insect cells and determine glycan binding by SPR, glycan array analysis, tissue binding, and structural analysis. Although the work is carefully done, many of the novelty claims are not true, and most observations have been previously published and/or reported on bioRxiv by several other groups. It is a pity that previous literature is hardly properly discussed nor cited, to make it appear that novel observations are made.

Given that the H5N8 HA and H5N1 HA currently circulating are so closely related, as noted by the authors, I do not see the added value of the current study. Perhaps the paper is of interest to a more specialized journal.

Major novelty problems include

1. Sle^X binding and the responsible amino acids have been shown previously by Hiono PMID: 26542967 and Guo PMID: 27869615. Although the latter is cited, it appears to be a complete afterthought in the discussion. Furthermore, there are numerous literature examples of the H5N2 strain from the Hiono paper and a 2016 2.3.4.4b H5N8 HA, including the mutations described in this paper, that demonstrated Sle^X binding on a variety of glycan structures.

Response: We appreciate this insightful comment regarding the literature on SLe^X. We have expanded our Discussion section to better contextualize these findings. While Hiono et al. (2016, PMID: 26542967) demonstrated SLe^X binding in earlier H5N2 influenza strains (A/chicken/Ibaraki/1/2005 [clade Am_nonGsGD; IBR] and A/duck/Mongolia/54/2001 [EA_nonGsGD; MNG]) and Guo et al. analyzed an H5N8 strain (A/chicken/Netherlands/14015526/2014 H5N8 strain [clade 2.3.4.4c]), our

study focuses on the currently prevalent 2.3.4.4b clade. Importantly, to our knowledge, our work provides the first experimentally determined structure of a clade 2.3.4.4b H5 HA bound to SLe^X, together with quantitative SPR kinetics and mutational validation (Q222K/R227S).

Lines 339-358: Hiono et al. studied recombinant HAs from two H5N2 influenza strains: A/chicken/Ibaraki/1/2005 (clade Am_nonGsGD; IBR) and A/duck/Mongolia/54/2001 (EA_nonGsGD; MNG) using glycan microarray analysis. The IBR HA (R222/R227) selectively binds to SLe^X glycans, whereas the MNG HA (K222/S227) preferentially binds to non-fucosylated α2-3 glycans. Remarkably, swapping these residues completely reversed the receptor-binding specificity of the HAs (Hiono et al, 2016). Similarly, Guo et al. reported that Q222/R227 enhances SLe^X binding in the HA of A/chicken/Netherlands/14015526/2014 H5N8 (clade 2.3.4.4c), while retaining its affinity for α2-3 glycans, as shown by glycan array analysis (Guo et al, 2017). Good et al. demonstrated that A/Texas/37/2024 H5N1 exhibits broad α2,3-sialoglycan binding, including SLe^X (Good et al, 2024). More recently, two preprints further reported SLe^X-binding capacity in clade 2.3.4.4b strains A/duck/France/161108h/2016 H5N8 and A/Bovine/OH/B24OSU-432/2024 H5N1 (Ghotekar et al, 2025; Weber et al., 2025). Our study focuses on the currently predominant clade 2.3.4.4b strains, particularly those that have infected humans, including A/Texas/37/2024 H5N1, A/Astrakhan/3212/2020 H5N8, and A/whooper swan/Henan/CAS001-K/2020 H5N8. By combining structural, biophysical, and mutational analyses, we further revealed how naturally occurring substitutions (Q222K/R227S) shape the receptor-binding pocket to accommodate SLe^X and human-type glycans. Importantly, to our knowledge, this work provides the first experimentally determined structure of a clade 2.3.4.4b H5 HA bound to SLe^X, supported by quantitative SPR kinetics and mutational validation, thereby elucidating the molecular mechanism underlying the SLe^X preference and the potential for mammalian adaptation of these human-infecting strains.

2. Sle^X binding is also observed for H7 HA proteins and viruses. This needs to be

discussed. Why do those viruses not have an extended host repertoire?

Response: We thank the reviewer for highlighting this important point. We agree that SLe^{X} binding is observed in H7 viruses and have expanded our discussion to incorporate this literature.

Lines 359-369: Beyond studies on H5 subtype IAVs, research has also explored the role of SLe^{X} in H7 subtype infections. H7 IAVs can sporadically infect mammals, including humans, and frequently cause outbreaks in domestic poultry. Guan et al. demonstrated that SLe^{X} expression varies across wild dabbling duck species. Importantly, their work revealed that H7 IAVs exhibits strong SLe^{X} binding affinity, enhancing viral attachment and infection. Binding assays showed that the H7 viruses specifically adhered to SLe^{X} expressing chicken colon crypt cells, while binding was significantly weaker in mallard cells lacking SLe^{X}. These findings strongly suggest that SLe^{X} facilitates viral attachment and infection initiation (Guan et al, 2022). Spruit et al. discovered that attachment of NeuGc-adapted H7 IAVs to tri-antennary N-glycans (particularly the α2,3-linked NeuAc and SLe^{X} structures) enables viral replication and shedding in chickens and ducks, potentially promoting cross-species transmission of equine-adapted H7 viruses (Spruit et al, 2024).

3. A recent PNAS paper demonstrated receptor binding properties of H5N8-derived H5 proteins, including binding to human tracheal tissues.

Response: We appreciate the reference. We now clarify that Sun et al. characterized the receptor usage and human tracheal binding by an H5N8 strain, whereas our work adds the structural basis for SLe^{X} recognition by clade 2.3.4.4b HA and quantitative SPR with SLe^{X} tetrasaccharide.

Lines 325-329: In contrast, Sun et al. found that a clade 2.3.4.4 H5N8 strain, A/duck/northern China/08/2014, exhibited dual receptor-binding specificity as determined by ELISA assays, and could effectively bind to the human tracheal epithelium (Sun et al, 2022). These observations are consistent with our findings, further supporting that certain H5Ny viruses possess limited yet detectable affinity for human-like receptors.

4. A recent Science paper demonstrated strict avian-type receptor specificity of 2.3.4.4b H5 and employed mutants that convert binding to human-type receptors, including structural analyses. Although it is cited, this is done in a byline.

Response: We appreciate the reviewer's thoughtful observation regarding the Lin et al.'s (2024) Science paper. We appreciate this observation. We agree Lin et al. rigorously demonstrate strict avian-type specificity for clade 2.3.4.4b on glycan arrays and identify mutations enabling α2-6 binding. Our results are complementary: at higher HA concentrations in SPR we detect weak yet quantifiable interactions with α2-6 analogs, while structurally focusing on SLe^X recognition, which was not the emphasis of Lin et al. We have cross-referenced these differences and aligned our claims accordingly.

Lines 314-316: A recent study revealed that a Q226L substitution can switch the binding specificity of TxH5N1 HA from avian-type receptors to human-type receptors (Lin et al., 2024).

Lines 320-322: Notably, structural alignment demonstrated comparable *cis* conformations between huH5N8/LSTc and the human-receptor-preferential TxH5N1mut/LSTc (Lin et al., 2024).

Lines 380-382: These discrepancies could explain the conflicting results in recent reports, some of which concluded that bovine H5 lacks α2-6-linked receptor binding based on glycan microarray analyses (Good et al., 2024; Lin et al., 2024; Pulit-Penaloza et al., 2024).

5. Sle^X binding by H5N8 and H5N1 viruses has been demonstrated by many papers, including the cited Good manuscript and several recent bioRxiv preprints. https://doi.org/10.1101/2025.06.26.661710, and https://doi.org/10.1101/2025.04.24.650378

Response: We have incorporated an expanded discussion of Good et al. (2024) and recent preprints (Ghotekar et al., 2025; Weber et al., 2025), which together support the relevance of SLeX recognition in currently circulating three distinct 2.3.4.4b clade

strains: A/Texas/37/2024 H5N1, A/duck/France/161108h/2016 H5N8, and A/Bovine/OH/B24OSU-432/2024 H5N1. These studies collectively reinforce the need for mechanistic understanding in 2.3.4.4b viruses. The recent preprints do not affect our work's novelty.

Lines 347-349: More recently, Good *et al.* (2024) demonstrated that the A/Texas/37/2024 H5N1 virus exhibits broad α2-3 glycan binding, including SLe^X, and two recent preprints further reported SLe^X-binding capacity in clade 2.3.4.4b strains A/duck/France/161108h/2016 H5N8 and A/Bovine/OH/B24OSU-432/2024 H5N1 (Ghotekar et al, 2025; Weber et al., 2025).

6. Binding of H5N1 HA proteins and viruses to human respiratory tract tissue sections has also been demonstrated by several other groups using a variety of different H5 HA proteins and viruses isolated between 2016 and 2024 (doi: https://doi.org/10.1101/2024.11.27.625596)

Response: We appreciate the reviewer's point regarding H5N1 binding to human respiratory tract tissues. Our discussion has been expanded to incorporate findings from recent studies, including Bauer et al. (2024), which demonstrates more abundant binding of the contemporary 2.3.4.4b clade H5N1 (A/Caspian gull/Netherlands/1/2022) virus to human respiratory tract sections compared to earlier clade 2.1.3.2 strains. Again, I don't think the preprints' studies affect our work's novelty.

Lines 331-333: Another preprint indicated that A/Caspian gull/Netherlands/1/2022 H5N1 virus (clade 2.3.4.4b) attached more extensively to the human respiratory tract than A/Indonesia/5/2005 (clade 2.1.3.2) (Bauer et al, 2024).

7. The lack of binding to bovine respiratory tissues has also been previously demonstrated.

Response: We appreciate this important observation regarding H5Ny HAs binding in bovine respiratory tissues. While our previous research (2025) demonstrated limited H5N1 binding in bovine trachea, our study extends these findings to H5N8.

Lines 164-178: To assess the tissue tropism profiles of huH5N8 and wsH5N8, as we recently analyzed for TxH5N1 (Song et al., 2025), we conducted immunohistochemical staining on paraffin-embedded human, bovine, and duck tissues using soluble recombinant HA proteins. The H&E staining was conducted to assess tissue quality (Appendix Fig. S2). Both huH5N8 and wsH5N8 HAs demonstrated robust binding comparable to TxH5N1 HA across multiple human tissues, including human conjunctiva (Fig. 3A), trachea (Fig. 3B), lung (Fig. 3C), and mammary gland (Fig. 3D). These bindings were particularly pronounced in the stratified columnar epithelium of the conjunctiva (Fig. 3A), the tracheal epithelial pseudostratified ciliated columnar epithelium (Fig. 3B), the alveolar cells (Fig. 3C), and the mammary gland alveolar epithelium (Fig. 3D). Notably, these HAs also exhibited significant affinity for bovine mammary gland (Fig. 3F) and duck tracheal/intestinal tissues (Fig. 3G, H). In contrast, the early InH5 showed strong binding to most tissues except human trachea. H1N1 and seasonal H3N2 HAs, known for their preferential recognition of human-like receptors, displayed poor binding across tissues aside from the human trachea (Fig. 3). Interestingly, none of the tested HAs stained bovine trachea effectively (Fig. 3E). Collectively, huH5N8 and wsH5N8 HAs exhibited the broadest tissue tropism among the tested HAs, mirroring TxH5N1 HA, in both binding intensity and multi-species tissue penetration capacity.

8. There are numerous published manuscripts that refute the binding of H5 proteins and viruses to 2,6-linked SIA, including virus-based ELISAs and glycan array analyses, yet none of these are discussed.

Response: We appreciate the reviewer's insightful comments regarding the controversial evidence on H5 binding to α2,6-linked glycans. We have significantly expanded our discussion to systematically address these conflicting findings.

Lines 376-386: Differences in research methods might explain the inconsistent findings regarding α2-6-linked glycan receptor binding among H5Ny viruses. Our glycan microarray analyses, conducted at relatively low HA concentrations, did not detect α2-6-linked glycan receptor binding for H5N8 HAs. In contrast, sensitive label-

and wash-free SPR assays performed at higher protein concentrations revealed weak but measurable binding to human receptor analogs. These discrepancies could explain the conflicting results in recent reports, some of which concluded that bovine H5 lacks α2-6-linked receptor binding based on glycan microarray analyses (Good et al., 2024; Lin et al., 2024; Pulit-Penaloza et al., 2024). More importantly, recent virus-based solid-phase assays using high-density sialyl glycopolymers detected limited α2-6-linked receptor interactions (Eisfeld et al., 2024; Gu et al., 2024), which aligns with our findings. While the viral α2-6-linked receptor binding affinity remains relatively low, even a modest enhancement could amplify multivalent binding avidity, thereby elevating the risk of enhanced viral attachment and transmission outcomes.

9. Is there any reason to note cite bioRxv manuscripts? In the light of open science, I think this is warranted.

Response: We appreciate your perspective and fully agree. In support of open science, our manuscript explicitly acknowledges several impactful preprints hosted on bioRxiv.

10. Also, the structural analyses are hardly novel; a recent preprint demonstrates the fucose in a 2016 H5N8 HA protein.

Response: We appreciate this insightful critique. To our knowledge, our study provides the first experimentally determined structure of a clade 2.3.4.4b H5 HA in complex the SLe^{X} glycan, thereby extending previous structural observations to the currently predominant and human-infecting lineage. Our structural data demonstrate that the H5Ny HA (clade 2.3.4.4b) engages human-type receptors in a manner closely resembling that of airborne-transmissible H5N1 mutants, while maintaining the canonical binding geometry for avian receptors. This reveals an evolutionary adaptation in which partial human-receptor specificity emerges without the complete loss of avian host affinity, highlighting structural plasticity at the receptor-binding pocket. Weber et al. (2025, bioRxiv preprint) recently reported that the fucose moiety forms additional hydrogen bonds with Q222 and G225 in a 2.3.4.4b strain

(A/duck/France/161108h/2016), independently supporting our conclusions and reinforcing the generality of this fucose-mediated interaction across H5Ny lineages.

Lines 236-240: Collectively, the complex structures demonstrate that the recently 2.3.4.4b H5Ny HA binds to human receptors in a manner that closely resembles the binding mode of airborne-transmissible H5N1 mutants (Fig. 5B-F). In contrast, engagement with avian receptors maintains the canonical binding geometry observed in avian-adapted HAs (Fig. 4B-F). This structural plasticity suggests an evolutionary trajectory in which partial adaptation to human receptors occurs without a complete loss of avian host specificity.

Lines 245-248: Compared to the huH5N8/LSTa complex, SLe^{X} formed two additional hydrogen bonds (Appendix Table S4). Gln222 and Gly225 established three hydrogen bonds with Fuc-4 (Fig. 6A), pulling the GlcNAc-3 glycan ring closer to the 220-loop by approximately 1.8 Å.

Lines 254-257: Consistent with our results, Weber et al. (2025, bioRxiv preprint) observed that the fucose forms additional hydrogen bonds with Q222 and G225 in a 2.3.4.4b strain (A/duck/France/161108h/2016), further validating the key role of fucose-mediated contacts in SLe^{X} recognition by H5Ny HAs.

Experimental problems include

11. The receptor binding studies have been conducted with H5 proteins, which are derived from insect cells; their glycosylation patterns are profoundly different compared to proteins derived from mammalian cells. In nature, these H5 proteins are produced in avian or mammalian cells and then depleted of sialic acid by the neuraminidase. In light of the discussion on human-type receptor binding, I believe it is warranted to deploy these H5 proteins from mammalian cells treated with neuraminidase. Would the SPR binding to 2,6-linked SIA become negligible?

Response: Thank you for this valuable comment. We acknowledge that recombinant HAs expressed in insect cells exhibit simplified glycosylation compared with those expressed in mammalian systems, mainly due to the absence of complex terminal modifications. However, structural analysis identified four potential N-linked

glycosylation sites located distal from the receptor-binding site (RBS), making it unlikely that these glycans influence receptor engagement.

Moreover, several independent studies of influenza HA receptor binding have successfully used insect cell–expressed proteins, reporting results consistent with virus-based assays (Xiong *et al.*, 2013; Yang *et al.*, 2018). Similarly, the recent *Science* paper by Lin *et al.* (2024), which demonstrated that a single mutation in TxH5N1 HA can switch receptor specificity toward human-type receptors, also utilized insect cell–derived HA proteins. Together, these precedents support the validity of our expression system and reinforce the robustness of our receptor-binding results.

Regarding the discrepancies between our results and those reported by other groups, we have reviewed the relevant articles and acknowledge the differences in findings. Several factors may contribute to these discrepancies, including variations in experimental conditions, the source of HA proteins, and differences in assay sensitivity. In our study, although the binding to α2-6 receptors was significantly weaker compared to α2-3 receptors, it was detectable at higher concentrations using highly sensitive surface plasmon resonance (SPR) assays. We believe that differences in detection methods may explained the observed discrepancies.

12. The authors state that the lack of human-type receptor binding on the glycan array is due to a low amount of HA. Why not add more? Or analyze a virus on the array as done by others previously?

Response: In our glycan array assays using the neoglycolipid (NGL)-based system, the HA concentration was maintained at the standard 50 µg/mL to minimize non-specific interactions and reduce background fluorescence. To avoid signal saturation of the α2-3 sialyl glycans and ensure fair comparison across all glycan probes, the laser power for microarray scanning was further reduced to 50%, enabling maximal signal detection without spot saturation (see Supplementary MIRAGE document). To complement the array results, we therefore used high-sensitivity SPR to measure receptor interactions over a wider range of concentrations. The SPR data

showed that clade 2.3.4.4b H5 HAs displayed measurable, although weak, binding to α2-6-linked glycans at higher protein concentrations. This binding was much weaker than that to α2-3 glycans but clearly above background, and notably stronger than that observed for the earlier InH5 strain, indicating a low-affinity yet detectable interaction with human-type receptors. While virus-based glycan arrays provide valuable information, they require high-titer live virus stocks and BSL-3 containment, which were beyond the logistical scope of this structural study. Instead, by integrating glycan array screening with quantitative SPR analysis, we achieved a complementary and reliable characterization of receptor specificity while avoiding biosafety limitations.

13. The Sle^X ligand identified in this study is a mere terminal structure of more complex N- and O-linked glycans; additional data regarding binding such structures and those structures in the structural analyses would significantly add novelty to the manuscript and should be required before publication.

Response: Thank you for your constructive feedback. We agree that investigating extended glycans could further enhance the scope of this study. However, receptor binding of influenza HA is predominantly mediated by hydrogen bonds involving the terminal residues of glycan receptors. Due to the weak affinity and intrinsic flexibility of the inner glycan rings, larger glycan structures often exhibit conformational heterogeneity that prevents clear visualization in high-resolution structural studies. Therefore, the use of terminal glycan analogs has become a well-established and widely accepted approach for dissecting HA–glycan interactions (Yamada *et al.*, 2006, *Nature*; Shi *et al.*, 2013, *Science*; Xiong *et al.*, 2013, *Nature*; Zhang *et al.*, 2013, *Science*; Lin *et al.*, 2024, *Science*).

In this work, we employed a tetrasaccharide form of SLe^X, which captures the complete terminal epitope recognized by HA. Structural analysis showed that the Sia-1, Gal-2, and Fuc-4 residues form the key hydrogen-bonding network, whereas GlcNAc-3 contributes neither hydrogen bonds nor van der Waals contacts (Fig. 6B; Appendix Table S4). This experimental strategy is consistent with a recent study by Weber *et al.* (2025, bioRxiv), which also utilized a tetrasaccharide SLe^X analog to

define fucose-mediated interactions.

14. The specificity of the Sle^X antibody has not been identified using glycan array analyses, or SPR analyses. Similarly, the H4 control is a significantly different subtype. The InH5, TxH5, H5 Sle^X mutants, H1, and H3 controls should also be analyzed on the glycan array.

Response: Thank you for this insightful comment. The SLe^X antibody used in our study was commercially sourced from BioLegend, supplied with a certificate of analysis confirming its target specificity. This antibody is widely used for its specific recognition of SLe^X and has been cited in multiple publications. Notably, recent studies (PMID: 39906564; PMID: 39508360) have independently applied this antibody for immunofluorescence staining and verified its high specificity toward SLe^X epitopes in tissue sections.

The H4N6 strain represents a typical low-pathogenic avian influenza virus, and the inclusion of its HA as a control was essential for revealing the broad α2-3-linked glycan binding spectrum of the H5N8 HA. The H4 HA thus serves as an appropriate avian-type comparator because it recognizes only α2-3-linked glycans and exhibits a narrow binding profile, providing a clear functional contrast. Importantly, our SPR results demonstrate that clade 2.3.4.4b H5 HAs have evolved a distinct SLe^X-binding capability compared with the ancestral InH5 HA (Fig. 2D–G), directly supporting the functional specificity of this interaction.

We agree that performing glycan array analyses for additional strains (TxH5, H5 mutants, H1, and H3) would further enrich the dataset. However, such analyses require custom synthesis of more than 600 lipid-linked probes by our UK-based collaborator, and the international shipment of these reagents introduces significant logistical constraints. Nevertheless, all HA–glycan interactions have been comparatively analyzed by SPR, and the combined SPR and tissue-binding data provide robust and reproducible evidence supporting the specific recognition of SLe^X by clade 2.3.4.4b H5 HAs.

15. The tissue staining panels have different orientations; specifically, for the tracheal section, they should have the same orientation. Several plant lectin controls are missing, and finally, H-E or brightfield images are necessary to assess tissue quality properly.

Response: Thank you for these valuable suggestions. We have implemented all the recommended improvements. All tracheal sections have been uniformly re-oriented to ensure visual consistency across panels (Figure 3). Plant lectin staining was previously performed for most tissues, as reported in our recently published study (Song *et al.*, 2025, *Cell*). Additional staining of duck trachea and intestinal tissues has now been completed and incorporated into Appendix Figure S2. H&E (hematoxylin and eosin) brightfield images have also been added to Appendix Figure S2 to enable assessment of tissue integrity and morphology.

Regarding the additional lectin controls mentioned by the reviewer, these specific assays were not repeated in this study to preserve the limited and irreplaceable tissue specimens. Nevertheless, the included controls sufficiently demonstrate tissue quality and confirm the specificity of HA binding patterns.

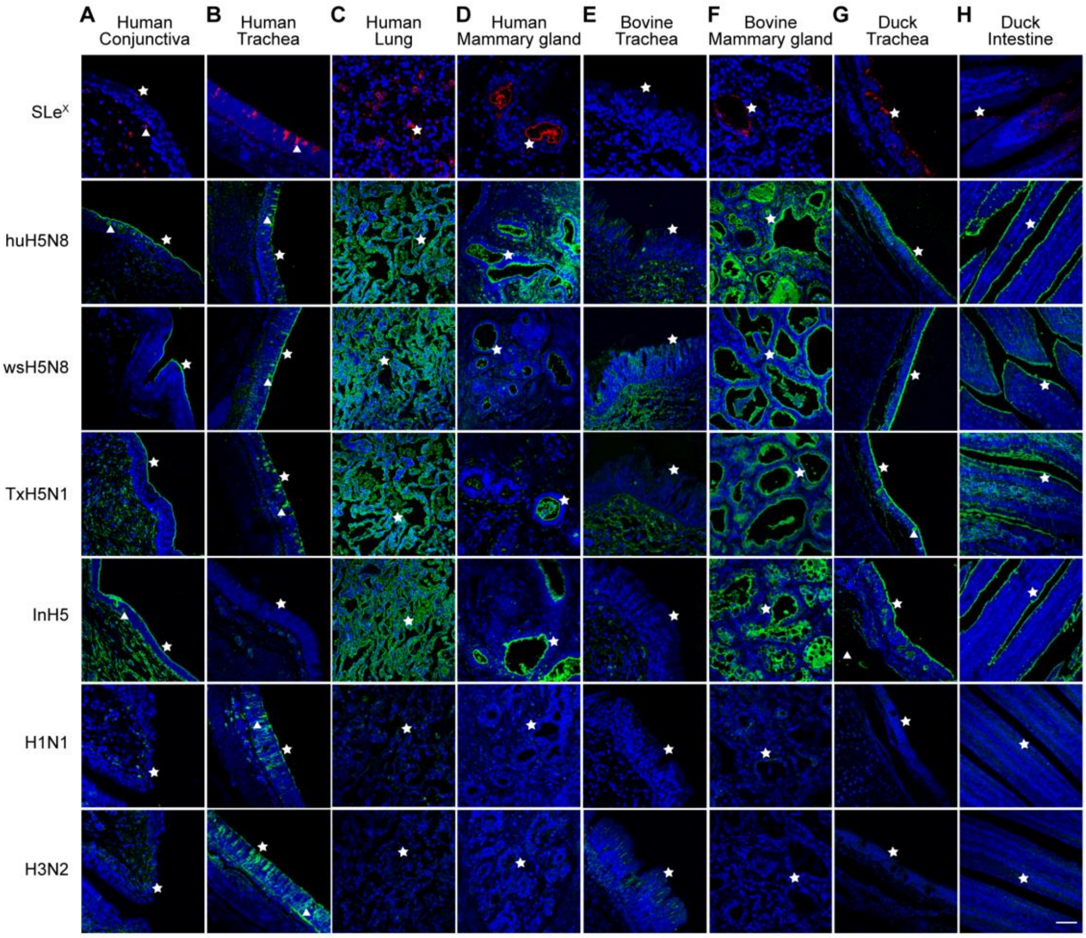

Figure 3. Host tissue tropism of H5N8 HAs and widespread distribution of SLe^{X} in tissues.

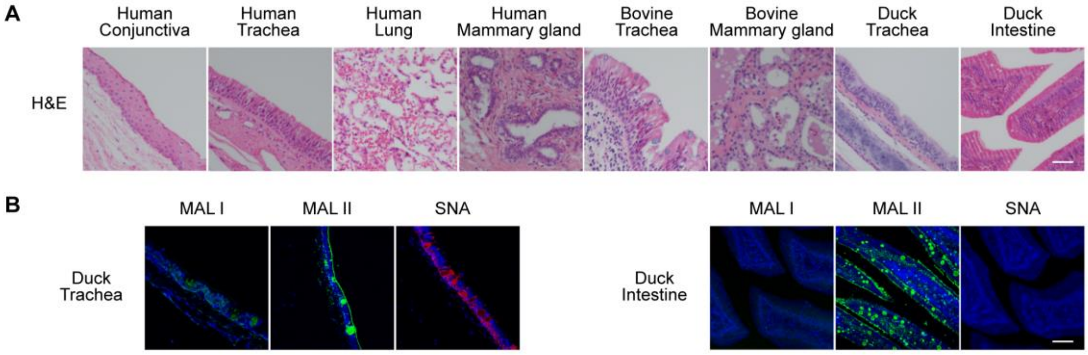

Appendix Figure S2. Representative images of H&E staining and staining of duck tissue sections with lectin.

16. The level of reproducibility not given for any of the assays, this is vital for a Journal such as EMBOJ.

Response: Thank you for emphasizing this critical requirement. We now specify in

the Methods and figure legends that all SPR and tissue staining assays were independently repeated at least twice, with consistent results. Details regarding biological replicates and experimental reproducibility have been added to the corresponding figure legends.

17. With this suite of synthetic Sle^X structure, it is surprising that the authors did not utilize them in competition experiments to illustrate their biological significance. Specifically, in the context of fixed tissue based binding experiments. Even in the absence of soluble glycocalyx, competition experiments in these tissue base studies would have been helpful.

Response: Thank you for your valuable suggestion regarding SLe^X competition experiments in fixed tissues. We acknowledge that such competition assays would provide additional functional evidence, and we plan to include them in future work. However, we were unable to perform these experiments due to experimental constraints and the limited availability of reagents. Nevertheless, Guan et al. (2022, J Virol) demonstrated the role of SLe^X in H7 viral replication and tissue specificity. H7 IAV showed enhanced replication in SLe^X-overexpressing MDCK cells compared with SLe^X-deficient cells. Furthermore, viral binding assays revealed preferential attachment to SLe^X-positive chicken colon crypt cells rather than SLe^X-negative mallard cells, confirming the functional contribution of SLe^X to viral entry.

18. Formalin-fixed tissue from chicken and human are great starting points for binding experiments. However, normal biology of respiratory virus infection via respiratory tract would first encounter soluble glycocalyx, such as mucus. The 'mucus hurdle' is likely to remove most viral particles, which is also likely to neutralizing. Supplementation of relevant mucus on these fixed tissued based binding experiment could be very informative (and potentially strengthen) the claim of the authors regarding Sle^X in the biology of IAV.

Response: Thank you for emphasizing the importance of mucus effects on influenza binding. While fixed-tissue binding assays exclude soluble glycocalyx components,

we recognize that mucus (primarily gel-forming mucins) plays a crucial role in trapping respiratory viruses. Notably, Nakamura et al. demonstrated that mucin production, driven by GALNT3 upregulation, enhances early-stage IAV replication (Nakamura et al., 2016, J Virol). Correspondingly, Weber et al. reported that 2.3.4.4b H5Ny strains have evolutionarily adapted to bind avian-type mucin O-glycans (Weber et al., 2025, bioRxiv). Together, these findings confirm the mucin-mediated regulation of IAV infection and support our claims regarding SLe^X tropism within this biological context. We agree that supplementing fixed-tissue assays with relevant mucus or mucin components could further substantiate this conclusion and will consider this refinement in follow-up studies. We prioritized cellular receptor mapping due to our research focus but acknowledge that mucus-pathogen interactions require dedicated experimental systems, such as air-liquid interface cultures.

19. Other complementary experimental approach could be glycosylase enzyme treatment experiments to evaluate the contribution of Sle^X in IAV biology. Fucosidases should be employed.

Response: We appreciate your insightful suggestion regarding the use of fucosidase treatments to assess the role of SLe^X in IAV infection. Although we were experimentally constrained from performing these enzyme assays, Guan et al. provide compelling evidence (Guan et al., 2022, J Virol). They demonstrated enhanced H7 replication in SLe^X-overexpressing MDCK cells compared to SLe^X-deficient controls, as well as preferential viral binding to SLe^X-positive chicken intestinal crypt cells. These data strongly support the functional importance of SLe^X, in line with our findings. These findings directly substantiate the mechanistic contribution of SLe^X to virus-tissue tropism, supporting our core conclusions. While enzyme-based glycan modification assays such as fucosidase treatment represent a rigorous complementary approach, we believe that our current binding and replication data already provide robust validation of SLe^X-mediated tropism mechanisms in avian influenza systems.

Reviewer #2: This study investigated the specificity and structural basis for the

influenza virus clade 2.3.4.4b H5N8 hemagglutinin (HA) recognition of human and avian glycan receptors. In 2020, the 2.3.4.4b H5N8 virus caused significant losses in poultry and infected a number of humans, and this virus has reassorted with LPAIVs to generate the current HPAIV H5N1 outbreak in dairy cows. Understanding how these viruses have adapted to transmit between avian and mammalian hosts is essential to develop preventative measures.

In this study, two H5N8 HA proteins, from strains obtained from human and whooper swan samples, were demonstrated with SPR to bind both a2-3 (avian) and a2-6 (human) glycans, albeit the human glycan with ~100-fold lower affinity. These data are consistent with previously published data on the related HA from TxH5N1. A glycan array extended these findings and identified fucose-modified a2-3 glycans (SLe^X) as having enhanced binding by the H5N8 HAs, and SPR studies confirmed high nanomolar affinity for SLe^X. The HAs were used to stain diverse human, bovine, and duck tissues. The H5N8 HAs broadly bound all tissues that had SLe^X, consistent with previous studies on TX H5N1 HA, and suggesting a potential role in SLe^X in tissue tropism. Six crystal structures of H5N8 HAs alone and bound to a2-3 (avian) and a2-6 (human) glycans provide a molecular basis for glycan specificity. Finally, the cryo-EM structure of the H5N8 HA bound to SLe^X reveals a structural basis for its preference for this glycan, and mutagenesis studies support the role of Q222K and R227S substitutions in these recent H5Nx HAs for SLe^X binding. Notably, this is the first reported structure of a 2.3.4.4b H5 HA and SLe^X. Overall, this study expands our understanding of glycan interactions by the recently emerged H5Nx HAs and adds depth to our understanding of the ability of these viruses to spread between avian and mammalian hosts.

Minor comments:

1. Line 88 - Clarify that these were human deaths. Also, it would be helpful to explain the symptoms and locations of infection in these individuals.

Response: We thank the reviewer for these valuable suggestions. We have revised the text to clarify that the fatalities occurred in whooper swans rather than humans. In addition, we have expanded the description of clinical symptoms and infection sites

observed in the infected swans.

Lines 86-89: In November 2020, an outbreak of H5N8 avian influenza virus infection in whooper swans occurred in the Yellow River Reservoir region of China. The infected swans exhibited weakness, cloudy eyes, and labored breathing, and several deaths were recorded (Li et al., 2021).

2. Line 112-113 - Add residues 226 and 228 to the Appendix Figure S1.

Response: We appreciate this helpful reminder and have made the requested additions. Residues 226 and 228 have now been highlighted in Appendix Figure S1 to facilitate comparison across the HA sequences of huH5N8, wsH5N8, TxH5N1, and early H5Ny strains.

Lines 772-776:

				SP													130-loop						150-loop	
Clade	Year	H5Ny Strain	Host	-4	2	46	55	62	81	93	101	102	111	119	127	128	131	133	137	142	144	145	159	160
1	2004	A/VietNam/1203/2004/H5N1	human	K	F	K	D	R	N	V	D	F	L	Q	S	S	A	L	S	Q	K	S	S	T
2.1.3.2	2005	A/Indonesia/5/2005/H5N1	human	K	L	T	D	R	N	T	S	F	L	Q	S	D	A	S	S	L	S	S	S	T
2.3.4.4b	2020	A/whooper_swan/Henan/CAS001-G/2020/H5N8	swan	N	L	T	N	K	R	V	S	L	L	L	P	N	T	L	A	Q	A	P	D	A
2.3.4.4b	2020	A/Astrakhan/3212/2020/H5N8	human	N	L	T	N	K	R	A	S	L	L	L	P	N	T	L	A	Q	A	P	D	A
2.3.4.4b	2024	A/Texas/37/2024/H5N1	human	N	L	T	N	K	R	A	S	L	M	Q	P	N	T	L	A	Q	A	P	D	A

							190-helix								220-loop									
Clade	Year	H5Ny Strain	Host	166	173	185	187	189	192	193	196	199	204	214	222	226	227	228	231	240	244	266	268	271
1	2004	A/VietNam/1203/2004/H5N1	human	R	Q	P	D	A	T	K	Q	T	V	V	K	Q	S	G	E	N	N	T	M	E
2.1.3.2	2005	A/Indonesia/5/2005/H5N1	human	K	Q	P	D	A	T	R	Q	T	I	V	K	Q	S	G	E	N	N	A	M	E
2.3.4.4b	2020	A/whooper_swan/Henan/CAS001-G/2020/H5N8	swan	I	R	S	N	E	I	N	K	T	V	V	Q	Q	R	G	D	D	H	T	M	G
2.3.4.4b	2020	A/Astrakhan/3212/2020/H5N8	human	I	R	S	N	E	T	N	K	T	V	V	Q	Q	R	G	D	D	H	T	M	G
2.3.4.4b	2024	A/Texas/37/2024/H5N1	human	I	R	S	N	E	T	N	K	I	V	A	Q	Q	R	G	D	D	H	T	M	G

													TM	
Clade	Year	H5Ny Strain	Host	276	285	313	325	328	481	487	510	512	522	532
1	2004	A/VietNam/1203/2004/H5N1	human	N	M	R	Q	R	V	D	I	I	V	V
2.1.3.2	2005	A/Indonesia/5/2005/H5N1	human	N	M	R	Q	S	I	N	I	T	V	M
2.3.4.4b	2020	A/whooper_swan/Henan/CAS001-G/2020/H5N8	swan	N	V	K	L	K	V	D	I	T	A	M
2.3.4.4b	2020	A/Astrakhan/3212/2020/H5N8	human	H	V	K	L	K	V	D	I	T	A	M
2.3.4.4b	2024	A/Texas/37/2024/H5N1	human	H	V	K	L	K	V	D	V	T	A	M

Appendix Figure S1. Sequence alignment of huH5N8, wsH5N8, TxH5N1 and early H5Ny HAs.

3. Line 137 - Reference Figure 1 again here for clarity.

Response: We appreciate this helpful suggestion. A reference to Figure 1 has now been added for clarity, as indicated in the revised text

Lines 138-140: This binding pattern contrasts with previous SPR data (Fig. 1A-F), where weak human receptor analog binding was observed.

4. Figure 3 - The triangles and pentagrams in the images are too small to distinguish, and the arrows are unnecessary to point out the green stain.

Response: We appreciate this technical feedback. All arrows have been removed, and the triangles and pentagrams have been enlarged by approximately 50% to improve visibility and consistency in Figure 3.

Lines 610-620:

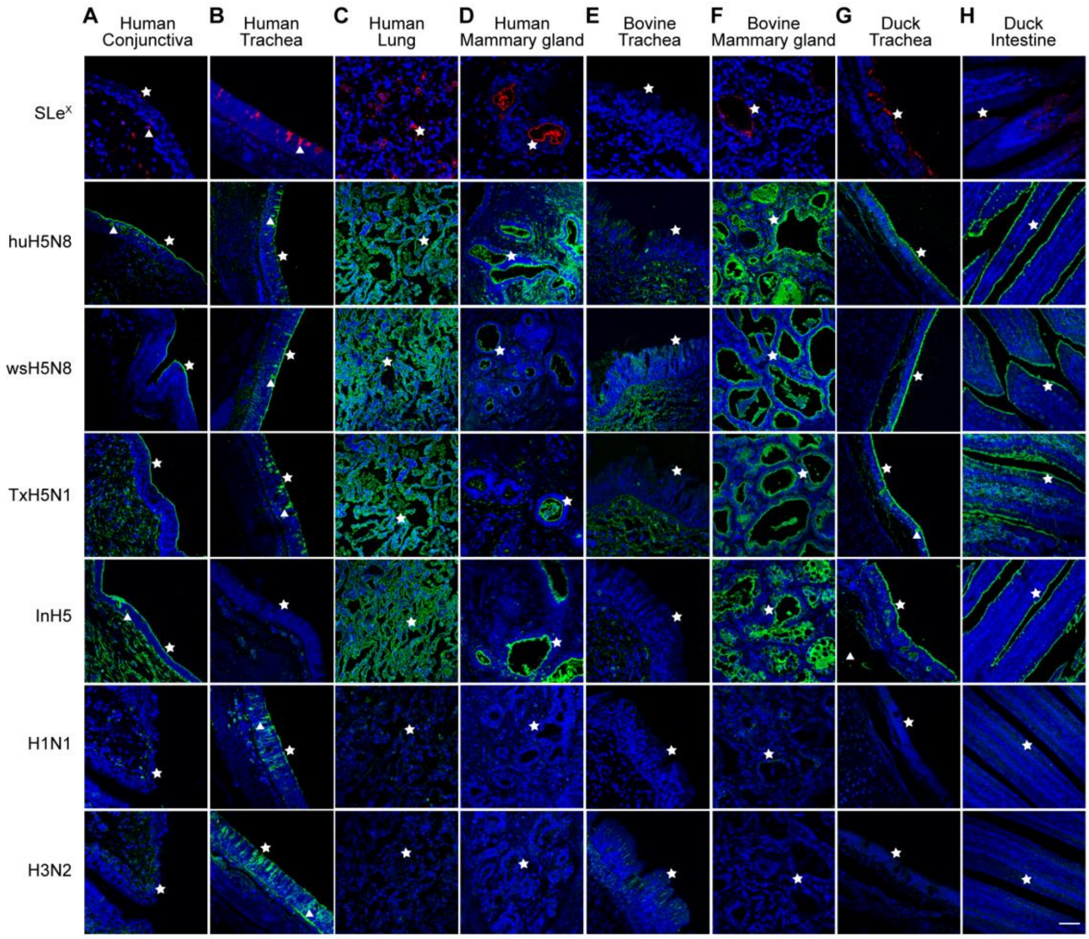

Figure 3. Host tissue tropism of H5N8 HAs and widespread distribution of SLe^{X} in tissues.

5. Line 182-183 states "Collectively, the conserved SLe^{X} expression across distant species tissues creates a permissive molecular environment for H5N8 HA-mediated infection." While HA binding and SLe^{X} stain similar tissues, these data are correlative and do not definitively show what receptor is promoting binding to these tissues. Thus, it is recommended to modify this statement.

Response: We are grateful for your meticulous suggestions. The statement has been

revised to avoid overinterpretation and to better reflect the correlative nature of the data.

Lines 192-194: Collectively, the conserved expression of SLe^{X} across tissues in diverse species may contribute to the broad tissue tropism of H5N8 HA, encompassing the ocular, respiratory, and glandular systems.

6. Tables S1 and S2 - Please add the CC1/2 values for all crystal structures, to validate cutoff resolution.

Response: Thank you for your professional advice. We have already added this data in revised Tables S1 and S2.

7. Tables S1 and S2 - The Ramachandran outliers should be fixed in these structures.

Response: Thank you for your professional advice. The Ramachandran outliers have been corrected, and the updated validation statistics are now included in revised Tables S1 and S2.

8. Line 193 - Should be labeled Fig. S4A, S4D (not S3A, S3D).

Response: Thank you for your reminder and request. The figure references have been corrected accordingly (now Fig. S5A and S5D) in the revised manuscript.

Lines 202-203: Both complexes exhibited well-defined electron density for the terminal trisaccharide motif (Sia1-Gal2-GlcNAc3) (Appendix Fig. S5A, S5D).

9. Figure 5 - The panel labels are in the incorrect order.

Response: Thank you for your comment. We have corrected panel labeling in Figure 5.

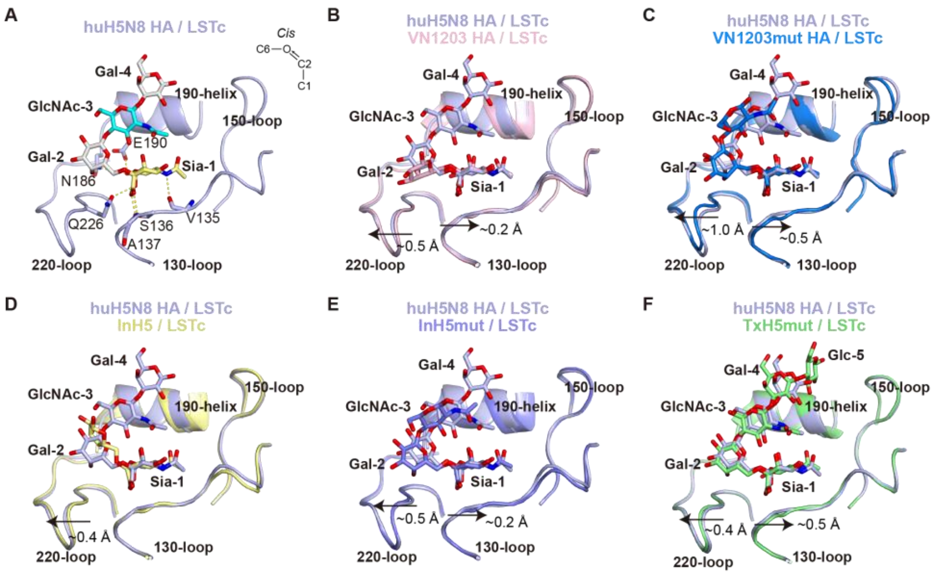

Figure 5. Structural comparison of huH5N8 HA with LSTc to other H5 HAs bound to LSTc.

Reviewer #3: This is a very detailed study on the naturally occurring double mutants K222Q and S227R of the haemagglutinin of avian influenza H5N8. This avian virus subtype was reported to infect human in 2020 and was found to be able to bind to both alpha 2,6 linked and Sialyl Lewis X glycans. Structural analysis showed that these mutants can bind to these glycans abundantly present in human respiratory and duck intestinal epithelium. The authors speculated that these may facilitate cross species transmission and the reason for sporadic infection in human and widespread dissemination in birds and other mammals.

Strength

1. The finding in this study confirms the importance of K222Q and S227R in its dual binding specificity to different types of glycans.

2. This group is very strong in the area of structural biology of virus-receptor interaction.

Recommendation

This paper should be accepted after addressing the weaknesses.

Weakness which needs to be addressed before publication.

1. There is no demonstration of the importance of this mutant in vivo. All the experiments are interaction between protein-glycan or virus-cell line.

Response: We appreciate your valuable suggestion. We fully acknowledge that in vivo validation would further strengthen the mechanistic conclusions. However, due

to biosafety and logistical constraints, such experiments could not be performed in the current study. Our work therefore focuses on protein-level, biophysical, and structural analyses to precisely define the receptor-binding characteristics of clade 2.3.4.4b H5 HAs. These findings provide a solid molecular foundation for future in vivo studies aimed at investigating the functional impact of these mutations on viral transmission and pathogenicity.

2. The author should check the differential pathogenicity of the wild type and mutants in mice or chickens (mainly 2,3 sialic acid) & ferret (mainly 2, 6 sialic acid).

Response: We appreciate your constructive suggestion. Indeed, comparative pathogenicity studies in different animal models (avian and mammalian) would be highly informative. However, since this virus has not been reported in China and is subjected to stringent biosafety and animal use restrictions, we were unable to perform such experiments. Our current research therefore centers on elucidating the receptor-binding specificity, structural adaptations, and tissue tropism of the H5N8 HA at the molecular level. We believe these insights are essential prerequisites for future animal infection studies.

3. The meaning of the sentence in line 30 to 32 is misguided. How can the double mutant facilitate robust staining of mammalian tissue. Such careless mistakes must be corrected in the abstract.

Response: Thank you for your comment. We have rigorously revised the abstract to avoid any ambiguity.

Lines 30-33: Here, we demonstrate that naturally occurring double mutations (K222Q and S227R) in the hemagglutinin (HA) of the human-infecting H5N8 strain, first identified in 2020, enhance binding affinity for both α2-6-linked and Sialyl Lewis^{X} (SLe^{X}) glycans, which may underlie the broad tissue binding and cross-species potential.

Dear George,

I sincerely apologize for the unusual delay in handling your manuscript. I have meanwhile received feedack from referee 2 who considered her concerns rather minor and I have therefore checked these myself. In addition, I received feedback from an expert advisor in the field, which I copy below. The advisor considers your structural data of value and response to the referee concerns adequate but also considers textual changes and an extended discussion important.

Comments from the advisor:

"Reviewer 1 has merit. Main critiques brought by the reviewer are the fact that there is nothing fundamentally new on the amino acids in the HA responsible for receptor specificity towards sugars of H5 viruses, and that the studies do not demonstrate that the receptor specificity conferred by the studied amino acid polymorphisms are responsible for the enhanced ability of recent H5 viruses to infect both birds and mammals, including humans, at the same time. And I agree with both points.

Having said that, it is true that this is a well conducted study, that sheds light on sialic acid preferences of the recent H5 viruses, and that includes a novel structure that demonstrates the molecular basis for these preferences. For me, this is enough to merit publication in EMBOR. But the authors need to moderate their conclusions. While the data suggest that the sugar preference for the recent H5s might enhance their ability to infect both ducks and mammals, this is not demonstrated here. Only by generating loss of function viruses and comparing their infectivity in ducks and mammals one can conclude this. I understand the bias of the authors to overstate the implications of their study to increase the impact of the data, but one does not need to push the conclusions, as the data are interesting and compelling enough (at least for me) to be published in EMBOR. So I would recommend the authors to still modify the ms to moderate the conclusions to indicate that although the data are quite suggestive, more studies are needed to demonstrate whether these mutations are implicated in enhanced host tropism for H5 viruses. The authors should also discuss the possibility that binding to non-sialic acid receptors, for example binding to MHC class II, should also be examined in the near future as an additional factor that might broad the tropism of these viruses."

Please address these concerns in the manuscript and please highlight the changes to ease evaluation.

In addition, I need you to format your manuscript along our guidelines. You will find below our general formatting guidelines, but I have listed a few speficic points that will need to be addressed:

- Please order the manuscript sections like this:
Title page - Abstract - Introduction - Results - Discussion - Methods - Acknowledgements - Disclosure and competing interests statement - References - Figure legends - Tables and their legends (not EV tables) - Expanded View Figure legends

- The Reagents and Tools table needs to be uploaded as separate Word file. Please use our template which you can download from our Author Guidelines.

- The Deposited Data you currently have in the R&T table needs to be listed in a Data Availability section at the end of Materials following this outline:

The datasets produced in this study are available in the following databases:
• data type]: [name of the resource] [accession number/identifier/doi] ([URL or identifiers.org/DATABASE:ACCESSION])

- We need the figures as individual production quality files.

- The Appendix Figures and Tables and their legends need to be combined to an Appendix PDF, which needs a title page with a table of content and page numbers.

- Appendix Supplementary Information seems to be a complex dataset and should be uploaded as .xls file called Dataset EV1. The legend is added to a tab in the .xls file.

- All funding listed in the Acknowledgment must be specified in the online manuscript submission system as well. The information needs to be congruent.

- Regarding the Author Contributions, we now use CRediT to specify the contributions of each author in the journal submission system. CRediT replaces the author contribution section, which therefore needs to be removed from the manuscript text. You can use the free text box in our system if you wish to provide more detailed descriptions. See also guide to authors

- The synopsis image needs to be uploaded as a separte file.

- Please note point (8) below regarding source data.

- Please fill the Author Checklist (point 5)

- I noticed a callout to Extended Data Fig. 5 and Extended Data Table 5 in the legend of Figure 2, which need to be updated to match our format.

Once your formatted manuscript is back it, our editorial assistants will perform a check, which should be rapid if everything is formatted along these lines.

GENERAL FORMATTING GUIDELINES:

When submitting your revised manuscript, please review the submission guidelines (https://link.springer.com/journal/44319/submission-guidelines#cms-Revised-submissions) and carefully review the instructions that follow below. Failure to include requested items will delay the evaluation of your revision.

1) a .docx formatted version of the manuscript text (including legends for main figures, EV figures and tables). Please make sure that the changes are highlighted to be clearly visible.

2) individual production quality figure files as .eps, .tif, .jpg (one file per figure). See hhttps://media.springernature.com/original/springer-cms/rest/v1/content/27825798/data/v1 for more info on how to prepare your figures.

3) We replaced Supplementary Information with Expanded View (EV) Figures and Tables that are collapsible/expandable online. A maximum of 5 EV Figures can be typeset. EV Figures should be cited as 'Figure EV1, Figure EV2" etc... in the text and their respective legends should be included in the main text after the legends of regular figures.

- For the figures that you do NOT wish to display as Expanded View figures, they should be bundled together with their legends in a single PDF file called *Appendix*, which should start with a short Table of Content. Appendix figures should be referred to in the main text as: "Appendix Figure S1, Appendix Figure S2" etc. See 'Expanded View' section of the submission guidelines.

- Additional Tables/Datasets should be labeled and referred to as Table EV1, Dataset EV1, etc. Legends have to be provided in a separate tab in case of .xls files. Alternatively, the legend can be supplied as a separate text file (README) and zipped together with the Table/Dataset file.

4) a .docx formatted letter INCLUDING the reviewers' reports and your detailed point-by-point responses to their comments. As part of the EMBO Press transparent editorial process, the point-by-point response is part of the Review Process File (RPF), which will be published alongside your paper.

5) a complete author checklist, which you can download from our author guidelines. Please insert information in the checklist that is also reflected in the manuscript. The completed author checklist will also be part of the RPF.

6) Please note that all corresponding authors are required to supply an ORCID ID for their name upon submission of a revised manuscript (<https://orcid.org/>), which can be linked to your profile in the manuscript submission system.

7) Before submitting your revision, primary datasets produced in this study need to be deposited in an appropriate public database. Please remember to provide a reviewer password if the datasets are not yet public. The accession numbers and database should be listed in a formal "Data Availability" section placed after Methods. Please note that the Data Availability Section is restricted to new primary data that are part of this study. * Note - All links should resolve to a page where the data can be accessed. *

If your study has not produced novel datasets, please mention this fact in the Data Availability Section.

8) At EMBO Press we ask authors to provide source data for the main manuscript figures. You will receive a separate email with instructions for providing source data with your revised manuscript, including how to upload and organize the files.

9) Our journal also encourages inclusion of *data citations in the reference list* to directly cite datasets that were re-used and obtained from public databases. Data citations in the article text are distinct from normal bibliographical citations and should directly link to the database records from which the data can be accessed. In the main text, data citations are formatted as follows: "Data ref: Smith et al, 2001" or "Data ref: NCBI Sequence Read Archive PRJNA342805, 2017". In the Reference list, data citations must be labeled with "[DATASET]". A data reference must provide the database name, accession number/identifiers and a resolvable link to the landing page from which the data can be accessed at the end of the reference.

10) Regarding data quantification, the following points must be specified in each figure legend:

- the name of the statistical test used to generate error bars and P values,

- the number (n) of independent experiments (please specify technical or biological replicates) underlying each data point,

- the nature of the bars and error bars (s.d., s.e.m.),

- If the data are obtained from n {less than or equal to} 5, show the individual data points in addition to the SD or SEM.
- If the data are obtained from n {less than or equal to} 2, use scatter blots showing the individual data points.

Discussion of statistical methodology can be reported in the materials and methods section, but figure legends should contain a basic description of n, P and the test applied.

- Please also include scale bars in all microscopy images.

11) The journal requires a statement specifying whether or not authors have competing interests (defined as all potential or actual interests that could be perceived to influence the presentation or interpretation of an article). In case of competing interests, this must be specified in your disclosure statement.

12) All Materials and Methods need to be described in the main text using our 'Structured Methods' format, which is required for all research articles. According to this format, the Methods section includes a Reagents and Tools Table (listing key reagents, experimental models, software and relevant equipment and including their sources and relevant identifiers) followed by a Methods and Protocols section describing the methods using a step-by-step protocol format. The aim is to facilitate adoption of the methodologies across labs. More information on how to adhere to this format as well as a downloadable template (.docx) for the Reagents and Tools Table can be found in our author guidelines.

We would also welcome the submission of cover suggestions, or motifs to be used by our Graphics Illustrator in designing a cover.

As part of the EMBO publication's Transparent Editorial Process, EMBO reports publishes online a Review Process File (RPF) to accompany accepted manuscripts. This File will be published in conjunction with your paper and will include the referee reports, your point-by-point response and all pertinent correspondence relating to the manuscript.

You are able to opt out of this by letting the editorial office know (emboreports@embo.org). If you do opt out, the Review Process File link will point to the following statement: "No Review Process File is available with this article, as the authors have chosen not to make the review process public in this case."

I look forward to seeing a revised form of your manuscript when it is ready.

Kind regards,

Martina

Martina Rembold, PhD
Senior Editor
EMBO reports

Point-by-point responses:

"Reviewer 1 has merit. Main critiques brought by the reviewer are the fact that there is nothing fundamentally new on the amino acids in the HA responsible for receptor specificity towards sugars of H5 viruses, and that the studies do not demonstrate that the receptor specificity conferred by the studied amino acid polymorphisms are responsible for the enhanced ability of recent H5 viruses to infect both birds and mammals, including humans, at the same time. And I agree with both points.

Having said that, it is true that this is a well conducted study, that sheds light on sialic acid preferences of the recent H5 viruses, and that includes a novel structure that demonstrates the molecular basis for these preferences. For me, this is enough to merit publication in EMBOR. But the authors need to moderate their conclusions. While the data suggest that the sugar preference for the recent H5s might enhance their ability to infect both ducks and mammals, this is not demonstrated here. Only by generating loss of function viruses and comparing their infectivity in ducks and mammals one can conclude this. I understand the bias of the authors to overstate the implications of their study to increase the impact of the data, but one does not need to push the conclusions, as the data are interesting and compelling enough (at least for me) to be published in EMBOR. So, I would recommend the authors to still modify the manuscript to moderate the conclusions to indicate that although the data are quite suggestive, more studies are needed to demonstrate whether these mutations are implicated in enhanced host tropism for H5 viruses. The authors should also discuss the possibility that binding to non-sialic acid receptors, for example binding to MHC class II, should also be examined in the near future as an additional factor that might broad the tropism of these viruses."

Response: We are grateful for your acknowledgment and the rigorous, professional comments you have provided. We have addressed these concerns in accordance with your suggestions.

Lines 35-37: Structural analyses reveal that these mutations expand receptor specificity

for these glycans, which are abundant in the human respiratory tract and duck trachea, providing a possible molecular basis for cross-species transmission.

Lines 104-108: Our findings show that both H5N8 and bovine H5N1 have acquired dual receptor specificity, targeting both α2-6-linked sialic acids and SLe^X. While these data suggest that such adaptation may enhance the ability to infect a range of hosts and could facilitate sporadic human infections, further studies are needed to directly evaluate the contribution of these mutations to transmission of clade 2.3.4.4b H5Ny viruses.

Lines 436-441: Further functional studies, including assessments using loss-of-function viruses, are needed to directly evaluate the contribution of these mutations to 2.3.4.4b H5Ny virus transmission. Additionally, close monitoring should consider the possibility that binding to non-sialic acid receptors, such as MHC class II (Giotis et al, 2019; Karakus et al, 2024; Karakus et al, 2019), may also influence influenza virus tropism as the virus continues to evolve.

In addition, I need you to format your manuscript along our guidelines. You will find below our general formatting guidelines, but I have listed a few specific points that will need to be addressed:

Response: We have formatted the manuscript in accordance with the journal's guidelines.

- Please order the manuscript sections like this:

Title page - Abstract - Introduction - Results - Discussion - Methods - Acknowledgements - Disclosure and competing interests statement - References - Figure legends - Tables and their legends (not EV tables) - Expanded View Figure legends

Response: Completed.

- The Reagents and Tools table needs to be uploaded as separate Word file. Please use our template which you can download from our Author Guidelines.

Response: We have now provided as required.

- The Deposited Data you currently have in the R&T table needs to be listed in a Data Availability section at the end of Materials following this outline:

Response: Completed.

The datasets produced in this study are available in the following databases:

• data type]: [name of the resource] [accession number/identifier/doi] ([URL or identifiers.org/DATABASE:ACCESSION])

Response: Completed.

- We need the figures as individual production quality files.

Response: Completed.

- The Appendix Figures and Tables and their legends need to be combined to an Appendix PDF, which needs a title page with a table of content and page numbers.

Response: Completed.

- Appendix Supplementary Information seems to be a complex dataset and should be uploaded as .xls file called Dataset EV1. The legend is added to a tab in the .xls file.

Response: Completed.

- All funding listed in the Acknowledgment must be specified in the online manuscript submission system as well. The information needs to be congruent.

Response: Completed.

- Regarding the Author Contributions, we now use CRediT to specify the contributions of each author in the journal submission system. CRediT replaces the author contribution section, which therefore needs to be removed from the manuscript text. You can use the free text box in our system if you wish to provide more detailed descriptions. See also guide to authors

Response: We have checked and modified as suggested.

- The synopsis image needs to be uploaded as a separate file.

Response: Completed.

- Please note point (8) below regarding source data.

Response: We have now provided as required.

- Please fill the Author Checklist (point 5)

Response: Completed.

- I noticed a callout to Extended Data Fig. 5 and Extended Data Table 5 in the legend of Figure 2, which need to be updated to match our format.

Response: We have checked and modified as suggested.

Manuscript number: EMBOR-2025-63394V2
Title: Double-mutation enhances binding of human-infecting clade 2.3.4.4b H5 to human and SLeX receptors
Author(s): George Gao, Xiyue Jin, Pu Han, Yuxuan Wang, Haichen Wang, Chunge Zhang, Antonio Di Maio, Jin Yu, Tianjiao Hao, Yuhang Gu, Zeyu Zhang, Wei Zhang, Jianxun Qi, Yuhai Bi, Xu Zhang, Lei Sun, Ningli Wang, Yan Liu, and Hao Song

Dear George,

Thank you for your patience while we have reviewed your revised manuscript from the editorial side. All looks fine except for a few formatting issues that need to be taken care of. I am therefore writing with an 'accept in principle' decision, which means that I will be happy to accept your manuscript for publication once the issues/corrections have been addressed, as follows.

- Please confirm whether the characters for authors names on the title page are chinese or japanese. I assume the former.

- Callouts to figures should be in a sequential manner. Here we note that Fig. 6 is called out before Fig. 3A.
- A callout for Fig. 6G missing.

- The dataset legends need to be removed from the manuscript and provided in the same sheet of each Excel file or in another sheet/tab of the Excel file(s).
Dataset EV1: I suggest to add the legend (description) that is currently at the end of the list to a separate tab called legend.

- Please upload the Appendix as a PDF file.

- Could you please provide the synopsis image as jpeg, TIFF or png format: 550 pixels wide. Ideally it is in landscape up to 350 pixels high, but the current format is also fine as long as it has the correct file format.

- Please remove the synopsis image and text from the manuscript file and upload the text as separate document. Please also add a one or two-sentence summary of the findings.

- Bauer et al 2024, Ghotekar et al 2025, and Weber et al 2025 are preprints. Please cite them as (preprint: Bauer et al, 2024) in the text and add the tag [PREPRINT] at the end of the reference in the reference list.

- "The approval of the study protocol was obtained from the Ethics Committee of Beijing Ditan Hospital, Capital Medical University." Do you also have an approval number that could be addedd?

- BioRender should be acknowledged at the end of the Methods section in the following way:
Graphics:
(some of the... OR Figure #... OR synopsis) Graphics were created with BioRender.com.

- Data availability statement: Please add the specific URLs for datasets (PDB - 8X28, 8X2D, 8X26, 8X27, 8X2F, 8X29, 8X2C).

- Figure legends:
- Please note that the legends for figures EV-4; are not provided in the sequential manner (legend for figure EV-4D is provided before legend of figure EV-4B,C). This needs to be rectified.
- Please note that the legend for figures 1G-J are missing in the manuscript. This needs to be rectified.
- Please note that the error bars are not defined in the legends of figures 2A,B,C.
- Please note that the white pentagrams are not defined in the legend of figures 3C,G,H. This needs to be rectified.
- Please note that the white triangles are not defined in the legend of figure 3G; This needs to be rectified.

- I think the title should define H5 as avian influenza virus to make it more accessible to the general readership.

Once you have made these minor revisions, please use the following link to submit your corrected manuscript:

Link Not Available

If all remaining corrections have been attended to, you will then receive an official decision letter from the journal accepting your manuscript for publication in the next available issue of EMBO reports. This letter will also include details of the further steps you need to take for the prompt inclusion of your manuscript in our next available issue.

Thank you for your contribution to EMBO reports.

Best regards,

Martina

Martina Rembold, PhD
Senior Editor
EMBO reports

Point-by-point responses:

- Please confirm whether the characters for authors names on the title page are chinese or japanese. I assume the former.

Response: Thank you for the query. The authors' names on the title page are Chinese characters (not Japanese), as you assumed.

- Callouts to figures should be in a sequential manner. Here we note that Fig. 6 is called out before Fig. 3A.

Response: Thank you for pointing this out. This was a typographical error and has been corrected.

Lines 166-168: In this study, we found that the affinity of recent strains, including huH5N8, wsH5N8 and TxH5N1 HAs, for SLe^X is comparable to that for 3’SLNLN (**Fig. 1A, C, E; Fig. 2D-F**).

- A callout for Fig. 6G missing.

Response: Thank you for pointing this out. The missing callout for Fig. 6G has been added accordingly.

Lines 323-328: We further examined the tissue tropism of huH5N8 Q222K_R227S HA. Despite reduced binding affinity, huH5N8 Q222K_R227S HA maintained a binding profile similar to wild-type huH5N8 HA across multiple tissues (**Fig. 6G**), including the conjunctiva, trachea, lung, and mammary gland in humans, as well as bovine mammary gland, duck tracheal, and duck intestinal tissues.

- The dataset legends need to be removed from the manuscript and provided in the same sheet of each Excel file or in another sheet/tab of the Excel file(s).

Dataset EV1: I suggest to add the legend (description) that is currently at the end of the list to a separate tab called legend.

Response: Thank you for raising this. The legends have been removed from the manuscript. For Dataset EV1, the legend (description) has been placed in a separate tab named "legend," as suggested. For other datasets, the legends have also been provided in a separate sheet of the respective Excel files, in accordance with your instructions.

- Please upload the Appendix as a PDF file.

Response: As requested, we have uploaded the appendix in PDF format.

- Could you please provide the synopsis image as jpeg, TIFF or png format: 550 pixels wide. Ideally it is in landscape up to 350 pixels high, but the current format is also fine as long as it has the correct file format.

Response: We have uploaded the synopsis image as a JPEG file (550 pixels wide), as requested. The current aspect ratio is retained, which the editor indicated would be acceptable as long as the file format is correct.

- Please remove the synopsis image and text from the manuscript file and upload the text as separate document. Please also add a one or two-sentence summary of the findings.

Response: Thank you for the instructions. We have deleted the synopsis image and text from the manuscript, uploaded the text as a separate document, and included a brief (one-sentence) summary of the findings, all in accordance with your request.

- Bauer et al 2024, Ghotekar et al 2025, and Weber et al 2025 are preprints. Please cite them as (preprint: Bauer et al, 2024) in the text and add the tag [PREPRINT] at the end of the reference in the reference list.

Response: Thank you for your reminders. These three preprints have been accepted. We have updated the corresponding information.

Lines 303-306: Consistent with our results, Weber et al. observed that the fucose forms additional hydrogen bonds with Q222 and G225 residues in a 2.3.4.4b strain (A/duck/France/161108h/2016) (Weber *et al*, 2026), further validating the key role of fucose-mediated contacts in SLe^X recognition by H5Ny HAs.

Lines 380-386: Additionally, a recent study reported that the HAs of A/duck/France/161108h/2016 (clade 2.3.4.4b H5N8), efficiently bound to both chicken and human tracheal tissues(Weber *et al.*, 2026). Another article found that the A/Caspian gull/Netherlands/1/2022 H5N1 virus (clade 2.3.4.4b) attached more extensively to the human respiratory tract than A/Indonesia/5/2005 (clade 2.1.3.2) (Bauer *et al*, 2026).

Lines 400-402: More recently, two studies further reported SLe^X-binding capacity in the clade 2.3.4.4b strains A/duck/France/161108h/2016 H5N8 and A/Bovine/OH/B24OSU-432/2024 H5N1(Ghotekar *et al*, 2025; Weber *et al.*, 2026).

Lines 615-617: Bauer L, Leijten L, Iervolino M, Chopra V, van Dijk L, Power M, Rijnink W, Pronk M, Spronken M, Funk M *et al* (2026) Attachment and replication of clade 2.3.4.4b influenza A (H5N1) viruses in human respiratory epithelium: an in-vitro study. *Lancet Microbe* 7: 101230

Lines 649-650: Ghotekar BK, Bhagwat SK, Chopra P, Buckley T, Boons GJ (2025) Chemoenzymatic Synthesis of Asymmetric Bisecting Bi-, Tri-, and Tetra-Antennary N-Glycans. *J Am Chem Soc* 147: 37357-37371

Lines 747-749: Weber J, Ponse NLD, Zhu X, Rios Carrasco M, Han AX, Funk M, Lin TH, Gabarroca Garcia A, Spruit CM, Zhang D *et al* (2026) The receptor binding properties of H5Ny influenza A viruses have evolved to bind to avian-type mucin-like O-glycans. *PLoS Pathog* 22: e1013812

- "The approval of the study protocol was obtained from the Ethics Committee of Beijing Ditan Hospital, Capital Medical University." Do you also have an approval number that could be added?

Response: Thank you for pointing this out. We have now added the approval number

(DTEC-KY2024-031-01) to the ethics statement, as you requested.

Lines 558-560: The study protocol was approved by the Ethics Committee of Beijing Ditan Hospital, Capital Medical University (approval number: DTEC-KY2024-031-01).

- BioRender should be acknowledged at the end of the Methods section in the following way:

Graphics:

(some of the... OR Figure #... OR synopsis) Graphics were created with BioRender.com.

Response: Thank you for specifying the required format. We have now included the BioRender acknowledgment at the end of the Methods section, exactly as you indicated.

Lines 586-587:

Graphics

Some of the graphical elements used in the synopsis figure were provided by BioRender.com.

- Data availability statement: Please add the specific URLs for datasets (PDB - 8X28, 8X2D, 8X26, 8X27, 8X2F, 8X29, 8X2C).

Response: Thank you for pointing this out. As requested, we have now added hyperlinked URLs for each PDB entry (8X28, 8X2C, 8X26, 8X27, 8X2F, 8X29, 8X2D) in the Data availability statement.

Lines 594-596: The structures of huH5N8 HA, swH5N8 HA, huH5N8 HA/LSTa, huH5N8 HA/LSTc, huH5N8 HA/SLe^{X}, swH5N8 HA/LSTa, and swH5N8 HA/LSTc have been deposited in the Protein Data Bank (PDB; www.rcsb.org) under accession numbers 8X28, 8X2C, 8X26, 8X27, 8X2F, 8X29, and 8X2D, respectively.

- Figure legends:

- Please note that the legends for figures EV-4; are not provided in the sequential manner (legend for figure EV-4D is provided before legend of figure EV-4B, C). This needs to be rectified.

Response: Thank you for pointing out the incorrect order. We have rectified the sequence so that the legend for EV-4B now appears before EV-4C, and EV-4C before EV-4D, as you requested.

Lines 854-857: Schematic representation of: (A) huH5N8 HA RBS with sticks representing key receptor-binding residues (colored in marine); (B) huH5N8 HA with LSTa bound; (C) huH5N8 HA with LSTc bound; (D) wsH5N8 HA RBS with sticks representing key receptor-binding residues (colored in lime); (E) wsH5N8 HA RBS with LSTa bound; and (F) wsH5N8 HA RBS with LSTc bound.

- Please note that the legend for figures 1G-J is missing in the manuscript. This needs to be rectified.

Response: Thank you for noting the omission. We have now added the legend for Figures 1G–J, which was previously missing, as you requested.

Lines 759-760: As controls, InH5 HA specifically binds 3'SLNLN (G-H), whereas H1N1 (I) and H3N2 (J) HA specifically bind 6'SLNLN.

- Please note that the error bars are not defined in the legends of figures 2A, B, C.

Response: Thank you for pointing out the missing definition. We have now added the definition of error bars to the legends of Figures 2A-C, as you requested.

Lines 764-765: Error bars represent half the difference in signal intensities between duplicate spots of each glycan probe.

- Please note that the white pentagrams are not defined in the legend of figures 3C, G, H. This needs to be rectified.

- Please note that the white triangles are not defined in the legend of figure 3G; This needs to be rectified.

Response: Thank you for noting these omissions. We have rectified the legends accordingly: white pentagrams are now defined in Figures 3C, 3G, and 3H, and white triangles are now defined in Figure 3G, as you requested.

Lines 773-781: (A-D) Staining of human conjunctiva (A), trachea (B), lung (C) and mammary gland tissue sections (D) with huH5N8, wsH5N8, TxH5N1, InH5, H1N1, H3N2 HA proteins and anti-SLe^{X} antibodies. White triangles indicate goblet cells in (A) and (B); white pentagrams indicate conjunctival epithelium in (A), respiratory epithelium in (B), alveolar epithelium in (C), and mammary glandular epithelium in (D). (E-F) Staining of bovine trachea (E) and mammary gland (F) with the same HA proteins and anti-SLe^{X} antibodies. White pentagrams indicate respiratory epithelium in (E), and mammary glandular epithelium in (F). (G-H) Staining of duck trachea (G) and intestine (H) with the same HA proteins and anti-SLe^{X} antibodies. White triangles indicate goblet cells in (G); white pentagrams indicate respiratory epithelium in (G) and intestinal epithelium in (H).

- I think the title should define H5 as avian influenza virus to make it more accessible to the general readership.

Response: We appreciate your suggestion to define H5 as avian influenza virus to make the title more accessible. However, the journal imposes a strict character limit on titles, and we believe the original title is already clear within that constraint. Adding any further wording would exceed the limit. Therefore, we propose keeping the original title. For reference, an alternative title "Hemagglutinin double-mutation enhances binding of human-infecting avian influenza virus clade 2.3.4.4b H5Ny to human and SLe^{X} receptors" is suggested with 35 more characters over the allowed limit.

Prof. George Gao
Institute of Microbiology,CAS
CAS Key Laboratory of Pathogen Microbiology and Immunology
No. 1 Beichen West Road,Chaoyang District
Beijing, Beijing 100101
China

Dear George,

Thank you very much for implementing the final minor changes. I am very pleased to accept your manuscript for publication in the next available issue of EMBO reports. Thank you for your contribution to our journal.

Your manuscript will be processed for publication by EMBO Press. It will be copy edited and you will receive page proofs prior to publication. Please note that you will be contacted by Springer Nature Author Services to complete licensing and payment information.

You may qualify for financial assistance for your publication charges - either via a Springer Nature fully open access agreement or an EMBO initiative. Check your eligibility: https://link.springer.com/journal/44319/how-to-publish-with-us

Should you be planning a Press Release on your article, please notify the production team via this form in order to coordinate publication and release dates: https://forms.cloud.microsoft/e/eH4A8JVP6g

If you have any questions, please do not hesitate to contact the Editorial Office. Thank you for your contribution to EMBO Reports.

Kind regards,

Martina

Martina Rembold, PhD
Senior Editor
EMBO reports

>>> Please note that it is EMBO Reports policy for the transcript of the editorial process (containing referee reports and your response letter) to be published as an online supplement to each paper. If you do NOT want this, you will need to inform the Editorial Office via email immediately. More information is available here: https://link.springer.com/partners/embo-press/editorial-policies#Peer%20review

EMBO Press Author Checklist

Corresponding Author Name: George F. Gao(高福)	
Journal Submitted to: EMBO Reports	
Manuscript Number: EMBOR-2025-63394-T	

USEFUL LINKS FOR COMPLETING THIS FORM
The EMBO Journal - Author Guidelines
EMBO Reports - Author Guidelines
Molecular Systems Biology - Author Guidelines
EMBO Molecular Medicine - Author Guidelines

Reporting Checklist for Life Science Articles (updated January
This checklist is adapted from Materials Design Analysis Reporting (MDAR) Checklist for Authors. MDAR establishes a minimum set of requirements in transparent reporting in the life sciences (see Statement of Task: 10.31222/osf.io/9sm4x). Please follow the journal's guidelines in preparing your
Please note that a copy of this checklist will be published alongside your article.

Abridged guidelines for figures
1. Data
The data shown in figures should satisfy the following conditions:
- the data were obtained and processed according to the field's best practice and are presented to reflect the results of the experiments in an accurate and unbiased manner.
- ideally, figure panels should include only measurements that are directly comparable to each other and obtained with the same assay.
- plots include clearly labeled error bars for independent experiments and sample sizes. Unless justified, error bars should not be shown for technical
- if n<5, the individual data points from each experiment should be plotted. Any statistical test employed should be justified.
- Source Data should be included to report the data underlying figures according to the guidelines set out in the authorship guidelines on Data

2. Captions
Each figure caption should contain the following information, for each panel where they are relevant:
- a specification of the experimental system investigated (eg cell line, species name).
- the assay(s) and method(s) used to carry out the reported observations and measurements.
- an explicit mention of the biological and chemical entity(ies) that are being measured.
- an explicit mention of the biological and chemical entity(ies) that are altered/varied/perturbed in a controlled manner.
- the exact sample size (n) for each experimental group/condition, given as a number, not a range;
- a description of the sample collection allowing the reader to understand whether the samples represent technical or biological replicates (including how many animals, litters, cultures, etc.).
- a statement of how many times the experiment shown was independently replicated in the laboratory.
- definitions of statistical methods and measures:
  - common tests, such as t-test (please specify whether paired vs. unpaired), simple χ2 tests, Wilcoxon and Mann-Whitney tests, can be unambiguously identified by name only, but more complex techniques should be described in the methods section;
  - are tests one-sided or two-sided?
  - are there adjustments for multiple comparisons?
  - exact statistical test results, e.g., P values = x but not P values < x;
  - definition of 'center values' as median or average;
  - definition of error bars as s.d. or s.e.m.

Please complete ALL of the questions below.
Select "Not Applicable" only when the requested information is not relevant for your study.

Materials

Newly Created Materials	Information included in the manuscript?	In which section is the information available? (Reagents and Tools Table, Materials and Methods, Figures, Data Availability Section)
New materials and reagents need to be available; do any restrictions apply?	Not Applicable	

Antibodies	Information included in the manuscript?	In which section is the information available? (Reagents and Tools Table, Materials and Methods, Figures, Data Availability Section)
For **antibodies** provide the following information:<br>- Commercial antibodies: RRID (if possible) or supplier name, catalogue number and or/clone number<br>- Non-commercial: RRID or citation	Yes	Resgents and Tools Table

DNA and RNA sequences	Information included in the manuscript?	In which section is the information available? (Reagents and Tools Table, Materials and Methods, Figures, Data Availability Section)
**Short novel DNA or RNA including primers, probes**: provide the sequences.	Not Applicable	

Cell materials	Information included in the manuscript?	In which section is the information available? (Reagents and Tools Table, Materials and Methods, Figures, Data Availability Section)
**Cell lines:** Provide species information, strain. Provide accession number in repository **OR** supplier name, catalog number, clone number, and/**OR** RRID.	Yes	Resgents and Tools Table
**Primary cultures:** Provide species, strain, sex of origin, genetic modification status.	Not Applicable	
Report if the cell lines were recently **authenticated** (e.g., by STR profiling) and tested for mycoplasma contamination.	Not Applicable	

Experimental animals	Information included in the manuscript?	In which section is the information available? (Reagents and Tools Table, Materials and Methods, Figures, Data Availability Section)
**Laboratory animals or Model organisms:** Provide species, strain, sex, age, genetic modification status. Provide accession number in repository **OR** supplier name, catalog number, clone number, **OR** RRID.	Not Applicable	
**Animal observed in or captured from the field:** Provide species, sex, and age where possible.	Not Applicable	
Please detail **housing and husbandry conditions**.	Not Applicable	

Plants and microbes	Information included in the manuscript?	In which section is the information available? (Reagents and Tools Table, Materials and Methods, Figures, Data Availability Section)
**Plants:** provide species and strain, ecotype and cultivar where relevant, unique accession number if available, and source (including location for collected wild specimens).	Not Applicable	
**Microbes:** provide species and strain, unique accession number if available, and source.	Not Applicable	

Human research participants	Information included in the manuscript?	In which section is the information available? (Reagents and Tools Table, Materials and Methods, Figures, Data Availability Section)
If collected and within the bounds of privacy constraints report on age, sex and gender or ethnicity for all study participants.	Not Applicable	

Core facilities	Information included in the manuscript?	In which section is the information available? (Reagents and Tools Table, Materials and Methods, Figures, Data Availability Section)
If your work benefited from core facilities, was their service mentioned in the acknowledgments section?	Yes	Acknowledgements

Design

Study protocol	Information included in the manuscript?	In which section is the information available? (Reagents and Tools Table, Materials and Methods, Figures, Data Availability Section)
If study protocol has been **pre-registered, provide DOI in the manuscript**. For clinical trials, provide the trial registration number **OR** cite DOI.	Not Applicable	
Report the **clinical trial registration number** (at ClinicalTrials.gov or equivalent), where applicable.	Not Applicable	

Laboratory protocol	Information included in the manuscript?	In which section is the information available? (Reagents and Tools Table, Materials and Methods, Figures, Data Availability Section)
Provide DOI OR other citation details if **external detailed step-by-step protocols** are available.	Not Applicable	

Experimental study design and statistics	Information included in the manuscript?	In which section is the information available? (Reagents and Tools Table, Materials and Methods, Figures, Data Availability Section)
Include a statement about **sample size** estimate even if no statistical methods were used.	Not Applicable	
Were any steps taken to minimize the effects of subjective bias when allocating animals/samples to treatment (e.g. **randomization procedure**)? If yes, have they been described?	Not Applicable	
Include a statement about **blinding** even if no blinding was done.	Not Applicable	
Describe **inclusion/exclusion criteria** if samples or animals were excluded from the analysis. Were the criteria pre-established?<br><br>If sample or data points were omitted from analysis, report if this was due to attrition or intentional exclusion and provide justification.	Not Applicable	
For every figure, are **statistical tests** justified as appropriate? Do the data meet the assumptions of the tests (e.g., normal distribution)? Describe any methods used to assess it. Is there an estimate of variation within each group of data? Is the variance similar between the groups that are being statistically compared?	Not Applicable	

Sample definition and in-laboratory replication	Information included in the manuscript?	In which section is the information available? (Reagents and Tools Table, Materials and Methods, Figures, Data Availability Section)
In the figure legends: state number of times the experiment was **replicated** in laboratory.	Yes	Figures
In the figure legends: define whether data describe **technical or biological replicates**.	Yes	Figures

Ethics

Ethics	Information included in the manuscript?	In which section is the information available? (Reagents and Tools Table, Materials and Methods, Figures, Data Availability Section)
Studies involving **human participants**: State details of **authority granting ethics approval** (IRB or equivalent committee(s), provide reference number for approval.	Not Applicable	
Studies involving **human participants**: Include a statement confirming that **informed consent** was obtained from all subjects and that the experiments conformed to the principles set out in the WMA Declaration of Helsinki and the Department of Health and Human Services Belmont Report.	Not Applicable	
Studies involving **human participants:** For publication of **patient photos**, include a statement confirming that consent to publish was obtained.	Not Applicable	
Studies involving experimental **animals**: State details of **authority granting ethics approval** (IRB or equivalent committee(s), provide reference number for approval. Include a statement of compliance with ethical regulations.	Not Applicable	
Studies involving **specimen and field samples:** State if relevant **permits** obtained, provide details of authority approving study; if none were required, explain why.	Yes	the Ethics Committee of Beijing Ditan Hospital, Capital Medical University.

Dual Use Research of Concern (DURC)	Information included in the manuscript?	In which section is the information available? (Reagents and Tools Table, Materials and Methods, Figures, Data Availability Section)
Could your study fall under dual use research restrictions? Please check biosecurity documents and list of **select agents and toxins** (CDC): https://www.selectagents.gov/sat/list.htm	Not Applicable	
If you used a select agent, is the security level of the lab appropriate and reported in the manuscript?	Not Applicable	
If a study is subject to dual use research of concern regulations, is the name of the **authority granting approval and reference number** for the regulatory approval provided in the manuscript?	Not Applicable	

Reporting

The MDAR framework recommends adoption of discipline-specific guidelines, established and endorsed through community initiatives. Journals have their own policy about requiring specific guidelines and recommendations to complement MDAR.

Adherence to community standards	Information included in the manuscript?	In which section is the information available? (Reagents and Tools Table, Materials and Methods, Figures, Data Availability Section)
State if relevant guidelines or checklists (e.g., **ICMJE, MIBBI, ARRIVE, PRISMA**) have been followed or provided.	Not Applicable	
For **tumor marker prognostic studies,** we recommend that you follow the **REMARK** reporting guidelines (see link list at top right). See author guidelines, under 'Reporting Guidelines'. Please confirm you have followed these guidelines.	Not Applicable	
For **phase II and III randomized controlled trials**, please refer to the **CONSORT** flow diagram (see link list at top right) and submit the CONSORT checklist (see link list at top right) with your submission. See author guidelines, under 'Reporting Guidelines'. Please confirm you have submitted this list.	Not Applicable	

Data Availability

Data availability	Information included in the manuscript?	In which section is the information available? (Reagents and Tools Table, Materials and Methods, Figures, Data Availability Section)
Have **primary datasets** been deposited according to the journal's guidelines (see 'Data Deposition' section) and the respective accession numbers provided in the Data Availability Section?	Yes	The structures for huH5N8 HA, swH5N8 HA, huH5N8 HA/LSTa, huH5N8 HA/LSTc, huH5N8 HA/SLeX, swH5N8 HA/LSTa and swH5N8 HA/LSTc are deposited to Protein Data Bank (PDB, www.rcsb.org) with the accession numbers: 8X28, 8X2D, 8X26, 8X27, 8X2E, 8X29, 8X2C, respectively
Were **human clinical and genomic datasets** deposited in a public access-controlled repository in accordance to ethical obligations to the patients and to the applicable consent agreement?	Not Applicable	
Are **computational models** that are central and integral to a study available without restrictions in a machine-readable form? Were the relevant accession numbers or links provided?	Not Applicable	
If publicly available data were reused, provide the respective **data citations in the reference list**.	Not Applicable	
